# Multicatalysis-Enabled Multicomponent Reactions Generate a PTP1B Inhibitor

**DOI:** 10.1021/acscentsci.5c00041

**Published:** 2025-05-19

**Authors:** Taoda Shi, Yukai Li, Jiying Yang, Weining Weng, Mengchu Zhang, Jirong Shu, Yu Qian, Tianyuan Zhang, Wenhao Hu

**Affiliations:** † Guangdong Key Laboratory of Chiral Molecule and Drug Discovery, School of Pharmaceutical Sciences, 26469 Sun Yat-sen University, Guangzhou 510006, China; ‡ State Key Laboratory of Anti-Infective Drug Discovery and Development, Guangdong Provincial Key Laboratory of Chiral Molecule and Drug Discovery, School of Pharmaceutical Sciences, 26469 Sun Yat-sen University, Guangzhou 510006, China; § Guangdong Basic Research Center of Excellence for Functional Molecular Engineering, 26469 Sun Yat-sen University, Guangzhou 510006, China

## Abstract

Multicomponent reactions are powerful tools for expanding the chemical space in drug discovery, yet achieving selectivity remains a formidable challenge. Here, we introduce a multicatalytic strategy to enable a multicomponent reaction, utilizing a cooperative system of rhodium, copper, Brønsted acid, and magnesium catalysts. This approach achieves excellent chemo-, diastereo-, and enantioselectivity (up to 99% yield, >20:1 dr, and 99% ee). Mechanistic studies, combining experimental and computational analyses, reveal a cascade sequence involving cyclopropenation, desilylation, cyclization, isomerization, aldol addition, and hydrolysis. This highly selective method exhibits broad substrate generality, producing 50 diverse CHBOs. Virtual screening and rapid biological evaluation led to the discovery of (*S*, *S*)-**3ak**, a potent PTP1B inhibitor with a submicromolar IC_50_ value. Notably, (*S*, *S*)-**3ak** demonstrated 3-fold higher potency than its enantiomer, underscoring the critical role of chirality. Molecular docking studies elucidated the enantioselective binding mechanism, revealing key interactions responsible for activity differences. In summary, this MMCR strategy enables efficient access to enantiopure bioactive molecules and facilitates drug discovery, exemplified by a novel chiral PTP1B inhibitor.

## Graphical abstract

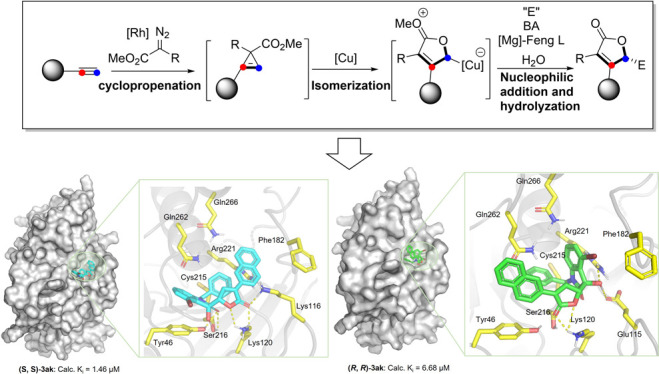

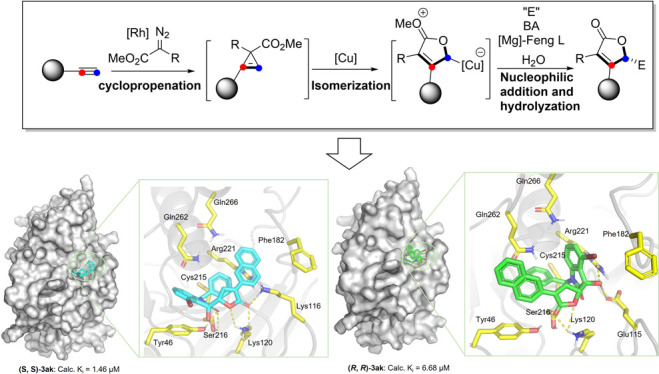

## Introduction

Multicomponent reactions (MCRs) are powerful tools for constructing molecular libraries in drug discovery.^1–4^ Compared to stepwise synthetic approaches, MCRs offer superior atom and step economy while enabling rapid generation of structurally diverse libraries (Figure 1a). Their value in medicinal chemistry has been demonstrated in the development of E3 ligase modulators,^5^ Nav1.7 inhibitors,^6^ and central nervous system-targeting molecules.^7^ Recently, our group established the MCR-based Readily Available Library (MREAL) via the capture of highly reactive intermediates with electrophiles, facilitating the discovery of bioactive molecules, including potential analgesics and anticancer reagents.^8–10^ Looking for bioactive molecules from MREAL, which selectively target pathologically important proteins, will be of longstanding significance in drug discovery. We here present an alternative application of the MREAL: the discovery of chiral hybrids of γ-butenolide and oxindoles (CHBOs) as potent PTP1B inhibitors via the development of multicatalysis-enabled MCRs (MMCRs) of diazoacetates, akynes, water, and isatins.

**Figure 1:**
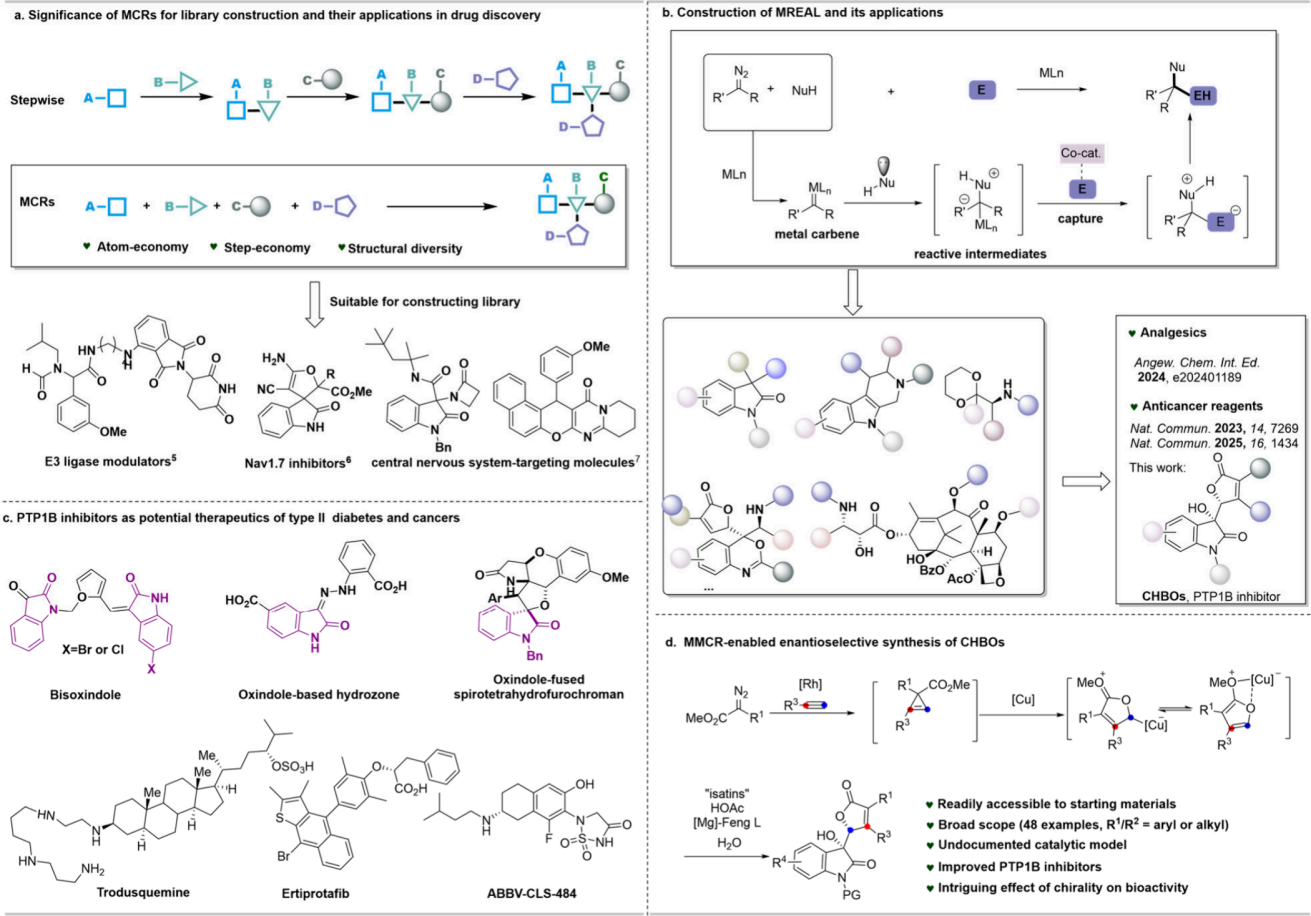
Significance and proposal of development of MMCR for the discovery of PTP1B inhibitors.

Oxindole-derived molecules possess diverse pharmacological activities,^11–13^ making them highly relevant in drug discovery. Notably, they have been identified as protein tyrosine phosphatase 1B (PTP1B) inhibitors (Figure 1c), a promising class of compounds for Type II diabetes and cancer treatment.^14–16^ Despite the clinical advancement of PTP1B inhibitors such as trodusquemine^17^ and ertiprotafib,^18^ their development stalled due to limited efficacy (Figure 1c). More recently, ABB-CLS-484, a dual PTP1B/TCPTP inhibitor, progressed to Phase II clinical trials as an immunotherapeutic.^19,20^ Given the growing interest in PTP1B inhibitors for cancer immunotherapy, the continued discovery of new potent drug-like scaffolds remains essential.

To this end, we screened the MREAL database with around 3000 scaffold-diversified molecles against PTP1B via molecular docking, prioritizing compounds with docking scores Δ*G* < −7.0 kcal/mol and reasonable binding pose. This led to the identification of four promising scaffold classes: 2,5-dihydrofurans (2HF), tetrahydrocarbolines (THCB), oxindole-branch (OB), and *rac*-CHBOs (Scheme S6). It is well-known that the stereochemistry of small molecules usually plays a significant role in interacting with protein targets. We are wondering if the chirality of CHBOs affects their inhibitory activity against PTP1B. This motivated us to develop asymmetric catalytic synthesis of CHBOs.

However, existing asymmetric syntheses of CHBOs are limited to two reported examples, either metal-^21^ or organocatalyzed.^22^ These methods suffer from a narrow substrate scope and rely on γ-lactone precursors that are not readily available, restricting their utility in rapidly generating diverse screening libraries. Based on our previous study on aldol-type addition to isatins via transiently stable nucleophiles,^9,23,24^ we here designed a multicatalysis-enabled multicomponent reaction (MMCR) to efficiently construct CHBOs with excellent chemo-, diastereo-, and enantioselectivity, while maintaining broad substrate scope (Figure 1d).

The reaction mechanism and catalytic model of this MMCR were investigated through a combination of experimental and computational studies, revealing a complex cascade sequence involving cyclopropenation, desilylation, cycloisomerization, aldol-type addition, and hydrolysis, orchestrated by trimetal/organo relay catalysis. This well-established MMCR provides a powerful platform for synthesizing CHBOs, paving the way for the discovery of potent PTP1B inhibitors and contributing to the advancement of PTP1B-based cancer immunotherapies.

## Results and Discussion

### Development of Asymmetric Multifunctionalization of Alkynes via a Four-Component Cascade

First, we intend to search for a catalyst system to control the chemo- and stereoselectivity in the zwitterion capture process. We started from the reaction of cyclopropene carboxylic acid (CCA) and isatins, which gave good diastereoselectivity in our previous work.^23^ However, enantiocontrol of the reaction went nowhere after we tried many chiral catalysts. The main reason for the difficulty is the strong background reaction, which could finish in a minute. The DFT study revealed that CCA itself as a Brønsted acid catalyst can promote the aldol-type addition to isatin, rendering *anti*-selectivity (Figure 2). The double H-bonding interactions in the transition state may dictate the diasteroselectivity. Based on the observation, we decide to use cyclopropene carboxylate ester (CCE) as a substrate to slow down the rate of background reaction. Not surprisingly, the reaction slowed down sharply and finished in 1 h but gave *syn*-**3a** as major product. The DFT calculations indicate the TS from CCE could not form a bifunctional activation model like the TS from CCA, resulting in much lower reactivity than CCA. And the fact could be further supported by the competition experiment between CCA and CCE, which gave the similar diastereoselectivity to the CCA alone. Therefore, we decided to choose less reactive CCE as the substrate for condition optimization.

**Figure 2:**
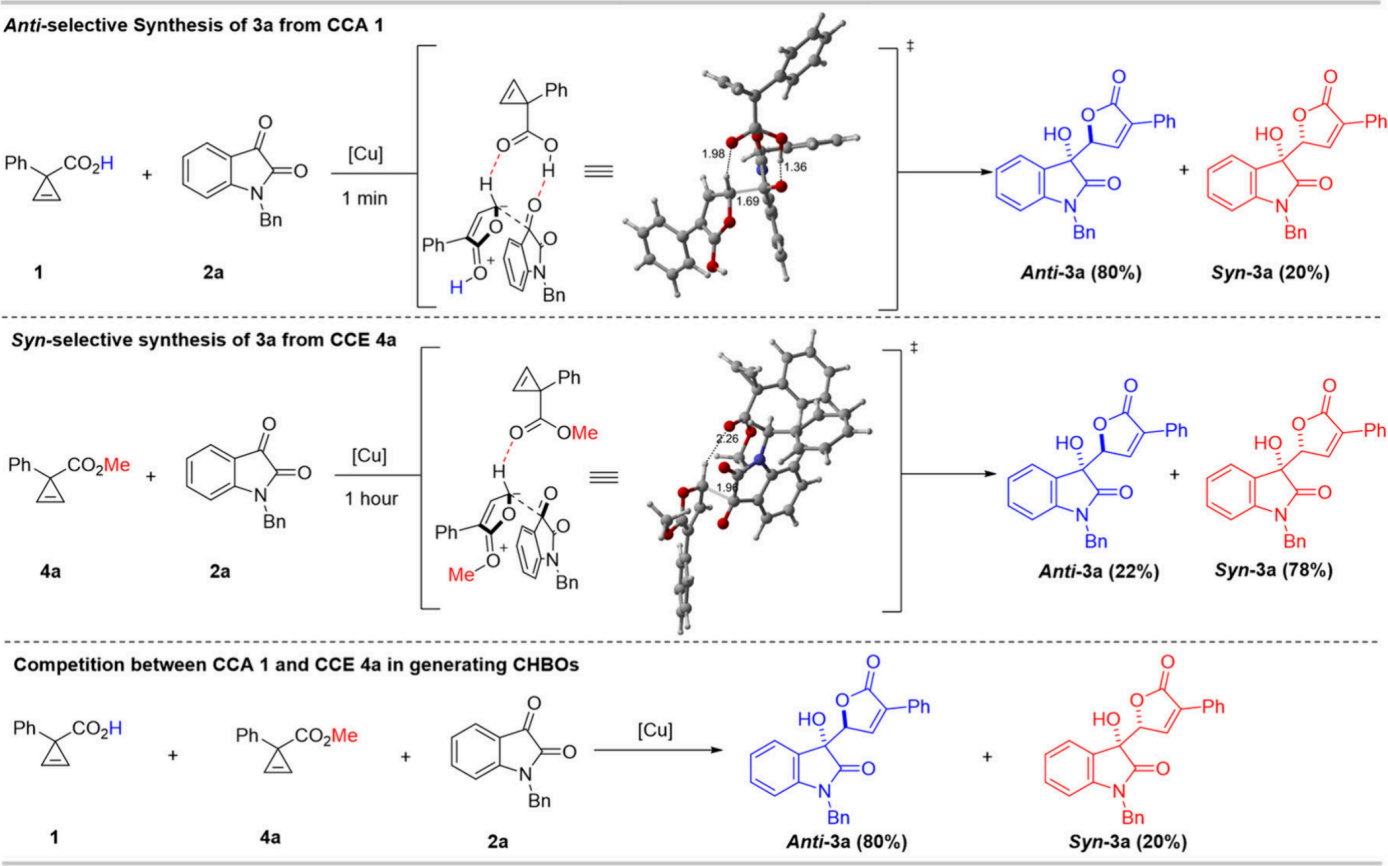
Rational selection of substrate for the catalytic enantioselective synthesis of CHBOs.

Next, the reaction of CCE **4a** and isatin **2a** was set as the template reaction for screening optimal conditions. First, chiral copper complexes are expected to be capable for enantiocontrol in the isatins or their derivatives-involved reactions.^25–28^ Therefore, a panel of ligands including bisoxazolines (L1-L2),^29,30^ Binol (L4),^31^ Trost-type bisamides (L3 and L5),^32^ and Feng ligands (L7-L10)^33,34^ were evaluated. These results showed ligands with H-bonding donors like Feng’s ligand gave higher reactivities (Table 1, entries 1 vs entry 2–3). Based on the ligand–metal match model of Feng’s ligand, chiral manganese complexes are usually efficient in the aldol-type reactions. Mg–ligand complexes may outcompete Cu–ligands in interacting and activating carbonyl groups according to hard-soft acid–base (HSAB) theory. Therefore, Mg­(OTf)_2_ was used as a cocatalyst resulting in improvement of reactivity and selectivity (Table 1, entry 3 vs entry 4). Then three other Feng ligands **L8**–**L10** were evaluated in the cocatalyst system, giving **L10** as the optimal ligand with 77% yield, 18:82 dr, and 70% ee for the major diastereoisomer (Table 1, entry 5–7). Next, **L10** was chosen for the screening of combinations of metal catalysts. Mg­(ClO_4_)_2_ turned out to be a better cocatalyst than Mg­(OTf)_2_ (Table 1, entries 7 and 8). Therefore, Mg­(ClO_4_)_2_ was selected to test a group of transition metal catalysts, resulting in CuBF_4_(CH_3_CN)_4_ as the best partner (Table 1, entries 8–10). Then the ratio of the two cocatalysts was adjusted and 5 mol % Mg­(ClO_4_)_2_ and 10 mol % CuBF_4_(CH_3_CN)_4_ was identified as the best match, with 96% yield, 22:78 dr, and 71% ee for major diastereoisomers in 4 h (Table 1, entry 11). With the cocatalyst match being set, a group of solvents was then screened and ethyl butyrate was identified as the best matched solvent, giving 92% yield, 28:72 dr, 73% ee (major), and 70% ee (minor) (Table 1, entries 11–18). Proton-donor solvents like MeOH reduce catalytic efficiency significantly (Table 1, entry 17). This is probably due to the strong coordination between MeOH and Mg­(ClO_4_)_2_. Lastly, additives are known to be crucial to stereocontrol in asymmetric catalysis. Therefore, a panel of additives were tested in the reaction (Table 1, entries 19–25). Interestingly, the reaction was totally inhibited if 4 Å molecular sieves was added, indicating that water may be essential to the reaction (Table 1, entry 19). The other H-bonding donors including alcohol, acetic acid (HOAc), formic acid, and trifluoracetic acid could boost the reaction, and HOAc gave the optimal results, with 90% yield, 28:72 dr, 90% ee (major), and 90% ee (minor) (Table 1, entry 25). Overall, the recipe of the reaction was established as “5 mol % Mg­(ClO_4_)_2_, 10 mol % CuBF_4_(CH_3_CN)_4_, 5 mol % **L10**, 10 mol % HOAc, in ethyl butyrate, 4 h” (Table 1, entry 25).

**Table 1:**
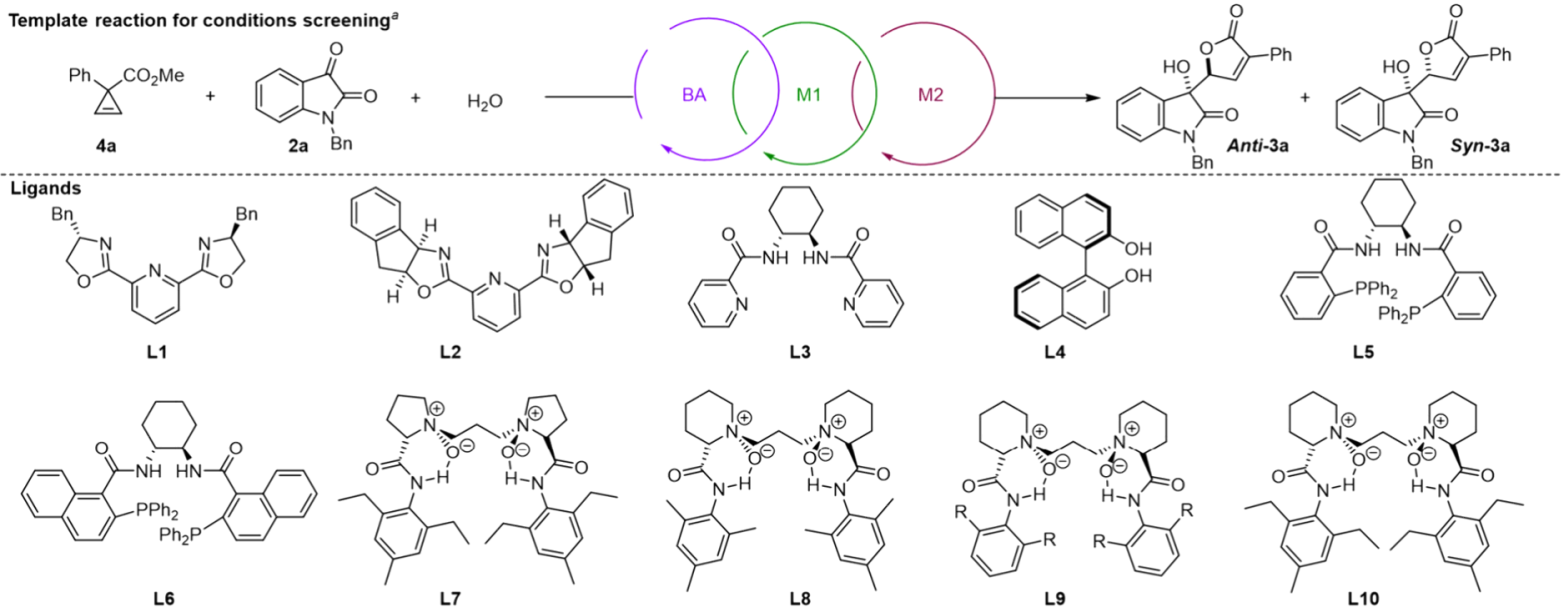
Condition Screening of Catalytic Asymmetric Reaction of CCE **4a** and Isatin **2a** ^a^

entry	[M1]	[M2]	ligand	organocatalyst	solvent	time (h)	yield (%)^b^	ee (%)^c^	dr^i^ (syn:anti)^d^
1	Cu(OTf)_2_	/	**L1**, **L2**, or **L3**	/	EtOAc	48	<5	**/**	**/**
2	Cu(OTf)_2_	/	**L6**	/	EtOAc	48	44	0/0	80:20
3	Cu(OTf)_2_	/	**L7**	/	EtOAc	48	62	18/22	79:21
4	Cu(OTf)_2_	Mg(OTf)_2_	**L7**	/	EtOAc	12	68	44/32	87:13
5	Cu(OTf)_2_	Mg(OTf)_2_	**L8**	/	EtOAc	12	93	36/48	80:20
6	Cu(OTf)_2_	Mg(OTf)_2_	**L9**	/	EtOAc	12	58	71/44	90:10
7	Cu(OTf)_2_	Mg(OTf)_2_	**L10**	/	EtOAc	12	77	70/10	82:18
8	Cu(OTf)_2_	Mg(ClO_4_)_2_	**L10**	/	EtOAc	12	92	68/62	83:17
9	Cu(MeCN)_4_PF_6_	Mg(ClO_4_)_2_	**L10**	/	EtOAc	12	90	50/48	83:17
10	CuTc	Mg(ClO_4_)_2_	**L10**	/	EtOAc	12	59	0/12	64:36
11	Cu(MeCN)_4_BF_4_	Mg(ClO_4_)_2_	**L10**	/	EtOAc	4	96	71/66	78:22
12	Cu(MeCN)_4_BF_4_	Mg(ClO_4_)_2_	**L10**	/	THF	4	97	44/42	81:19
13	Cu(MeCN)_4_BF_4_	Mg(ClO_4_)_2_	**L10**	/	MTBE	4	50	32/40	79:21
14	Cu(MeCN)_4_BF_4_	Mg(ClO_4_)_2_	**L10**	/	xylene	4	43	26/26	76:24
15	Cu(MeCN)_4_BF_4_	Mg(ClO_4_)_2_	**L10**	/	toluene	4	48	41/40	77:23
16	Cu(MeCN)_4_BF_4_	Mg(ClO_4_)_2_	**L10**	/	DCE	4	76	44/36	82:18
17	Cu(MeCN)_4_BF_4_	Mg(ClO_4_)_2_	**L10**	/	MeOH	4	45	25/4	53:47
18	Cu(MeCN)_4_BF_4_	Mg(ClO_4_)_2_	**L10**	/	ethyl butyrate	4	92	73/70	78:22
19	Cu(MeCN)_4_BF_4_	Mg(ClO_4_)_2_	**L10**	4 Å MS	ethyl butyrate	4	0	/	/
20	Cu(MeCN)_4_BF_4_	Mg(ClO_4_)_2_	**L10**	EtOH	ethyl butyrate	4	97	73/70	72:28
21	Cu(MeCN)_4_BF_4_	Mg(ClO_4_)_2_	**L10**	AcOH	ethyl butyrate	4	90	90/90	78:22
22	Cu(MeCN)_4_BF_4_	Mg(ClO_4_)_2_	**L10**	TFA	ethyl butyrate	4	65	90/89	70:30
23	Cu(MeCN)_4_BF_4_	Mg(ClO_4_)_2_	**L10**	HCOOH	ethyl butyrate	4	78	90/90	71:29
24	Cu(MeCN)_4_BF_4_	Mg(ClO_4_)_2_	**L10**	TsOH	ethyl butyrate	4	90	78/79	73 27
**25**	**Cu(MeCN)**_**4**_ **BF**_**4**_	**Mg(ClO**_**4**_ **)**_**2**_	**L10**	**AcOH**	**ethyl butyrate**	**4**	**83**	90/91	63:34

a Unless otherwise indicated, the reactions were all on the scale of 0.05 mmol with 1 mL of solvent, **4a**:**2a** = 1.5:1, [M1] = 10 mol %, [M2] = 5 mol %, ligand = 5 mol %, additive = 5 mol %.

b Determined by ^1^H NMR analysis of the crude products based on internal standard (1,3,5-trimethoxybenzene).

c Determined by HPLC analysis using a chiral stationary phase.

d Determined by ^1^H NMR analysis of the crude products.

Then we asked if we could use *in situ* generated CCE from diazoesters and alkynes. We assumed that the silylated CCE could be deprotected by the multicatalyst system and be activated for the upcoming cycloisomerization/aldol-type addition/hydrolyzation cascade, and the rhodium catalyst is compatible in the downstream process. Gladly, the masked cyclopropene by trimethylsilyl group could be activated by the multicatalyst system, and the reactivity and selectivity are not influenced under the cascade scenario (Figure 3, row 6). Though the synthesis of cyclopropenes is well-established by the Davies group via the cyclopropenation between alkynes and carbenoids,^35–39^ 2–3 step transformations are usually needed to get the isolated cyclopropenes, which are used as versatile substrates in many useful reactions, including carbene transfer reactions,^40–42^ photoclick reactions,^43^ [4 + 2] annulation,^44,45^ carbozincation,^46^ magnesiation,^47^ C–H alkenylation and allylation,^48^ and ring expansion/[3,3]-sigmatropic rearrangements.^49^ We here successfully merged the generation of reactive cyclopropenes from alkynes with the utilization of cyclopropenes together, developing a straightforward method of functionalizing alkynes bridged by cyclopropene chemistry.

**Figure 3:**
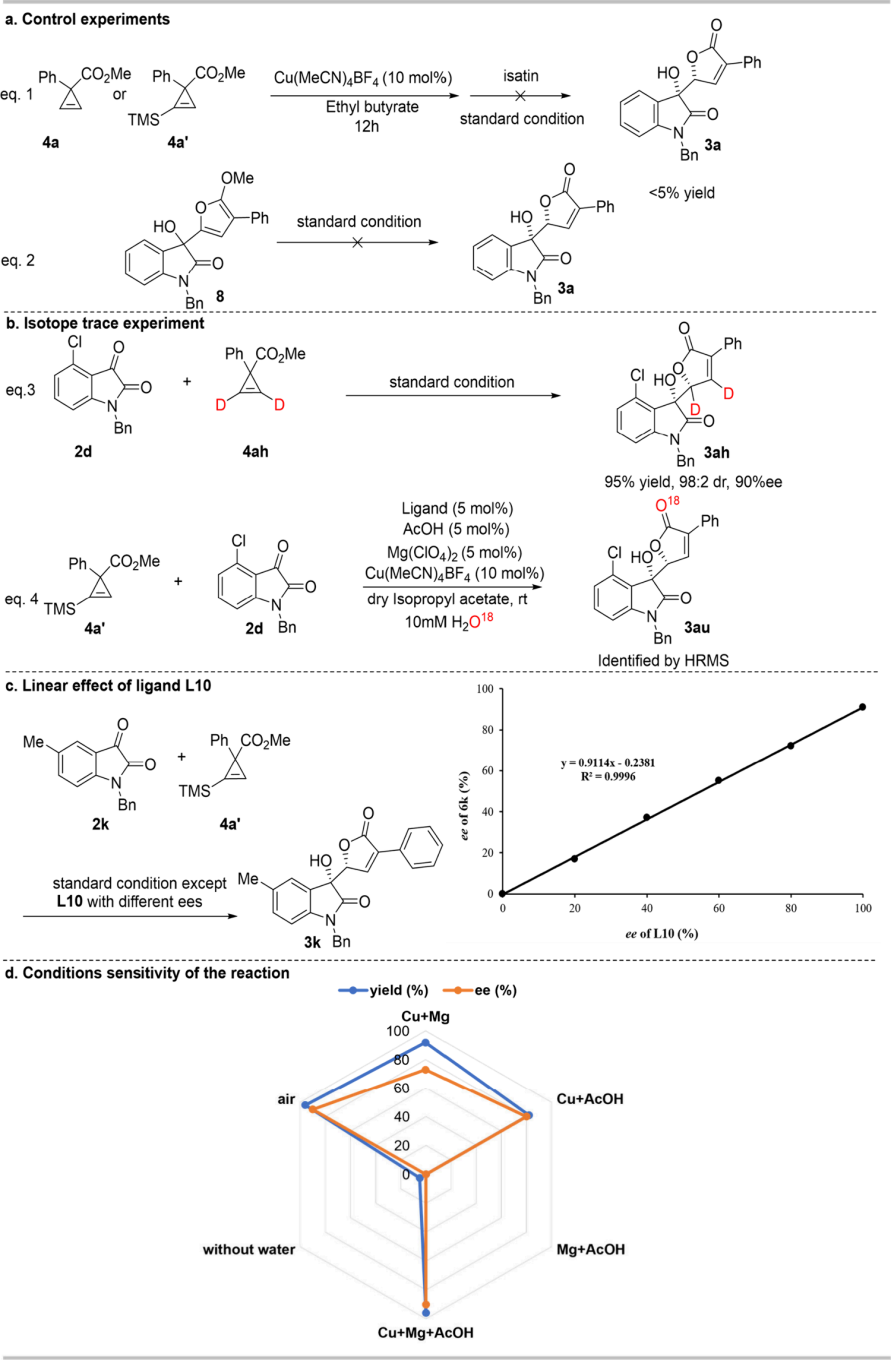
Mechanistic investigation of the MMCR. (a) Control experiment; (b) isotope trace experiment; (c) linear effect of chiral ligand; and (d) condition sensitivity of the MMCR.

### Mechanism Investigation

We then aimed to elucidate the mechanism of MMCR of diazoesters, alkynes, isatins, and water. First, we asked whether the cycloisomerization/aldol-type addition of cyclopropenes and isatins proceeds via a stepwise or concerted pathway. Altering the feeding sequence to sequentially add Cu­(MeCN)_4_BF_4_, Mg­(ClO_4_)_2_-**L10**, and isatin resulted in no CHBO **3a** (Figure 3a), suggesting the process goes through a concerted pathway instead of a stable intermediate.

Next, we asked if the reaction goes through intermediate **8** (Figure 4a), previously observed in the photopromoted cascade reaction between cyclopropene and isatin.^9^ Testing this intermediate under current asymmetric catalytic conditions yielded no desired CHBO **3a** (Figure 3b, eq 2). And the jugement. This conclusion could be further supported by an isotope trace experiment which showed no cleavage of the C–D bond during the reaction, indicating that intermediate **8** does not participate in the reaction pathway (Figure 3b, eq 3, and Figures S1 and S2).

**Figure 4:**
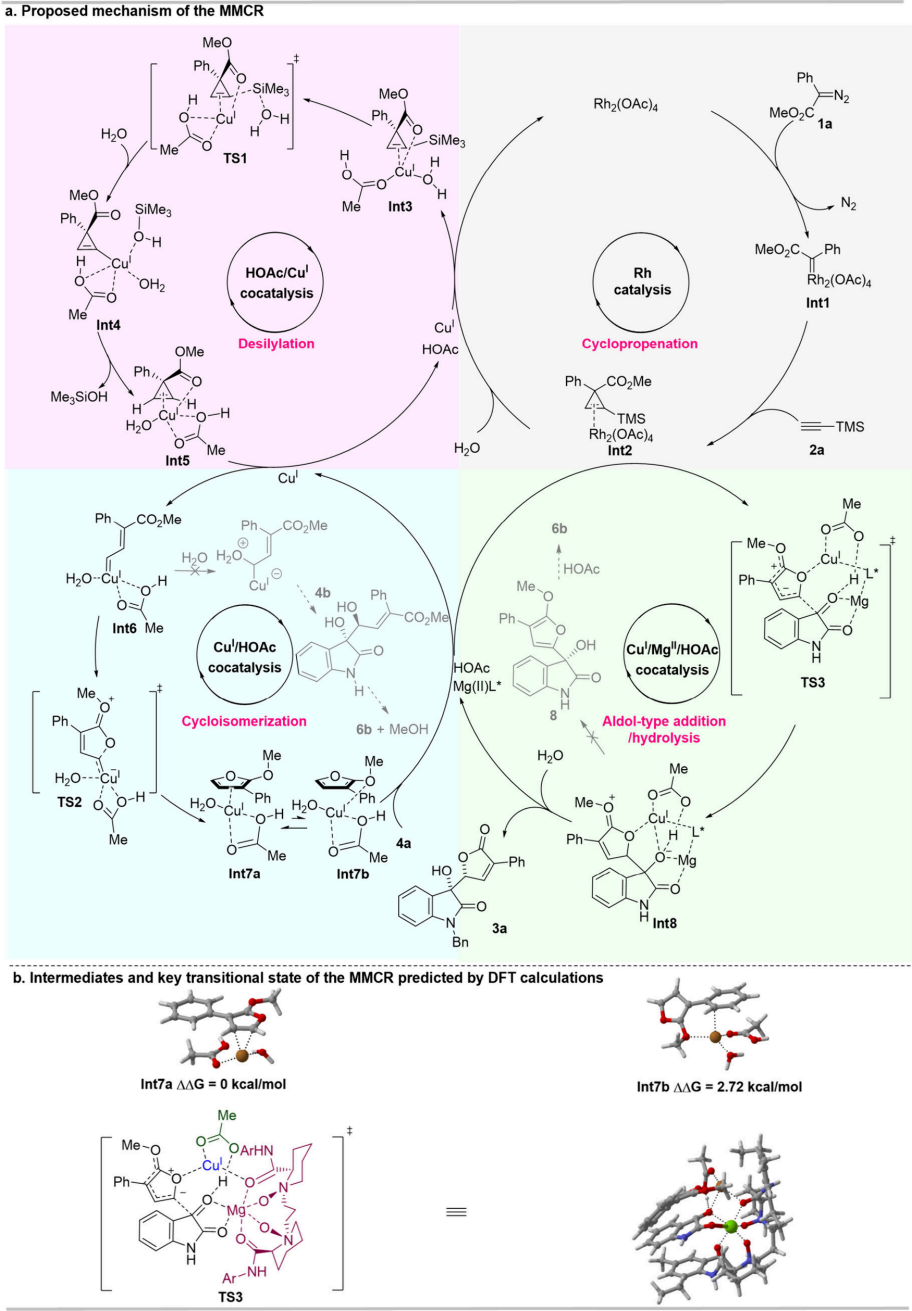
Proposed mechanism of the MMCR of diazoesters, alkynes, water, and isatins and the predicted TS and intermediates by DFT calculations.

Subsequently, we asked how water participates in the MMCR. O^18^-labeled water was introduced in the reaction, and O^18^ was detected in the carbonyl group of the lactone, suggesting water is involved in the hydrolysis process instead of oxonium-ylide formation (Figure 3b, eq 4, and Figure S3). The excess water in the reaction mixture does not affect the yield or enantioselectivity until it reaches a level where it becomes the solvent. At that point, phase separation occurs, isolating the catalysts and substrates into different phases and resulting in no desired product.

Next, we examined the possible form of the Mg/Feng ligand complex in the key TS via exploration of the relationship between the ee of ligands and the ee of the product. It turned out to be a linear relationship between the ee of Feng ligand and the ee of the product (Figure 3c and Figure S4). The results indicate the involvement of a monomeric Mg/Feng ligand complex in the TS.^50,51^

Then we asked if the cooperation of the Mg/Feng ligand complex, HOAc, and CuPF_6_(CH_3_CN)_4_ is essential to the reactivity and selectivity. The control experiment showed that after elimination of either catalyst, the reactivity and enantioselectivity dropped sharply and the best results were achieved when these catalysts were working together (Figure 3d). The radar graph also implicated that the reaction is robust in the air and moisture.

Based on mechanistic experiments, we propose a possible pathway for the MMCR. As shown in Figure 4a, the reactive rhodium-carbenoid intermediate (**INT1**) reacts with trimethylsilylacetylene to form a relatively stable rhodium-associated cyclopropene intermediate (**INT2**). Upon the addition of the copper catalyst and water, **INT2** rapidly converts into **INT3** via ligand exchange. **INT3** subsequently undergoes desilylation through transition state **TS1**, yielding **INT4**. During this process, the copper catalyst plays a dual role. On one hand, it weakens the C–Si bond by coordinating with the C–C double bond. On the other hand, it facilitates nucleophilic attack by bringing water into proximity to the silyl group. **INT4** is then protonated to form **INT5**, which is unstable and immediately undergoes cycloisomerization via copper-carbenoid **INT6**. The formation of **INT7a** occurs through an intramolecular nucleophilic attack by the carbonyl group of the ester on the copper-carbenoid center. The zwitterionic intermediate **INT7a** can isomerize into the enolate form of **INT7b**. With the involvement of isatin and a magnesium catalyst, **INT8** is rapidly generated through the transition state **TS3**. Ultimately, CHBO **3a** is obtained after the hydrolysis of **INT8**.

A total of eight intermediates (INTs) and three transition states (TSs) were predicted through density functional theory (DFT) calculations (Figure 4b and Table S11). Among these, **INT7a** is energetically more stable than **INT7b** by 2.72 kcal/mol (Figure 4b), suggesting that the copper−π complex is a more favorable intermediate than the copper−σ complex. **TS3** represents the proposed model for stereocontrol, where the catalysts, including HOAc, Cu­(I), and Mg­(II), establish a stable interaction network through hydrogen bonding and σ- or π-coordination.

The multicatalyst system, comprising rhodium, copper, and magnesium catalysts along with HOAc, functions sequentially and synergistically to promote the cyclopropenation/desilylation/cycloisomerization/aldol-type addition/hydrolysis cascade. This system exemplifies precise control over chemo-, diastereo-, and enantioselectivity in the MMCR. The rhodium catalyst facilitates cyclopropenation between diazoesters and alkynes but does not influence the selectivities of the subsequent transformations. The copper catalyst plays three key roles: activating the C–Si bond for desilylation, promoting cycloisomerization via cyclopropene activation, and synergistically facilitating zwitterion activation for the aldol-type addition. The chiral magnesium/Feng ligand catalyst, as previously reported, is a robust system for activating carbonyl compounds such as isatin. Additionally, HOAc exhibits bifunctionality in the MMCR, serving as both a ligand for copper and a Brønsted acid that promotes desilylation, protonation, and aldol-type addition. Overall, this multicatalyst system is precisely orchestrated to efficiently control the chemo-, diastereo-, and enantioselectivity of the MMCR. The concept of multicatalysis demonstrated in this work provides valuable insights for designing new multicomponent reactions, particularly those mediated by reactive intermediates, such as carbenes or zwitterions.

### Generality of the MMCR

We then started to investigate the substrate scope of the MMCR of diazoesters, alkynes, isatin, and water. To begin with, various isatins were evaluated by tuning the substituents at the 1-, 4-, 5-, 6-, and 7-positions. As to substituents at the 1-position, the benzyl group gave better enantioselectivity than the hydrogen and methyl groups, while the reactivities and diastereoselectivities are similar to each other. Interestingly, the istatins with substitutents at the 4-position gave excellent yields and excellent diastereoselectivities (Figure 5, **3d**–**3i**, >20:1 dr). The enantioselectivities were also excellent except **3h**, which also has a chloro at the 7-position (91–99% ee for **3d**–**3g** and **6i**, 80% ee for **3h**). Then isatins with halide, electron-donating group (EDG), and electron withdrawing group (EWG) substituents at the 5-position were evaluated, with halide and EDG giving excellent ee’s and good dr’s and EWG giving slightly lower stereoselectivity (Figure 5, **3j**–**3m**). This is probably because the electrophilicity of the carbonyl group of isatin is increased by the NO_2_ group and makes the stain highly reactive under the racemic pathway. Then isatins with substituents at the 6- and 7-positions were tested, resulting in 88–99% yields, 62:38–90:10 dr’s, and 88–92 ee’s (Figure 5, **3n**–**3r**). Finally, the reaction could be compatible with structurally complex isatins by introducing a linkable moiety at the 1-position, providing excellent yields and excellent dr’s and ee’s (Figure 5, **3s**–**3u**). This makes the sequential reaction attractive to link CHBOs with other potentially interesting moieties.

**Figure 5:**
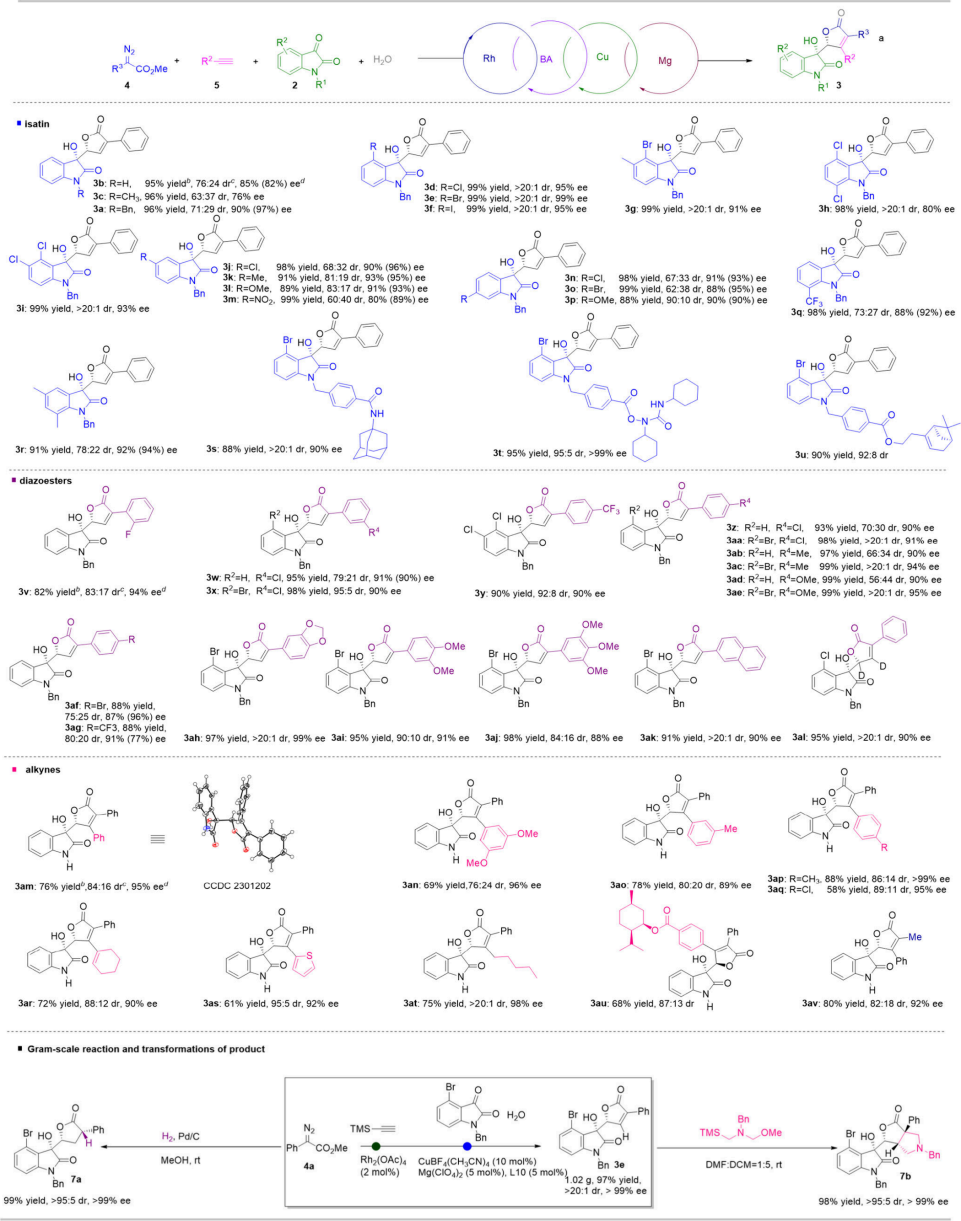
Substrate scope of catalytic asymmetric reaction of diaozesters, alkynes, water, and isatins. Standard reaction conditions: 0.25 mmol scale, **4**:**5**:**2** = 2.5:5 or >5:1, 1 mL trimethylacetylene, 2.5 mL ethyl burate, 10 mol % Cu­(CH_3_CN)_4_BF_4_, 5 mol % AcOH. ^b^Isolated yield. ^c^ee value was determined by chiral HPLC. ^d^dr value was determined by crude HNMR. ee values of minor diastereoisomers are in the parentheses.

Next, we investigated the compatibility of diazoesters. Various diazoesters with substituents at the *ortho*-, *meta*-, *para*-, and multiple positions of the aromatic ring were tested. *ortho*-F substituted diazoeseter gave 82% yield, 83:17 dr, and 94% ee by matching with isatin **2a**, while *meta*-Cl substituted diazoester provided 95% yield, 79:21 dr, and 91% ee (Figure 5, **3v** and **3w**). As we observed before, the diastereoselectivity increased substantially when 4-Br-isatin was used as a partner, and the trend could be observed in *para*-substituted diaozesters as well (Figure 5, **3w**–**3x** and **3y**–**3ae**). For *para*-substituted diazoesters, yields fluctuated between 88% and 99%, dr’s varied between 66:34 and >20:1, and ee’s changed between 87% and 95% (Figure 5, **3y**–**3ag**). Then multiple-substituted diazoesters gave 95–98% yields, 84:16 to >20:1 dr’s, and 88–99% ee’s when partnering with 4-Br-isatin. Naphthalenyldiazoester could afford 91% yield, >20:1 dr, and 90% ee. Lastly, a dideuterated product could be synthesized from a phenyldiazoester and 4-Cl-isatin. Overall, various groups at different positions of aromatic ring of aryldiazoesters are well tolerated, demonstrating the potential of the reaction in creating molecular diversity by changing diazoester substrates.

In the following, we test the compatibility of alkyne substrates. Terminal alkynes with aromatic/heteroaromatic, alkenyl, alkyl substituents are all tolerated in the reaction, giving 58–88% yields, 76:24 to >20:1 dr’s, 89–99% ee’s (Figure 5, **3am**–**3av**). This is a striking improvement in substrate scope compared to our previous work of racemic synthesis CHBO with cyclopropane carboxylic acid as the starting material. What’s more, the alkyldiazoester is compatible in the reaction by matching with phenyl acetylene, giving 80% yield, 80:20 dr, and 92% ee. This represents the first successful example of alkyldiazoester in the cyclopropene-initiated carbenoid chemistry.

### Synthetic Application

To further validate the value of the reaction, a gram-scale sequential reaction of diazoester **5a**, trimethylsilyl acetylene, isatin **2a**, and water produced **3e** with 97% yield, >20:1 dr, and >99% ee. The resulting **3e** could be easily reduced to **7a** by hydrogen under the catalysis of Pd/C. Additionally, [3 + 2] cycloaddition of **3e** and dipole is smooth at room temperature, providing a new polyhetercycle **7b** with near quantitative yield and excellent dr and ee (Figure 5, bottom).

To conclude, the asymmetric multicomponent sequential reaction of diazoesters, alkynes, isatins, and water is featured by broad substrate scope, excellent reactivity, and stereoselectivity, representing an ideal methodology of the optically pure CHBOs. And the CHBOs could be further modified by [3 + 2] cycloaddition or hydrogenation, rendering molecular diversity for building up screening library of CHBOs. However, achieving the reactivity of other cyclopropene derivatives, such as amides, thioesters, and acceptor–acceptor-type cyclopropenes, requires advanced catalytic strategies. Additionally, the reactivity of other carbonyl electrophiles, including aldehydes and unactivated ketones, in the MMCR still requires an in-depth investigation.

### Discovery of PTP1B Inhibitors from CHBO Library

To determine the value of CHBOs in drug discovery, we constructed a synthetically accessible library of CHBOs based on the substrate scope of the reaction we newly developed. A quick *in silico* screening and DiFMUP assay screening identified CHBO **3ak** as an interesting PTP1B inhibitor. To explore the relationship between inhibitor activity of **3ak** against PTP1B and the stereochemistry, two enantiomers (*S*, *S*)-**3ak** and (*R*, *R*)-**3ak** were synthesized and evalutated in the DiFUMP assay (Figure 6a). Interestingly, (*S*, *S*)-**3ak** is 3-fold more potent than (*R*, *R*)-**3ak**, implicating the stereochemistry of CHBO is pivotal to the inhibitory activity (Figure 6b).

**Figure 6:**
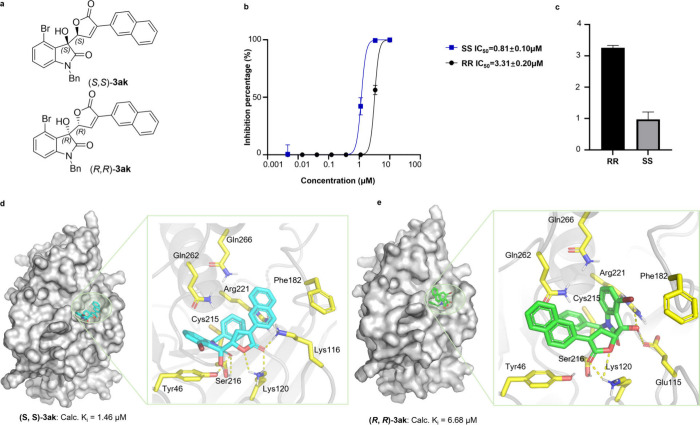
Importance of stereochemistry of **3ak** in inhibiting PTP1B. (a) The structures of enantiomers of *syn*-**3ak**. (b) The dose–response graph of the enantiomers of *syn*-**3ak** against PTP1B. (c) The comparison of IC_50_ values of the enantiomers of *syn*-**3ak**. (d) The docking model of (*S*, *S*)-**3ak** and PTP1B. Left, the surface pocket view of the model; right, the close look at the interactions between (*S*, *S*)-**3ak** and PTP1B. (e) The docking model of (*R*, *R*)-**3ak** and PTP1B. Left, the surface pocket view of the model; right, the close look at the interactions between (*R*, *R*)-**3ak** and PTP1B.

### Molecular Docking

Further molecular docking revealed that both of the enantiomers bind to the active site of PTP1B and the calculated *K*_i_ values are consistent with the trend of experimental IC_50_ values (Figures 6d and 6e). A close look at the binding pocket reveals that residues Gln266, Gln262, Arg221, Phe182, Cys215, Ser216, Lys116, Lys120, and Tyr46 are involved in the interactions between (*S*, *S*)-**3ak** and PTP1B (Figure 6d). In comparison, Glu115, instead of Lys116, is invovled in the model of (*R*, *R*)-**3ak** and PTP1B (Figure 6e). The extra H-bonding network constituted by Ser216, Tyr46, and the carbonyl group of oxindole and the extra π–π interactions between naphthalenyl ring and the phenyl ring of Phe182 in the model of (*S*, *S*)-**3ak** and PTP1B may interpret its superior inhibitory activity. The predicted model paves the way for further understanding the inhibition mechanism of (*S*, *S*)-**3ak** via other structural biology tools, and the advancement will be updated shortly.

### Structure and Activity Relationship of CHBOs

As shown in Table 2, oxindoles **6n**, **6u**, **6x**, **6aa**, **6ae**, **6ak**, **6ah**, and **6au** showed 0.29–1.58 μM IC_50_ values, representing the first tier among the compounds tested. Oxindoles **6b**, **6e**–**6i**, **6k**, **6m**, **6p**, **6s**, **6ab**–**6ad**, and **6am** are the second tier with IC_50_ values ranging from 2 to 10 μM. Oxindoles **6a**, **6d**, **6j**, **6l**, **6ae**, **6an**, **6ai**, and **6aj** gave 10–30 μM IC_50_ values, ranking at the third tier. And the other oxindoles have no significant inhibition at 50 μM. Some trends of the SAR could be summarized: (1) the long hydrophobic tails at the N-1 position of oxindole significantly contribute to the inhibitory activity against PTP1B (e.g., **6s** and **6u**); (2) the right side rigid aromatic rings on the γ-lactone, which are favorable for π–π stacking, are benefical to the bioactivity, consistent with the molecular docking model (e.g., **6ah** and **6ak**), where the aromatic ring tends to interact with the side chain of Tyr46 via π–π stacking; and (3) the long and hydrophobic tail of the left side aromatic ring has an obvious contribution the bioactivity (e.g., **6au**). This long tail is supposed to be close to the side chain Phe280, giving potential hydrophobic interactions. In summary, the preliminary investigation on the SAR gave some consistent results with the molecular docking. However, to fully understand the inhibition mechanism and build up a reliable SAR for prediction, structural biology tools like cryogenic electron microscopy or X-ray crystallography are needed, and more analogues should be designed and tested based on the structural biology data.

**Table 2:** Structure and Activity Relationship of CHBOs^a^

comp. ID	PTP1B lC_50_ (μM)	comp. ID	PTP1B lC_50_ (μM)	comp. ID	PTP1B lC_50_ (μM)	comp. ID	PTP1B lC_50_ (μM)
**6a**	29.67 ± 0.56	**6k**	9.08 ± 0.12	**6x**	1.01 ± 0.16	**6ai**	22.65 ± 0.75
**6b**	6.93 ± 0.77	**6l**	21.65 ± 1.48	**6aa**	1.14 ± 0.14	**6aj**	19.94 ± 0.37
**6d**	17.75 ± 1.20	**6m**	5.03 ± 0.83	**6ab**	6.68 ± 0.88	**6ak**	1.46 ± 0.28
**6e**	2.10 ± 0.31	**6n**	1.58 ± 0.28	**6ac**	9.13 ± 0.62	**6ah**	0.92 ± 0.18
**6f**	8.17 ± 1.06	**6p**	8.23 ± 0.91	**6ad**	7.82 ± 1.44	**6au**	1.56 ± 0.24
**6g**	8.13 ± 0.06	**6q**	1.00 ± 0.25	**6ae**	11.17 ± 2.87		
**6i**	3.02 ± 0.22	**6s**	2.11 ± 0.22	**6am**	4.03 ± 0.45		
**6j**	15.17 ± 1.02	**6u**	0.29 ± 0.02	**6an**	16.54 ± 1.42		

a The IC_50_ values were evaluated via a DiFMUP assay at room temperature. Positive control, ABBV-CLS-484; negative control, DMSO.

### Cellular Anticancer Immunity of the CHBO (*S*, *S*)-**3ak**

We further evaluated (*S*, *S*)-**3ak** with a cellular anticancer immunity assay. The assay measures the inhibition of cancer cell growth by compounds with or without cytokine IFNγ.^19,20,52^ The synergy of testing compounds and IFNγ in inhibiting cancer cells implicates the testing compound contributes anticancer immunity. The synergy index is defined by the formula index = (IC_50_^IFNγ–^ – IC_50_^IFNγ+^)/IC_50_^IFNγ+^. The higher the synergy index is, the more potent the anticancer immunity is. (*S*, *S*)-**3ak** showed anticancer immunity in the MB231 cell line with a synergy index of 3.49. In other words, we identified (*S*, *S*)-**3ak** as a promising PTP1B inhibitor, preparing for the coming hit-lead-candidate optimization.

## Conclusions

In conclusion, we developed the MMCRs of diazoesters, alkynes, isatins, and water. The MMCRs featured excellent chemo-, diastereo-, and enantioselectivities and mild conditions, allowing for rapid access to CHBOs with a broad substrate scope. The experimental and calculational study on the mechanism of the MMCRs revealed an intriguing cascade pathway composed of cyclopropenation, desilylation, cycloisomerization, aldol-type addition, and hydrolysis and characterized an undocumented catalytic model of Mg/Feng ligand complex, Cu catalyst, and HOAc. The MMCRs allow for quick generation of CHBOs, which are inaccessible otherwise, and for discovery of (*S*, *S*)-**3ak** as a potent PTP1B inhibitor. The DiFMUP assay and molecular docking underscore the significance of chirality in the potency of (*S*, *S*)-**3ak**, demonstrating the necessity in synthesizing enantiopure CHBOs via the MMCRs. This work represents a research mode which bridges synthetic methodology and drug discovery. Looking ahead, future efforts will focus on medicinal chemistry to optimize CHBOs as PTP1B inhibitors and explore their *in vivo* efficacy for immuno-oncology.

## Supplementary Material





## Notes

Materials; methods; condition optimization; general experimental procedure for compounds **3a**–**3av**; gram scale amplification reaction and product derivation; isotope tracer experiment; nonlinear effect experiment; single crystal X-ray diffraction data; ^1^H NMR and ^13^C NMR; HPLC; computational study; and biological study (PDF)
Transparent Peer Review report available (PDF)
