# Supplementary material for: Multicatalysis-Enabled Multicomponent Reactions Generate a PTP1B Inhibitor
Source: ACS Cent Sci. 2025 May 19;11(6):938–49. doi: 10.1021/acscentsci.5c00041 (PMC12203432; doi:10.1021/acscentsci.5c00041)
Supplement: Supplementary file 1 [file oc5c00041_si_001.pdf]

Supplementray Information

Multicatalysis-Enabled Multicomponent Reactions Generate A PTP1B Inhibitor

Taoda Shi,[1, 2, 3, §, *] Yukai Li, [1, 2, 3, §] Jiying Yang, [1, 2, 3] Weining Weng, [1, 2, 3] Mengchu Zhang, [1, 2, 3] Jirong Shu, [1, 2, 3] Yu Qian, [1, 2, 3, *] Tianyuan Zhang, [1, 2, 3] and Wenhao Hu[1, 2, 3, *]

Present Addresses

1. Guangdong Key Laboratory of Chiral Molecule and Drug Discovery, School of Pharmaceutical Sciences, Sun Yat-sen University, Guangzhou 510006, China.
2. State Key Laboratory of Anti-Infective Drug Discovery and Development, Guangdong Provincial Key Laboratory of Chiral Molecule and Drug Discovery, School of Pharmaceutical Sciences, Sun Yat-sen University, Guangzhou 510006, China.
3. Guangdong Basic Research Center of Excellence for Functional Molecular Engineering, Sun Yat-sen University, Guangzhou 510006, China.

[§] These authors contributed equally

Corresponding Authors

* Corresponding author Email address: shitd@mail.sysu.edu.cn; qianyu5@mail.sysu.edu.cn; huwh9@mail.sysu.edu.cn.

Content

1. General information S2

2. Condition optimization S2

3. general experimental procedure for compound **3a**-**3av** S7

4. Gram scale amplification reaction and product derivation S37

5. Isotope tracer experiment S40

6. Nonlinear effect experiment S44

7. Single Crystal X-ray Diffraction Data S44

8. ^{1}H NMR and ^{13}C NMR spectra S53

9. HPLC spectra. S114

10. Computational study S164

11. *In silico* screening and molecular dockin S190

12. Biological study S194

13. References S196

1. General information

All reagents and solvents are commercially available and applied without further purification. The cyclopropene esters and isatins were prepared according to the known protocol[1]. ^{1}H NMR and ^{13}C NMR raw data were collected by a Bruker AMX500 (400MHz and 500MHz) spectrometer and spectrums were exported through MestReNova software. Deuterated solvent included DMSO-d_6 and $CDCl_3$, tetramethylsilane used as internal standard. The chemical shifts (δ) are reported in parts per million (ppm) and the coupling constant (J) unit is Hz. High resolution mass spectrometry (HRMS) was obtained through an Agilent 6538 UHD Accurate-Mass ultra-performance liquid chromatography quadrupole time-of-flight mass (UPLC-QTOF/MS) spectrometer. HPLC analyses were performed on a Waters-Aliance e2695, using n-hexane and isopropyl alcohol as mobile phase. Flash chromatography carried out on silica gel 200-300 mesh with newly configured eluent.

2. Condition optimization

Table S1. Condition screening of the asymmetric reaction of cyclopropene (4a), isatin (2a) and water. [a]

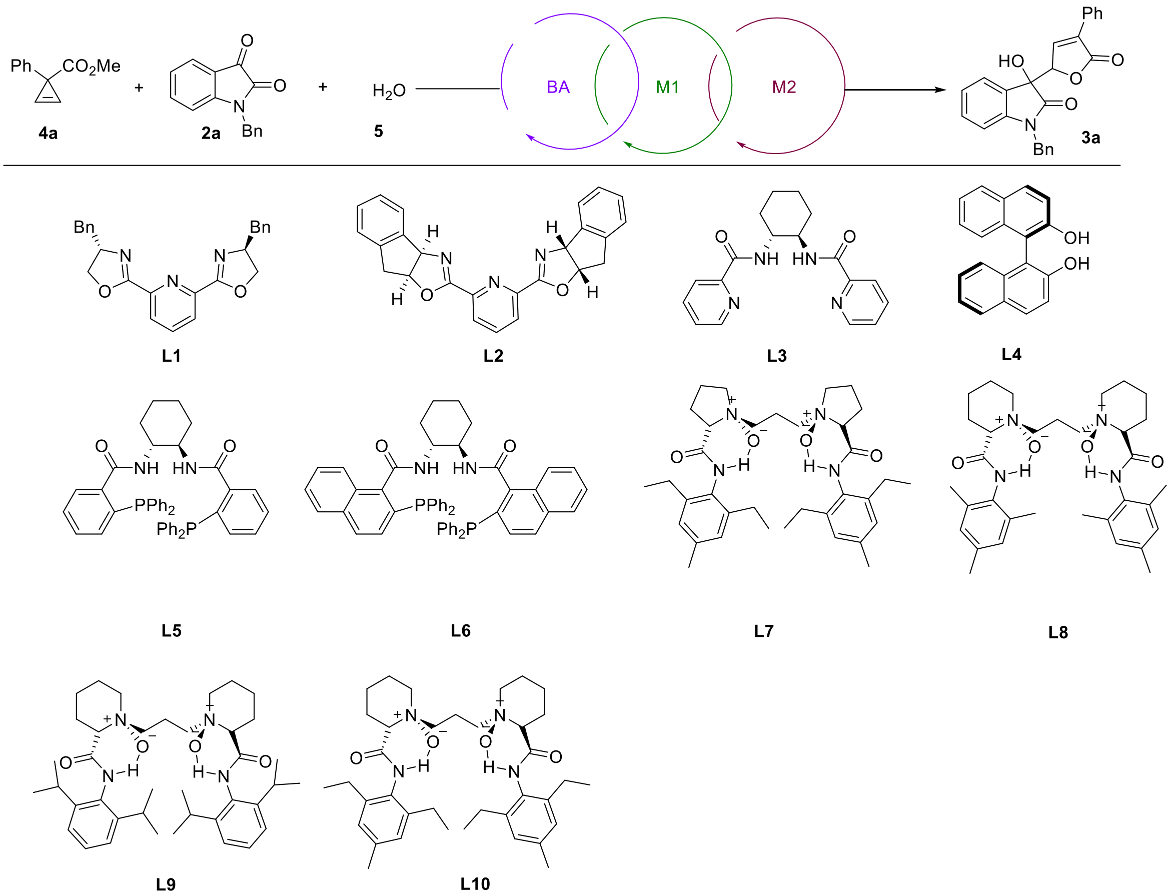

entry	[M1]	[M2]	Ligand	organocatalyst	Solvent	Time /h	yield/ %[f]	ee/%[h]	*dr*[i] (syn:anti)
**1**	/	$Cu(OTf)_2$	**L1**	/	EtOAc	48h	<5	/	/
**2**	/	$Cu(OTf)_2$	**L2**	/	EtOAc	48h	<5	/	/
**3**	/	$Cu(OTf)_2$	**L3**	/	EtOAc	48h	<5	/	/
**4**	/	$Cu(OTf)_2$	**L4**	/	EtOAc	48h	77	0/6	76:24
**5**	/	$Cu(OTf)_2$	**L5**	/	EtOAc	48h	80	0/0	80:20
**6**	/	$Cu(OTf)_2$	**L6**	/	EtOAc	48h	44	0/0	80:20
**7**	/	$Cu(OTf)_2$	**L7**	/	EtOAc	48h	62	18/22	79:21
**8**	$Cu(OTf)_2$	$Mg(OTf)_2$	**L7**	/	EtOAc	12h	68	44/32	87:13
**9**	$Cu(OTf)_2$	$Mg(OTf)_2$	**L8**	/	EtOAc	12h	93	36/48	80:20
**10**	$Cu(OTf)_2$	$Mg(OTf)_2$	**L9**	/	EtOAc	12h	58	71/44	90:10
**11**	$Cu(OTf)_2$	$Mg(OTf)_2$	**L10**	/	EtOAc	12h	77	70/10	82:18

**12**	$Cu(OTf)_2$	$Mg(ClO_4)_2$	**L10**	/	EtOAc	12h	92	68/62	83:17
**13**	$Cu(OTf)_2$	$Sc(OTf)_2$	**L10**	/	EtOAc	8h	92	40/46	80:20
**14**	$Cu(MeCN)_4BF_4$	$Mg(ClO_4)_2$	**L10**	/	EtOAc	8h	95	68/66	80:20
**15**	$Cu(MeCN)_4PF_6$	$Mg(ClO_4)_2$	**L10**	/	EtOAc	12h	90	50/48	83:17
**16**	CuTc	$Mg(ClO_4)_2$	**L10**	/	EtOAc	12h	59	0/12	64:36
**17**	$Rh_2(esp)_2$	$Mg(ClO_4)_2$	**L10**	/	EtOAc	12h	<5	/	/
**18**[b]	$Cu(MeCN)_4BF_4$	$Mg(ClO_4)_2$	**L10**	/	EtOAc	8h	95	70/66	79:21
**19**[c]	$Cu(MeCN)_4BF_4$	$Mg(ClO_4)_2$	**L10**	/	EtOAc	4h	96	71/65	78:22
**20**[d]	$Cu(MeCN)_4BF_4$	$Mg(ClO_4)_2$	**L10**	/	EtOAc	3h	77	68/68	77:23
**21**[e]	$Cu(MeCN)_4BF_4$	$Mg(ClO_4)_2$	**L10**	/	EtOAc	4h	96	71/66	78:22
**22**[c]	$Cu(MeCN)_4BF_4$	$Mg(ClO_4)_2$	**L10**	/	Ethyl propanoate	4h	<20	/	/
**23**[c]	$Cu(MeCN)_4BF_4$	$Mg(ClO_4)_2$	**L10**	/	Isopropyl acetate	4h	98	67/33	78:22
**24**[c]	$Cu(MeCN)_4BF_4$	$Mg(ClO_4)_2$	**L10**	/	Dry EA+4Å molecular sieves	4h	<5	/	/
**25**[c]	$Cu(MeCN)_4BF_4$	$Mg(ClO_4)_2$	**L10**	/	Isopropyl acetate+EtOAc	4h	93	68/70	77:23
**26**[c]	Cu(MeCN)	$Mg(ClO_4)_2$	**L10**	/	*tert*-Butyl	4h	85	71/65	75:25

	$_4$BF$_4$				acetate				
**27**[c]	Cu(MeCN)$_4$BF$_4$	Mg(ClO$_4$)$_2$	**L10**	/	THF	4h	97	44/42	81:19
**28**[c]	Cu(MeCN)$_4$BF$_4$	Mg(ClO$_4$)$_2$	**L10**	/	MTBE	4h	50	32/40	79:21
**29**[c]	Cu(MeCN)$_4$BF$_4$	Mg(ClO$_4$)$_2$	**L10**	/	xylene	4h	43	26/26	76:24
**30**[c]	Cu(MeCN)$_4$BF$_4$	Mg(ClO$_4$)$_2$	**L10**	/	toluene	4h	48	41/40	77:23
**31**[c]	Cu(MeCN)$_4$BF$_4$	Mg(ClO$_4$)$_2$	**L10**	/	DCE	4h	76	44/36	82:18
**32**[c]	Cu(MeCN)$_4$BF$_4$	Mg(ClO$_4$)$_2$	**L10**	/	Ethyl butyrate	4h	92	73/70	78:22
**33**[c]	Cu(MeCN)$_4$BF$_4$	Mg(ClO$_4$)$_2$	**L10**	EtOH	Ethyl butyrate	4h	97	73/70	72:28
**34**[c]	Cu(MeCN)$_4$BF$_4$	Mg(ClO$_4$)$_2$	**L10**	AcOH	Ethyl butyrate	4h	90	90/90	78:22
**35**[c]	Cu(MeCN)$_4$BF$_4$	Mg(ClO$_4$)$_2$	**L10**	TFA	Ethyl butyrate	4h	65	90/89	70:30
**36**[c]	Cu(MeCN)$_4$BF$_4$	Mg(ClO$_4$)$_2$	**L10**	HCOOH	Ethyl butyrate	4h	78	90/90	71:29
**37**[c]	Cu(MeCN)$_4$BF$_4$	Mg(ClO$_4$)$_2$	**L10**	TsOH	Ethyl butyrate	4h	90	78/79	73:27
**38**[c]	**Cu(MeCN)$_4$BF$_4$**	**Mg(ClO$_4$)$_2$**	**L10**	**AcOH**[f]	**Ethyl butyrate**	**4h**	**83**	**90/91**	**66:34**

[a]Unless otherwise indicated, the reactions were all on the scale of 0.05 mmol with 1 mL of solvent, **4a**: **2a**=1.5:1，[M1]=10 mol%，[M2]=5 mol%，Ligand=5 mol%，Additive=5 mol%；[b] [M1]=5 mol %；[c] [M1]=5 mol %, [M2]=10 mol %；[d] [M1]=5 mol %,[M2]=15 mol %;[e] Ligand=10 mol%, M2=10 mol %；[f]Additive=10 mol%；[g] Determined by ^{1}H NMR analysis of the crude products based on internal standard (1,3,5-trimethoxybenzene)；[h] Determined by HPLC analysis using a chiral stationary phase；[i] Determined by ^{1}H NMR analysis of the crude products.

Table S2. Condition screening of the asymmetric reaction of cyclopropene (4o), isatin (2a) and water.[a]

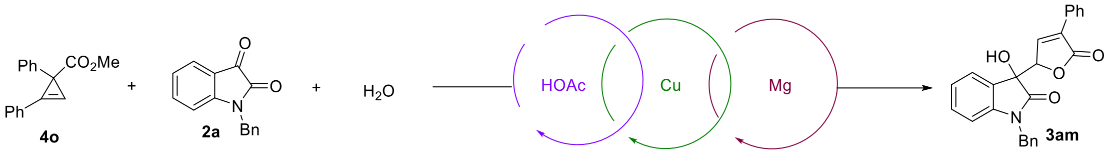

Entry	Cu(MeCN4)BF4 (mol%)	L10 (mol%)	AcOH (mol%)	Mg(ClO4)2 (mol%)	Time (h)	yield (%)[b]	*dr*[c]	ee[d] (%)
1	15	5	5	5	12	68	85：15	77
2	15	5	10	5	12	67	85：15	85
3	15	10	10	10	12	70	85：15	95
**4**	**20**	**10**	**10**	**10**	**4**	**76**	**84：16**	**95**
5	25	10	10	10	2	61	85：15	94

[a] Unless otherwise indicated, the reactions were all on the scale of 0.1 mmol with 1.5 mL of solvent, **4o** : **2a**=1.5:1; [b] Determined by ^{1}H NMR analysis of the crude products based on internal standard (1,3,5-trimethoxybenzene); [c] Determined by ^{1}H NMR analysis of the crude products; [d] Determined by HPLC analysis using a chiral stationary phase.

Firstly, a panel of ligands including bisoxazolines[1], Binol[2], Trost-type bisamides[3], and Feng ligands[4] were evaluated. The results showed ligands with H-bonding donor gave higher reactivities than the ones without H-bonding donor (Supporting information, table 1, **L3**, **L5**-**L7** *vs* **L1**-**L2**). L4 is lack of reactivity probably since the strong coordination of bispyridine makes the ligand/substrate exchange slow and inhibits the catalytic cycle. Feng-ligand **L7** gave 18% ee, while the other ligands resulted in 0-6% ee. Interestingly, the rate and ee of the reaction was obviously improved if $Mg(OTf)_2$ was used as a co-catalyst. Then three other Feng-ligands **L8**-**L10** were evaluated in the cocatalyst system, giving **L10** as the optimal ligand with 77% yield, 82:18 dr and 70% ee for the major diastereoisomer.

Next, **L10** was chosen for the screening of combinations of metal catalysts. As shown in the Table 2, $Mg(ClO_4)_2$ gave higher reactivity than $Mg(OTf)_2$ when matching with $Cu(OTf)_2$. Therefore, $Mg(ClO_4)_2$ was selected to test a group of transition metal catalysts including $CuPF_6(CH_3CN)_4$, $CuBF_4(CH_3CN)_4$, CuTc, and $Rh_2(esp)_2$. It turned out that $CuBF_4(CH_3CN)_4$ is

the best partner, giving higher yield, faster velocity and comparable stereoselectivity. Then the ratio of the two co-catalysts were adjusted and 5 mol% $Mg(ClO_4)_2$ and 10 mol% $CuBF_4(CH_3CN)_4$ was identified as the best match, with 96% yield, 78:22 dr and 71% ee for major diastereoisomers in 4 hours .With the co-catalyst match being set, a group of solvent including ethers, arenes, halids and esters were then screened. It showed. that esters gave better enantioselectivity than the other solvents, with ethyl butyrate giving the best results (92% yield, 72:28 dr, 73% ee (major), 70% ee (minor), Supporting information, Table 1).

To further improve the stereoselectivity, a series of additives were tested. Interestingly, the reaction was totally inhibited if 4 Å molecular sieves was added, indicating that water may be essential to the reaction. The other H-bonding donors including alcohol, acetic acid (HOAc), formic acid and trifluoracetic acid could boost the reaction and HOAc gave the optimal results, with 90% yield, 72: 28 dr, 90% ee (major), 90% ee (minor) (Table 1). Overall, the recipe of the reaction was established as: "5 mol% $Mg(ClO_4)_2$,10 mol% $CuBF_4(CH_3CN)_4$, 5 mol% **L10**, 10 mol% HOAc, in ethyl butyrate, 4 hours".

Condition screening of the asymmetric reaction of cyclopropene (4o), isatin (2a) and water: The reaction rate and enantioselectivity increased with the increase in the loading of $CuPF_6(CH_3CN)_4$, $Mg(ClO_4)_2$, ligand **L10** and HOAc (Supporting information, Table 2). Eventually, 20 mol% $CuPF_6(CH_3CN)_4$, 10 mol% $Mg(ClO_4)_2$, 10 mol% ligand **L10** and 10 mol% HOAc was adopt as the optimal conditions.

3. general experimental procedure for compound 3a-3av

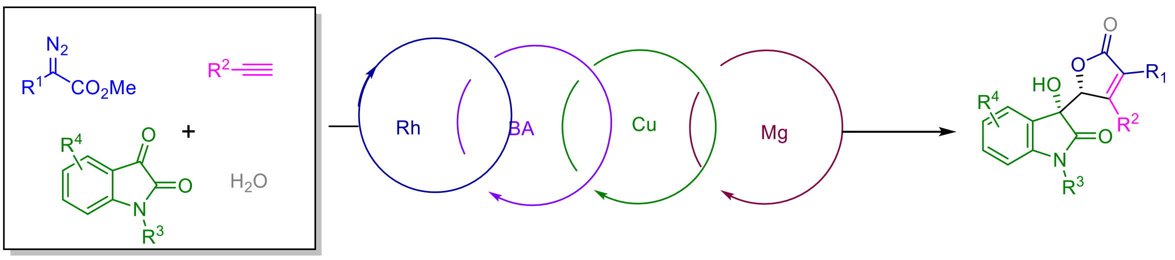

Scheme S1. Four-component cascade reaction of diazoacetates, alkynes, istains and water enabled by relay catalysis.

(1) **3a-3al:** Isatin (**2**, 0.25 mmol, 1eq), $Mg(ClO_4)_2$ (5 mol%), **L10** (5 mol%) were dissolved in 2.5 mL ethyl butyrate. At the same time, the diazo compound (**1**, 0.625 mmol, 2.5 eq) was dissolved in trimethylsilyne (**6a**, 0.4mL) and injected into the suspension of $Rh_2(OAc)_4$ (2 mol%) in trimethylsilyne (**6a**, 0.6 mL) by injection pump at room temperature within 20 minutes and continued to react for 10 minutes. The reaction mixture was concentrated and then redissolved in 2.5 mL ethyl butyrate. The resulting solution was immediately added to the preactivated isatins by $Mg(ClO_4)_2$-**L10**, then $Cu(MeCN)_4BF_4$ (10 mol%) and AcOH (5 mol%). was added (Scheme 1). The reaction was monitored by TLC. Until the disappear of isatins, the reaction mixture was concentrated to obtain the crude product, the dr values of the crude product were determined by ^{1}H NMR, the ee values were determined by chiral high performance liquid chromatography, and then the pure products **3a-3al** were purified by column chromatography (petroleum ether: ethyl acetate = 2:1).

(2) **3am-3av:** Isatin (**2**, 0.25 mmol, 1eq), $Mg(ClO_4)_2$ (10 mol%), **L10** (10 mol%) were dissolved in 2.5 mL ethyl butyrate. At the same time, the diazo compound (**1**, 0.625 mmol, 2.5 eq), terminal alkyne (**6**, 1.375 mmol, 5.5 eq) was dissolved in anhydrous dichloromethane (0.5 mL) and injected into the suspension of $Rh_2(OAc)_4$ (2 mol%) in anhydrous dichloromethane (0.5 mL) at room temperature within 20 minutes by injection pump and continued to react for 10 minutes. The reaction mixture was concentrated and then redissolved in 2.5 mL ethyl butyrate. The resulting solution was immediately added to the preactivated isatins by $Mg(ClO_4)_2$-**L10**, then $Cu(MeCN)_4BF_4$ (10 mol%) and AcOH (5 mol%). was added. The reaction was monitored by TLC. Until the disappear of isatins, the reaction mixture was concentrated to obtain the crude product. The dr values of the crude products were determined by ^{1}H NMR, the ee values were determined by chiral high performance liquid chromatography, and then the pure products **3am-3av** were purified by column chromatography (petroleum ether: ethyl acetate = 1:1). The preparation of **3av** is the same except that $Rh_2(OAc)_4$ (2 mol%) is replaced by $Rh_2(TPA)_4$ (2 mol%).

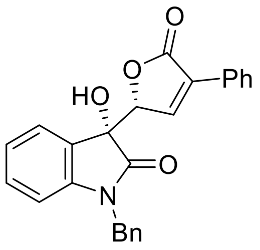

(**3a**)

(*R*)-1-benzyl-3-hydroxy-3-((R)-5-oxo-4-phenyl-2,5-dihydrofuran-2-yl)indolin-2-one (3a)：White solid, mp:160.0-161.2℃; 96% yield, 71:29 *dr*, 90% (97%) *ee*, $[\alpha]_D^{20}$ =30.3 (c=0.033,CH_2Cl_2); ^1H NMR (500 MHz, $CDCl_3$) δ 7.83 (dd, *J* = 6.7, 2.9 Hz, 2H), 7.73 (d, *J* = 1.8 Hz, 1H), 7.46 – 7.39 (m, 3H), 7.32 (d, *J* = 7.3 Hz, 1H), 7.27 – 7.21 (m, 6H), 7.01 (t, *J* = 7.6 Hz, 1H), 6.74 (d, *J* = 7.9 Hz, 1H), 5.37 (d, *J* = 1.9 Hz, 1H), 5.05 (d, *J* = 15.7 Hz, 1H), 4.75 (d, *J* = 15.7 Hz, 1H), 3.47 (s, 1H);^{13}C NMR (101 MHz, DMSO) δ 175.49, 170.69, 147.30, 143.54, 136.33, 132.28, 130.63, 129.84, 129.73, 129.16, 129.03, 127.86, 127.58, 127.35, 126.41, 125.89, 122.53, 110.01, 82.06, 76.77, 43.15;HRMS(TOF MS ESI$^+$) caculated for $C_{25}H_{19}NO_4Na$ $[M+Na]^+$:420.1206, found 420.1206; HPLC conditions for determination enantiomeric excess: Chiral ADH, λ=254 nm, hexane:2-propanol=80:20, flow rate=1.0 mL/min, t_{minor}=18.270, t_{major}=21.741.

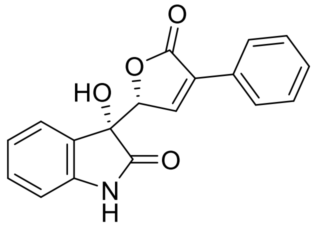

(**3b**)

(*R*)-3-hydroxy-3-((R)-5-oxo-4-phenyl-2,5-dihydrofuran-2-yl)indolin-2-one (3b)：White solid, mp: 171.1-171.9℃; 95% yield, 76:24 *dr*, 85% (82%) *ee*, $[\alpha]_D^{20}$ =63.6 (c=0.033,CH_2Cl_2); ^1H NMR (400 MHz, DMSO) δ 10.53 (s, 1H), 8.32 (d, *J* = 1.8 Hz, 1H), 7.93 – 7.87 (m, 2H), 7.45 (dt, *J* = 14.6, 4.8 Hz, 3H), 7.22 (t, *J* = 7.7 Hz, 1H), 7.05 (d, *J* = 7.4 Hz, 1H), 6.85 (t, *J* = 7.0 Hz, 2H), 6.81 (s, 1H), 5.38 (d, *J* = 1.7 Hz, 1H); ^{13}C NMR (126 MHz, DMSO) δ 176.91, 170.81, 147.50, 143.11, 132.08, 130.56, 129.76, 129.14, 127.30, 126.91, 126.02, 121.69, 110.43, 81.98, 76.84; HRMS(TOF MS ESI$^+$) caculated for $C_{18}H_{14}NO_4Na$ $[M+Na]^+$:330.0737, found 330.0738; HPLC

conditions for determination enantiomeric excess: Chiral IC, λ=254 nm, hexane:2-propanol=80:20, flow rate=1.0 mL/min, t_{minor}=13.764, t_{major}=28.158.

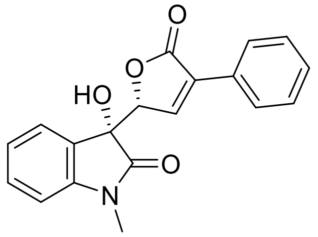

(**3c**)

(*R*)-3-hydroxy-1-methyl-3-((R)-5-oxo-4-phenyl-2,5-dihydrofuran-2-yl)indolin-2-one (3c): White solid, mp: 177.8-179.2℃; 96% yield, 63:37 *dr*, 76% *ee*, $[\alpha]_D^{20}$ =87.8 (c=0.033,CH_2Cl_2); ^1H NMR (400 MHz, DMSO) δ 8.33 (s, 1H), 7.89 (d, *J* = 6.7 Hz, 2H), 7.45 (d, *J* = 7.0 Hz, 3H), 7.33 (t, *J* = 7.4 Hz, 1H), 7.11 (d, *J* = 7.2 Hz, 1H), 7.05 (d, *J* = 7.5 Hz, 1H), 6.94 (t, *J* = 7.2 Hz, 1H), 6.88 (s, 1H), 5.42 (s, 1H), 3.17 (s, 3H); ^{13}C NMR (126 MHz, DMSO) δ 175.21, 170.74, 147.37, 144.53, 132.14, 130.69, 129.79, 129.73, 129.14, 127.30, 126.38, 125.61, 122.41, 109.35, 81.84, 76.55, 26.59; HRMS(TOF MS ESI$^+$) caculated for $C_{19}H_{15}NO_4Na$ $[M+Na]^+$:344.0893, found 344.0898; HPLC conditions for determination enantiomeric excess: Chiral IC, λ=254 nm, hexane:2-propanol=70:30, flow rate=1.0 mL/min, t_{minor}=12.861, t_{major}=20.748.

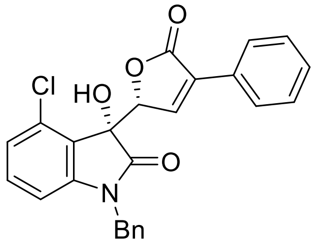

(**3d**)

(*R*)-1-benzyl-4-chloro-3-hydroxy-3-((R)-5-oxo-4-phenyl-2,5-dihydrofuran-2-yl)indolin-2-one (3d): White solid, mp: 122.1-123.8℃; 99% yield, >20:1 *dr*, 95% *ee*, $[\alpha]_D^{20}$ =120.7 (c=0.033,CH_2Cl_2); ^1H NMR (500 MHz, DMSO) δ 8.22 (d, *J* = 1.8 Hz, 1H), 7.89 (dd, *J* = 7.8, 1.4 Hz, 2H), 7.47 – 7.40 (m, 3H), 7.33 (dd, *J* = 6.4, 2.6 Hz, 2H), 7.28 – 7.17 (m, 5H), 6.96 (d, *J* = 8.2 Hz, 1H), 6.83 (d, *J* = 7.9 Hz, 1H), 5.65 (d, *J* = 1.8 Hz, 1H), 5.03 (d, *J* = 16.0 Hz, 1H), 4.82 (d, *J* = 16.0 Hz, 1H); ^{13}C NMR (101 MHz, DMSO) δ 175.02, 170.69, 147.75, 145.47, 135.93,

132.33, 131.31, 130.14, 129.77, 129.74, 129.09, 128.98, 127.88, 127.52, 127.25, 124.41, 122.99, 109.03, 82.43, 78.24, 43.35; HRMS(TOF MS ESI+) caculated for $C_{25}H_{18}ClNO_4Na$ $[M+Na]^+$:454.0817, found 454.0817; HPLC conditions for determination enantiomeric excess: Chiral ODH, λ=254 nm, hexane:2-propanol=70:30, flow rate=1.0 mL/min, t_{minor}=24.492, t_{major}=9.895.

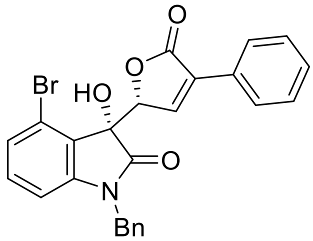

(**3e**)

(*R*)-1-benzyl-4-bromo-3-hydroxy-3-((R)-5-oxo-4-phenyl-2,5-dihydrofuran-2-yl)indolin-2-one (3e)：White solid, mp: 152.0-153.2℃; 99% yield, >20:1 *dr*, 99% *ee*, $[\alpha]_D^{20}$ =118.2 (c=0.033,CH_2Cl_2); ^1H NMR (400 MHz, DMSO) δ 8.14 (d, *J* = 1.7 Hz, 1H), 7.94 – 7.86 (m, 2H), 7.43 (q, *J* = 3.5 Hz, 3H), 7.33 – 7.27 (m, 2H), 7.24 – 7.11 (m, 6H), 6.86 (d, *J* = 7.6 Hz, 1H), 5.67 (d, *J* = 1.7 Hz, 1H), 5.01 (d, *J* = 16.0 Hz, 1H), 4.79 (d, *J* = 16.0 Hz, 1H); ^{13}C NMR (126 MHz, DMSO) δ 174.91, 170.70, 147.22, 145.49, 135.91, 132.45, 130.31, 129.75, 129.73, 129.06, 128.95, 127.85, 127.51, 127.31, 124.89, 119.61, 109.48, 82.31, 78.62, 43.28; HRMS(TOF MS ESI+) caculated for $C_{25}H_{18}BrNO_4Na$ $[M+Na]^+$:498.0311, found 498.0310; HPLC conditions for determination enantiomeric excess: Chiral ADH, λ=254 nm, hexane:2-propanol=80:20, flow rate=1.0 mL/min, t_{major}=25.910.

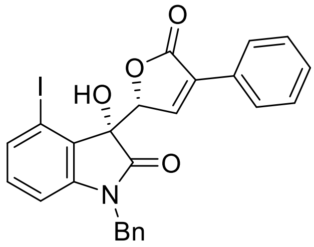

(**3f**)

(*R*)-1-benzyl-3-hydroxy-4-iodo-3-((R)-5-oxo-4-phenyl-2,5-dihydrofuran-2-yl)indolin-2-one (3f): White solid, mp: 168.8-169.8℃; 99% yield, >20:1 *dr*, 95% *ee*, $[\alpha]_D^{20}$ =133.8 (c=0.033,CH_2Cl_2); ^1H NMR (400 MHz, DMSO) δ 8.02 (d, *J* = 1.5 Hz, 1H), 7.94 – 7.83 (m, 2H), 7.48 – 7.37 (m, 4H),

7.26 (d, J = 7.1 Hz, 2H), 7.16 (dt, J = 23.0, 7.1 Hz, 3H), 7.06 – 6.94 (m, 2H), 6.87 (d, J = 7.8 Hz, 1H), 5.69 (d, J = 1.3 Hz, 1H), 4.98 (d, J = 16.0 Hz, 1H), 4.74 (d, J = 16.0 Hz, 1H); ^{13}C NMR (126 MHz, DMSO) δ 174.88, 170.75, 146.46, 144.90, 135.95, 134.05, 132.33, 130.73, 129.76, 129.73, 129.04, 128.91, 128.26, 127.81, 127.51, 127.42, 109.98, 92.46, 82.16, 78.74, 43.08; HRMS(TOF MS ESI$^+$) caculated for $C_{25}H_{18}INO_4Na$ [M+Na]$^+$:546.0173, found 546.0177; HPLC conditions for determination enantiomeric excess: Chiral ADH, λ=254 nm, hexane:2-propanol=80:20, flow rate=1.0 mL/min, t_{minor}=22.969, t_{major}=26.867.

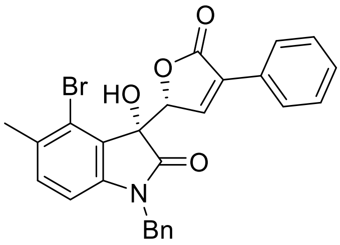

(**3g**)

(*R*)-1-benzyl-4-bromo-3-hydroxy-5-methyl-3-((R)-5-oxo-4-phenyl-2,5-dihydrofuran-2-yl)indolin-2-one (3g): White solid, mp: 188.2-189.9℃; 99% yield, >20:1 *dr*, 91% *ee*, $[\alpha]_D^{20}$ =69.6 (c=0.033,CH_2Cl_2); ^{1}H NMR (500 MHz, DMSO) δ 8.02 (d, J = 1.9 Hz, 1H), 7.91 – 7.84 (m, 2H), 7.47 – 7.41 (m, 3H), 7.25 (dd, J = 7.7, 2.5 Hz, 3H), 7.18 (t, J = 7.3 Hz, 1H), 7.16 – 7.05 (m, 3H), 6.78 (d, J = 8.0 Hz, 1H), 5.77 (d, J = 1.9 Hz, 1H), 4.99 (d, J = 16.0 Hz, 1H), 4.73 (d, J = 16.0 Hz, 1H), 2.21 (s, 3H); ^{13}C NMR (126 MHz, DMSO) δ 174.48, 170.88, 146.93, 142.97, 135.99, 132.60, 132.43, 130.66, 129.80, 129.73, 129.06, 128.89, 127.77, 127.46, 127.33, 125.47, 122.26, 109.26, 82.11, 79.00, 43.17, 22.38; HRMS(TOF MS ESI$^+$) caculated for $C_{26}H_{20}BrNO_4Na$ [M+Na]$^+$:512.0468, found 512.0467; HPLC conditions for determination enantiomeric excess: Chiral ADH, λ=254 nm, hexane:2-propanol=80:20, flow rate=1.0 mL/min, t_{minor}=19.282, t_{major}=28.501.

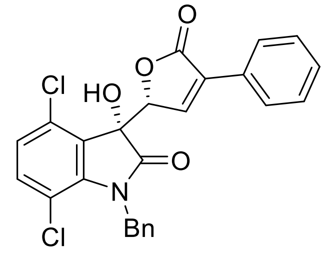

(**3h**)

(*R*)-1-benzyl-4,7-dichloro-3-hydroxy-3-((R)-5-oxo-4-phenyl-2,5-dihydrofuran-2-yl)indolin-2-one (3h)：White solid, mp: 177.8-179.2℃; 98% yield, >20:1 *dr*, 80% *ee*, $[\alpha]_D^{20}$ =29.8 (c=0.033,CH_2Cl_2); ^{1}H NMR (500 MHz, DMSO) δ 8.27 (d, *J* = 1.7 Hz, 1H), 7.95 – 7.88 (m, 2H), 7.48 – 7.41 (m, 3H), 7.36 – 7.31 (m, 2H), 7.26 – 7.17 (m, 5H), 7.05 (d, *J* = 8.8 Hz, 1H), 5.71 (d, *J* = 1.7 Hz, 1H), 5.28 (d, *J* = 16.9 Hz, 1H), 5.21 (d, *J* = 16.9 Hz, 1H);^{13}C NMR (126 MHz, DMSO) δ 175.67, 170.67, 147.26, 141.11, 137.52, 134.39, 130.52, 130.42, 129.84, 129.64, 129.11, 128.86, 127.41, 127.29, 126.26, 125.96, 125.90, 113.97, 82.25, 77.73, 44.90.; HRMS(TOF MS ESI$^+$) caculated for $C_{25}H_{17}Cl_2NO_4Na$ $[M+Na]^+$:488.0427, found 488.0429; HPLC conditions for determination enantiomeric excess: Chiral ADH, λ=254 nm, hexane:2-propanol=80:20, flow rate=1.0 mL/min, t_{minor}=20.330, t_{major}=27.890.

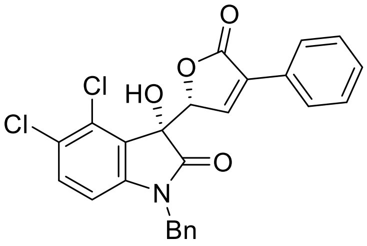

(**3i**)

(*R*)-1-benzyl-4,5-dichloro-3-hydroxy-3-((R)-5-oxo-4-phenyl-2,5-dihydrofuran-2-yl)indolin-2-one (3i)：White solid, mp: 155.8-156.6℃; 99% yield, >20:1 *dr*, 93% *ee*, $[\alpha]_D^{20}$ =154.6 (c=0.033,CH_2Cl_2); ^{1}H NMR (500 MHz, DMSO) δ 8.18 (t, *J* = 11.3 Hz, 1H), 7.90 (ddd, *J* = 19.2, 7.8, 1.6 Hz, 2H), 7.57 (d, *J* = 8.4 Hz, 1H), 7.47 – 7.42 (m, 3H), 7.31 (s, 1H), 7.28 (d, *J* = 6.7 Hz, 2H), 7.25 – 7.15 (m, 3H), 6.88 (d, *J* = 8.5 Hz, 1H), 5.71 (t, *J* = 4.2 Hz, 1H), 5.03 (d, *J* = 16.1 Hz, 1H), 4.81 (d, *J* = 16.1 Hz, 1H); ^{13}C NMR (126 MHz, DMSO) δ 174.58, 170.70, 147.30, 143.87, 135.64, 132.54, 130.54, 129.82, 129.66, 129.11, 128.99, 127.92, 127.46, 127.28, 126.26, 125.32, 110.19, 82.17, 78.71, 43.31; HRMS(TOF MS ESI$^+$) caculated for $C_{25}H_{17}Cl_2NO_4Na$

[M+Na]$^+$:488.0427, found 488.0427; HPLC conditions for determination enantiomeric excess: Chiral IC, λ=254 nm, hexane:2-propanol=80:20, flow rate=1.0 mL/min, t_{minor}=28.390, t_{major}=23.530.

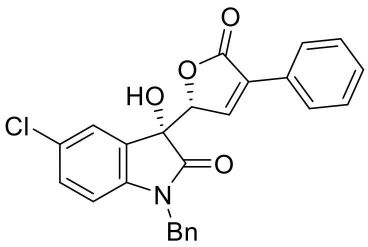

(**3j**)

(*R*)-1-benzyl-5-chloro-3-hydroxy-3-((R)-5-oxo-4-phenyl-2,5-dihydrofuran-2-yl)indolin-2-one (3j): White solid, mp: 113.4-115.0℃; 98% yield, 68:32 *dr*, 90% (96%) *ee*, $[\alpha]_D^{20}$ =101.4 (c=0.033,CH_2Cl_2); ^{1}H NMR (500 MHz, $CDCl_3$) δ 7.82 (dd, *J* = 6.6, 2.9 Hz, 1H), 7.70 (d, *J* = 1.7 Hz, 1H), 7.48 (d, *J* = 1.9 Hz, 1H), 7.47 – 7.41 (m, 2H), 7.37 – 7.34 (m, 2H), 7.31 (dd, *J* = 9.5, 4.6 Hz, 1H), 7.28 – 7.21 (m, 5H), 6.67 (dd, *J* = 11.6, 8.4 Hz, 1H), 5.32 (d, *J* = 1.7 Hz, 1H), 5.03 (d, *J* = 15.7 Hz, 1H), 4.74 (d, *J* = 15.7 Hz, 1H), 3.51 (s, 1H); ^{13}C NMR (126 MHz, DMSO) δ 175.05, 170.66, 147.07, 142.33, 135.95, 132.53, 130.41, 129.90, 129.65, 129.14, 129.06, 128.57, 127.93, 127.52, 127.41, 127.20, 126.13, 111.53, 81.92, 76.77, 43.23; HRMS(TOF MS ESI$^+$) caculated for $C_{25}H_{18}ClNO_4Na$ [M+Na]$^+$:454.0817, found 454.0814; HPLC conditions for determination enantiomeric excess: Chiral ADH, λ=254 nm, hexane:2-propanol=85:15, flow rate=1.0 mL/min, t_{minor}=27.245, t_{major}= 31.868.

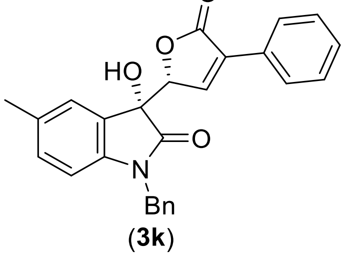

(**3k**)

(*R*)-1-benzyl-3-hydroxy-5-methyl-3-((R)-5-oxo-4-phenyl-2,5-dihydrofuran-2-yl)indolin-2-one (3k): White solid, mp: 90.6-91.7℃; 91% yield, 81:19 *dr*, 93% (95%) *ee*, $[\alpha]_D^{20}$ =66.8 (c=0.033,CH_2Cl_2); ^{1}H NMR (500 MHz, DMSO) δ 8.29 (d, *J* = 1.6 Hz, 1H), 7.88 (d, *J* = 6.9 Hz, 2H), 7.49 – 7.46 (m, 1H), 7.46 – 7.40 (m, 2H), 7.33 (dd, *J* = 12.7, 5.0 Hz, 2H), 7.30 – 7.21 (m,

3H), 7.05 (dd, J = 16.5, 5.2 Hz, 1H), 7.01 (d, J = 8.7 Hz, 2H), 6.72 (d, J = 7.9 Hz, 1H), 5.50 (d, J = 1.6 Hz, 1H), 4.97 (d, J = 16.0 Hz, 1H), 4.85 (d, J = 16.0 Hz, 1H), 2.10 (s, 3H); ^{13}C NMR (126 MHz, DMSO) δ 175.30, 170.76, 147.37, 141.07, 136.38, 132.42, 131.40, 130.66, 129.80, 129.13, 129.06, 128.98, 127.78, 127.51, 127.37, 126.80, 126.52, 109.73 , 82.09, 76.89, 43.12, 20.97; HRMS(TOF MS ESI$^+$) caculated for $C_{26}H_{21}NO_4Na$ [M+Na]$^+$:434.1363, found 434.1362; HPLC conditions for determination enantiomeric excess: Chiral ODH, λ=254 nm, hexane:2-propanol=70:30, flow rate=1.0 mL/min, t_{minor}=18.739, t_{major}= 12.731.

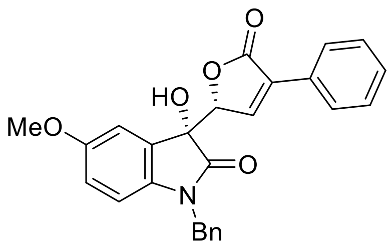

(**3l**)

(*R*)-1-benzyl-3-hydroxy-5-methoxy-3-((R)-5-oxo-4-phenyl-2,5-dihydrofuran-2-yl)indolin-2-one (3l)：White solid, mp: 168.8-169.7℃; 89% yield, 83:17 *dr*, 91% (93%) *ee*, $[\alpha]_D^{20}$=124.1 (c=0.033,CH_2Cl_2); ^{1}H NMR (400 MHz, DMSO) δ 8.35 (d, J = 1.6 Hz, 1H), 7.92 (d, J = 6.3 Hz, 2H), 7.49 – 7.42 (m, 3H), 7.35 (d, J = 7.3 Hz, 2H), 7.32 – 7.25 (m, 3H), 7.07 (s, 1H), 6.81 – 6.72 (m, 3H), 5.52 (d, J = 1.5 Hz, 1H), 4.97 (d, J = 15.9 Hz, 1H), 4.85 (d, J = 16.0 Hz, 1H), 3.52 (s, 3H); ^{13}C NMR (126 MHz, DMSO) δ 175.12, 170.66, 155.31, 147.34, 136.70, 136.39, 132.31, 129.86, 129.71, 129.15, 128.98, 127.79, 127.54, 127.31, 115.08, 113.08, 110.49, 82.03, 77.05, 55.86, 43.17; HRMS(TOF MS ESI$^+$) caculated for $C_{26}H_{21}NO_5Na$ [M+Na]$^+$:450.1312, found 450.1312; HPLC conditions for determination enantiomeric excess: Chiral ADH, λ=254 nm, hexane:2-propanol=80:20, flow rate=1.0 mL/min, t_{minor}=26.587, t_{major}= 29.761.

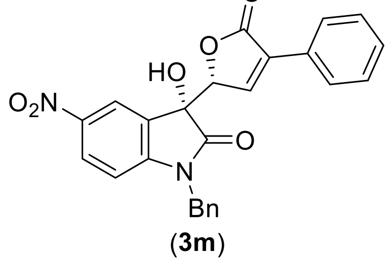

(**3m**)

(*R*)-1-benzyl-3-hydroxy-5-nitro-3-((R)-5-oxo-4-phenyl-2,5-dihydrofuran-2-yl)indolin-2-one (3m): White solid, mp: 191.4-192.4℃; 99% yield, 60:40 *dr*, 80% (89%) *ee*, $[\alpha]_D^{20}$ =177.9 (c=0.033,CH_2Cl_2); ^{1}H NMR (500 MHz, DMSO) 8.40 (d, *J* = 1.7 Hz, 1H), 8.31 – 8.25 (m, 1H), 8.10 (d, *J* = 2.3 Hz, 1H), 7.89 (dd, *J* = 7.8, 1.4 Hz, 2H), 7.48 – 7.42 (m, 4H), 7.35 (t, *J* = 7.1 Hz, 2H), 7.30 (dt, *J* = 8.9, 6.8 Hz, 3H), 7.14 (d, *J* = 8.8 Hz, 1H), 5.65 (d, *J* = 1.7 Hz, 1H), 5.11 (d, *J* = 16.1 Hz, 1H), 5.03 (d, *J* = 16.2 Hz, 1H); ^{13}C NMR (126 MHz, DMSO) δ 175.76, 170.58, 149.41, 146.79, 142.79, 135.53, 132.78, 129.96, 129.53, 129.16, 129.13, 128.10, 127.86, 127.68, 127.50, 127.45, 121.26, 110.44, 81.76, 76.31, 43.52; HRMS(TOF MS ESI$^+$) caculated for $C_{25}H_{18}N_2O_6Na$ $[M+Na]^+$:465.1057, found 465.1059; HPLC conditions for determination enantiomeric excess: Chiral ADH, λ=254 nm, hexane:2-propanol=75:25, flow rate=1.0 mL/min, t_{minor}=22.835, t_{major}= 26.708.

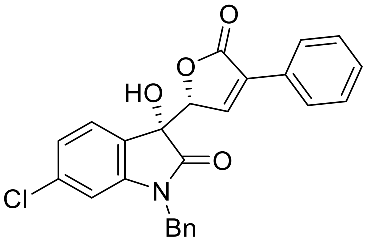

(**3n**)

(*R*)-1-benzyl-6-chloro-3-hydroxy-3-((R)-5-oxo-4-phenyl-2,5-dihydrofuran-2-yl)indolin-2-one (3n): White solid, mp: 198.8-199.7℃; 98% yield, 67:33 *dr*, 91% (93%) *ee*, $[\alpha]_D^{20}$ =111.6 (c=0.033,CH_2Cl_2); ^{1}H NMR (500 MHz, DMSO) δ 8.36 (m, 1H), 7.92 (d, *J* = 6.3 Hz, 2H), 7.45 (d, *J* = 7.1 Hz, 3H), 7.38 – 7.27 (m, 5H), 7.22 – 7.13 (m, 2H), 7.04 (d, *J* = 23.6 Hz, 2H), 5.54 (s, 1H), 5.03 (d, *J* = 16.0 Hz, 1H), 4.92 (d, *J* = 16.0 Hz, 1H); ^{13}C NMR (126 MHz, DMSO) δ 175.51, 170.61, 147.06, 145.06, 135.99, 135.09, 132.38, 129.89, 129.61, 129.16, 129.07, 127.96, 127.55, 127.38, 127.24, 125.31, 122.30, 110.36, 81.87, 76.38, 43.12; HRMS(TOF MS ESI$^+$) caculated for $C_{25}H_{18}ClNO_4Na$ $[M+Na]^+$:454.0817, found 454.0816; HPLC conditions for determination enantiomeric excess: Chiral ODH, λ=254 nm, hexane:2-propanol=80:20, flow rate=1.0 mL/min, t_{minor}=28.976, t_{major}= 20.273.

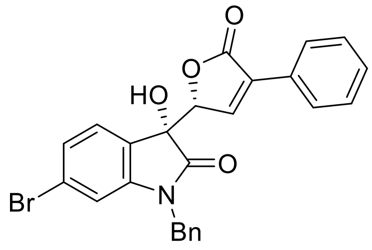

(**3o**)

(*R*)-1-benzyl-6-bromo-3-hydroxy-3-((R)-5-oxo-4-phenyl-2,5-dihydrofuran-2-yl)indolin-2-one (3o)：White solid, mp: 206.5-207.5℃; 99% yield, 62:38 *dr*, 88% (95%) *ee*, $[\alpha]_D^{20}$ =150.7 (c=0.033,CH_2Cl_2); ^1H NMR (400 MHz, DMSO) δ 8.34 (d, *J* = 1.8 Hz, 1H), 7.91 (dd, *J* = 7.7, 1.6 Hz, 2H), 7.45 (q, *J* = 5.6 Hz, 3H), 7.38 – 7.31 (m, 4H), 7.31 – 7.27 (m, 1H), 7.18 – 7.09 (m, 4H), 5.53 (d, *J* = 1.8 Hz, 1H), 5.03 (d, *J* = 16.0 Hz, 1H), 4.93 (t, *J* = 15.2 Hz, 1H); ^{13}C NMR (126 MHz, DMSO) δ 175.41, 170.61, 147.05, 145.13, 135.99, 132.38, 129.89, 129.61, 129.16, 129.07, 127.95, 127.52, 127.38, 125.75, 125.25, 123.54, 113.05, 81.82, 76.43, 43.09; HRMS(TOF MS ESI$^+$) caculated for $C_{25}H_{18}BrNO_4Na$ $[M+Na]^+$:498.0311, found 498.0306; HPLC conditions for determination enantiomeric excess: Chiral ODH, λ=254 nm, hexane:2-propanol=90:10, flow rate=1.0 mL/min, t_{minor}=78.857, t_{major}= 57.193.

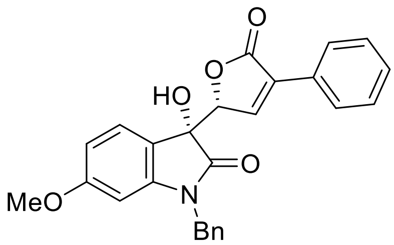

(**3p**)

(*R*)-1-benzyl-3-hydroxy-6-methoxy-3-((R)-5-oxo-4-phenyl-2,5-dihydrofuran-2-yl)indolin-2-one (3p)：White solid, mp: 146.1-147.9℃; 88% yield, 90:10 *dr*, 90% (90%) *ee*, $[\alpha]_D^{20}$ =67.9 (c=0.033,CH_2Cl_2); ^1H NMR (400 MHz, DMSO) δ 8.33 (d, *J* = 1.7 Hz, 1H), 7.94 – 7.88 (m, 2H), 7.49 – 7.42 (m, 3H), 7.37 (d, *J* = 7.0 Hz, 2H), 7.33 – 7.24 (m, 3H), 7.08 – 7.00 (m, 1H), 6.93 (s, 1H), 6.45 (dd, *J* = 5.9, 2.1 Hz, 2H), 5.48 (d, *J* = 1.6 Hz, 1H), 4.98 (d, *J* = 15.9 Hz, 1H), 4.88 (d, *J* = 15.9 Hz, 1H), 3.64 (s, 3H); ^{13}C NMR (126 MHz, DMSO) δ 175.99, 170.72, 161.49, 147.39, 145.02, 136.43, 132.24, 129.80, 129.76, 129.15, 128.99, 127.83, 127.61, 127.33, 126.81, 118.04, 106.26, 97.91, 82.05, 76.43, 55.78, 43.07; HRMS(TOF MS ESI$^+$) caculated for

$C_{26}H_{21}NO_5Na$ $[M+Na]^+$:450.1312, found 450.1313; HPLC conditions for determination enantiomeric excess: Chiral IA, λ=254 nm, hexane:2-propanol=85:15, flow rate=1.0 mL/min, t_{minor}=30.786, t_{major}= 36.492.

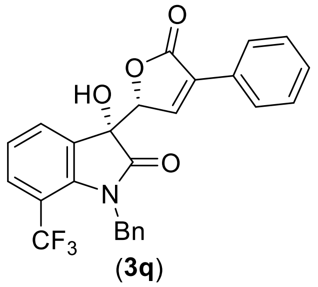

(**3q**)

(*R*)-1-benzyl-3-hydroxy-3-((*R*)-5-oxo-4-phenyl-2,5-dihydrofuran-2-yl)-7-(trifluoromethyl)indolin-2-one (3q)：Faint yellow solid, mp: 177.2-179.1℃; 98% yield, 73:27 *dr*, 88% (92%) *ee*, $[\alpha]_D^{20}$=106.7 (c=0.033,CH_2Cl_2); ^1H NMR (400 MHz, DMSO) δ 8.40 (d, *J* = 1.6 Hz, 1H) , 7.95 (dd, *J* = 10.2, 8.5 Hz, 2H), 7.65 (d, *J* = 8.1 Hz, 1H), 7.57 (d, *J* = 7.2 Hz, 1H), 7.50 – 7.36 (m, 4H), 7.31 – 7.18 (m, 6H), 5.60 (d, *J* = 1.5 Hz, 1H), 5.19 (d, *J* = 17.7 Hz, 1H), 5.06 (d, *J* = 17.7 Hz, 1H); ^{13}C NMR (101 MHz, DMSO) δ 177.04, 170.53, 146.88, 141.59, 136.47, 132.50, 130.36, 129.95, 129.73, 129.16, 128.81, 128.70, δ 128.07(q, *J* = 205.8 Hz)127.41, 127.21, 125.68, 125.41, 122.85, 111.81(d, *J* = 32.6 Hz), 81.92, 74.72, 45.60; ^{19}F NMR (376 MHz, DMSO) δ -53.63; HRMS(TOF MS ESI^+) caculated for $C_{26}H_{18}F_3NO_4Na$ $[M+Na]^+$:488.1080, found 488.1080; HPLC conditions for determination enantiomeric excess: Chiral ADH, λ=254 nm, hexane:2-propanol=90:10, flow rate=1.0 mL/min, t_{minor}=34.940, t_{major}= 24.565.

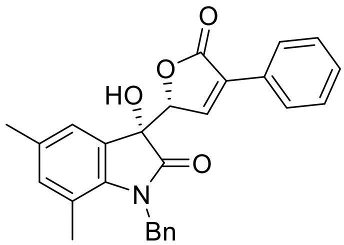

(**3r**)

(*R*)-1-benzyl-3-hydroxy-5,7-dimethyl-3-((R)-5-oxo-4-phenyl-2,5-dihydrofuran-2-yl)indolin-2-one (3r)：Pink solid, mp: 132.2-133.6℃; 91% yield, 78:22*dr*, 92% (94%) *ee*, $[\alpha]_D^{20}$ =90.0 (c=0.033,CH_2Cl_2); ^1H NMR (400 MHz, DMSO) δ 8.31 (d, *J* = 1.4 Hz, 1H), 7.88 (d, *J* =

14.4, 2H), 7.49 – 7.39 (m, 3H), 7.29 (dt, *J* = 14.8, 7.3 Hz, 5H), 6.99 (s, 1H), 6.87 (m, 1H), 6.80 (m, 1H), 5.51 (d, *J* = 1.4 Hz, 1H), 5.20 (d, *J* = 17.3 Hz, 1H), 5.08 (d, *J* = 17.3 Hz, 1H), 2.10 (s, 3H), 2.07 (s, 3H); ^{13}C NMR (101 MHz, DMSO) δ 176.34, 170.80, 147.47, 138.97, 138.17, 134.61, 132.45, 131.41, 129.83, 129.79, 129.19, 129.12, 129.07, 127.48, 127.38, 126.02, 124.84, 119.85, 82.26, 76.19, 44.60, 20.66, 18.33; HRMS(TOF MS ESI$^+$) caculated for $C_{27}H_{23}NO_4Na$ [M+Na]$^+$:448.1519, found 448.1519; HPLC conditions for determination enantiomeric excess: Chiral ADH, λ=254 nm, hexane:2-propanol=80:20, flow rate=1.0 mL/min, t_{minor}=17.287, t_{major}= 22.745.

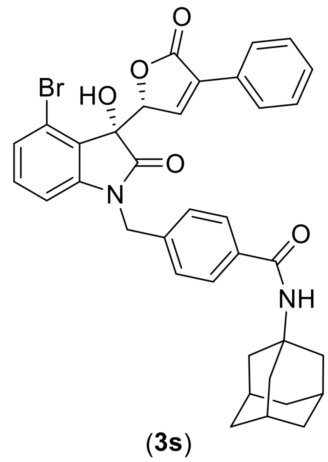

(**3s**)

***N*-((3S,5S,7S)-adamantan-1-yl)-4-(((*R*)-4-bromo-3-hydroxy-2-oxo-3-((R)-5-oxo-4-phenyl-2,5-dihydrofuran-2-yl)indolin-1-yl)methyl)benzamide (3s)**：White solid, mp: 210.6-211.8℃; 88% yield, >20:1 *dr*, 90% *ee*, $[\alpha]_D^{20}$=35.3(c=0.033,CH_2Cl_2); ^{1}H NMR (400 MHz, DMSO) δ 8.19 (d, J = 1.9 Hz, 1H), 7.90 (dd, J = 7.6, 1.8 Hz, 2H), 7.61 (d, J = 8.2 Hz, 2H), 7.53 (s, 1H), 7.44 (dt, J = 11.4, 4.1 Hz, 3H), 7.36 (d, J = 8.2 Hz, 2H), 7.20 – 7.10 (m, 3H), 6.82 (d, J = 7.5 Hz, 1H), 5.66 (d, J = 1.9 Hz, 1H), 5.08 (d, J = 16.3 Hz, 1H), 4.84 (d, J = 16.3 Hz, 1H), 2.05 (s, 9H), 1.65 (s, 6H); ^{13}C NMR (101 MHz, DMSO) δ 175.04, 170.66, 166.18, 147.39, 145.38, 138.54, 135.71, 132.42, 130.11, 129.75, 129.72, 129.07, 128.08, 127.60, 127.30, 127.15, 124.83, 119.66, 109.48, 82.40, 78.68, 51.92, 43.06, 41.34, 36.57, 29.37; HRMS(TOF MS ESI$^+$) caculated for $C_{36}H_{33}BrN_2O_5Na$ [M+Na]$^+$:675.1465, found 675.1466; HPLC conditions for determination enantiomeric excess: Chiral IA, λ=254 nm, hexane:2-propanol=70:30, flow rate=1.0 mL/min, t_{minor}=42.352, t_{major}=18.017.

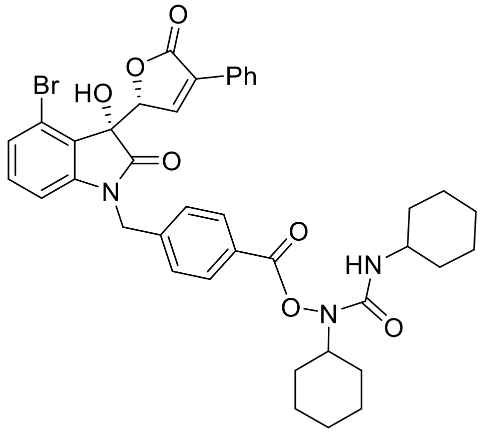

(**3t**)

1-((4-(((*R*)-4-bromo-3-hydroxy-2-oxo-3-((*R*)-5-oxo-4-phenyl-2,5-dihydrofuran-2-yl)indolin-1-yl)methyl)benzoyl)oxy)-1,3-dicyclohexylurea (3t)： White solid, mp: 199.9-201.8℃; 95% yield, >20:1 *dr*, >99% *ee*, $[\alpha]_D^{20}$ =166.0 (c=0.033,CH_2Cl_2); ^1H NMR (500 MHz, DMSO) δ 8.23 (s, 1H), 7.92 (dd, *J* = 6.7, 1.6 Hz, 2H), 7.80 (d, *J* = 7.9 Hz, 1H), 7.43 (dt, *J* = 10.9, 7.6 Hz, 3H), 7.40 – 7.34 (m, 2H), 7.32 (d, *J* = 7.8 Hz, 2H), 7.20 (d, *J* = 3.9 Hz, 1H), 7.12 (d, *J* = 4.2 Hz, 2H), 6.74 (dd, *J* = 8.5, 4.2 Hz, 1H), 5.64 (d, *J* = 1.8 Hz, 1H), 5.05 (d, *J* = 16.3 Hz, 1H), 4.84 (d, *J* = 16.4 Hz, 1H), 4.22 – 4.08 (m, 1H), 3.15 – 3.03 (m, 1H), 1.78 (m, 3H), 1.71 – 1.59 (m, 3H), 1.39 (t, *J* = 12.6 Hz, 3H), 1.28 (dd, *J* = 27.0, 14.2 Hz, 3H), 1.18 (m, 2H), 1.04 (dd, *J* = 33.3, 12.7 Hz, 3H), 0.89 (dd, *J* = 20.6, 10.7 Hz, 1H), 0.74 – 0.61 (m, 2H); ^{13}C NMR (126 MHz, DMSO) δ 175.11, 170.59, 168.17, 154.22, 147.38, 145.34, 137.78, 136.99, 132.23, 129.97, 129.74, 129.71, 129.08, 127.73, 127.59, 127.24, 126.74, 124.80, 119.69, 109.30, 82.42, 78.71, 53.92, 49.42, 42.94, 31.66, 31.63, 30.88, 30.70, 26.02, 25.60, 25.42, 24.58, 24.54; HRMS(TOF MS ESI$^+$) caculated for $C_{39}H_{40}BrN_3O_7Na$ $[M+Na]^+$:764.1942, found 764.1945; HPLC conditions for determination enantiomeric excess: Chiral ODH, λ=254 nm, hexane:2-propanol=75:25, flow rate=1.0 mL/min, t_{major}= 20.467.

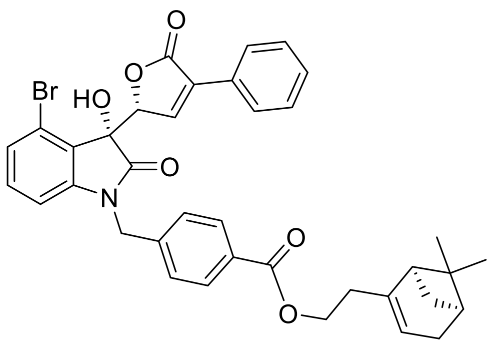

(**3u**)

2-((1*R*,5*S*)-6,6-dimethylbicyclo[3.1.1]hept-2-en-2-yl)ethyl 4-(((*R*)-4-bromo-3-hydroxy-2-oxo-3-((*R*)-5-oxo-4-phenyl-2,5-dihydrofuran-2-yl)indolin-1-yl)methyl)benzoate (3u)：White solid, mp: 220.5-222.8℃; 90% yield, 92:8 *dr*, $[\alpha]_D^{20}$=101.2 (c=0.033,CH_2Cl_2); ^{1}H NMR (500 MHz, DMSO) 8.18 (d, *J* = 1.5 Hz, 1H), 7.93 – 7.89 (m, 2H), 7.76 (d, *J* = 8.2 Hz, 2H), 7.49 – 7.35 (m, 5H), 7.22 – 7.11 (m, 3H), 6.86 (d, *J* = 7.5 Hz, 1H), 5.68 (d, *J* = 1.4 Hz, 1H), 5.35 (m, 1H), 5.12 (d, *J* = 16.5 Hz, 1H), 4.88 (d, *J* = 16.5 Hz, 2H), 4.30 – 4.20 (m, 2H), 2.36 (dd, *J* = 13.7, 5.4 Hz, 3H), 2.23 (d, *J* = 17.5 Hz, 1H), 2.14 (dd, *J* = 12.3, 6.8 Hz, 2H), 2.05 (m, 1H), 1.23 (s, 3H), 1.09 (d, *J* = 8.4 Hz, 1H), 0.77 (s, 3H); ^{13}C NMR (126 MHz, DMSO) δ 174.94, 170.67, 165.73, 147.19, 145.29, 144.73, 141.52, 132.52, 130.25, 129.75, 129.73, 129.69, 129.43, 129.03, 127.73, 127.68, 127.29, 124.83, 119.71, 118.85, 109.40, 82.33, 78.67, 63.31, 45.40, 43.05, 37.97, 35.92, 31.67, 31.38, 26.47, 21.44.; HRMS(TOF MS ESI$^+$) caculated for $C_{37}H_{34}BrNO_6Na$ $[M+Na]^+$:690.1462, found 690.1460.

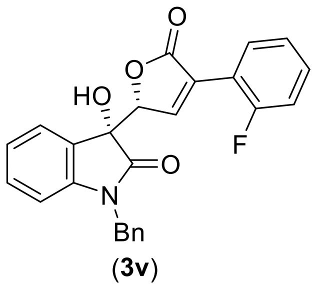

(**3v**)

(*R*)-1-benzyl-3-((R)-4-(2-fluorophenyl)-5-oxo-2,5-dihydrofuran-2-yl)-3-hydroxyindolin-2-one (3v): Pink solid, mp: 143.6-145.5℃; 82% yield, 83:17*dr*, 94% *ee*, $[\alpha]_D^{20}$ =55.8 (c=0.033,CH_2Cl_2); ^{1}H NMR (400 MHz, DMSO) δ 8.22 (m, 1H), 7.87 (t, *J* = 7.1 Hz, 1H), 7.50 (dd, *J* = 13.0, 6.2 Hz, 1H), 7.38 (dd, *J* = 12.6, 6.2 Hz, 3H), 7.34 – 7.22 (m, 5H), 7.18 (d, *J* = 7.2 Hz,

1H), 7.09 (s, 1H), 6.96 (t, *J* = 7.5 Hz, 1H), 6.88 (d, *J* = 7.8 Hz, 1H), 5.58 (d, *J* = 1.4 Hz, 1H), 4.99 (d, *J* = 16.0 Hz, 1H), 4.91 (d, *J* = 16.0 Hz, 1H); ^{13}C NMR (101 MHz, DMSO) δ 175.33, 170.34, 160.53(d, *J* = 250.2 Hz), 150.90(d, *J* = 8.7 Hz), 143.47, 136.32, 131.83(d, *J* = 8.1 Hz), 130.70, 129.81, 129.03, 127.88, 127.59, 127.03, 126.34, 125.70, 125.11, 122.57, 117.69(d, *J* = 13.0 Hz), 116.53(d, *J* = 21.5 Hz), 110.06, 82.46, 76.60, 43.18; ^{19}F NMR (376 MHz, DMSO) δ -113.28; HRMS(TOF MS ESI$^+$) caculated for $C_{25}H_{18}FNO_4Na$ $[M+Na]^+$:438.1112, found 438.1112; HPLC conditions for determination enantiomeric excess: Chiral IC, λ=254 nm, hexane:2-propanol=85:15, flow rate=1.0 mL/min, t_{minor}=46.277, t_{major}=28.454.

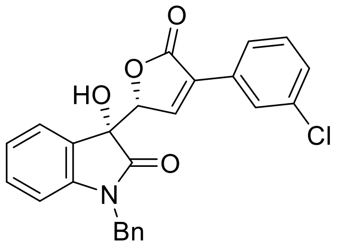

(**3w**)

(*R*)-1-benzyl-3-((R)-4-(3-chlorophenyl)-5-oxo-2,5-dihydrofuran-2-yl)-3-hydroxyindolin-2-one (3w): White solid, mp: 149.6-151.6℃; 95% yield, 79:21 *dr*, 91% (90%) *ee*, $[\alpha]_D^{20}$ =144.7 (c=0.033,CH_2Cl_2); ^1H NMR (500 MHz, DMSO) δ 8.46 (d, *J* = 5.0 Hz, 1H), 7.97 (dd, *J* = 8.4, 6.9 Hz, 2H), 7.57 – 7.49 (m, 2H), 7.39 – 7.22 (m, 6H), 7.12 – 7.06 (m, 1H), 6.90 (ddd, *J* = 19.1, 14.2, 7.1 Hz, 2H), 5.54 (d, *J* = 4.9 Hz, 1H), 5.01 (d, *J* = 16.0 Hz, 1H), 4.92 (d, *J* = 16.5 Hz, 1H); ^{13}C NMR (126 MHz, DMSO) δ 175.42, 170.50, 148.11, 143.51, 136.32, 134.56, 131.06, 130.64, 129.24, 129.16, 129.10, 129.02, 128.91, 128.55, 127.85, 127.66, 127.56, 125.86, 122.52, 110.02, 82.12, 76.73, 43.13; HRMS(TOF MS ESI$^+$) caculated for $C_{25}H_{18}ClNO_4Na$ $[M+Na]^+$:454.0817, found 454.0818; HPLC conditions for determination enantiomeric excess: Chiral ODH, λ=254 nm, hexane:2-propanol=75:25, flow rate=1.0 mL/min, t_{minor}=25.348, t_{major}=20.424.

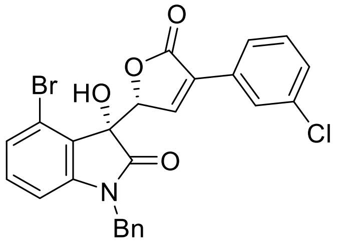

(**3x**)

(*R*)-1-benzyl-4-bromo-3-((R)-4-(3-chlorophenyl)-5-oxo-2,5-dihydrofuran-2-yl)-3-hydroxyindolin-2-one (3x): White solid, mp: 181.1-183.6℃; 98% yield, 95:5 *dr*, 90% *ee*, $[\alpha]_D^{20}$ =76.1 (c=0.033,CH_2Cl_2); ^{1}H NMR (500 MHz, DMSO) δ 8.27 (d, *J* = 1.6 Hz, 1H), 7.95 (d, *J* = 8.5 Hz, 2H), 7.51 (d, *J* = 8.6 Hz, 2H), 7.34 – 7.29 (m, 2H), 7.25 – 7.20 (m, 3H), 7.20 – 7.16 (m, 2H), 7.12 (d, *J* = 8.1 Hz, 1H), 6.87 (d, *J* = 7.7 Hz, 1H), 5.67 (d, *J* = 1.6 Hz, 1H), 5.01 (d, *J* = 16.0 Hz, 1H), 4.81 (d, *J* = 16.0 Hz, 1H); ^{13}C NMR (126 MHz, DMSO) δ 174.91, 170.53, 148.16, 145.52, 135.91, 134.48, 132.51, 129.16, 129.08, 129.04, 128.96, 128.60, 127.87, 127.52, 124.77, 119.58, 109.52, 82.42, 78.59, 43.27; HRMS(TOF MS ESI$^+$) caculated for $C_{25}H_{17}BrClNO_4Na$ $[M+Na]^+$:531.9922, found 531.9929; HPLC conditions for determination enantiomeric excess: Chiral IC, λ=254 nm, hexane:2-propanol=80:20, flow rate=1.0 mL/min, t_{minor}=29.790, t_{major}=24.345.

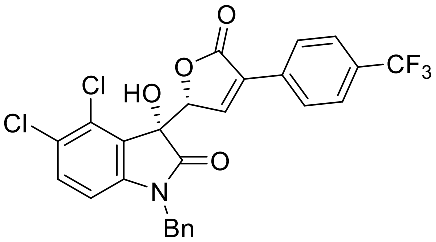

(**3y**)

(*R*)-1-benzyl-4,5-dichloro-3-hydroxy-3-((*R*)-5-oxo-4-(4-(trifluoromethyl)phenyl)-2,5-dihydrofuran-2-yl)indolin-2-one (3y): White solid, mp: 205.5-206.8℃; 90% yield, 92:8 *dr*, 90% *ee*, $[\alpha]_D^{20}$ =177.9 (c=0.033,CH_2Cl_2); ^{1}H NMR (500 MHz, DMSO) δ 8.47 (m, 1H), 8.25 (m, 1H), 8.21 (d, *J* = 7.8 Hz, 1H), 7.80 (d, *J* = 7.7 Hz, 1H), 7.71 (t, *J* = 7.8 Hz, 1H), 7.59 (d, *J* = 8.4 Hz, 1H), 7.35 (s, 1H), 7.27 (d, *J* = 7.3 Hz, 2H), 7.18 (dq, *J* = 14.1, 7.1 Hz, 3H), 6.92 (d, *J* = 8.4 Hz, 1H), 5.78 (s, 1H), 5.03 (d, *J* = 16.0 Hz, 1H), 4.81 (d, *J* = 16.1 Hz, 1H); ^{13}C NMR (126 MHz, DMSO) δ 174.35, 170.53, 149.38, 143.84, 135.67, 132.64, 131.31, 130.66, 130.37, 129.59,

129.28, 128.92, 127.87, 127.45, 126.35(d, *J* = 3.6 Hz), 125.78(d, *J* = 127.0 Hz), 123.56(d, *J* = 3.7 Hz), 124.47(d, *J* = 272.3 Hz), 110.28, 82.32, 78.59, 43.30; ^{19}F NMR (376 MHz, DMSO) δ -61.26; HRMS(TOF MS ESI^{+}) caculated for $C_{26}H_{16}Cl_2F_3NO_4Na$ [M+Na]$^{+}$:556.0301, found 556.0301; HPLC conditions for determination enantiomeric excess:Chiral IC, λ=254 nm, hexane:2-propanol=93:7, flow rate=1.0 mL/min, t_{minor}=34.332, t_{major}= 28.951.

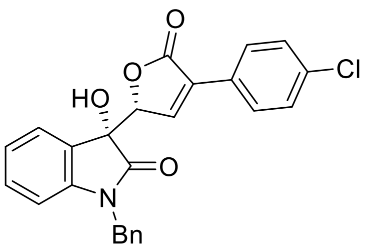

(**3z**)

(*R*)-1-benzyl-3-((R)-4-(4-chlorophenyl)-5-oxo-2,5-dihydrofuran-2-yl)-3-hydroxyindolin-2-one (3z)：White solid, mp: 135.4-136.9℃; 93% yield, 70:30 *dr*, 90% *ee*, $[\alpha]_D^{20}$ =44.8 (c=0.033,CH_2Cl_2); ^{1}H NMR (500 MHz, DMSO) δ 8.45 (d, *J* = 1.1 Hz, 1H), 7.97 (d, *J* = 8.5 Hz, 2H), 7.53 (d, *J* = 8.5 Hz, 2H), 7.37 (d, *J* = 7.4 Hz, 2H), 7.32 (d, *J* = 6.3 Hz, 2H), 7.31 – 7.26 (m, 1H), 7.23 (dd, *J* = 14.2, 6.4 Hz, 1H), 7.14 (d, *J* = 7.3 Hz, 1H), 7.09 (s, 1H), 6.91 (dd, *J* = 14.1, 7.0 Hz, 1H), 6.86 (d, *J* = 7.8 Hz, 1H), 5.53 (d, *J* = 1.1 Hz, 1H), 5.00 (d, *J* = 16.0 Hz, 1H), 4.90 (t, *J* = 16.2 Hz, 1H); ^{13}C NMR (126 MHz, DMSO) δ 175.42, 170.49, 148.10, 143.52, 136.32, 134.56, 131.06, 130.65, 129.24, 129.11, 129.02, 127.85, 127.56, 126.33, 125.87, 122.52, 110.02, 82.12, 76.73, 43.14; HRMS(TOF MS ESI^{+}) caculated for $C_{25}H_{18}ClNO_4Na$ [M+Na]$^{+}$:454.0817, found 454.0819; HPLC conditions for determination enantiomeric excess: Chiral ODH, λ=254 nm, hexane:2-propanol=75:25, flow rate=1.0 mL/min, t_{minor}=26.974, t_{major}=32.350.

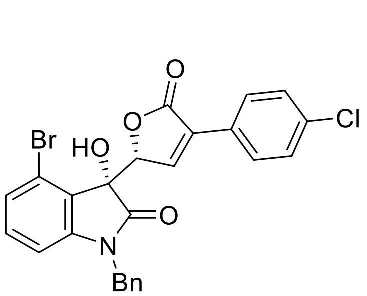

(**3aa**)

(*R*)-1-benzyl-4-bromo-3-((R)-4-(4-chlorophenyl)-5-oxo-2,5-dihydrofuran-2-yl)-3-hydroxyindolin-2-one (3aa): White solid, mp: 183.2-185.6℃; 98% yield, >20:1 *dr*, 91% *ee*, $[\alpha]_D^{20}$ =21.2 (c=0.033,CH_2Cl_2); ^1H NMR (400 MHz, DMSO) δ 8.26 (d, *J* = 1.4 Hz, 1H), 7.94 (d, *J* = 8.5 Hz, 2H), 7.51 (d, *J* = 8.5 Hz, 2H), 7.33 – 7.29 (m, 2H), 7.22 (dd, *J* = 7.9, 4.3 Hz, 3H), 7.18 (d, *J* = 5.1 Hz, 2H), 7.12 (d, *J* = 8.0 Hz, 1H), 6.87 (d, *J* = 7.6 Hz, 1H), 5.67 (d, *J* = 1.3 Hz, 1H), 5.01 (d, *J* = 16.0 Hz, 1H), 4.81 (d, *J* = 16.0 Hz, 1H); ^{13}C NMR (126 MHz, DMSO) δ 174.91, 170.52, 148.14, 145.52, 135.91, 134.49, 132.50, 129.25, 129.16, 129.08, 128.96, 128.60, 127.87, 127.52, 124.78, 119.58, 109.52, 100.00, 82.42, 78.59, 43.28; HRMS(TOF MS ESI^+) caculated for $C_{25}H_{17}BrClNO_4Na$ $[M+Na]^+$:531.9922, found 531.9923; HPLC conditions for determination enantiomeric excess: Chiral IC, λ=254 nm, hexane:2-propanol=80:20, flow rate=1.0 mL/min, t_{minor}=30.399, t_{major}=24.758.

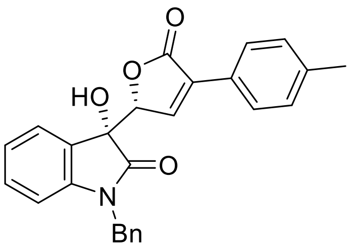

(**3ab**)

(*R*)-1-benzyl-3-hydroxy-3-((*R*)-5-oxo-4-(p-tolyl)-2,5-dihydrofuran-2-yl)indolin-2-one (3ab): White solid, mp: 105.4-107.6℃; 97% yield, 66:34 *dr*, 90% *ee*, $[\alpha]_D^{20}$ =98.2 (c=0.033,CH_2Cl_2); ^1H NMR (500 MHz, DMSO) δ 8.30 (d, *J* = 1.5 Hz, 1H), 7.82 (d, *J* = 8.1 Hz, 2H), 7.37 (d, *J* = 7.4 Hz, 2H), 7.31 (t, *J* = 7.3 Hz, 2H), 7.29 – 7.24 (m, 3H), 7.21 (t, *J* = 7.7 Hz, 1H), 7.12 (d, *J* = 7.3 Hz, 1H), 7.06 (s, 1H), 6.90 (t, *J* = 7.5 Hz, 1H), 6.85 (d, *J* = 7.8 Hz, 1H), 5.50 (d, *J* = 1.4 Hz, 1H), 5.00 (d, *J* = 16.0 Hz, 1H), 4.89 (d, *J* = 16.0 Hz, 1H), 2.34 (s, 3H); ^{13}C NMR (126 MHz, DMSO) δ 175.50, 170.75, 146.18, 143.53, 139.48, 136.33, 132.10, 130.59, 129.72, 129.02, 127.84, 127.56, 127.19, 126.91, 126.38, 125.87, 122.47, 109.98, 81.94, 76.76, 43.12, 21.38; HRMS(TOF MS ESI^+) caculated for $C_{26}H_{21}NO_4Na$ $[M+Na]^+$:434.1363, found 434.1363; HPLC conditions for determination enantiomeric excess: Chiral ADH, λ=254 nm, hexane:2-propanol=82:18, flow rate=1.0 mL/min, t_{minor}=22.849, t_{major}=26.058.

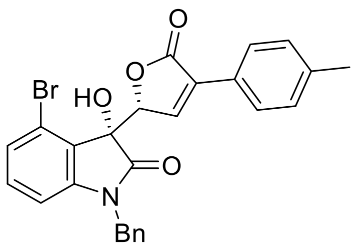

(**3ac**)

(*R*)-1-benzyl-4-bromo-3-hydroxy-3-((R)-5-oxo-4-(p-tolyl)-2,5-dihydrofuran-2-yl)indolin-2-one(3ac)：White solid, mp: 188.3-189.9℃; 99% yield, >20:1 *dr*, 94% (96%) *ee*, $[\alpha]_D^{20}$ =25.8 (c=0.033,CH_2Cl_2); ^{1}H NMR (400 MHz, DMSO) δ 8.08 (d, *J* = 1.9 Hz, 1H), 7.80 (d, *J* = 8.1 Hz, 2H), 7.34 – 7.29 (m, 2H), 7.21 (ddd, *J* = 22.0, 13.2, 8.0 Hz, 6H), 7.15 – 7.08 (m, 2H), 6.85 (d, *J* = 7.5 Hz, 1H), 5.64 (d, *J* = 1.8 Hz, 1H), 5.01 (d, *J* = 16.1 Hz, 1H), 4.80 (d, *J* = 16.1 Hz, 1H), 2.33 (s, 3H); ^{13}C NMR (126 MHz, DMSO) δ 174.98, 170.80, 146.17, 145.50, 139.38, 135.91, 132.43, 130.07, 129.62, 128.97, 127.86, 127.51, 127.19, 126.97, 124.87, 119.62, 109.46, 82.27, 78.65, 43.25, 21.38; HRMS(TOF MS ESI$^+$) caculated for $C_{26}H_{20}BrNO_4Na$ $[M+Na]^+$:512.0468, found 512.0469; HPLC conditions for determination enantiomeric excess: Chiral ADH, λ=254 nm, hexane:2-propanol=75:25, flow rate=1.0 mL/min, t_{minor}=29.564, t_{major}=10.899.

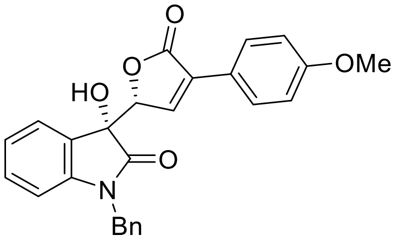

(**3ad**)

(*R*)-1-benzyl-3-hydroxy-3-((R)-4-(4-methoxyphenyl)-5-oxo-2,5-dihydrofuran-2-yl)indolin-2-one (3ad)：White solid, mp: 166.5-168.4℃; 99% yield, 56:44 *dr*, 90% *ee*, $[\alpha]_D^{20}$ =115.6 (c=0.033,CH_2Cl_2); ^{1}H NMR (500 MHz, $CDCl_3$) δ 7.82 (d, *J* = 8.8 Hz, 2H), 7.60 (d, *J* = 1.8 Hz, 1H), 7.32 (d, *J* = 7.4 Hz, 1H), 7.28 – 7.22 (m, 6H), 7.00 (t, *J* = 7.6 Hz, 1H), 6.94 (d, *J* = 8.9 Hz, 2H), 6.74 (d, *J* = 7.9 Hz, 1H), 5.34 (d, *J* = 1.9 Hz, 1H), 5.05 (d, *J* = 15.7 Hz, 1H), 4.75 (d, *J* = 15.7 Hz, 1H), 3.85 (s, 3H), 3.46 (s, 1H); ^{13}C NMR (126 MHz, DMSO) δ 175.53, 170.89, 160.52, 144.66, 143.53, 136.32, 131.70, 130.57, 129.01, 128.77, 127.84, 127.55, 126.40, 125.87, 122.47, 122.16, 114.56, 109.96, 81.89, 76.75, 55.70, 43.10; HRMS(TOF MS ESI$^+$) caculated for $C_{26}H_{21}NO_5Na$ $[M+Na]^+$:450.1312, found 450.1312; HPLC conditions for determination

enantiomeric excess: Chiral ODH, λ=254 nm, hexane:2-propanol=70:30, flow rate=1.0 mL/min, t_{minor}=29.076, t_{major}=47.288.

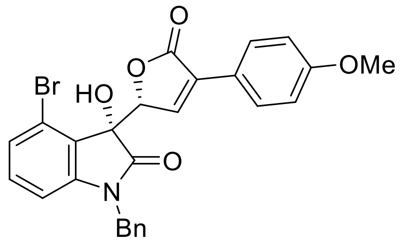

(**3ae**)

(*R*)-1-benzyl-4-bromo-3-hydroxy-3-((R)-4-(4-methoxyphenyl)-5-oxo-2,5-dihydrofuran-2-yl)indolin-2-one (3ae): White solid, mp: 208.3-210.3℃; 99% yield, >20:1 *dr*, 95% *ee*, $[\alpha]_D^{20}$ =188.6 (c=0.033,CH_2Cl_2); ^{1}H NMR (500 MHz, DMSO) δ 8.00 (d, *J* = 1.5 Hz, 1H), 7.87 (d, *J* = 8.7 Hz, 2H), 7.33 – 7.28 (m, 2H), 7.24 – 7.15 (m, 4H), 7.12 (d, *J* = 9.6 Hz, 2H), 6.99 (d, *J* = 8.8 Hz, 2H), 6.85 (d, *J* = 7.6 Hz, 1H), 5.63 (d, *J* = 1.5 Hz, 1H), 5.01 (d, *J* = 16.0 Hz, 1H), 4.79 (d, *J* = 16.0 Hz, 1H), 3.79 (s, 3H); ^{13}C NMR (126 MHz, DMSO) δ 174.97, 170.95, 160.46, 145.50, 144.63, 135.91, 132.41, 129.74, 128.97, 128.80, 127.86, 127.85, 127.50, 124.92, 122.25, 119.61, 114.47, 109.45, 82.22, 78.67, 55.70, 43.25; HRMS(TOF MS ESI$^+$) caculated for $C_{26}H_{20}BrNO_5Na$ [M+Na]$^+$:528.0417, found 528.0417; HPLC conditions for determination enantiomeric excess: Chiral ODH, λ=254 nm, hexane:2-propanol=75:25, flow rate=1.0 mL/min, t_{minor}=34.514, t_{major}=12.351.

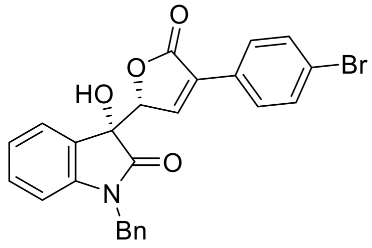

(**3af**)

(*R*)-1-benzyl-3-((R)-4-(4-bromophenyl)-5-oxo-2,5-dihydrofuran-2-yl)-3-hydroxyindolin-2-one (3af): White solid, mp:124.5-126.4℃; 88% yield, 75:25*dr*, 87% (96%) *ee*, $[\alpha]_D^{20}$ =20.8 (c=0.033,CH_2Cl_2); ^{1}H NMR (400 MHz, DMSO) δ 8.45 (d, *J* = 1.3 Hz, 1H), 7.89 (d, *J* = 8.5 Hz, 2H), 7.67 (d, *J* = 8.5 Hz, 2H), 7.37 (d, *J* = 7.3 Hz, 2H), 7.27 (ddd, *J* = 23.3, 15.0, 7.2 Hz, 4H),

7.14 (d, J = 7.3 Hz, 1H), 7.11 – 7.05 (m, 1H), 7.01 – 6.76 (m, 2H), 5.52 (d, J = 1.0 Hz, 1H), 5.00 (d, J = 16.0 Hz, 1H), 4.89 (d, J = 15.9 Hz, 1H); ^{13}C NMR (126 MHz, DMSO) δ 175.41, 170.45, 148.17, 143.51, 136.31, 132.17, 131.14, 130.65, 129.35, 129.02, 128.89, 127.85, 127.56, 126.33, 125.87, 123.32, 122.52, 110.02, 82.14, 76.72, 43.14; HRMS(TOF MS ESI$^+$) caculated for $C_{25}H_{18}BrNO_4Na$ [M+Na]$^+$:498.0311, found 498.0313; HPLC conditions for determination enantiomeric excess: Chiral IC, λ=254 nm, hexane:2-propanol=85:15, flow rate=1.0 mL/min, t_{minor}=51.059, t_{major}=34.871.

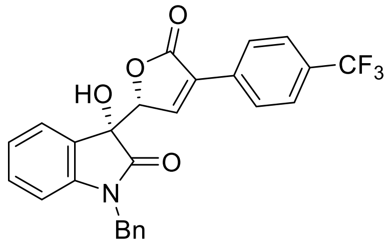

(**3ag**)

(*R*)-1-benzyl-3-hydroxy-3-((*R*)-5-oxo-4-(4-(trifluoromethyl)phenyl)-2,5-dihydrofuran-2-yl)indolin-2-one (3ag)：White solid, mp: 101.7-103.4℃; 88% yield, 80:20 *dr*, 91% (77%) *ee*, $[\alpha]_D^{20}$=155.0 (c=0.033,CH_2Cl_2); ^{1}H NMR (500 MHz, DMSO) δ 8.60 (d, J = 1.6 Hz, 1H), 8.15 (d, J = 8.2 Hz, 2H), 7.84 (d, J = 15.0 Hz, 2H), 7.38 (d, J = 7.2 Hz, 1H), 7.31 (t, J = 7.3 Hz, 3H), 7.27 (d, J = 7.1 Hz, 1H), 7.25 – 7.21 (m, 1H), 7.16 (d, J = 7.4 Hz, 1H), 7.12 (d, J = 4.0 Hz, 1H), 6.91 (ddd, J = 22.8, 11.3, 4.2 Hz, 2H), 5.58 (d, J = 1.6 Hz, 1H), 5.00 (d, J = 16.0 Hz, 1H), 4.90 (t, J = 16.0 Hz, 1H); ^{13}C NMR (126 MHz, DMSO) δ 175.36, 170.31, 150.17, 143.51, 136.31, 133.62, 130.68, 130.05(d, J = 241.3 Hz), 129.01, 128.11, 127.91, 127.85, 127.63, 127.57, 126.29, 126.06(q, J = 5.5 Hz), 125.86, 122.55, 110.05, 82.28, 76.74, 43.15; ^{19}F NMR (376 MHz, DMSO) δ -61.28; HRMS(TOF MS ESI$^+$) caculated for $C_{26}H_{18}F_3NO_4Na$ [M+Na]$^+$:488.1080, found 488.1081; HPLC conditions for determination enantiomeric excess: Chiral ODH, λ=254 nm, hexane:2-propanol=85:15, flow rate=1.0 mL/min, t_{minor}=30.209, t_{major}=25.187.

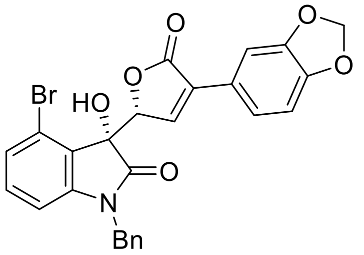

(**3ah**)

(*R*)-3-((*R*)-4-(benzo[d][1,3]dioxol-5-yl)-5-oxo-2,5-dihydrofuran-2-yl)-1-benzyl-4-bromo-3-hydroxyindolin-2-one (3ah)：White solid, mp: 172.1-178.9℃; 97% yield, >20:1 *dr*, 99% *ee*, $[\alpha]_D^{20}$=103.0 (c=0.033,CH_2Cl_2);^{1}H NMR (400 MHz, DMSO) δ 8.05 (d, *J* = 2.0 Hz, 1H), 7.53 (dd, *J* = 8.2, 1.6 Hz, 1H), 7.45 (d, *J* = 1.6 Hz, 1H), 7.31 (dd, *J* = 6.7, 2.7 Hz, 2H), 7.25 – 7.21 (m, 3H), 7.17 (dd, *J* = 17.1, 9.3 Hz, 2H), 7.11 (s, 1H), 6.96 (d, *J* = 8.2 Hz, 1H), 6.86 (d, *J* = 7.5 Hz, 1H), 6.07 (s, 2H), 5.62 (d, *J* = 1.9 Hz, 1H), 5.01 (d, *J* = 16.0 Hz, 1H), 4.79 (d, *J* = 16.1 Hz, 1H); ^{13}C NMR (126 MHz, DMSO) δ 174.92, 170.76, 148.57, 148.04, 145.50, 145.38, 135.92, 132.43, 129.63, 128.96, 127.86, 127.52, 124.88, 123.73, 121.50, 119.62, 109.46, 108.75, 107.52, 101.82, 82.12, 78.63, 43.25; HRMS(TOF MS ESI$^+$) caculated for $C_{26}H_{18}BrNO_6Na$ $[M+Na]^+$:542.0210, found 542.0209; HPLC conditions for determination enantiomeric excess: Chiral ADH, λ=254 nm, hexane:2-propanol=75:25, flow rate=1.0 mL/min, t_{minor}=22.590, t_{major}=30.791.

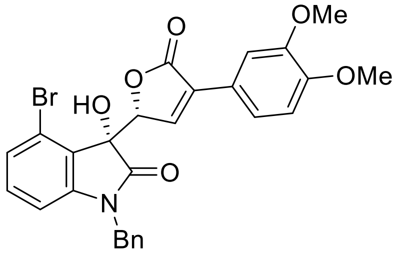

(**3ai**)

(*R*)-1-benzyl-4-bromo-3-((*R*)-4-(3,4-dimethoxyphenyl)-5-oxo-2,5-dihydrofuran-2-yl)-3-hydroxyindolin-2-one (3ai)：White solid, mp: 204.5-206.9℃; 95% yield, 90:10 *dr*, 91% *ee*, $[\alpha]_D^{20}$=157.6 (c=0.033,CH_2Cl_2);^{1}H NMR (400 MHz, DMSO) δ 8.10 (d, *J* = 1.7 Hz, 1H), 7.54 (dd, *J* = 8.4, 1.5 Hz, 1H), 7.48 (m, 1H), 7.35 – 7.28 (m, 2H), 7.25 – 7.09 (m, 6H), 7.00 (d, *J* = 8.5 Hz, 1H), 6.85 (d, *J* = 7.6 Hz, 1H), 5.62 (d, *J* = 1.6 Hz, 1H), 5.02 (d, *J* = 16.0 Hz, 1H), 4.79 (d, *J* = 16.1 Hz, 1H), 3.79 (s, 3H), 3.77 (s, 3H); ^{13}C NMR (126 MHz, DMSO) δ 175.02, 170.91, 150.25,

149.10, 145.54, 144.98, 135.91, 132.43, 129.77, 128.95, 127.84, 127.52, 124.91, 122.41, 120.20, 119.66, 111.94, 110.84, 109.44, 82.20, 78.70, 56.09, 56.00, 43.26; HRMS(TOF MS ESI$^+$) caculated for $C_{27}H_{22}BrNO_6Na$ $[M+Na]^+$:558.0523, found 558.0529; HPLC conditions for determination enantiomeric excess: Chiral ODH, λ=254 nm, hexane:2-propanol=70:30, flow rate=1.0 mL/min, t_{minor}=60.459, t_{major}=18.171.

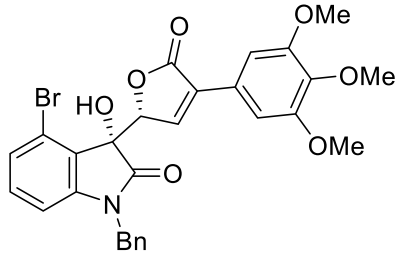

(**3aj**)

(*R*)-1-benzyl-4-bromo-3-hydroxy-3-((*R*)-5-oxo-4-(3,4,5-trimethoxyphenyl)-2,5-dihydrofuran-2-yl)indolin-2-one (3aj)：White solid, mp: 188.8-190.3℃; 98% yield, 84:16 *dr*, 88% *ee*, $[\alpha]_D^{20}$=81.8 (c=0.033,CH_2Cl_2);^{1}H NMR (400 MHz, DMSO) 8.25 (d, *J* = 1.8 Hz, 1H), 7.35 – 7.30 (m, 2H), 7.29 – 7.24 (m, 3H), 7.23 – 7.18 (m, 3H), 7.17 – 7.10 (m, 2H), 6.86 (d, *J* = 7.3 Hz, 1H) , 5.64 (d, *J* = 1.8 Hz, 1H), 5.03 (d, *J* = 16.0 Hz, 1H), 4.79 (d, *J* = 16.2 Hz, 1H), 3.78 (s, 6H), 3.70 (s, 3H); ^{13}C NMR (126 MHz, DMSO) δ 174.99, 170.73, 153.33, 146.87, 145.54, 138.95, 135.91, 132.48, 129.71, 128.92, 127.82, 127.52, 125.25, 124.85, 119.66, 109.47, 104.92, 82.21, 78.65, 60.56, 56.47, 43.27; HRMS(TOF MS ESI$^+$) caculated for $C_{28}H_{24}BrNO_7Na$ $[M+Na]^+$:588.0628, found 588.0628; HPLC conditions for determination enantiomeric excess: Chiral ODH, λ=254 nm, hexane:2-propanol=70:30, flow rate=1.0 mL/min, t_{minor}=46.001, t_{major}=15.468.

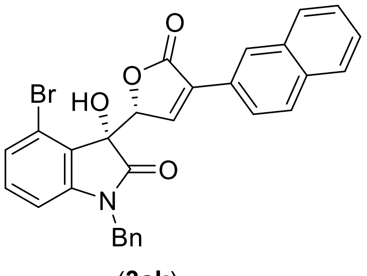

(**3ak**)

(*R*)-1-benzyl-4-bromo-3-hydroxy-3-((*R*)-4-(naphthalen-2-yl)-5-oxo-2,5-dihydrofuran-2-yl)indolin-2-one (3ak)：White solid, mp: 212.5-213.8℃; 91% yield, >20:1 *dr*, 90% *ee*, $[\alpha]_D^{20}$

=178.8 (c=0.033,CH_2Cl_2);^{1}H NMR (500 MHz, DMSO) δ 8.57 (s, 1H), 8.32 (d, J = 1.8 Hz, 1H), 7.97 (ddd, J = 18.4, 10.7, 8.8 Hz, 4H), 7.57 (ddd, J = 6.4, 5.1, 3.4 Hz, 2H), 7.35 – 7.30 (m, 2H), 7.23 – 7.14 (m, 5H), 7.11 (d, J = 8.1 Hz, 1H), 6.87 (d, J = 7.8 Hz, 1H), 5.73 (d, J = 1.8 Hz, 1H), 5.03 (d, J = 16.0 Hz, 1H), 4.81 (d, J = 16.0 Hz, 1H); ^{13}C NMR (126 MHz, DMSO) δ 174.94, 170.78, 147.63, 145.53, 135.91, 133.41, 133.02, 132.48, 130.06, 129.03, 128.94, 128.70, 128.05, 127.82, 127.54, 127.50, 127.22, 127.15, 126.46, 124.99, 124.89, 119.66, 109.51, 82.41, 78.71, 43.28; HRMS(TOF MS ESI$^+$) caculated for $C_{29}H_{20}BrNO_4Na$ [M+Na]$^+$:548.0468, found 548.0472; HPLC conditions for determination enantiomeric excess: Chiral ADH, λ=254 nm, hexane:2-propanol=78:22, flow rate=1.0 mL/min, t_{minor}=23.662, t_{major}=31.637.

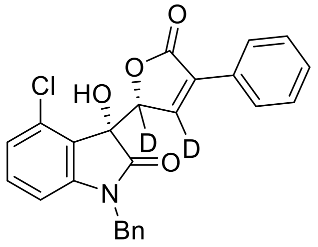

(**3al**)

(*R*)-1-benzyl-4-chloro-3-hydroxy-3-((*R*)-5-oxo-4-phenyl-2,5-dihydrofuran-2-yl-2,3-d2)indolin-2-one (3al): White solid, mp: 129.3-130.8℃; 95% yield, >20:1 *dr*, 90% *ee*, $[\alpha]_D^{20}$ =55.4 (c=0.033,CH_2Cl_2); ^{1}H NMR (500 MHz, DMSO) δ 7.89 (dd, J = 7.9, 1.5 Hz, 2H), 7.46 – 7.40 (m, 3H), 7.33 (dd, J = 6.4, 2.8 Hz, 2H), 7.24 (td, J = 13.1, 8.7 Hz, 5H), 6.96 (d, J = 8.0 Hz, 1H), 6.83 (d, J = 7.8 Hz, 1H), 5.03 (d, J = 16.0 Hz, 1H), 4.82 (d, J = 16.0 Hz, 1H); ^{13}C NMR (126 MHz, DMSO) δ 175.03, 170.72, 145.47, 135.92, 132.33, 131.31, 130.07, 129.74, 129.73, 129.09, 128.98, 127.88, 127.51, 127.25, 124.41, 122.97, 109.02, 78.18, 43.34; HRMS(TOF MS ESI$^+$) caculated for $C_{25}H_{16}D_2ClNO_4Na$ [M+Na]$^+$:456.0942, found 456.0945; HPLC conditions for determination enantiomeric excess: Chiral ODH, λ=254 nm, hexane:2-propanol=70:30, flow rate=1.0 mL/min, t_{minor}=19.457, t_{major}=8.601.

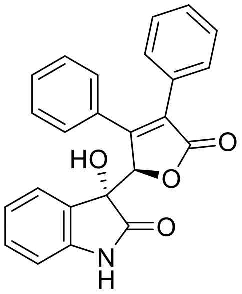

(**3am**)

(*R*)-3-hydroxy-3-((*R*)-5-oxo-3,4-diphenyl-2,5-dihydrofuran-2-yl)indolin-2-one (3am): White solid, mp: 98.7-100.5℃; 76% yield, 84:16 *dr*, 95% *ee*, $[\alpha]_D^{20}$ =63.6 (c=0.033,CH_2Cl_2);^{1}H NMR (500 MHz, DMSO) δ 10.48 (s, 1H), 7.49 – 7.44 (m, 2H), 7.38 – 7.31 (m, 6H), 7.24 (t, *J* = 7.2 Hz, 1H), 7.01 (dd, *J* = 9.4, 5.6 Hz, 3H), 6.94 (t, *J* = 7.4 Hz, 1H), 6.78 (d, *J* = 7.7 Hz, 1H), 6.33 (s, 1H), 6.09 (s, 1H); ^{13}C NMR (126 MHz, DMSO) δ 177.07, 170.84, 159.11, 143.44, 132.02, 130.70, 130.49, 130.05, 129.62, 129.29, 129.00, 128.99, 128.42, 126.40, 125.86, 121.49, 110.39, 82.16, 77.88; HRMS(TOF MS ESI$^+$) caculated for $C_{24}H_{17}NO_4Na$ $[M+Na]^+$:406.1050, found 406.1053; HPLC conditions for determination enantiomeric excess: Chiral IA, λ=254 nm, hexane:2-propanol=85:15, flow rate=1.0 mL/min, t_{minor}=25.276, t_{major}=16.587.

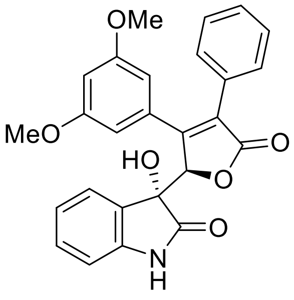

(**3an**)

(*R*)-3-((*R*)-3-(3,5-dimethoxyphenyl)-5-oxo-4-phenyl-2,5-dihydrofuran-2-yl)-3-hydroxyindolin-2-one(3an): White solid, mp: 91.3-92.7℃; 69% yield, 76:24 *dr*, 96% *ee*, $[\alpha]_D^{20}$ =100.9 (c=0.033,CH_2Cl_2);^{1}H NMR (500 MHz, DMSO) δ 10.47 (s, 1H), 7.39 – 7.35 (m, 2H), 7.27 (d, *J* = 3.6 Hz, 1H), 7.23 (t, *J* = 7.6 Hz, 1H), 7.07 – 6.93 (m, 4H), 6.77 (d, *J* = 7.7 Hz, 1H), 6.56 (s, 2H), 6.48 (s, 1H), 6.41 (s, 1H), 6.09 (s, 1H), 3.64 (s, 6H); ^{13}C NMR (126 MHz, DMSO) δ 177.02, 170.81, 160.23, 158.83, 143.37, 133.50, 130.68, 130.58, 129.29, 129.12, 128.97, 128.70, 126.42, 125.93, 121.51, 110.36, 107.95, 101.83, 82.34, 77.88, 55.62; HRMS(TOF MS

ESI$^+$) caculated for $C_{26}H_{21}NO_6Na$ [M+Na]$^+$:466.1261, found 466.1261; HPLC conditions for determination enantiomeric excess: Chiral ADH, λ=254 nm, hexane:2-propanol=80:20, flow rate=1.0 mL/min, t_{minor}=17.984, t_{major}=14.147.

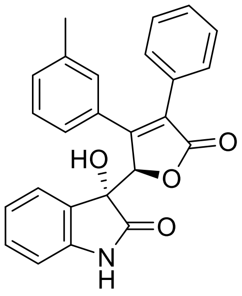

(**3ao**)

(*R*)-3-hydroxy-3-((*R*)-5-oxo-4-phenyl-3-(m-tolyl)-2,5-dihydrofuran-2-yl)indolin-2-one

(3ao)： White solid, mp: 119.0-121.5℃; 78% yield, 80:20 *dr*, 89% *ee*, $[\alpha]_D^{20}$ =89.7 (c=0.033,CH_2Cl_2); ^{1}H NMR (400 MHz, DMSO) δ 10.44 (s, 1H), 7.39 – 7.31 (m, 4H), 7.25 – 7.15 (m, 4H), 7.02 (d, J = 6.3 Hz, 3H), 6.94 (t, J = 7.4 Hz, 1H), 6.77 (d, J = 7.7 Hz, 1H), 6.32 (s, 1H), 6.06 (s, 1H), 2.25 (s, 3H);^{13}C NMR (126 MHz, DMSO) δ 177.07, 170.87, 159.13, 143.41, 137.53, 131.91, 130.71, 130.66, 130.57, 130.29, 129.28, 128.93, 128.85, 128.27, 126.53, 126.42, 125.88, 121.48, 110.36, 82.17, 77.88, 21.40; HRMS(TOF MS ESI$^+$) caculated for $C_{25}H_{19}NO_4Na$ [M+Na]$^+$:420.1206, found 420.1205; HPLC conditions for determination enantiomeric excess: Chiral IA, λ=254 nm, hexane:2-propanol=80:20, flow rate=1.0 mL/min, t_{minor}=15.868, t_{major}=10.048.

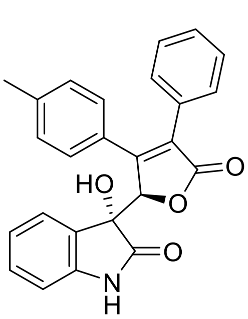

(**3ap**)

(*R*)-3-hydroxy-3-((*R*)-5-oxo-4-phenyl-3-(p-tolyl)-2,5-dihydrofuran-2-yl)indolin-2-one (3ap)： White solid, mp: 135.6-137.0℃; 88% yield, 86:14 *dr*, >99% *ee*, $[\alpha]_D^{20}$=69.7 (c=0.033,CH_2Cl_2);^{1}H

NMR (500 MHz, DMSO) δ 10.48 (s, 1H), 7.42 – 7.31 (m, 5H), 7.24 (dd, *J* = 10.2, 4.5 Hz, 1H), 7.15 (d, *J* = 8.0 Hz, 2H), 7.01 (dd, *J* = 6.1, 2.8 Hz, 2H), 6.97 – 6.91 (m, 2H), 6.79 (d, *J* = 7.7 Hz, 1H), 6.30 (s, 1H), 6.06 (s, 1H), 2.30 (s, 3H); ^{13}C NMR (126 MHz, DMSO) δ 177.15, 170.91, 159.15, 143.48, 139.96, 130.72, 130.66, 129.69, 129.27, 129.11, 129.03, 128.89, 128.22, 126.36, 125.90, 121.45, 110.38, 82.02, 77.91, 21.43; HRMS(TOF MS ESI$^+$) caculated for $C_{25}H_{19}NO_4Na$ [M+Na]$^+$:420.1206, found 420.1206; HPLC conditions for determination enantiomeric excess: Chiral IA, λ=254 nm, hexane:2-propanol=80:20, flow rate=1.0 mL/min, t_{major}=12.473.

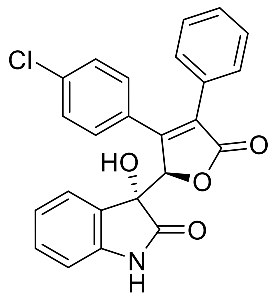

(**3aq**)

(*R*)-3-((*R*)-3-(4-chlorophenyl)-5-oxo-4-phenyl-2,5-dihydrofuran-2-yl)-3-hydroxyindolin-2-one (3aq)：White solid, mp: 100.4-102.0℃; 58% yield, 89:11 *dr*, 95% *ee*, $[\alpha]_D^{20}$ =144.6 (c=0.033,CH_2Cl_2);^{1}H NMR (500 MHz, DMSO) δ 10.50 (s, 1H), 7.47 (d, *J* = 7.9 Hz, 2H), 7.44 – 7.31 (m, 5H), 7.25 (t, *J* = 7.0 Hz, 1H), 7.08 – 6.92 (m, 4H), 6.78 (d, *J* = 7.4 Hz, 1H), 6.39 (s, 1H), 6.10 (s, 1H); ^{13}C NMR (126 MHz, DMSO) δ 176.94, 170.66, 157.76, 143.39, 134.80, 131.42, 130.90, 130.73, 130.10, 129.42, 129.24, 129.18, 129.14, 128.57, 126.32, 125.76, 121.56, 110.43, 82.14, 77.84; HRMS(TOF MS ESI$^+$) caculated for $C_{24}H_{16}ClNO_4Na$ [M+Na]$^+$:440.0660, found 440.0668; HPLC conditions for determination enantiomeric excess: Chiral IA, λ=254 nm, hexane:2-propanol=80:20, flow rate=1.0 mL/min, t_{minor}=23.258, t_{major}=15.526.

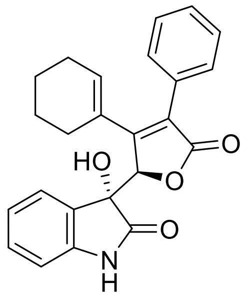

(**3ar**)

(*R*)-3-((*R*)-3-(cyclohex-1-en-1-yl)-5-oxo-4-phenyl-2,5-dihydrofuran-2-yl)-3-hydroxyindolin-2-one(3ar)：White solid, mp: 81.9-83.6℃; 72% yield, 88:12 *dr*, 90% *ee*, $[\alpha]_D^{20}$ =166.7 (c=0.033,CH_2Cl_2);^{1}H NMR (500 MHz, DMSO) δ 10.44 (s, 1H), 7.37 (dd, *J* = 17.9, 7.1 Hz, 3H), 7.26 (t, *J* = 7.2 Hz, 1H), 7.20 (d, *J* = 6.8 Hz, 2H), 7.11 (d, *J* = 6.9 Hz, 1H), 6.96 (t, *J* = 7.2 Hz, 1H), 6.81 (d, *J* = 7.5 Hz, 1H), 6.56 (s, 1H), 5.91 (s, 1H), 5.70 (s, 1H), 2.16 (d, *J* = 15.4 Hz, 1H), 2.01 (d, *J* = 17.3 Hz, 1H), 1.88 (dd, *J* = 30.3, 18.7 Hz, 2H), 1.43 (s, 4H); ^{13}C NMR (126 MHz, DMSO) δ 176.72, 171.34, 160.96, 143.28, 133.25, 131.25, 130.53, 130.47, 129.13, 128.67, 127.24, 126.64, 125.96, 121.60, 110.35, 82.17, 77.51, 27.15, 25.57, 22.23, 21.39; HRMS(TOF MS ESI$^+$) caculated for $C_{24}H_{21}NO_4Na$ $[M+Na]^+$:410.1363, found 410.1368; HPLC conditions for determination enantiomeric excess: Chiral IA, λ=254 nm, hexane:2-propanol=80:20, flow rate=1.0 mL/min, t_{minor}=15.704, t_{major}=10.326.

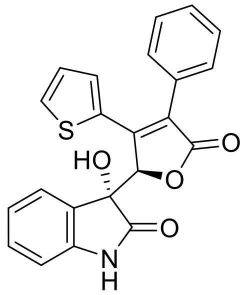

(**3as**)

(*R*)-3-hydroxy-3-((*R*)-5-oxo-4-phenyl-3-(thiophen-2-yl)-2,5-dihydrofuran-2-yl)indolin-2-one (3as)：White solid, mp: 97.7-99.1℃; 61% yield, 95:5 *dr*, 92% *ee*, $[\alpha]_D^{20}$ =149.8 (c=0.033,CH_2Cl_2);^{1}H NMR (500 MHz, DMSO) δ 10.50 (s, 1H), 7.86 (d, *J* = 3.1 Hz, 1H), 7.74 (d, *J* = 4.8 Hz, 1H), 7.46 (d, *J* = 5.7 Hz, 3H), 7.26 (t, *J* = 6.3 Hz, 1H), 7.16 (dd, *J* = 9.8, 5.6 Hz, 3H), 7.01 – 6.94 (m, 2H), 6.83 (d, *J* = 7.7 Hz, 1H), 6.75 (s, 1H), 5.94 (s, 1H); ^{13}C NMR (126 MHz, DMSO) δ 177.27, 170.69, 152.40, 143.71, 134.71, 132.84, 132.12, 130.87, 130.78, 129.99,

129.61, 129.28, 127.75, 126.34, 126.19, 126.03, 121.42, 110.48, 82.09, 77.87; HRMS(TOF MS ESI+) caculated for $C_{22}H_{15}NO_4SNa$ [M+Na]+:412.0614, found 412.0614; HPLC conditions for determination enantiomeric excess: Chiral IG, λ=254 nm, hexane:2-propanol=80:20, flow rate=1.0 mL/min, t_{minor}=33.178, t_{major}=38.231.

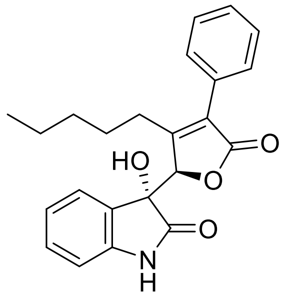

(**3at**)

(*R*)-3-hydroxy-3-((*R*)-5-oxo-3-pentyl-4-phenyl-2,5-dihydrofuran-2-yl)indolin-2-one (3at): White solid, mp: 79.0-81.1℃; 75% yield, 96:4*dr*, 98% *ee*, $[\alpha]_D^{20}$=139.7 (c=0.033,CH_2Cl_2);^{1}H NMR (400 MHz, DMSO) δ 10.49 (s, 1H), 7.43 (dt, *J* = 22.1, 6.5 Hz, 3H), 7.30 (d, *J* = 6.9 Hz, 2H), 7.23 (t, *J* = 7.5 Hz, 1H), 7.05 (d, *J* = 7.1 Hz, 1H), 6.90 (t, *J* = 7.4 Hz, 1H), 6.87 – 6.78 (m, 2H), 5.35 (s, 1H), 3.05 – 2.93 (m, 1H), 2.85 – 2.74 (m, 1H), 1.82 – 1.70 (m, 1H), 1.54 (dd, *J* = 17.1, 10.6 Hz, 1H), 1.24 (dd, *J* = 17.4, 11.4 Hz, 4H), 0.83 (d, *J* = 6.4 Hz, 3H); ^{13}C NMR (126 MHz, DMSO) δ 177.05, 171.06, 164.43, 143.51, 130.69, 130.26, 129.31, 128.93, 128.90, 126.04, 125.92, 121.62, 110.41, 81.60, 77.64, 31.52, 28.53, 27.94, 22.09, 14.21; HRMS(TOF MS ESI+) caculated for $C_{23}H_{23}NO_4Na$ [M+Na]+:400.1519, found 400.1519; HPLC conditions for determination enantiomeric excess: Chiral IA, λ=254 nm, hexane:2-propanol=80:20, flow rate=1.0 mL/min, t_{minor}=8.409, t_{major}=12.003.

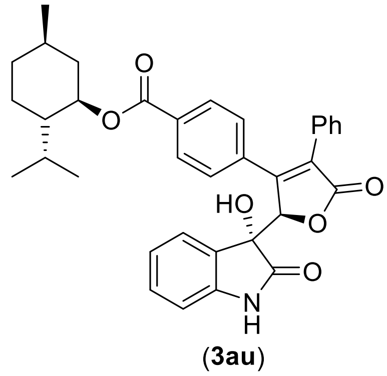

(**3au**)

(1*R*,2*R*,5*R*)-2-isopropyl-5-methylcyclohexyl 4-((R)-2-((R)-3-hydroxy-2-oxoindolin-3-yl)-5-oxo-4-phenyl-2,5-dihydrofuran-3-yl)benzoate (3au)：White solid, mp: 144.8-146.6℃; 68% yield, 87:13 *dr*, $[\alpha]_D^{20}$=-39.1(c=0.033,CH_2Cl_2);^{1}H NMR (500 MHz, DMSO) δ 10.50 (s, 1H), 7.88 (d, *J* = 8.3 Hz, 2H), 7.60 (d, *J* = 8.2 Hz, 2H), 7.37 (d, *J* = 2.5 Hz, 3H), 7.23 (t, *J* = 7.5 Hz, 1H), 7.12 – 6.97 (m, 3H), 6.94 (d, *J* = 7.3 Hz, 1H), 6.77 (d, *J* = 7.7 Hz, 1H), 6.38 (s, 1H), 6.13 (s, 1H), 4.85 (td, *J* = 10.8, 4.3 Hz, 1H), 1.99 (d, *J* = 11.9 Hz, 1H), 1.88 (dt, *J* = 13.9, 6.9 Hz, 1H), 1.69 (d, *J* = 11.0 Hz, 2H), 1.54 (t, *J* = 11.3 Hz, 2H), 1.23 (s, 1H), 1.16 – 1.06 (m, 2H), 0.94 – 0.87 (m, 6H), 0.76 (d, *J* = 6.9 Hz, 3H); ^{13}C NMR (126 MHz, DMSO) δ 176.87, 170.52, 165.20, 157.77, 143.38, 136.75, 130.92, 130.72, 130.30, 129.97, 129.94, 129.88, 129.28, 129.11, 129.08, 126.33, 125.74, 121.57, 110.44, 82.31, 77.82, 74.81, 47.04, 34.18, 31.34, 26.61, 23.57, 22.36, 20.97, 16.85; HRMS(TOF MS ESI$^+$) caculated for $C_{35}H_{35}NO_6Na$ [M+Na]$^+$:588.2357, found 588.2355;

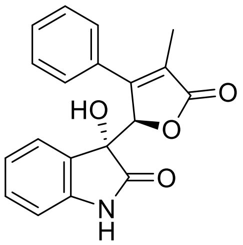

(**3av**)

(*R*)-3-hydroxy-3-((*R*)-4-methyl-5-oxo-3-phenyl-2,5-dihydrofuran-2-yl)indolin-2-one (3av)：White solid, mp: 72.9-74.5℃; 80% yield, 82:18 *dr*, 92% *ee*, $[\alpha]_D^{20}$=129.0 (c=0.033,CH_2Cl_2);^{1}H NMR (400 MHz, DMSO) δ 10.40 (s, 1H), 7.67 (d, *J* = 7.1 Hz, 2H), 7.46 (dt, *J* = 20.0, 6.7 Hz, 3H), 7.24 – 7.13 (m, 1H), 6.94 – 6.84 (m, 2H), 6.74 (d, *J* = 7.6 Hz, 1H), 6.23 (s, 1H), 5.92 (s, 1H), 1.84 (s, 3H); ^{13}C NMR (101 MHz, DMSO) δ 177.17, 172.66, 157.57, 143.35, 132.77, 130.44, 129.70, 129.37, 128.50, 126.54, 125.75, 125.72, 121.62, 110.23, 82.12, 77.61, 10.15; HRMS(TOF MS ESI$^+$) caculated for $C_{19}H_{15}NO_4Na$ [M+Na]$^+$:344.0893, found 344.0899; HPLC conditions for determination enantiomeric excess: Chiral IA, λ=254 nm, hexane:2-propanol=80:20, flow rate=1.0 mL/min, t_{minor}=19.895, t_{major}=13.556.

4. Gram scale amplification reaction and product derivation

(1) Gram scale amplification

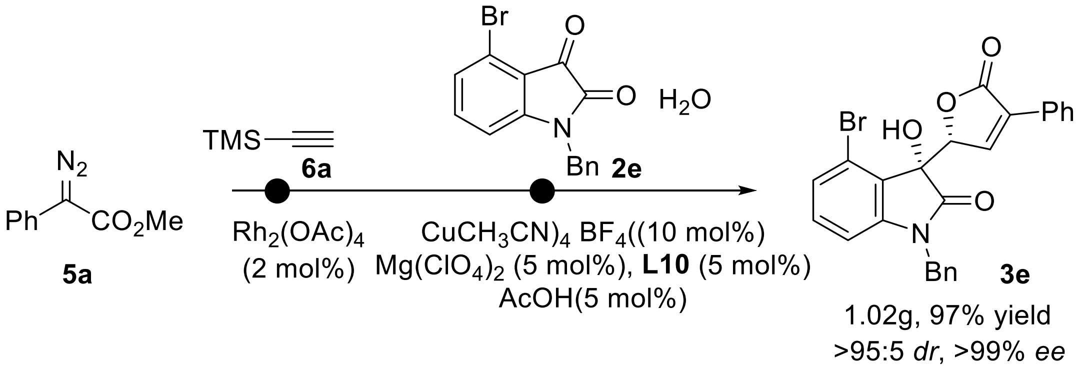

Scheme S2. Gram scale of four-component cascade reaction of methyl phenyldiazoacetate 5a, alkyne 6a, isatin 2e and water enabled by relay catalysis.

Isatin (**2e**, 2.2 mmol, 1eq), $Mg(ClO_4)_2$ (5 mol%), L10 (5 mol%) were dissolved in 20 mL ethyl butyrate. At the same time, the diazo compound (**5a**, 5.5 mmol, 2.5 eq) was dissolved in trimethylsilyne (**6a**, 2 mL) and injected into the suspension of $Rh_2(OAc)_4$ (2 mol%) in trimethylsilyne (**6a**, 3 mL) by injection pump at room temperature within 1 hour and continued to react for 30 minutes. The resulting solution was concentrated and dissolved in 20 mL ethyl butyrate, immediately added to the ligand-activated isatin solution, and then added $Cu(MeCN)_4BF_4$ (10 mol%) and AcOH (5 mol%) (Scheme 2). After the reaction was monitored by TLC (4 hours), the reaction liquid was concentrated to obtain the crude product. The dr value of the was determined by ^{1}H NMR, and the ee value was determined by chiral high performance liquid chromatography. After the determination, the crude product was purified by column chromatography to obtain 1.02g **3e** (petroleum ether: ethyl acetate =2: 1), the yield is 97%, dr is greater than 95:5, ee is greater than 99%.

(2) synthesis of **7a** and **7b**

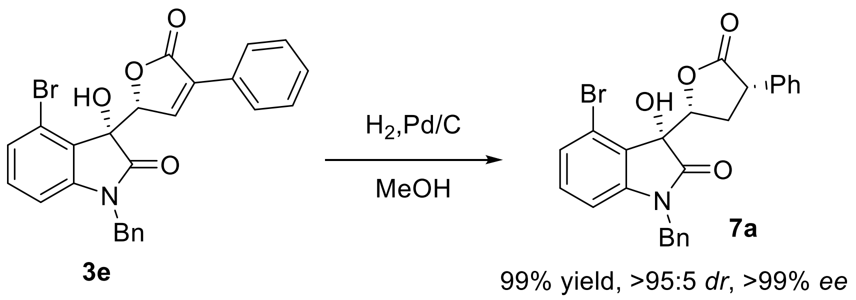

Scheme S3. Palladium/charcoal catalyzed hydrogenation of product 6e.

3e (0.1mmol, 47.6mg) and 10% Pd/C (5mg) were dissolved in 2mL methanol and stirred in

hydrogen atmosphere for reaction (Scheme 3). After the reaction was completed, the reaction liquid was filtered and concentrated to obtain the crude product. The dr value of the crude product was determined by ^{1}H NMR and ee value was determined by chiral high performance liquid chromatography. Then the crude product was purified by column chromatography (petroleum ether: ethyl acetate = 4:1) to obtain **7a** (47.4mg, >95:5 dr, >99% ee).

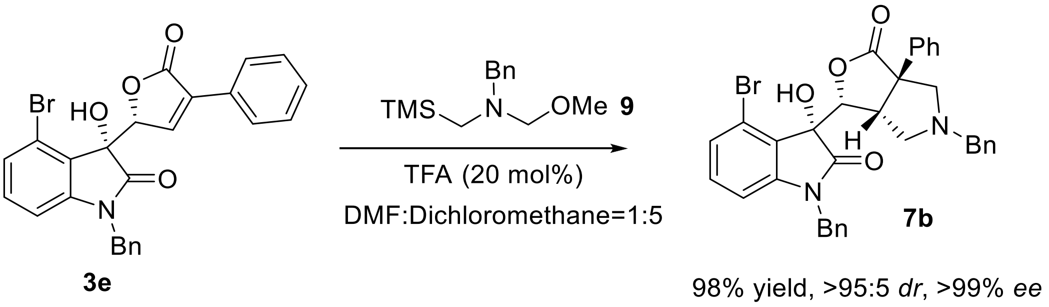

Scheme S4. TFA-catalyzed stereoselective [3+2] cycloaddition of product 3e and dipolar synthon 9.

3e (0.1 mmol, 47.6 mg) and compound **9** (0.5 mmol, 119 mg) were dissolved in 3mL solvent (DMF:DCM=1:5), and TFA (20 mol%) was added under agitation (Scheme 4). After the starting material **3e** was completely consumed, the reaction liquid was concentrated to obtain the crude product. The dr value of the crude product was determined by ^{1}H NMR and ee value was determined by chiral high performance liquid chromatography. Then the crude product was purified by column chromatography (petroleum ether: ethyl acetate = 4:1) to obtain **7b** (47.4mg, >95:5 dr, >99% ee).

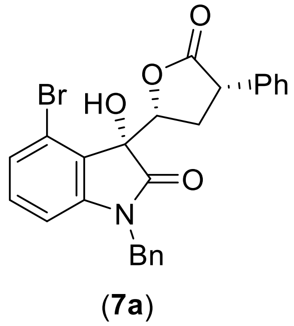

(**7a**)

(*R*)-1-benzyl-4-bromo-3-hydroxy-3-((2*R*,4S)-5-oxo-4-phenyltetrahydrofuran-2-yl)indolin-2-one (7a): White solid, mp: 166.6-168.7℃; 99% yield, >95:5 *dr*, >99% *ee*, $[\alpha]_D^{20}$ =31.0 (c=0.033,CH_2Cl_2); ^{1}H NMR (500 MHz, DMSO) δ 7.52 (d, *J* = 7.4 Hz, 1H), 7.38 – 7.36 (m, 3H), 7.34 – 7.24 (m, 5H), 7.23 (d, *J* = 7.6 Hz, 2H), 7.09 (t, *J* = 7.5 Hz, 1H), 6.91 (d, *J* = 7.9 Hz, 1H), 6.81 (s, 1H), 4.96 (d, *J* = 15.9 Hz, 1H), 4.88 (d, *J* = 15.9 Hz, 1H), 4.79 (dd, *J* = 9.8, 6.3 Hz, 1H),

4.15 (dd, J = 11.8, 9.7 Hz, 1H), 2.74 (ddd, J = 12.8, 9.5, 6.4 Hz, 1H), 2.65 (dd, J = 22.6, 12.3 Hz, 1H);^{13}C NMR (126 MHz, DMSO) δ 176.61, 175.80, 143.08, 138.04, 136.46, 130.25, 129.08, 129.05, 128.82, 128.75, 127.88, 127.72, 127.68, 126.03, 122.94, 109.97, 79.58, 75.38, 45.90, 43.11, 31.47; HRMS(TOF MS ESI$^+$) caculated for $C_{25}H_{20}BrNO_4Na$ [M+Na]$^+$:500.0468, found 500.0468; HPLC conditions for determination enantiomeric excess: Chiral ODH, λ=254 nm, hexane:2-propanol=80:20, flow rate=1.0 mL/min, t_{major}=31.999.

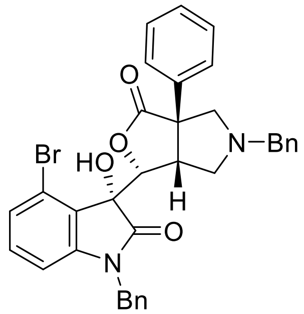

(**7b**)

(3*R*,3a*R*,6a*R*)-5-benzyl-3-((*R*)-1-benzyl-4-bromo-3-hydroxy-2-oxoindolin-3-yl)-6a-phenylhexahydro-1H-furo[3,4-c]pyrrol-1-one (7b)：White solid, mp: 199.5-201.0℃; 98% yield, >95:5 *dr*, >99% *ee*, $[\alpha]_D^{20}$=29.6 (c=0.033,CH_2Cl_2); ^{1}H NMR (400 MHz, DMSO) δ 7.40 – 7.32 (m, 5H), 7.32 – 7.21 (m, 8H), 7.21 – 7.14 (m, 4H), 6.89 – 6.83 (m, 2H), 4.87 (d, J = 4.0 Hz, 1H), 4.52 (s, 2H), 3.65 (d, J = 13.3 Hz, 1H), 3.53 (d, J = 13.2 Hz, 1H), 3.46 (d, J = 9.3 Hz, 1H), 2.90 – 2.84 (m, 1H), 2.76 (d, J = 9.3 Hz, 1H), 2.48 (s, 1H), 2.44 (dd, J = 9.3, 6.2 Hz, 1H);^{13}C NMR (126 MHz, DMSO) δ 178.45, 174.11, 144.85, 139.30, 138.59, 135.79, 132.20, 129.06, 128.81, 128.79, 128.74, 127.97, 127.67, 127.62, 127.52, 127.15, 126.46, 119.42, 109.58, 86.04, 77.91, 67.45, 61.15, 58.98, 57.84, 46.11, 42.92; HRMS(TOF MS ESI$^+$) caculated for $C_{34}H_{29}BrN_2O_4Na$ [M+Na]$^+$:631.1203, found 631.1209; HPLC conditions for determination enantiomeric excess: Chiral ODH, λ=254 nm, hexane:2-propanol=75:25, flow rate=1.0 mL/min, t_{major}=8.436.

5. Isotope tracer experiment

(1) Deuterium tracer experiment

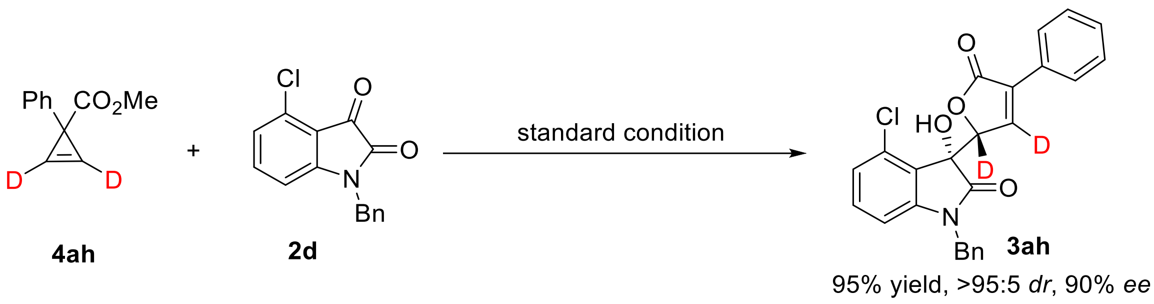

Scheme S5. Deuterium tracer experiment of the reaction involving cyclopropane 4ah and isatin 2d.

We synthesized dideuterated cyclopropylene ester **4ah**, with a deuteration rate of 97.5% (Figure 1). **4ah** was reacted with 4-chloroisatin **2d** under standard conditions, and **3ah** was successfully obtained with high yield and stereoselectivity. It can be seen from the comparison diagram of the separated pure product **3ah** and **3d** (Figure 2). The two deuterium atoms are retained in the product **3ah** (Scheme 5), which shows that the hydrogen in the 3-hydroxyl group of the product may come from water in the reaction solvent.

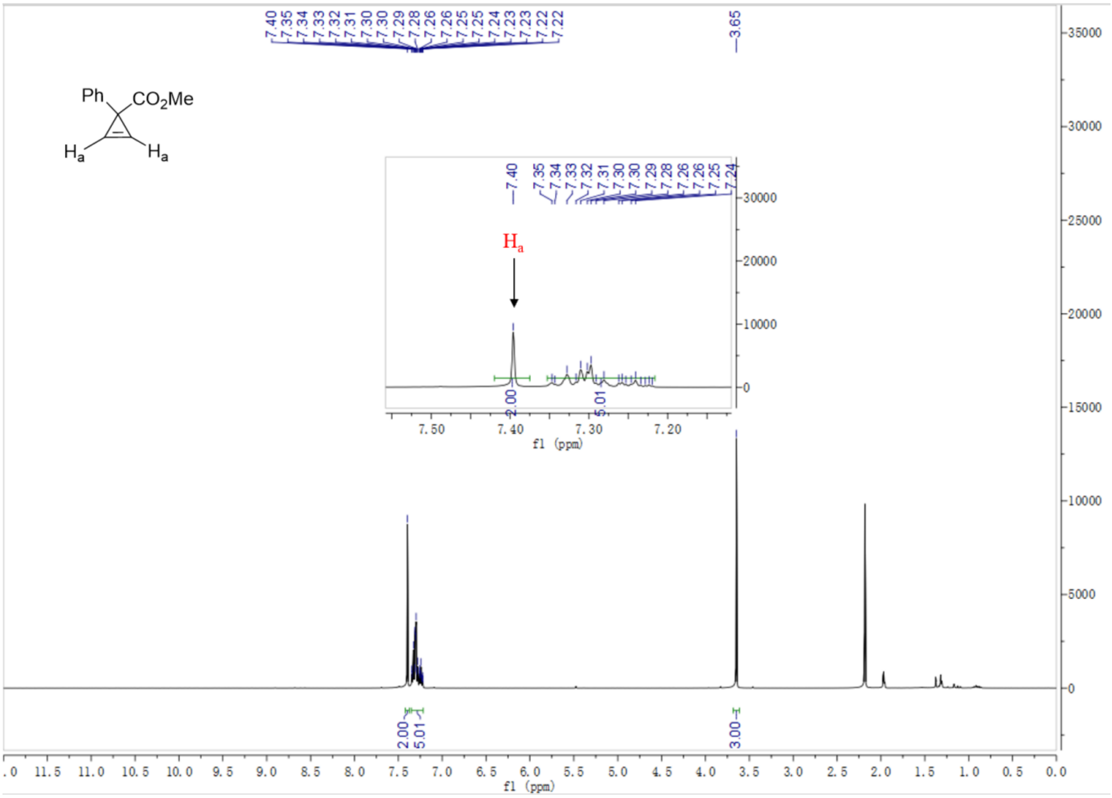

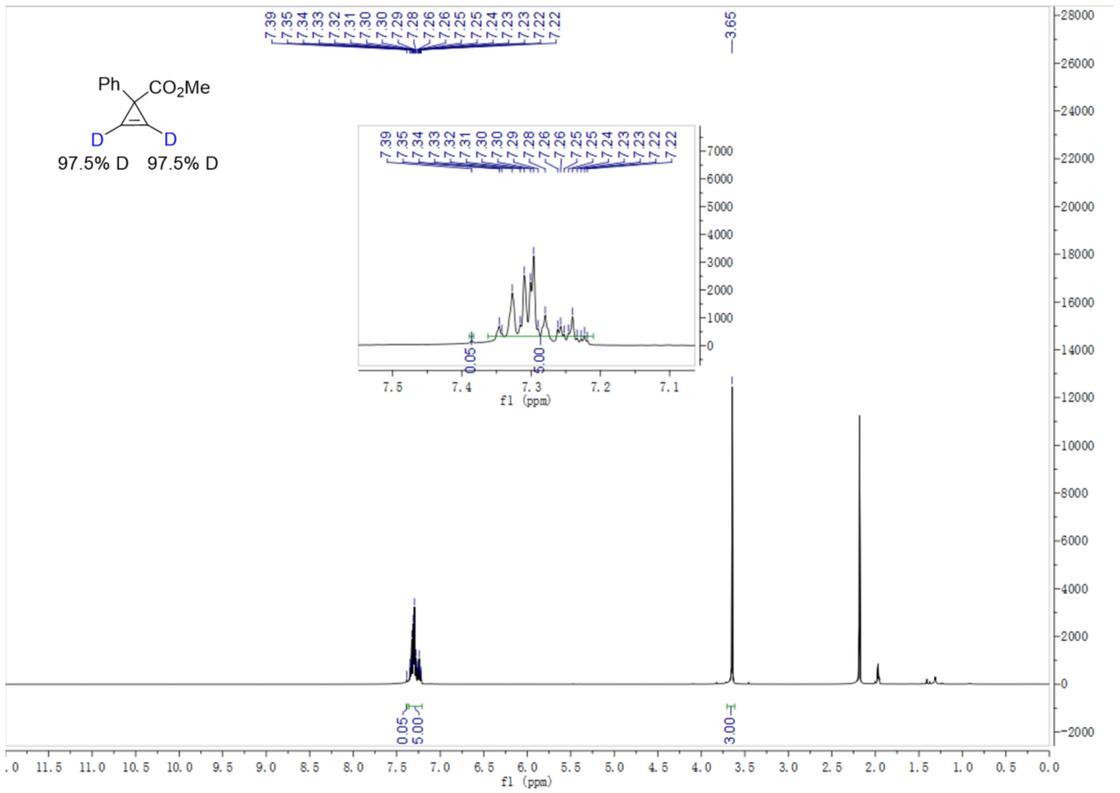

Figure S1 ^{1}H NMR in CD_3CN

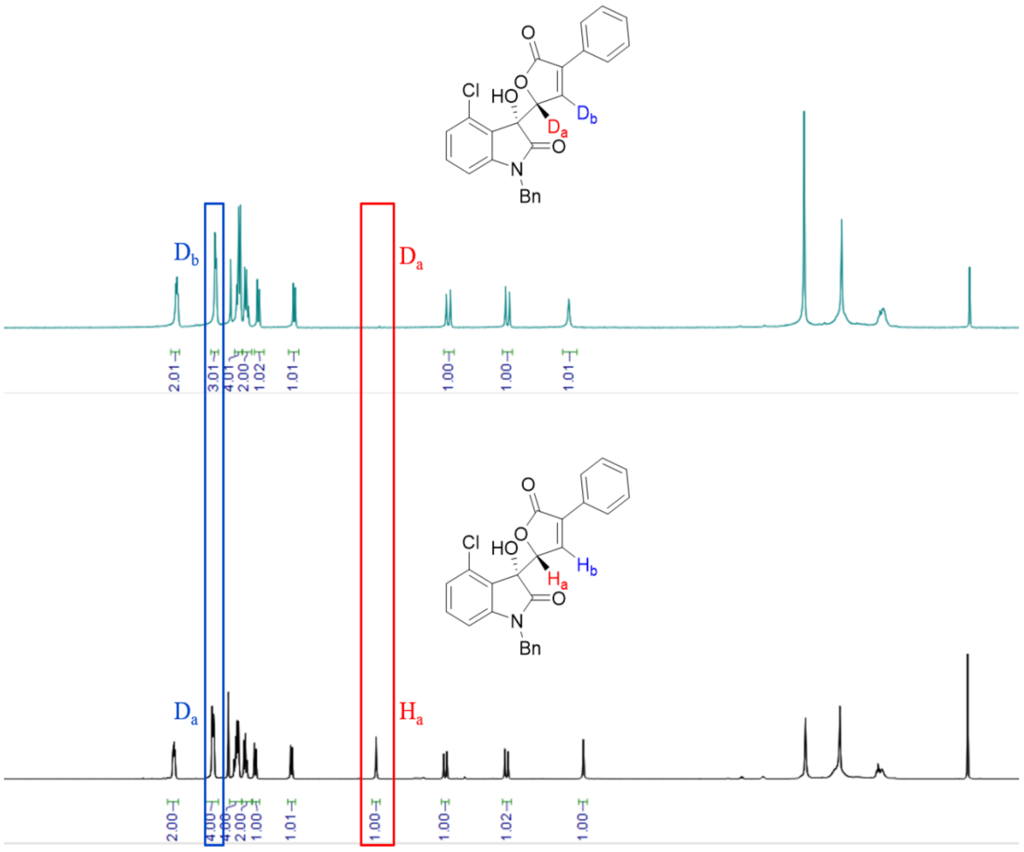

Figure S2 1H NMR comparison of 6ah and 6d

(2) O^{18} tracer experiment

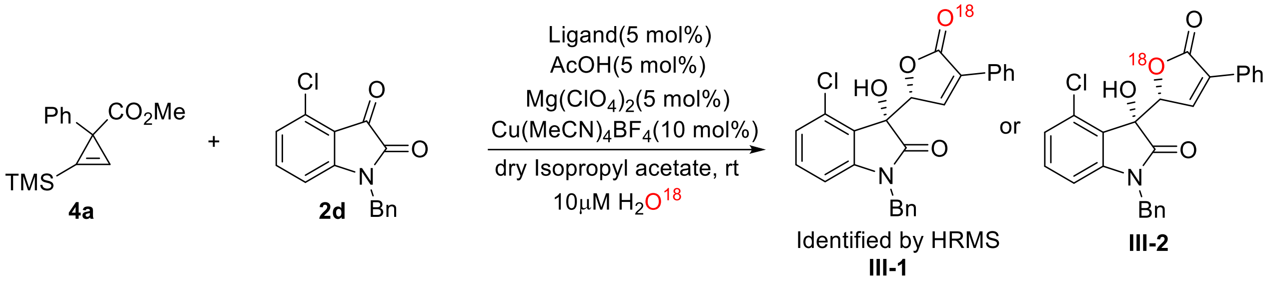

Scheme S6. ^{18}O tracer experiment of the reaction involving cyclopropen 4a and isatin 2d.

Isatin (**2d**, 0.1 mmol, 1eq), $Mg(ClO_4)_2$ (5 mol%), **L10** (5 mol%) were dissolved in 1 mL isopropyl acetate. After 30 minutes, cycloallyl ester (**4a,** 0.15 mmol, 1.5eq) was dissolved in 1 mL anhydrous isopropyl acetate and added to the ligand-activated isatin solution. Then $Cu(MeCN)_4BF_4$ (10 mol%) and AcOH (5 mol%) were added (Scheme 6). After the reaction was completed, the reaction liquid was concentrated to obtain the crude product, and the crude product was purified by column chromatography to obtain the pure product **III-1** or **III-2** (petroleum ether: ethyl acetate = 2:1). After HRMS confirmation, the structure of the product may be **III-1**(Figure **3**).

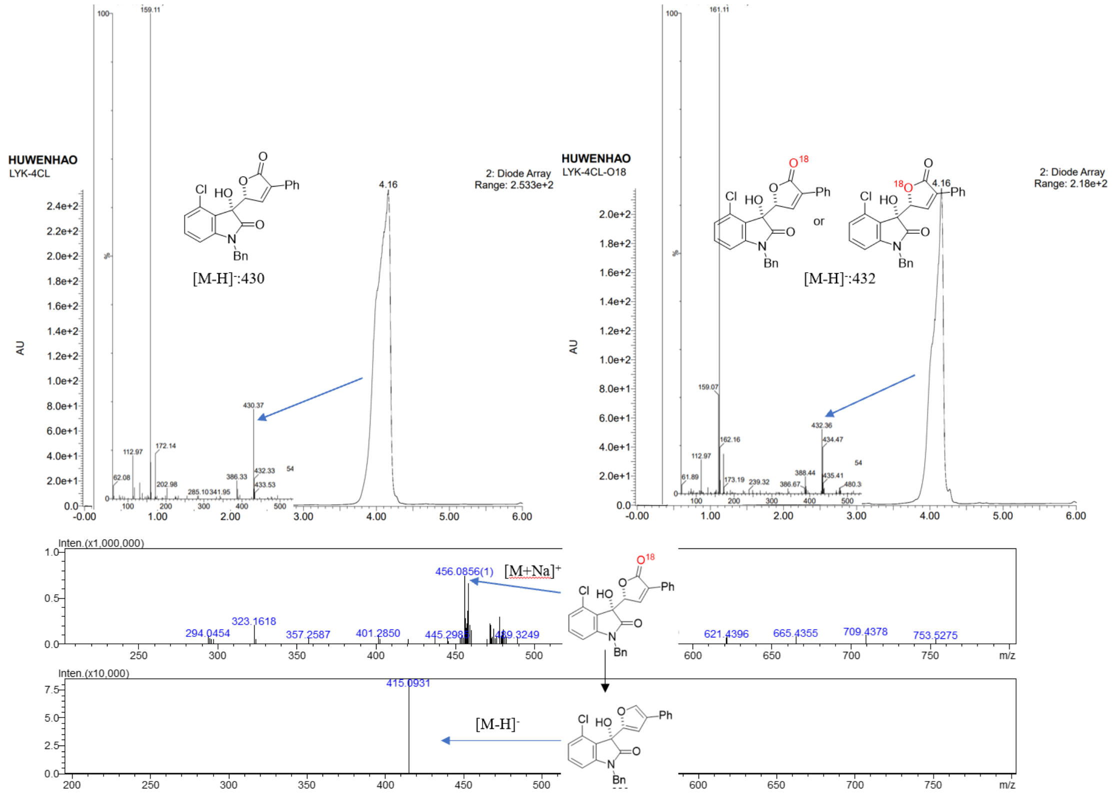

Figure S3 LC-MS and HRMS diagrams

6. Nonlinear effect experiment

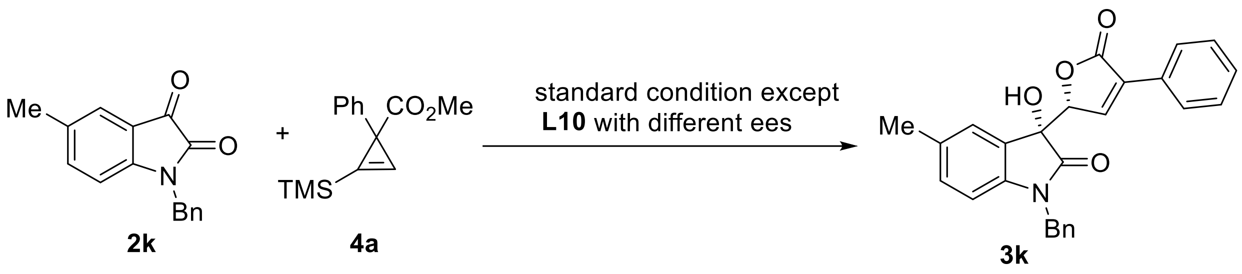

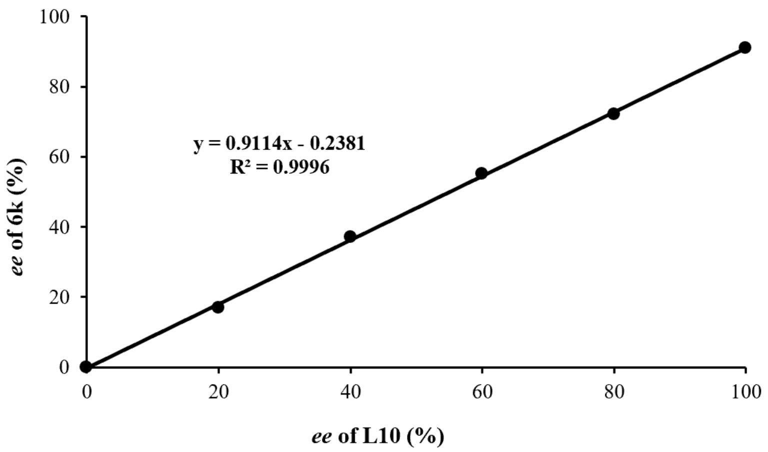

Figure S4. Nonlinear effect curve

We selected isatin **2k** and cycloallyl ester **4a** as raw materials, which were consistent with standard conditions except for different ligand ee. The corresponding ee values of product **3k** under different ligand ee were tested to explore the relationship between ligand ee values and product ee values. The result is shown in the figure 4.

7. Single Crystal X-ray Diffraction Data

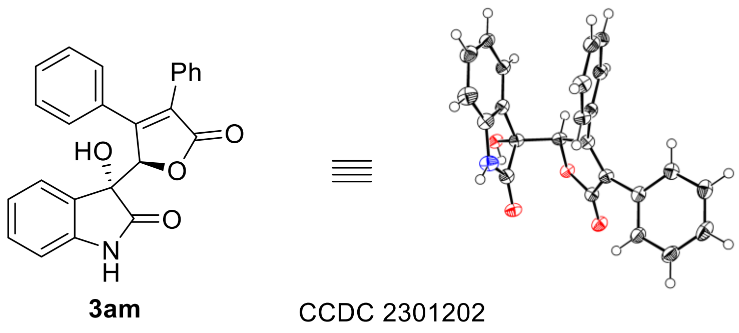

Figure S5. X-ray crystal structure of compound 3am.

```
Bond precision:   C-C = 0.0052 A          Wavelength=1.54184

Cell:             a=11.0612(2)     b=7.8538(1)        c=11.4457(3)
                  alpha=90         beta=112.689(3)    gamma=90
Temperature:      100 K
```

	Calculated	Reported
Volume	917.37(4)	917.37(4)
Space group	P 21	P 1 21 1
Hall group	P 2yb	P 2yb
Moiety formula	C24 H17 N O4	C24 H17 N O4
Sum formula	C24 H17 N O4	C24 H17 N O4
Mr	383.39	383.38
Dx,g cm-3	1.388	1.388
Z	2	2
Mu (mm-1)	0.776	0.776
F000	400.0	400.0
F000'	401.27	
h,k,lmax	14,9,14	13,9,14
Nref	3935[2114]	3766
Tmin,Tmax	0.955,0.962	0.703,1.000
Tmin'	0.925	

```
Correction method= # Reported T Limits: Tmin=0.703 Tmax=1.000
AbsCorr = MULTI-SCAN

Data completeness= 1.78/0.96       Theta(max)= 78.491

R(reflections)= 0.0515( 3600)                    wR2(reflections)=
                                                 0.1498( 3766)
S = 1.098                 Npar= 263
```

Table S3. Crystal data and structure refinement for liyk_231013_auto.

Identification code	liyk_231013_auto
Empirical formula	$C_{24}H_{17}NO_4$
Formula weight	383.38
Temperature/K	100.00(10)
Crystal system	monoclinic
Space group	$P2_1$
a/Å	11.0612(2)
b/Å	7.85380(10)
c/Å	11.4457(3)
α/°	90
β/°	112.689(3)
γ/°	90
Volume/Å^3	917.37(4)
Z	2

ρ_{calc}g/cm^3	1.388
μ/mm^{-1}	0.776
F(000)	400.0
Crystal size/mm^3	0.1 × 0.05 × 0.05
Radiation	Cu Kα (λ = 1.54184)
2Θ range for data collection/°	8.374 to 156.982
Index ranges	-13 ≤ h ≤ 13, -9 ≤ k ≤ 9, -14 ≤ l ≤ 14
Reflections collected	18471
Independent reflections	3766 [R_{int} = 0.0696, R_{sigma} = 0.0417]
Data/restraints/parameters	3766/1/263
Goodness-of-fit on F^2	1.098
Final R indexes [I>=2σ (I)]	R_1 = 0.0515, wR_2 = 0.1481
Final R indexes [all data]	R_1 = 0.0532, wR_2 = 0.1498
Largest diff. peak/hole / e Å^{-3}	0.43/-0.28
Flack parameter	-0.09(12)

Table S4. Fractional Atomic Coordinates (×104) and Equivalent Isotropic Displacement Parameters (Å2×103) for liyk_231013_auto. Ueq is defined as 1/3 of of the trace of the orthogonalised UIJ tensor.

Atom	x	y	z	U(eq)
O10	3979(2)	7982(3)	2690(2)	22.4(5)
O28	5008(3)	6349(3)	5157(2)	27.0(5)
O27	2044(2)	7194(3)	2703(2)	28.8(6)
O29	6518(2)	8931(3)	4254(2)	24.1(5)
N9	6883(3)	4951(4)	5280(3)	29.5(7)
C3	7464(3)	6396(4)	3844(3)	22.1(6)
C14	3010(3)	6817(4)	2521(3)	22.8(7)
C1	5934(3)	6145(4)	4841(3)	24.2(7)
C11	5074(3)	7156(4)	2543(3)	22.8(7)
C4	8221(3)	6716(4)	3152(3)	24.3(7)
C26	5845(3)	4608(5)	758(3)	25.2(7)
C21	5391(3)	4177(4)	1706(3)	23.0(7)
C22	5781(3)	2644(4)	2348(3)	24.7(7)

C25	6679(3)	3519(5)	472(3)	26.4(7)
C12	4580(3)	5393(4)	2068(3)	22.1(6)
C8	7803(3)	5077(5)	4719(3)	26.6(7)
C15	2455(3)	3737(4)	1685(3)	24.3(7)
C2	6235(3)	7233(4)	3843(3)	22.3(6)
C16	1747(3)	3281(5)	2412(4)	28.5(8)
C24	7099(3)	2008(5)	1146(4)	29.2(8)
C19	1341(4)	1557(5)	167(4)	30.9(8)
C20	2252(3)	2871(5)	563(3)	26.6(7)
C23	6648(4)	1581(5)	2080(4)	29.7(7)
C13	3375(3)	5197(4)	2080(3)	22.8(7)
C5	9324(3)	5714(5)	3359(3)	27.3(7)
C18	656(4)	1080(5)	899(4)	34.8(8)
C6	9662(4)	4429(5)	4261(4)	31.8(8)
C17	856(4)	1937(6)	2024(4)	35.3(9)
C7	8905(4)	4088(5)	4956(4)	35.3(9)

Table S5. Anisotropic Displacement Parameters (Å2×103) for liyk_231013_auto. The Anisotropic displacement factor exponent takes the form: -2π2[h2a*2U11+2hka*b*U12+…].

Atom	U11	U22	U33	U23	U13	U12
O10	22.3(11)	15.1(11)	30.9(12)	-1.6(9)	11.6(9)	0.9(9)
O28	27.9(11)	23.4(13)	34.4(13)	0.3(10)	17.3(10)	-1.1(9)
O27	24.4(11)	23.5(12)	40.1(14)	-4.4(11)	14.3(10)	1.5(10)
O29	23.3(11)	16.2(11)	33.8(12)	-2.0(10)	12.0(9)	-1.2(9)
N9	32.8(16)	24.0(16)	35.6(16)	10.7(12)	17.4(13)	5.9(12)
C3	22.0(15)	15.0(15)	28.0(15)	-1.7(13)	8.1(12)	-0.8(12)
C14	23.0(15)	18.4(16)	26.5(15)	-0.7(12)	9.2(12)	2.2(12)
C1	26.6(16)	18.4(16)	27.8(16)	-0.5(13)	10.6(13)	-2.4(12)
C11	24.6(15)	17.4(15)	29.9(16)	-0.8(13)	14.4(13)	0.2(13)

C4	23.8(15)	20.3(16)	28.6(16)	-1.7(13)	9.9(13)	-2.1(12)
C26	25.3(16)	20.8(16)	28.9(16)	-2.7(13)	9.9(13)	-1.3(13)
C21	21.2(15)	19.0(16)	27.8(16)	-4.5(13)	8.2(12)	-2.0(12)
C22	27.8(16)	17.3(16)	33.2(18)	-1.6(13)	16.2(14)	0.0(12)
C25	25.8(16)	25.9(17)	30.0(17)	-5.9(14)	13.5(13)	-4.9(14)
C12	24.9(15)	15.3(15)	26.1(15)	1.4(12)	9.7(12)	0.4(12)
C8	26.9(17)	20.7(17)	31.9(17)	3.0(14)	11.0(14)	1.9(13)
C15	22.0(15)	18.2(16)	30.4(16)	-0.9(13)	7.7(12)	1.9(13)
C2	25.4(15)	14.2(14)	29.7(16)	-1.0(12)	13.4(13)	-1.9(12)
C16	23.7(16)	28.6(19)	33.4(18)	-3.9(14)	11.4(14)	-3.3(13)
C24	24.0(16)	24.9(18)	39.4(18)	-11.0(15)	12.9(14)	-0.3(14)
C19	27.0(16)	24.4(17)	36.4(19)	-6.7(15)	6.7(14)	2.4(14)
C20	26.8(16)	21.9(17)	29.8(17)	-1.6(14)	9.3(13)	2.3(14)
C23	31.7(17)	16.8(16)	42.1(19)	0.1(14)	15.7(15)	3.6(13)
C13	23.8(16)	18.0(15)	27.9(16)	-0.4(13)	11.3(13)	3.0(12)
C5	22.4(15)	27.4(18)	33.2(17)	-5.6(15)	12.1(13)	-3.4(14)
C18	24.3(17)	26.6(18)	51(2)	-7.0(17)	11.6(15)	-6.2(14)
C6	25.0(17)	30.9(19)	38.8(19)	-1.0(16)	11.4(15)	7.8(14)
C17	29.1(17)	35(2)	44(2)	-3.3(17)	17.0(16)	-10.0(16)
C7	34(2)	29(2)	43(2)	8.1(16)	15.9(16)	10.1(15)

Table S6. Bond Lengths for liyk_231013_auto.

Atom	Atom	Length/Å	Atom	Atom	Length/Å
O10	C14	1.365(4)	C21	C22	1.390(5)
O10	C11	1.440(4)	C21	C12	1.475(5)
O28	C1	1.222(4)	C22	C23	1.392(5)
O27	C14	1.202(4)	C25	C24	1.394(6)
O29	C2	1.408(4)	C12	C13	1.347(5)
N9	C1	1.351(5)	C8	C7	1.382(5)

N9	C8	1.400(5)	C15	C16	1.392(5)
C3	C4	1.379(5)	C15	C20	1.393(5)
C3	C8	1.388(5)	C15	C13	1.484(5)
C3	C2	1.510(4)	C16	C17	1.394(5)
C14	C13	1.481(5)	C24	C23	1.385(5)
C1	C2	1.562(5)	C19	C20	1.390(5)
C11	C12	1.511(5)	C19	C18	1.381(6)
C11	C2	1.546(5)	C5	C6	1.388(6)
C4	C5	1.393(5)	C18	C17	1.393(6)
C26	C21	1.401(5)	C6	C7	1.386(5)
C26	C25	1.387(5)			

Table S7. Bond Angles for liyk_231013_auto.

Atom	Atom	Atom	Angle/˚	Atom	Atom	Atom	Angle/˚
C14	O10	C11	109.3(2)	C7	C8	N9	127.8(3)
C1	N9	C8	112.2(3)	C7	C8	C3	122.2(3)
C4	C3	C8	119.8(3)	C16	C15	C20	119.9(3)
C4	C3	C2	131.7(3)	C16	C15	C13	119.6(3)
C8	C3	C2	108.5(3)	C20	C15	C13	120.4(3)
O10	C14	C13	108.8(3)	O29	C2	C3	109.1(3)
O27	C14	O10	120.8(3)	O29	C2	C1	110.9(3)
O27	C14	C13	130.4(3)	O29	C2	C11	110.7(3)
O28	C1	N9	126.9(3)	C3	C2	C1	101.9(3)
O28	C1	C2	125.7(3)	C3	C2	C11	113.4(3)
N9	C1	C2	107.3(3)	C11	C2	C1	110.5(3)
O10	C11	C12	104.6(2)	C15	C16	C17	119.7(3)
O10	C11	C2	107.1(3)	C23	C24	C25	119.4(3)
C12	C11	C2	115.7(3)	C18	C19	C20	120.1(4)
C3	C4	C5	119.0(3)	C19	C20	C15	120.1(3)

C25	C26	C21	120.3(3)	C24	C23	C22	120.6(3)
C26	C21	C12	120.0(3)	C14	C13	C15	121.5(3)
C22	C21	C26	119.2(3)	C12	C13	C14	107.8(3)
C22	C21	C12	120.7(3)	C12	C13	C15	130.6(3)
C21	C22	C23	120.2(3)	C6	C5	C4	120.3(3)
C26	C25	C24	120.3(3)	C19	C18	C17	120.1(4)
C21	C12	C11	121.3(3)	C7	C6	C5	121.2(3)
C13	C12	C11	108.8(3)	C18	C17	C16	120.1(4)
C13	C12	C21	129.8(3)	C8	C7	C6	117.5(4)
C3	C8	N9	110.0(3)				

Table S8. Torsion Angles for liyk_231013_auto.

A	B	C	D	Angle/˚	A	B	C	D	Angle/˚
O10	C14	C13	C12	3.6(4)	C21	C22	C23	C24	-2.0(5)
O10	C14	C13	C15	-173.6(3)	C21	C12	C13	C14	-179.9(3)
O10	C11	C12	C21	175.4(3)	C21	C12	C13	C15	-3.0(6)
O10	C11	C12	C13	-6.0(4)	C22	C21	C12	C11	117.8(4)
O10	C11	C2	O29	-57.5(3)	C22	C21	C12	C13	-60.5(5)
O10	C11	C2	C3	179.5(3)	C25	C26	C21	C22	-0.3(5)
O10	C11	C2	C1	65.8(3)	C25	C26	C21	C12	175.7(3)
O28	C1	C2	O29	64.4(4)	C25	C24	C23	C22	0.0(5)
O28	C1	C2	C3	-179.5(3)	C12	C11	C2	O29	-173.5(3)
O28	C1	C2	C11	-58.7(4)	C12	C11	C2	C3	63.4(4)
O27	C14	C13	C12	-178.0(4)	C12	C11	C2	C1	-50.3(4)
O27	C14	C13	C15	4.8(6)	C12	C21	C22	C23	-173.8(3)

N9	C1	C2	O29	-115.6(3)	C8	N9	C1	O28	-179.8(3)
N9	C1	C2	C3	0.4(3)	C8	N9	C1	C2	0.3(4)
N9	C1	C2	C11	121.2(3)	C8	C3	C4	C5	-0.7(5)
N9	C8	C7	C6	178.6(4)	C8	C3	C2	O29	116.4(3)
C3	C4	C5	C6	-0.8(5)	C8	C3	C2	C1	-1.0(3)
C3	C8	C7	C6	-1.5(6)	C8	C3	C2	C11	-119.7(3)
C14	O10	C11	C12	8.3(3)	C15	C16	C17	C18	-1.4(6)
C14	O10	C11	C2	-115.0(3)	C2	C3	C4	C5	-179.9(3)
C1	N9	C8	C3	-0.9(4)	C2	C3	C8	N9	1.2(4)
C1	N9	C8	C7	178.9(4)	C2	C3	C8	C7	-178.7(3)
C11	O10	C14	O27	173.8(3)	C2	C11	C12	C21	-67.2(4)
C11	O10	C14	C13	-7.6(3)	C2	C11	C12	C13	111.5(3)
C11	C12	C13	C14	1.7(4)	C16	C15	C20	C19	0.3(5)
C11	C12	C13	C15	178.5(3)	C16	C15	C13	C14	-44.1(5)
C4	C3	C8	N9	-178.2(3)	C16	C15	C13	C12	139.4(4)
C4	C3	C8	C7	2.0(5)	C19	C18	C17	C16	0.2(6)
C4	C3	C2	O29	-64.4(4)	C20	C15	C16	C17	1.2(5)
C4	C3	C2	C1	178.3(4)	C20	C15	C13	C14	133.0(3)
C4	C3	C2	C11	59.6(5)	C20	C15	C13	C12	-43.5(5)
C4	C5	C6	C7	1.2(6)	C20	C19	C18	C17	1.3(6)
C26	C21	C22	C23	2.1(5)	C13	C15	C16	C17	178.3(3)
C26	C21	C12	C11	-58.2(5)	C13	C15	C20	C19	-176.8(3)
C26	C21	C12	C13	123.5(4)	C5	C6	C7	C8	-0.1(6)

C26	C25	C24	C23	1.8(5)	C18	C19	C20	C15	-1.5(6)
C21	C26	C25	C24	-1.7(5)					

Table S9. Hydrogen Atom Coordinates (Å×104) and Isotropic Displacement Parameters (Å2×103) for liyk_231013_auto.

Atom	x	y	z	U(eq)
H29	5860.8	9368.83	4333.42	36
H9	6918.35	4182.44	5851.45	35
H11	5308.09	7772.92	1895.32	27
H4	7993.35	7606.76	2542.98	29
H26	5580.49	5650.3	310.47	30
H22	5455.47	2321.43	2972.34	30
H25	6965.19	3804.59	-186.91	32
H16	1869.48	3882.7	3169.17	34
H24	7689.82	1278.04	965.91	35
H19	1189.74	986.89	-609.17	37
H20	2737.95	3177.64	67.79	32
H23	6931.99	552.82	2542.19	36
H5	9847.02	5911.41	2881.23	33
H18	47.47	166.18	634.67	42
H6	10426.54	3770.3	4403.73	38
H17	385.77	1604.86	2527.12	42
H7	9135.59	3207.81	5572.66	42

8. ^{1}H NMR and ^{13}C NMR spectrums.

^{1}H-NMR and ^{13}C-NMR for **3a:**

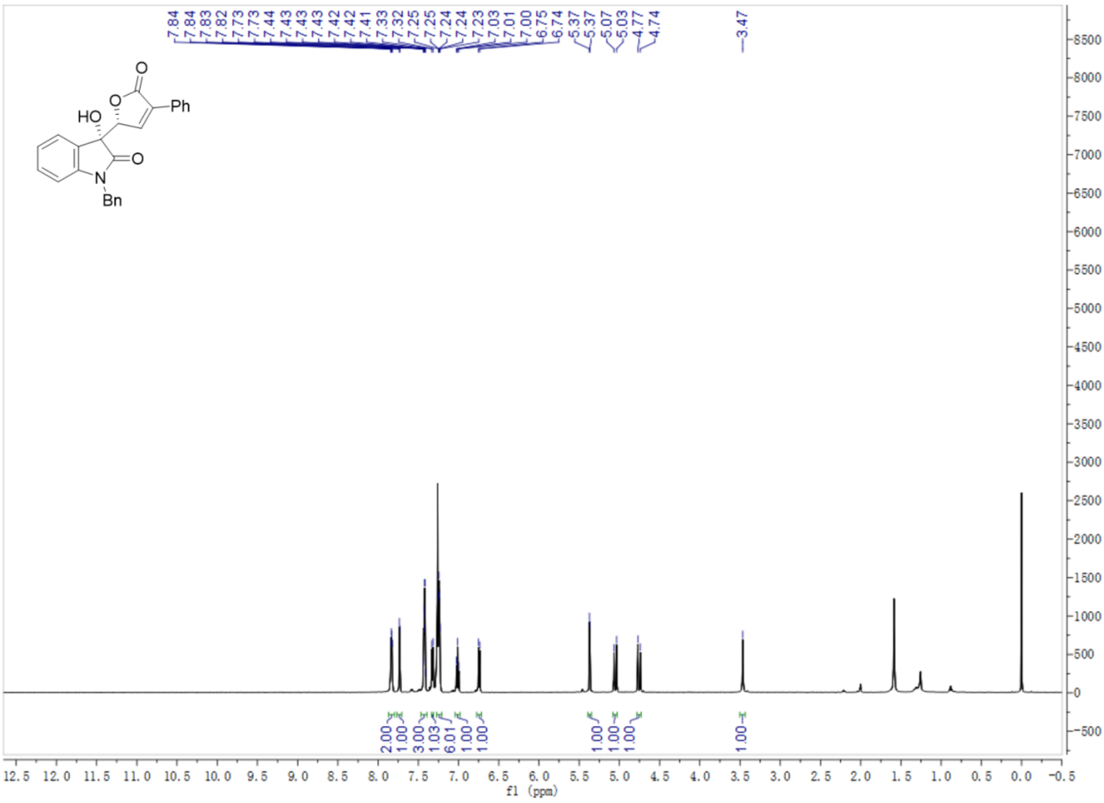

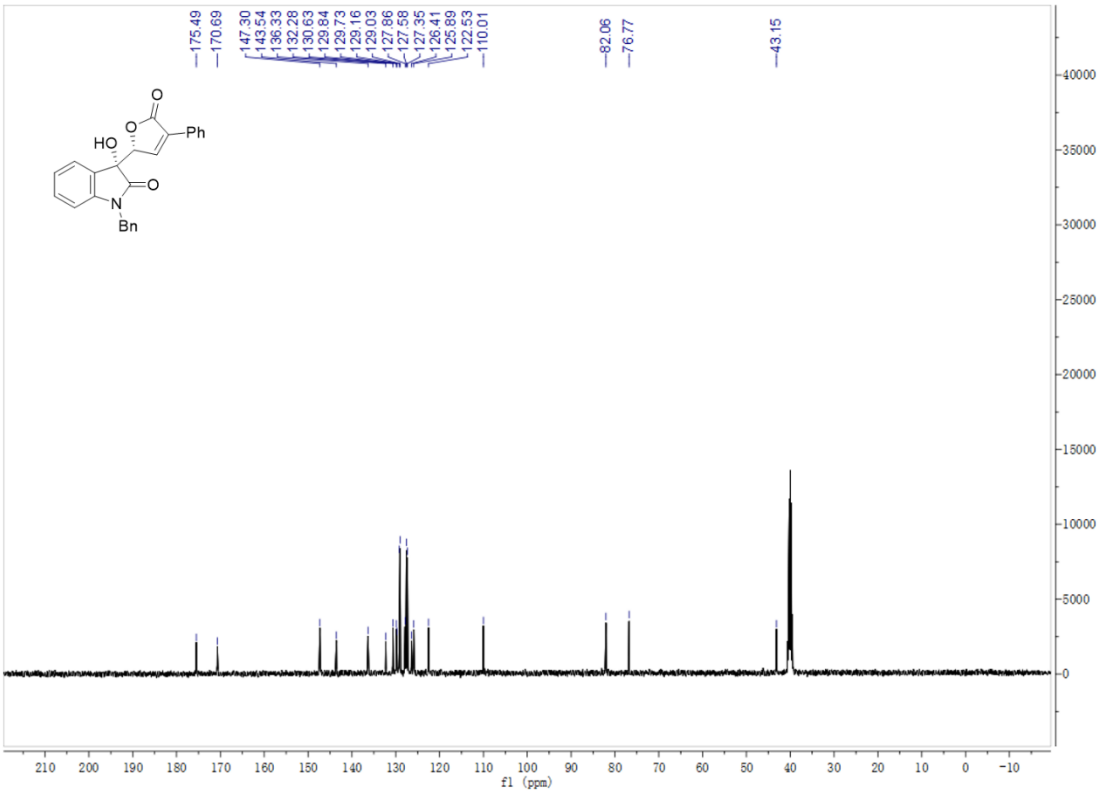

^{1}H-NMR and ^{13}C-NMR for **3b:**

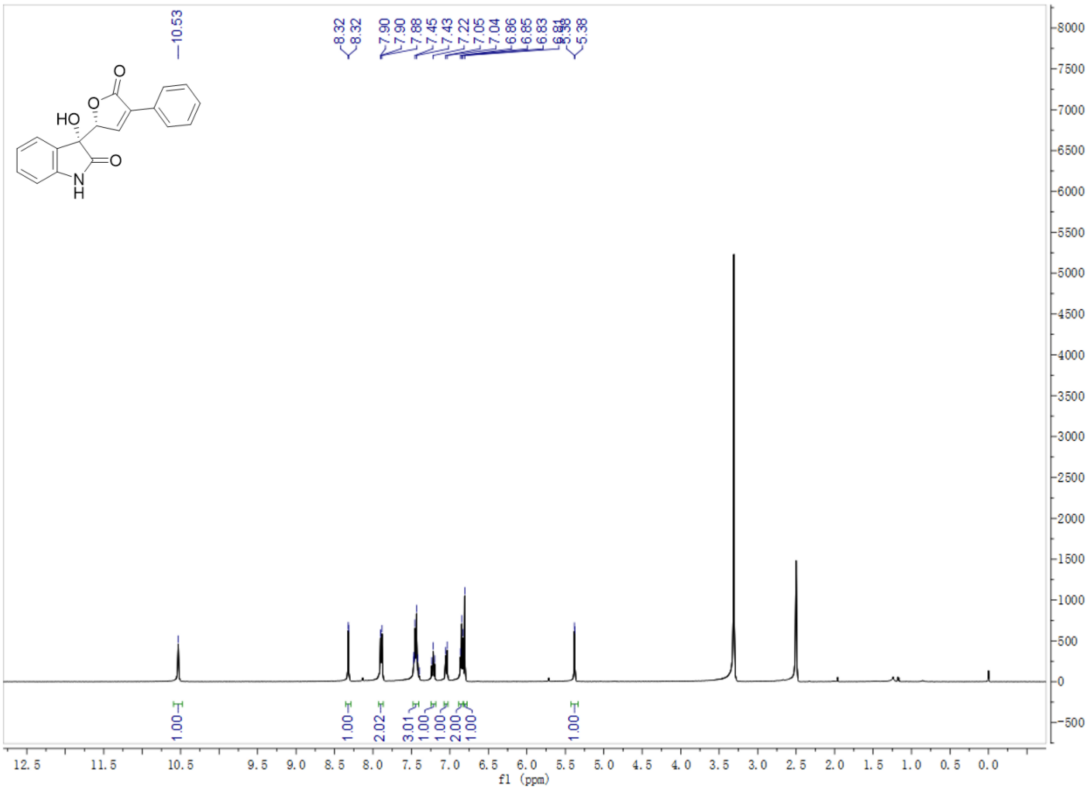

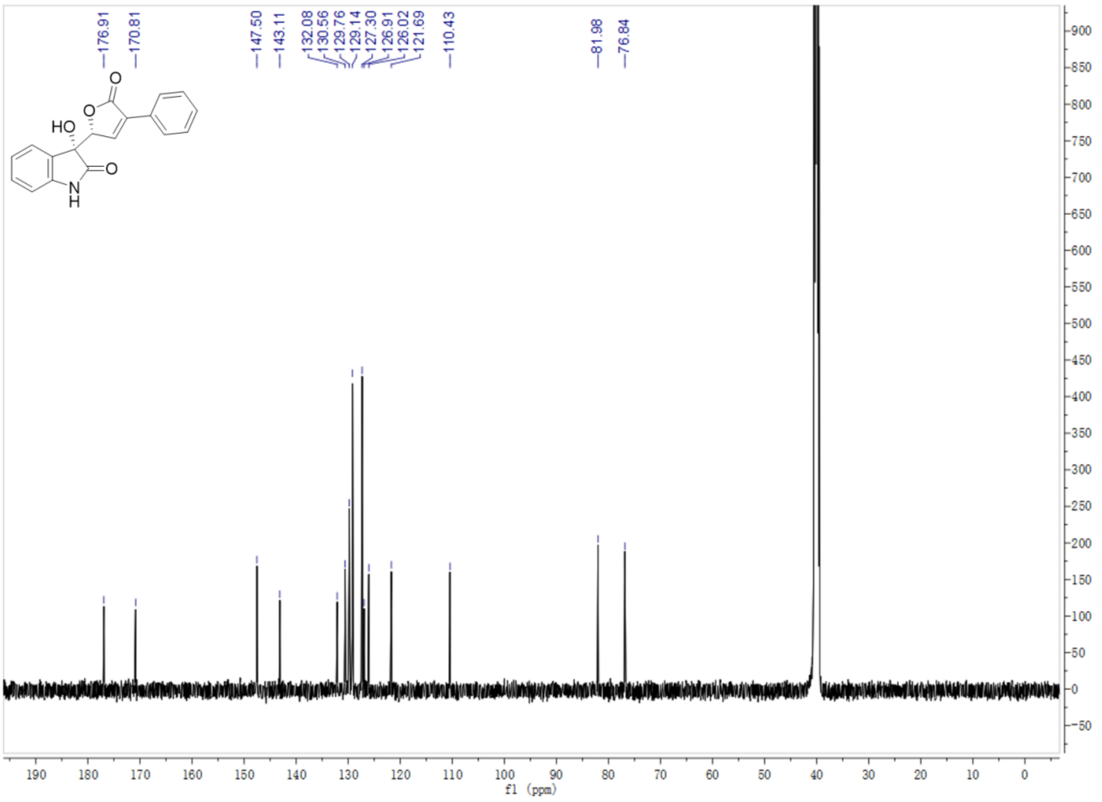

^{1}H-NMR and ^{13}C-NMR for **3c:**

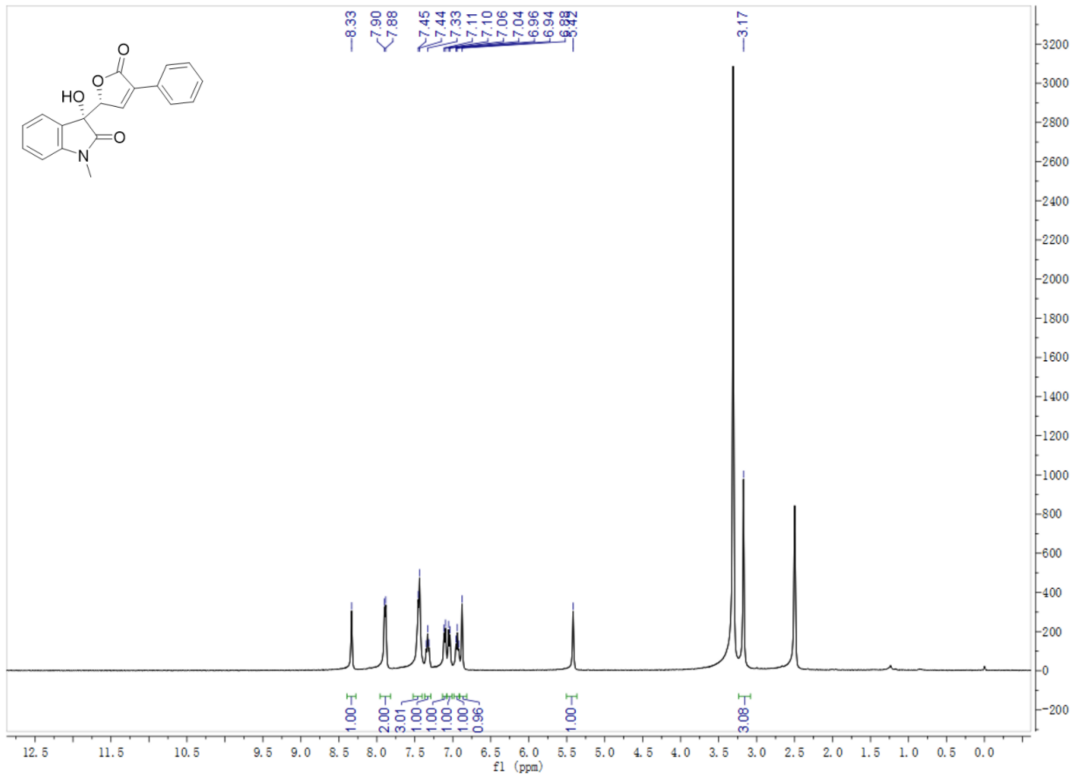

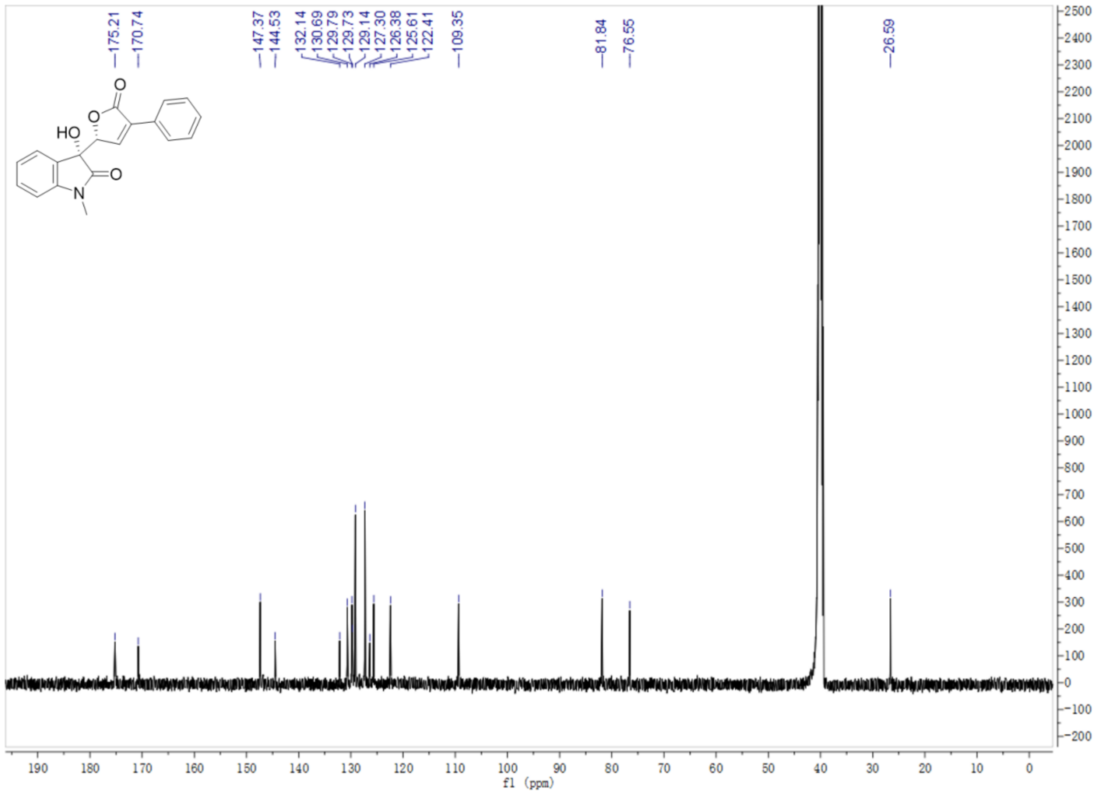

^{1}H-NMR and ^{13}C-NMR for **3d:**

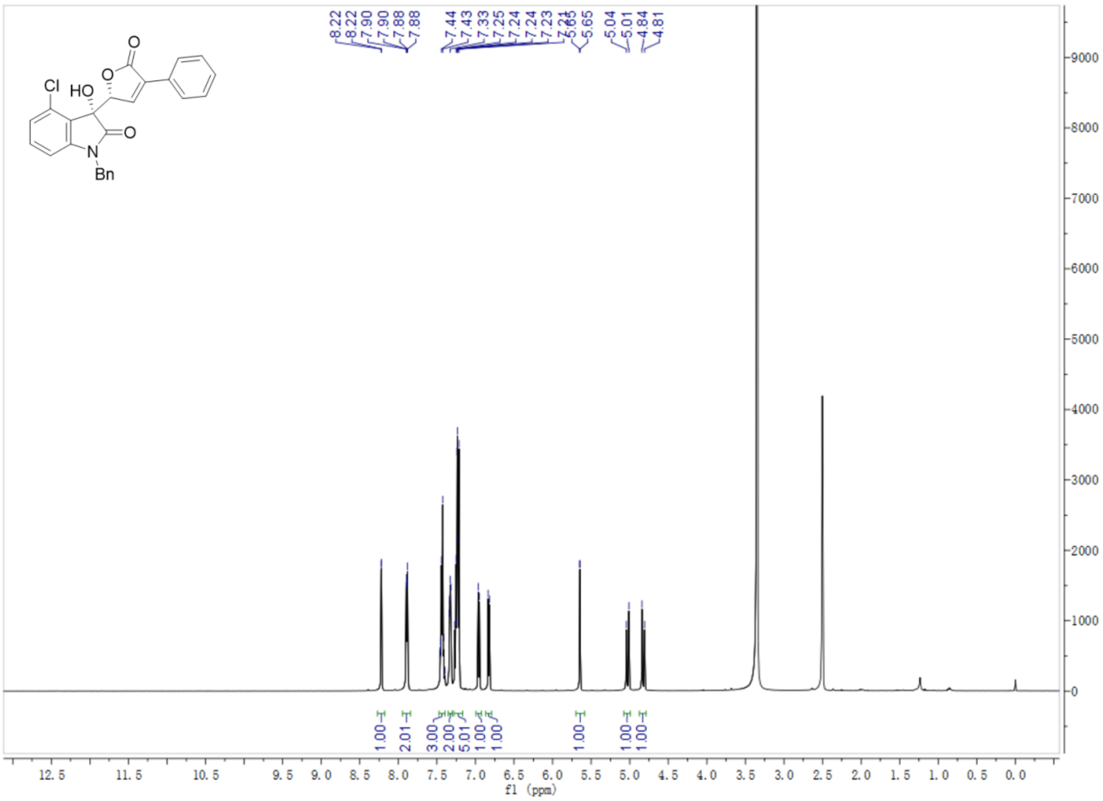

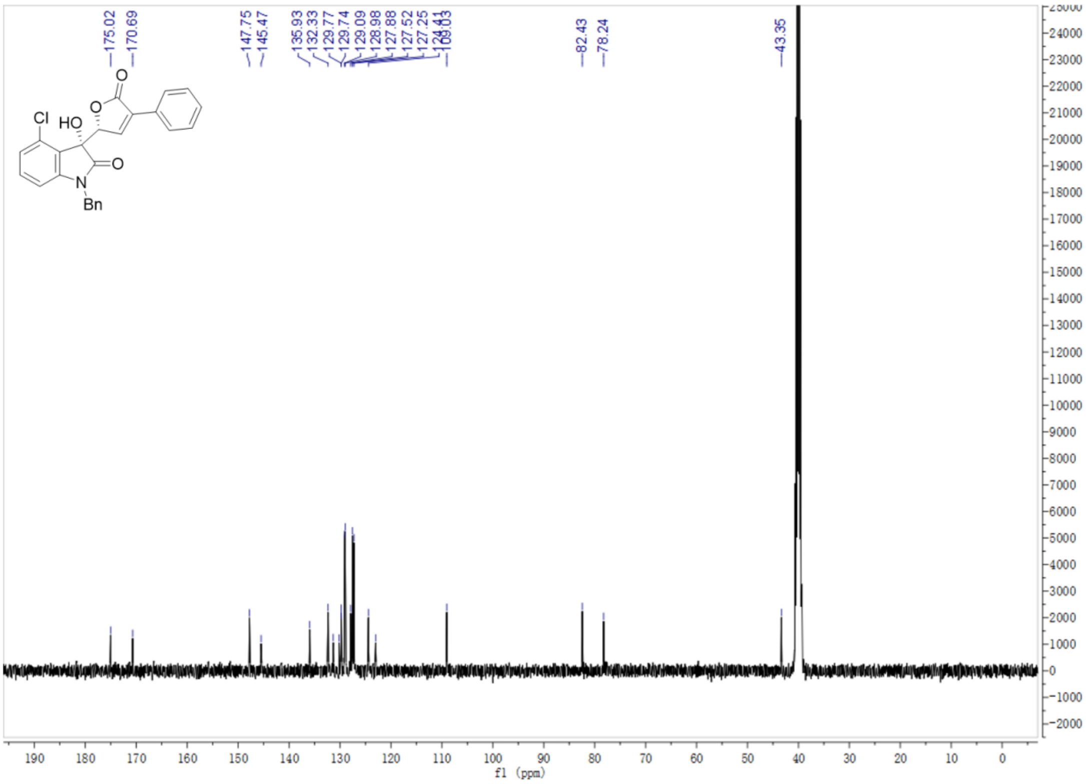

^{1}H-NMR and ^{13}C-NMR for **3e:**

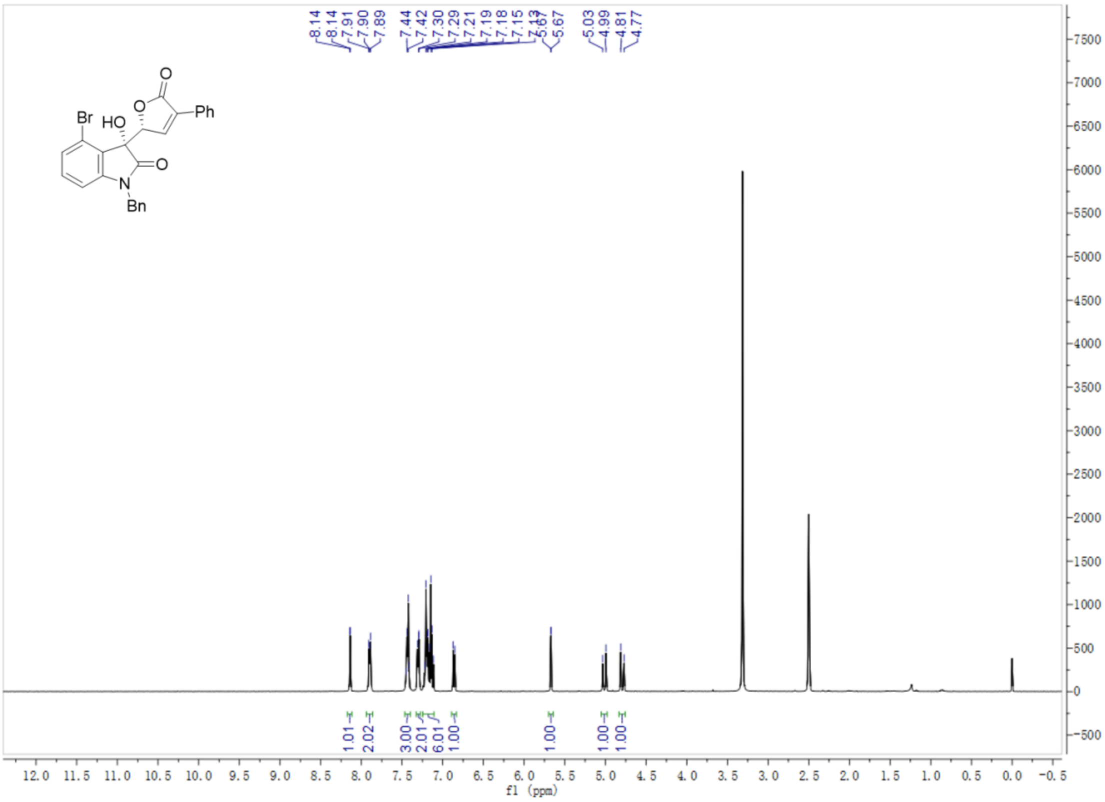

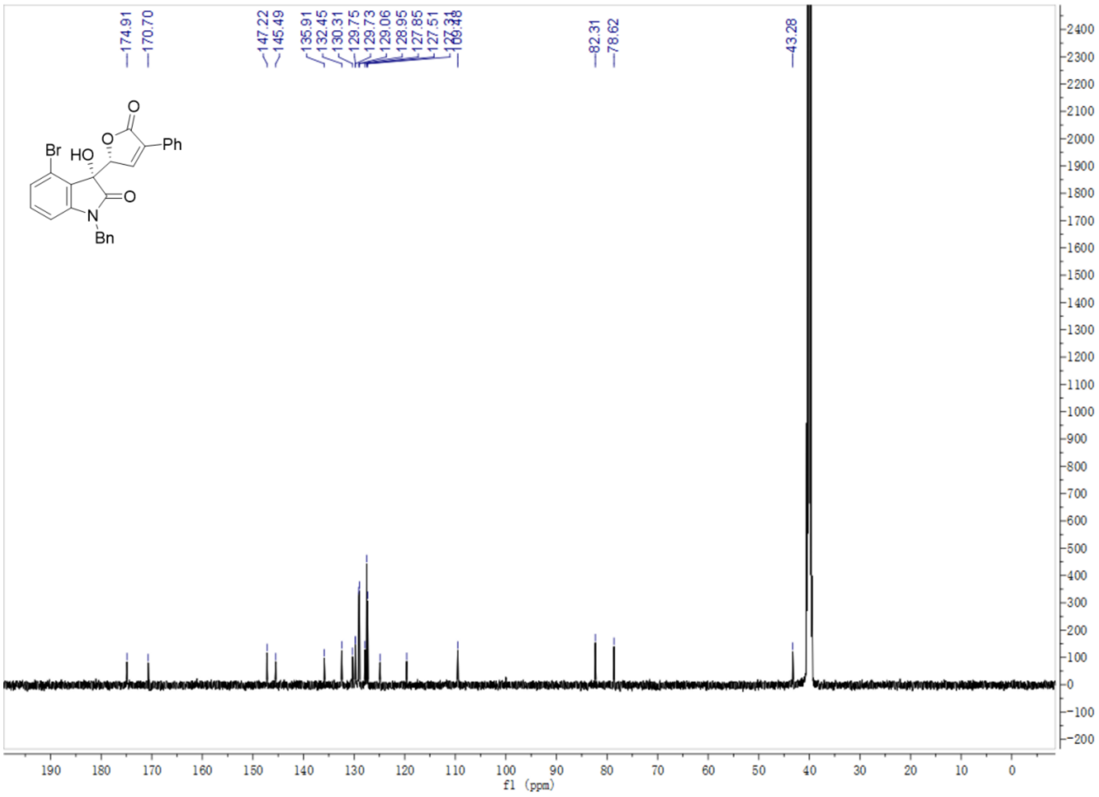

^{1}H-NMR and ^{13}C-NMR for **3f:**

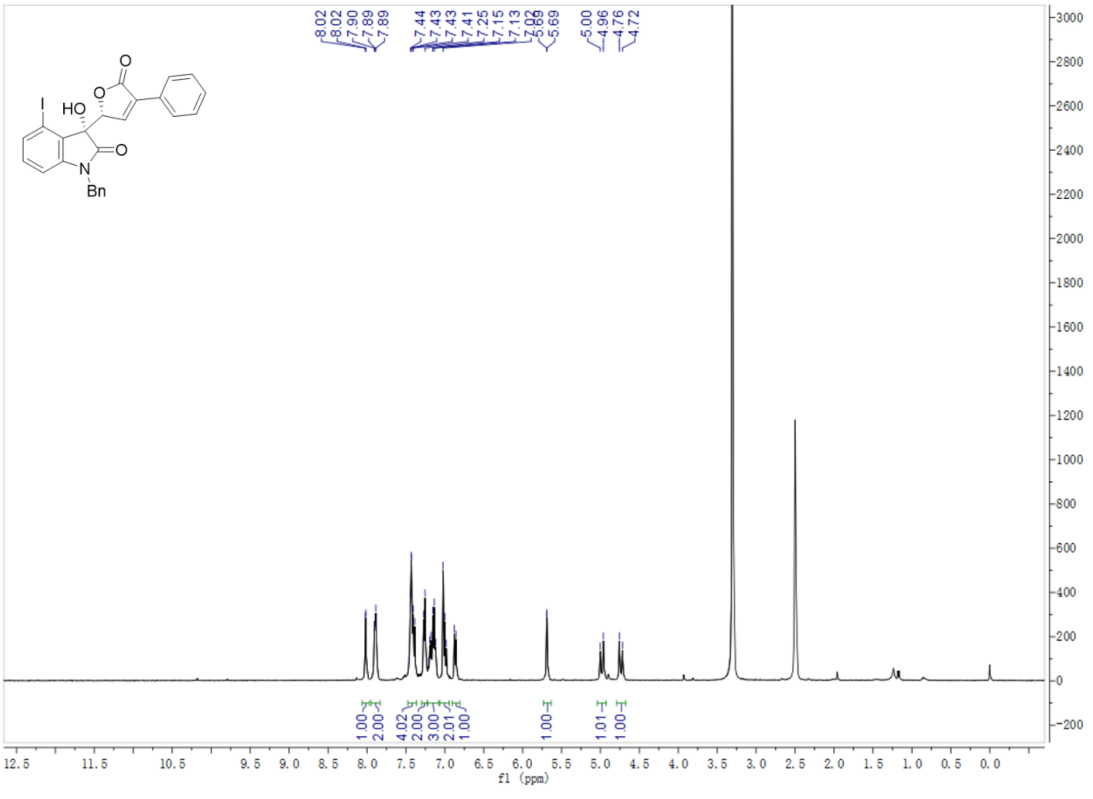

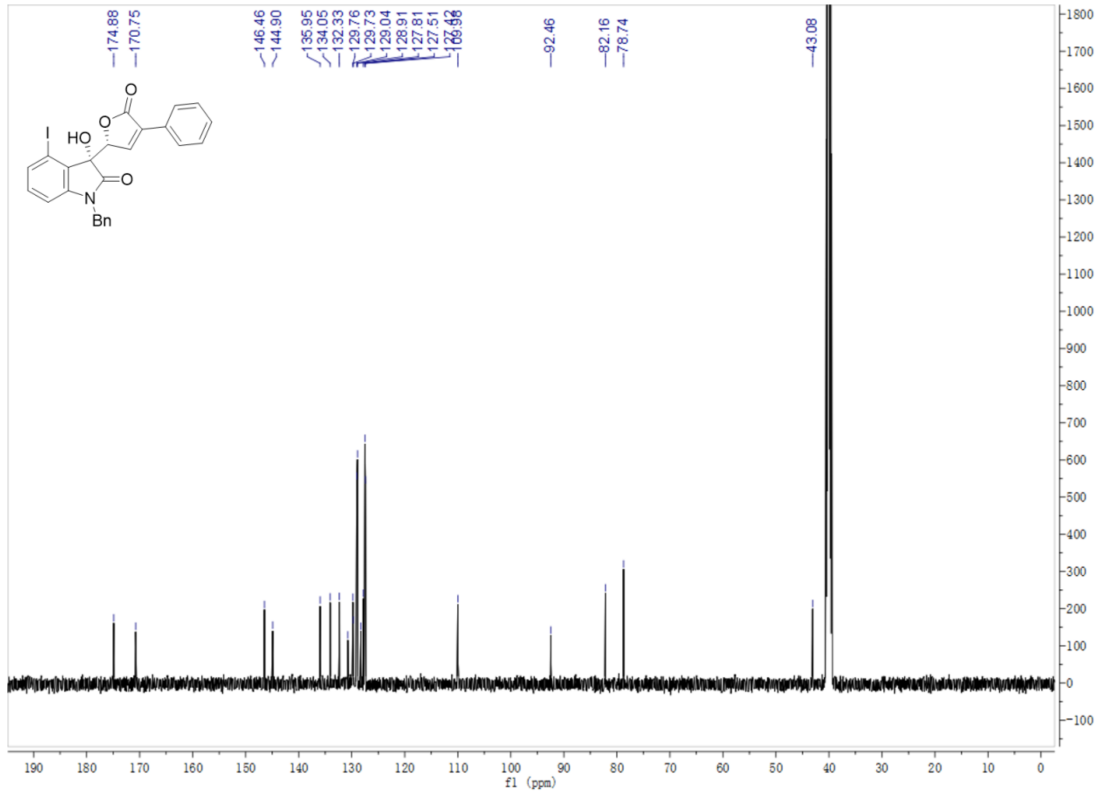

^{1}H-NMR and ^{13}C-NMR for **3g:**

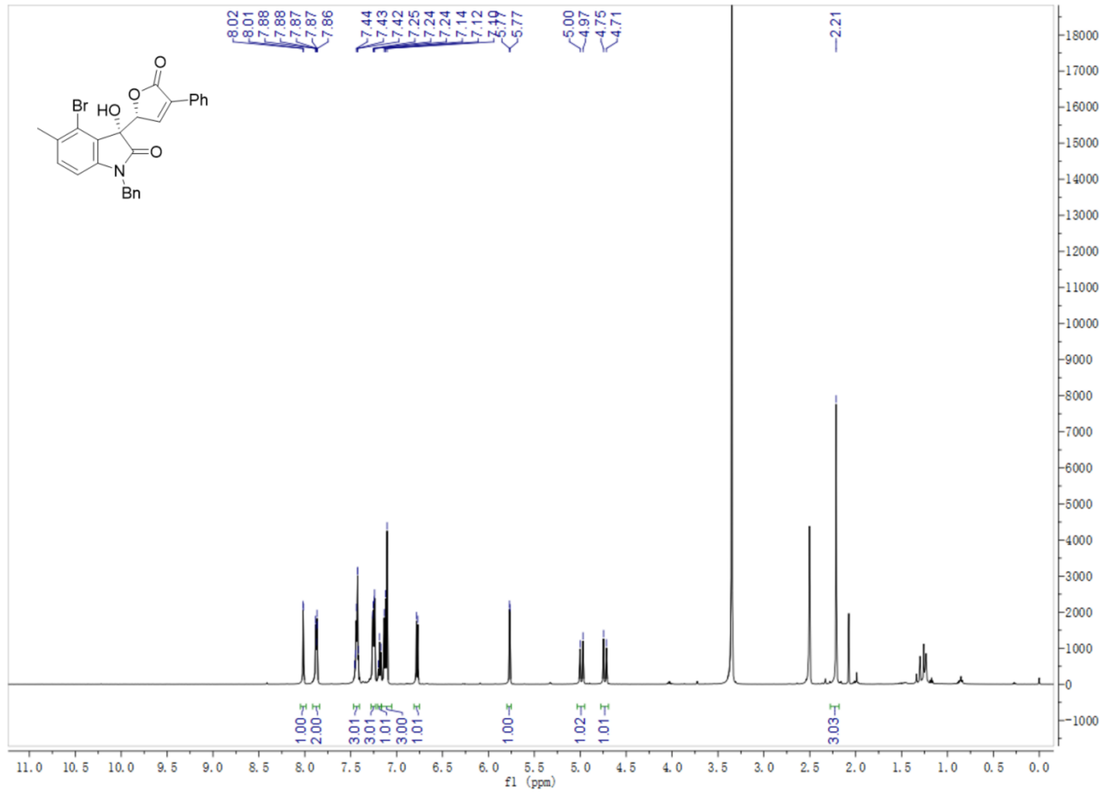

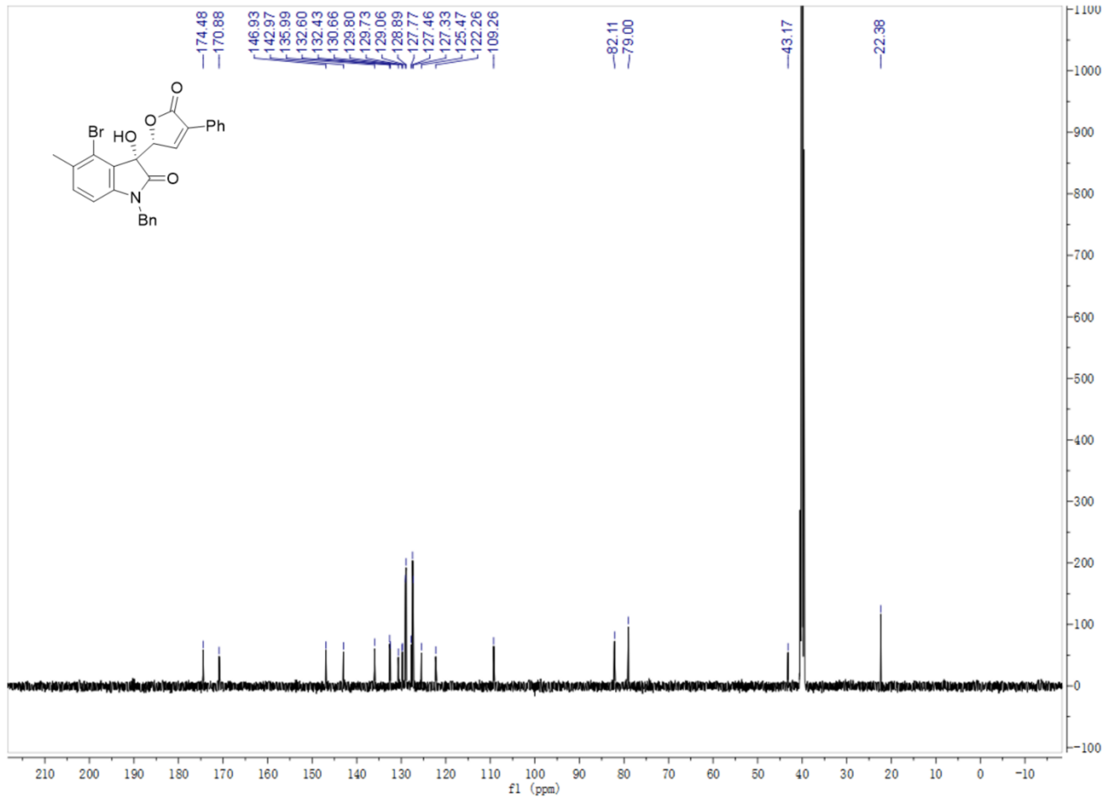

^{1}H-NMR and ^{13}C-NMR for **3h:**

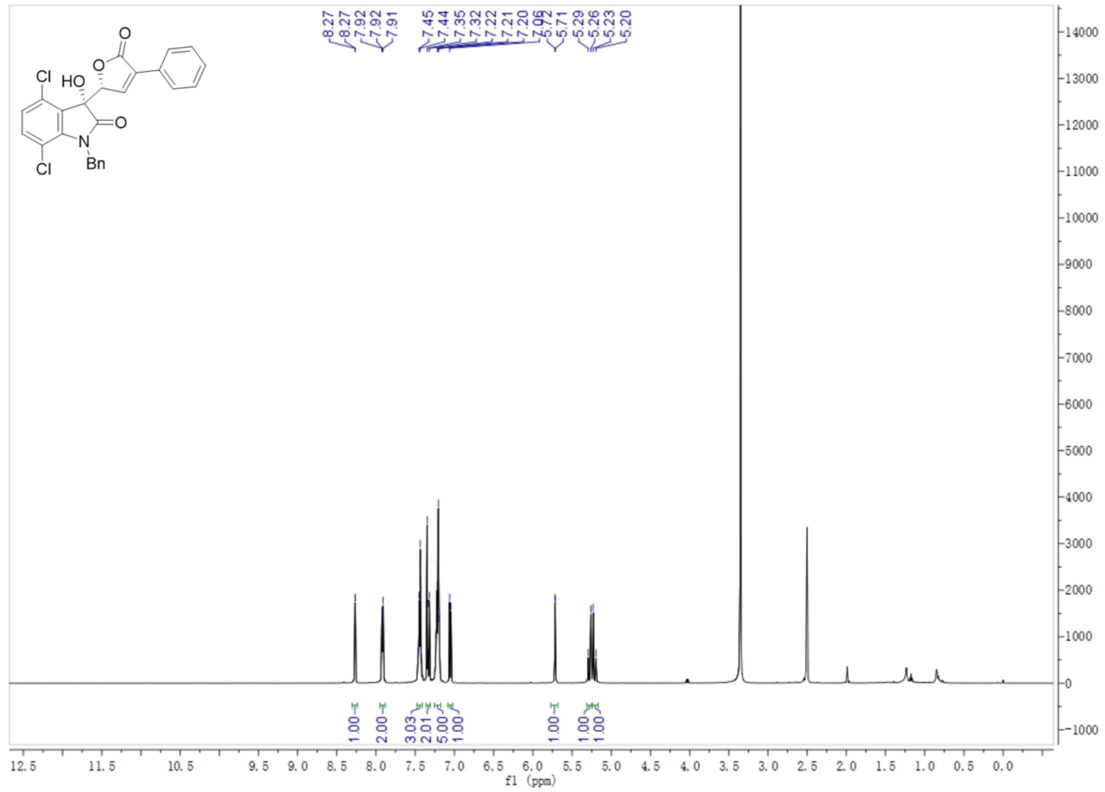

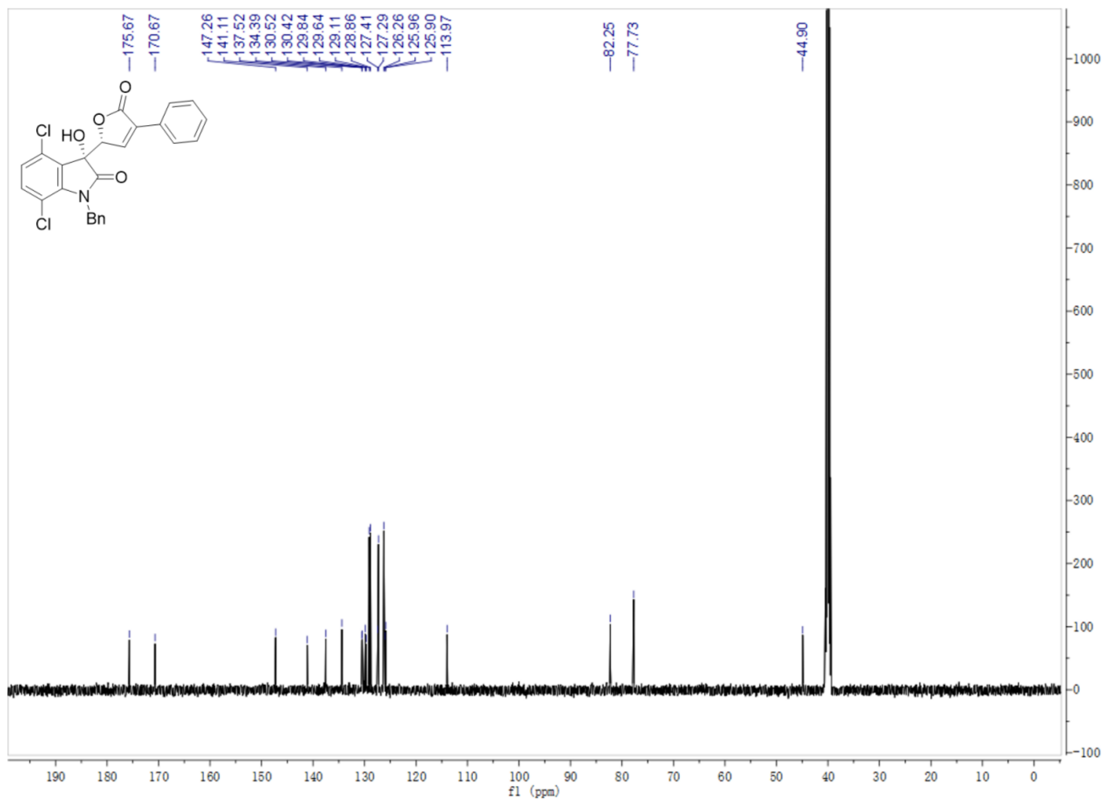

^{1}H-NMR and ^{13}C-NMR for **3i:**

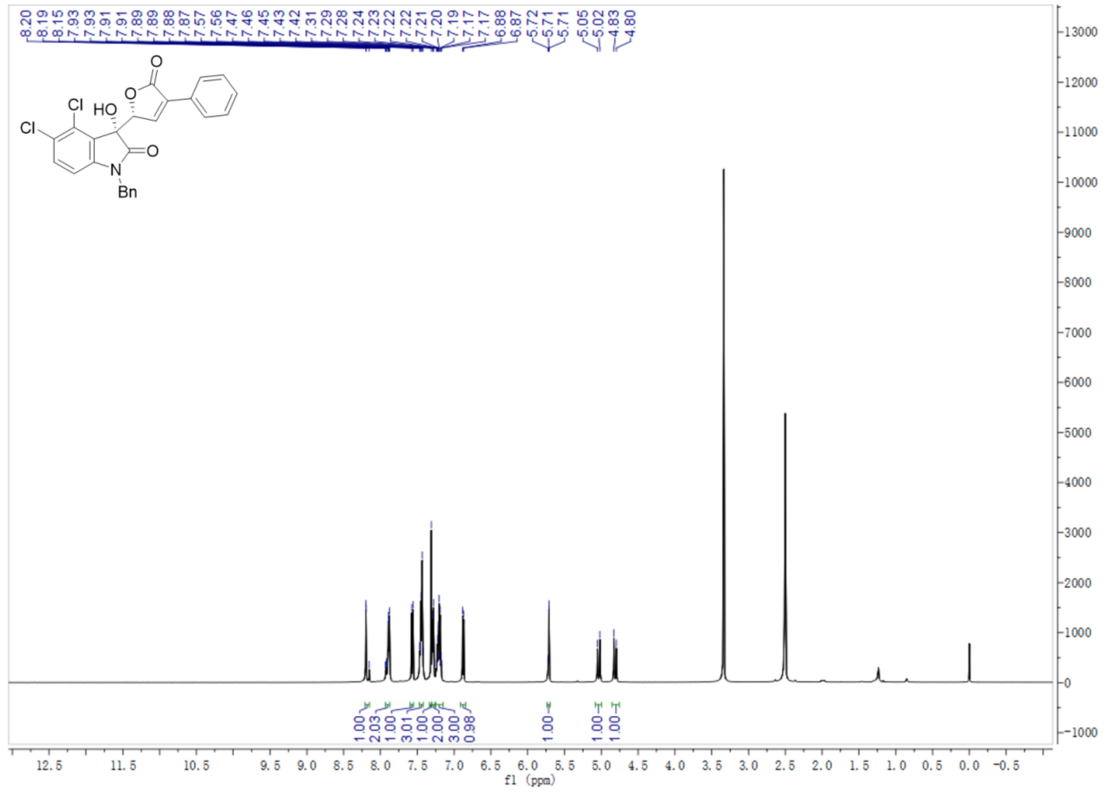

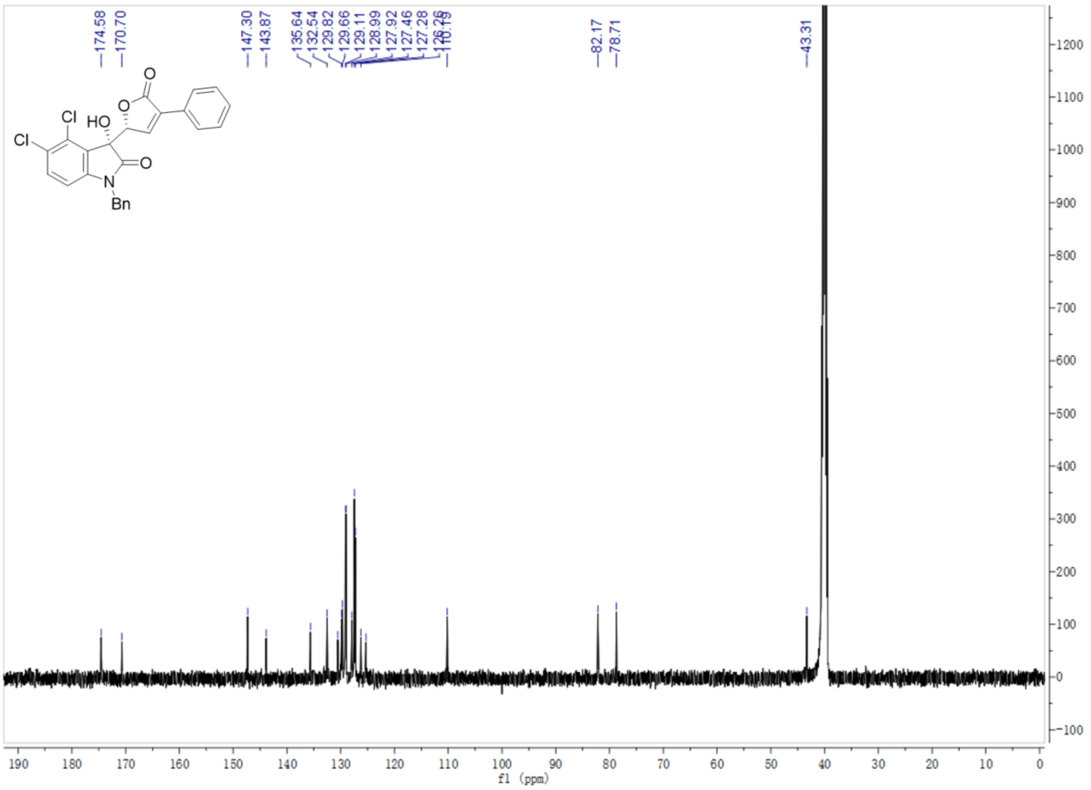

^{1}H-NMR and ^{13}C-NMR for **3j:**

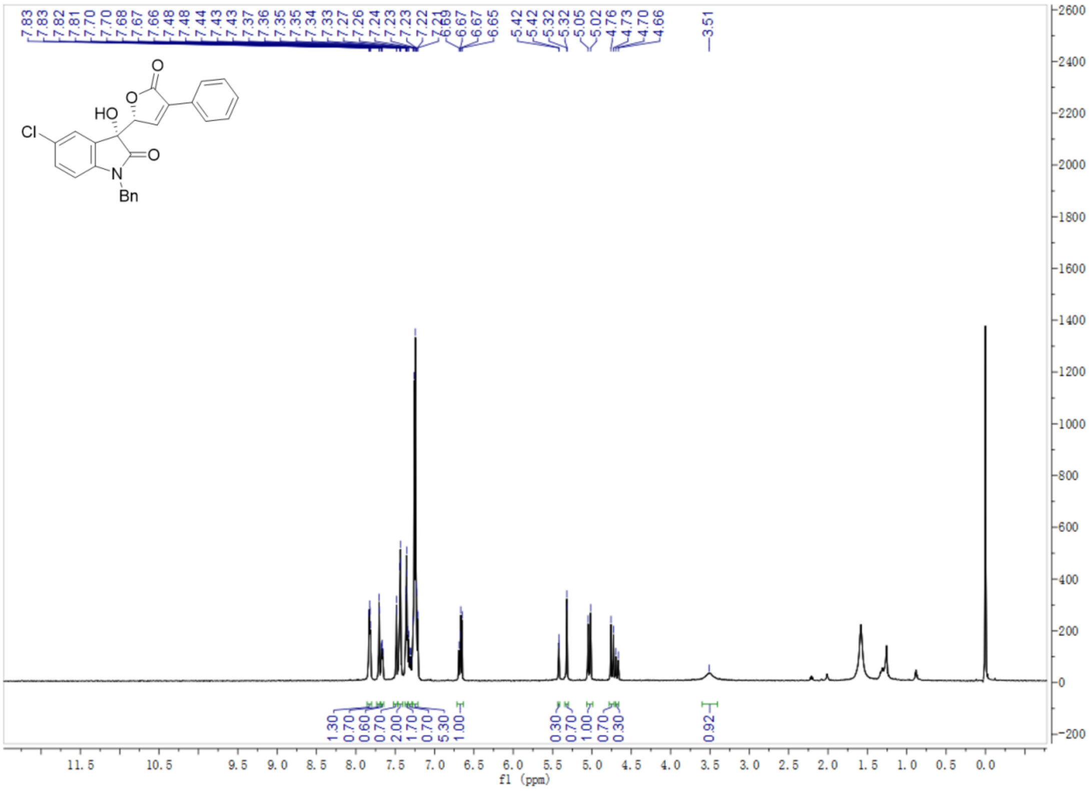

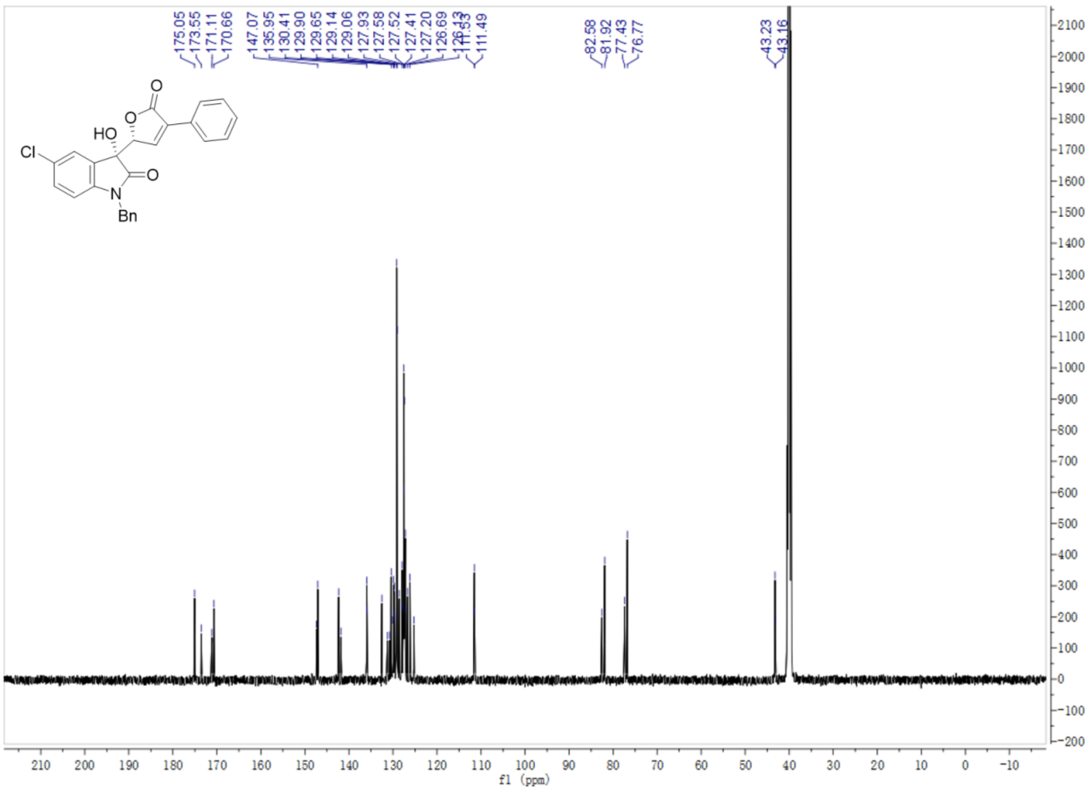

^{1}H-NMR and ^{13}C-NMR for **3k:**

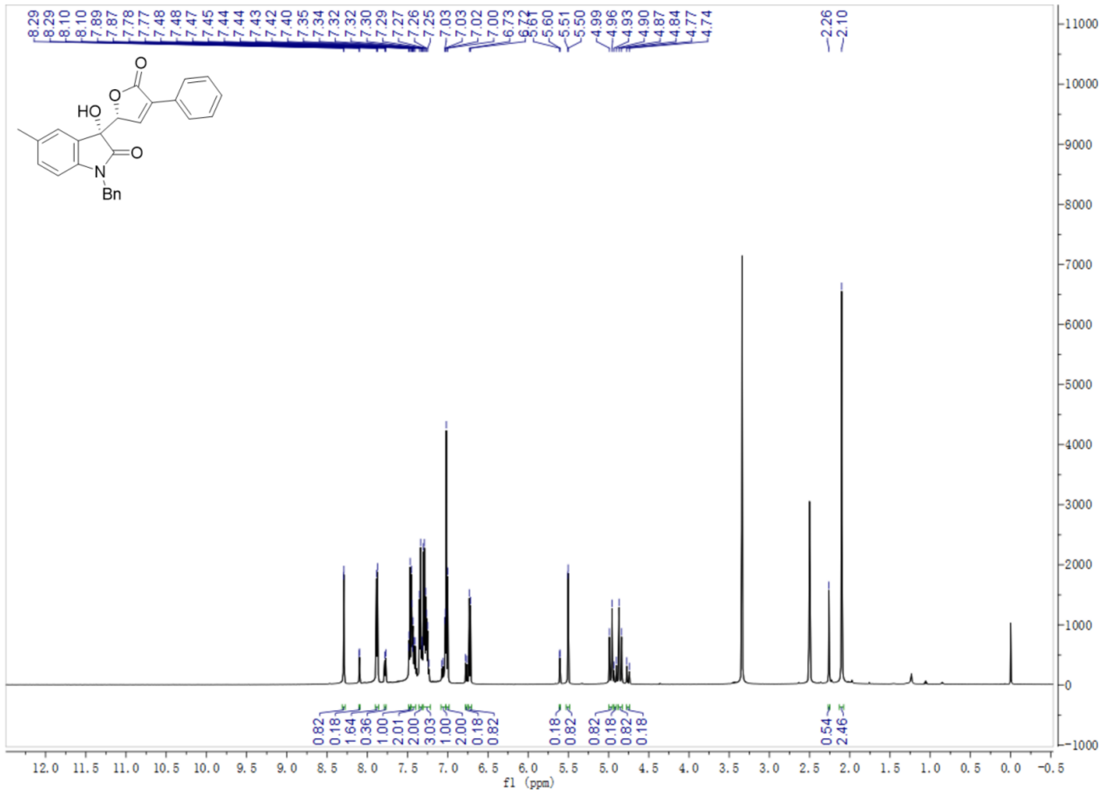

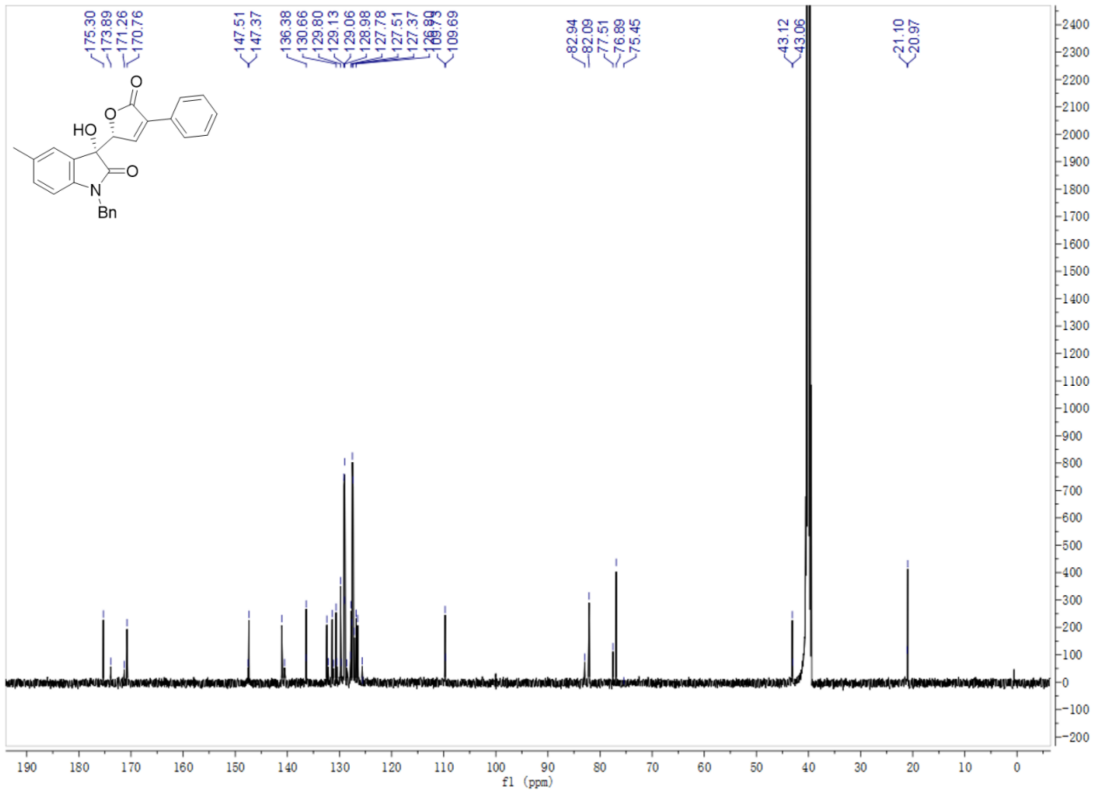

^{1}H-NMR and ^{13}C-NMR for **3l:**

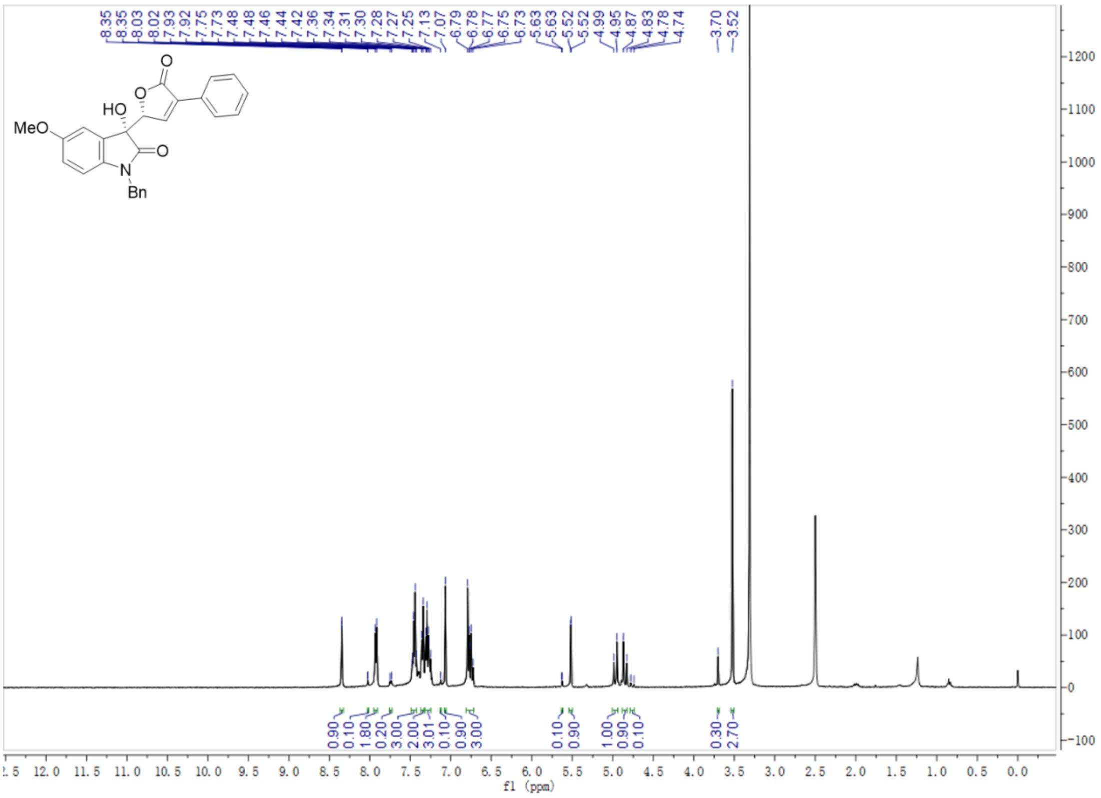

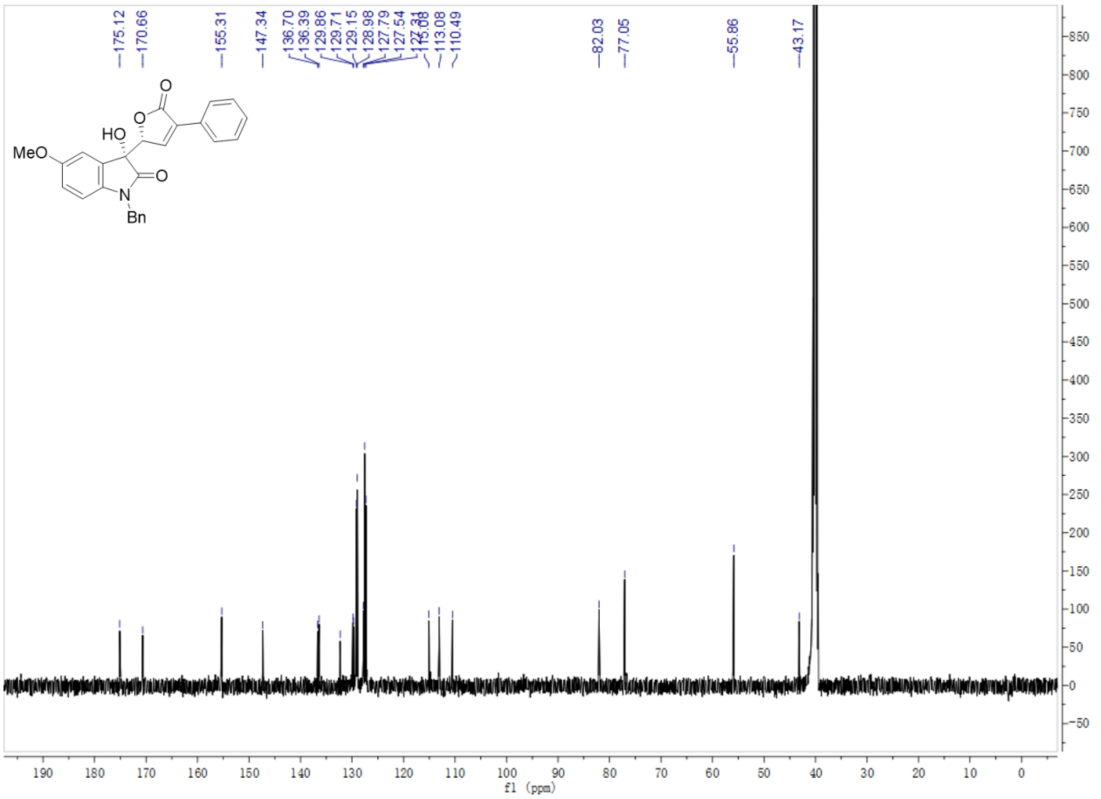

^{1}H-NMR and ^{13}C-NMR for **3m:**

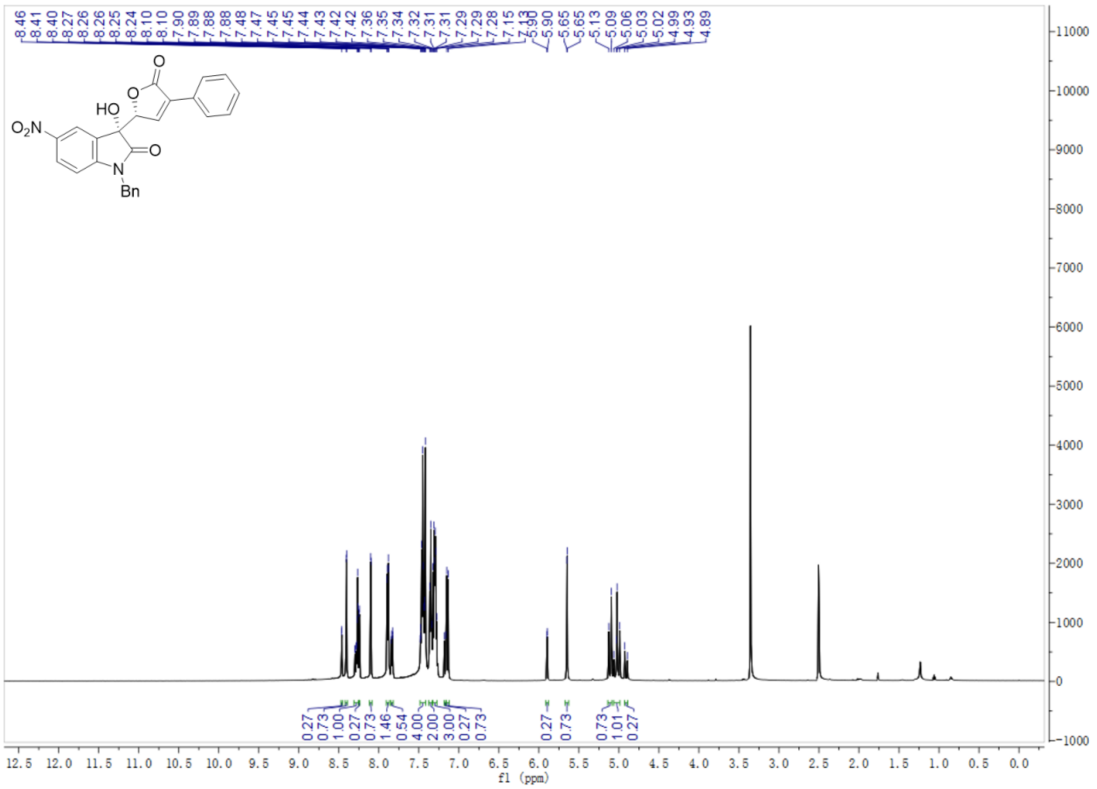

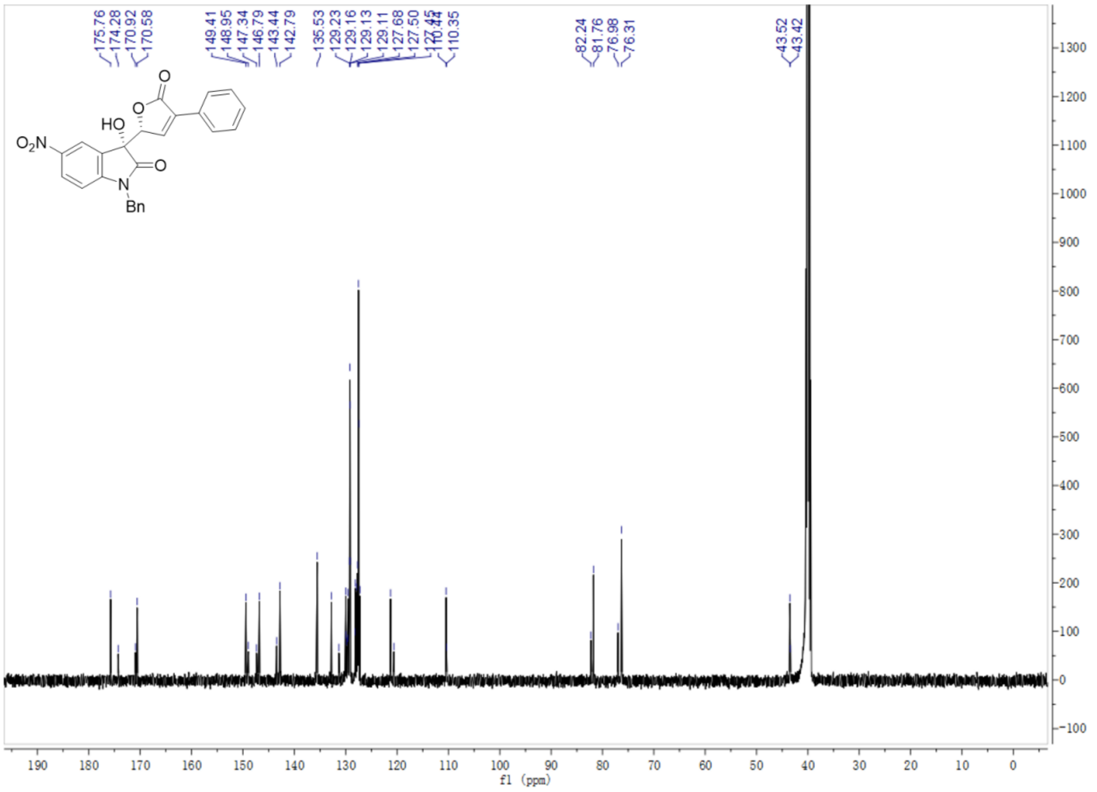

^{1}H-NMR and ^{13}C-NMR for **3n:**

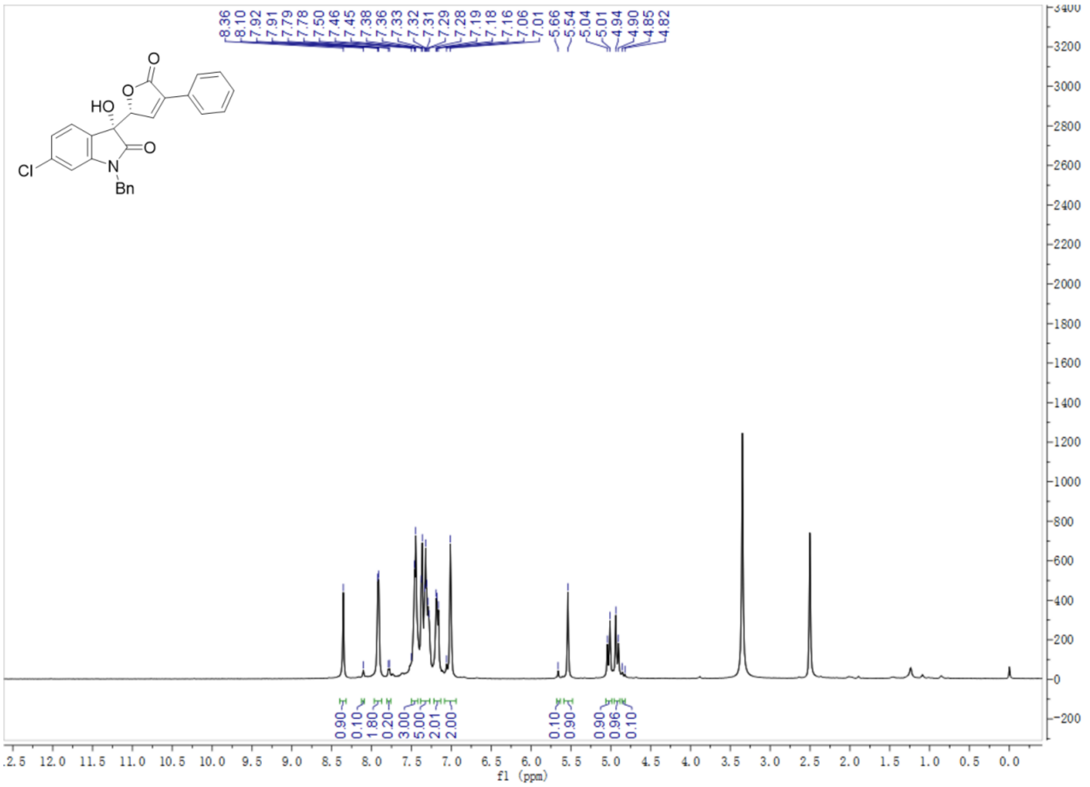

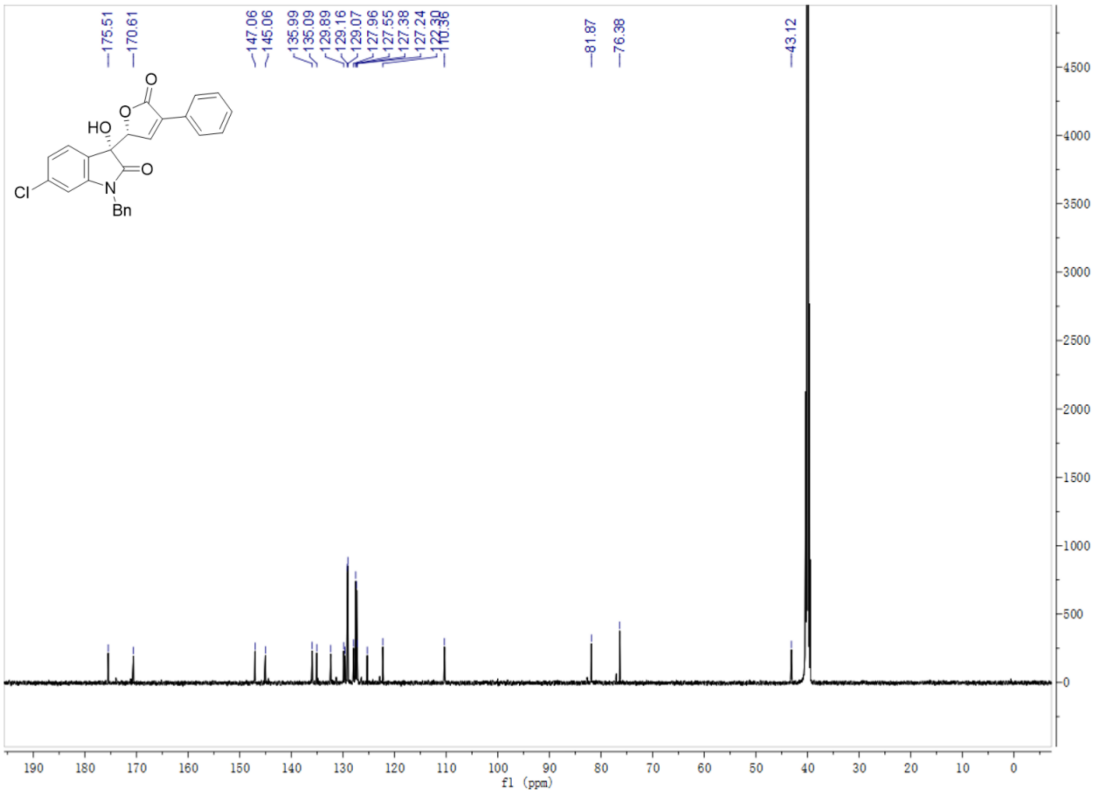

^{1}H-NMR and ^{13}C-NMR for **3o:**

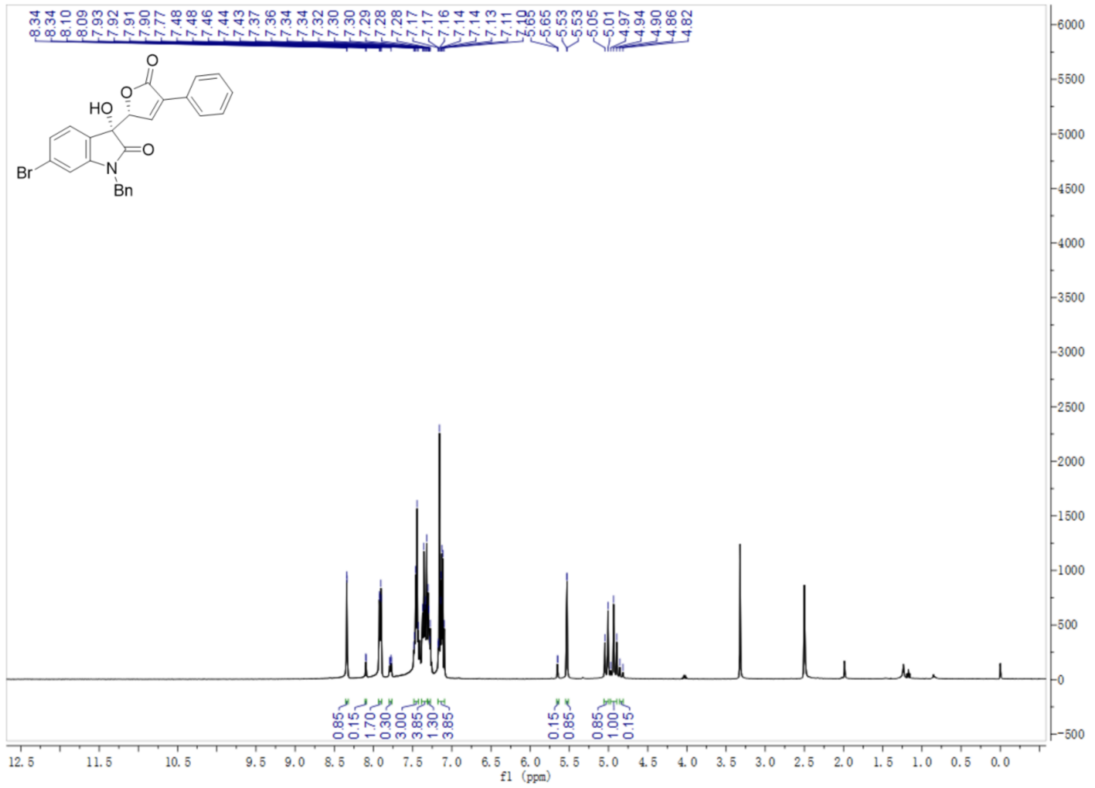

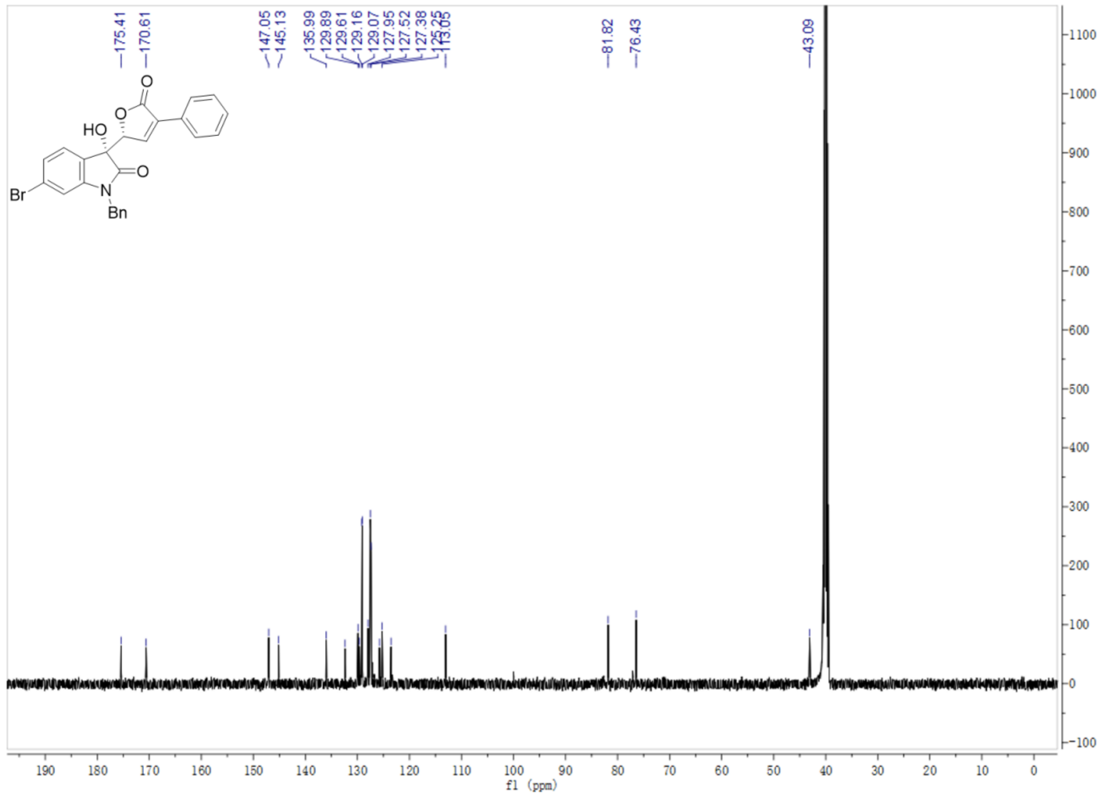

^{1}H-NMR and ^{13}C-NMR for **3p:**

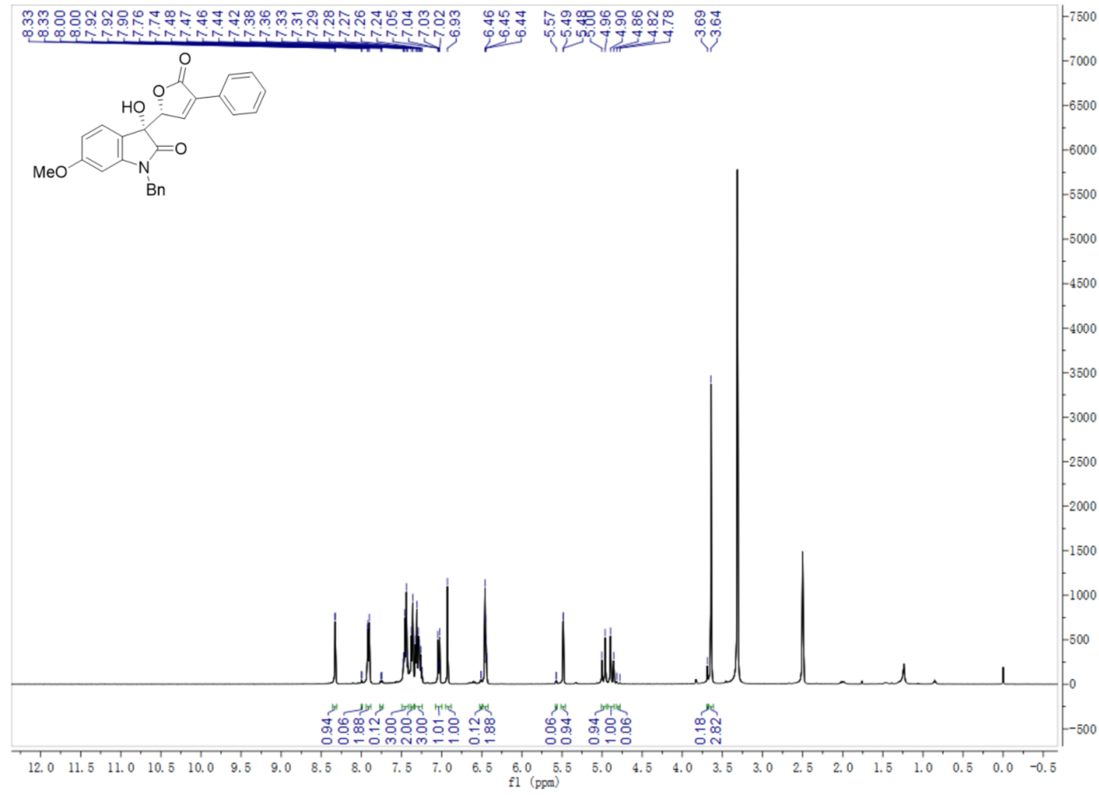

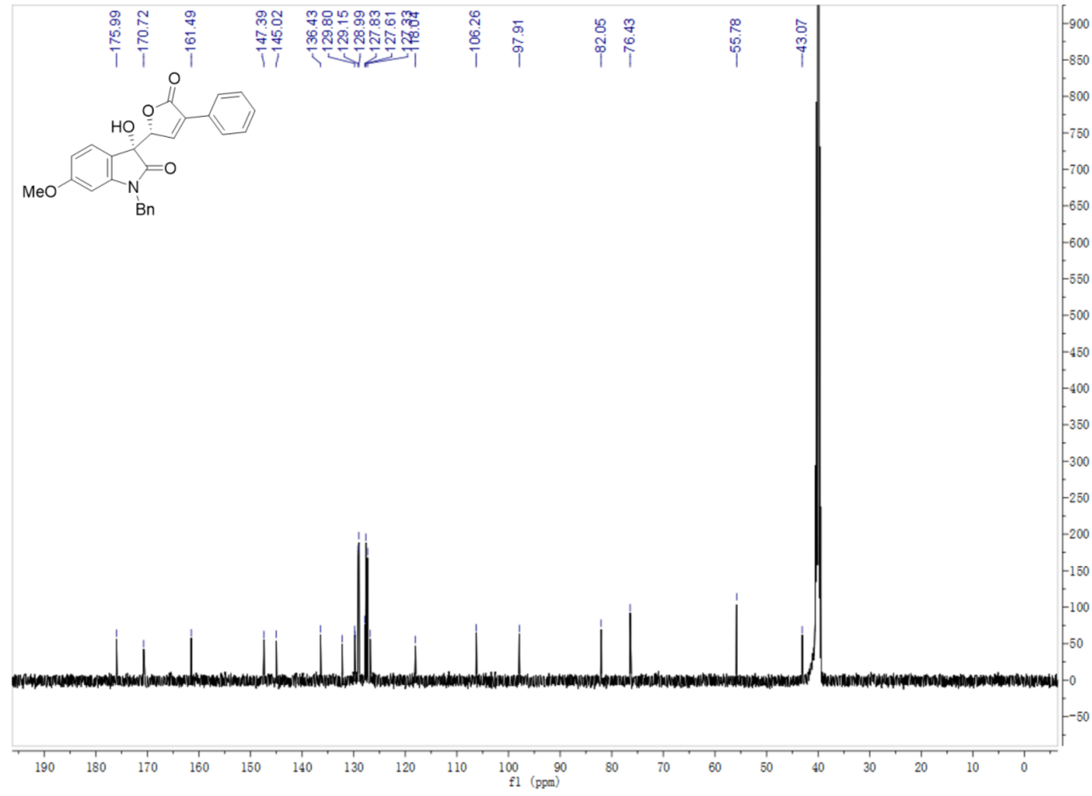

^{1}H-NMR , ^{13}C-NMR and ^{19}F NMR for **3q:**

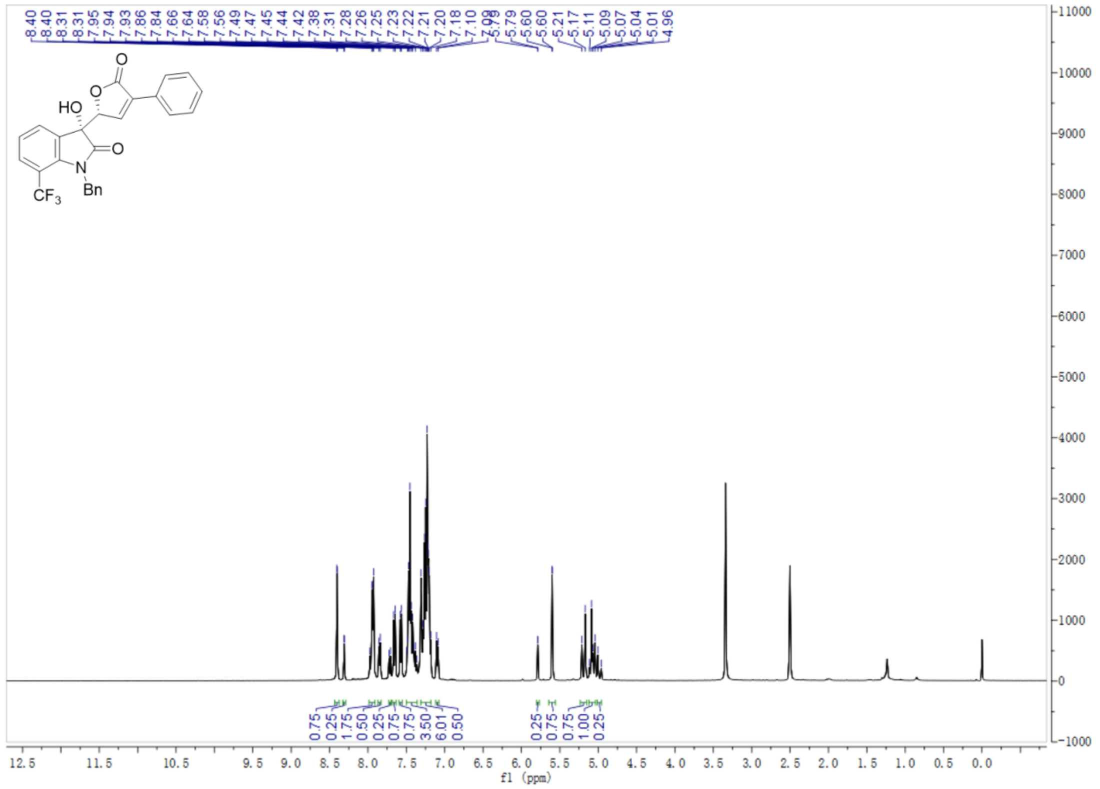

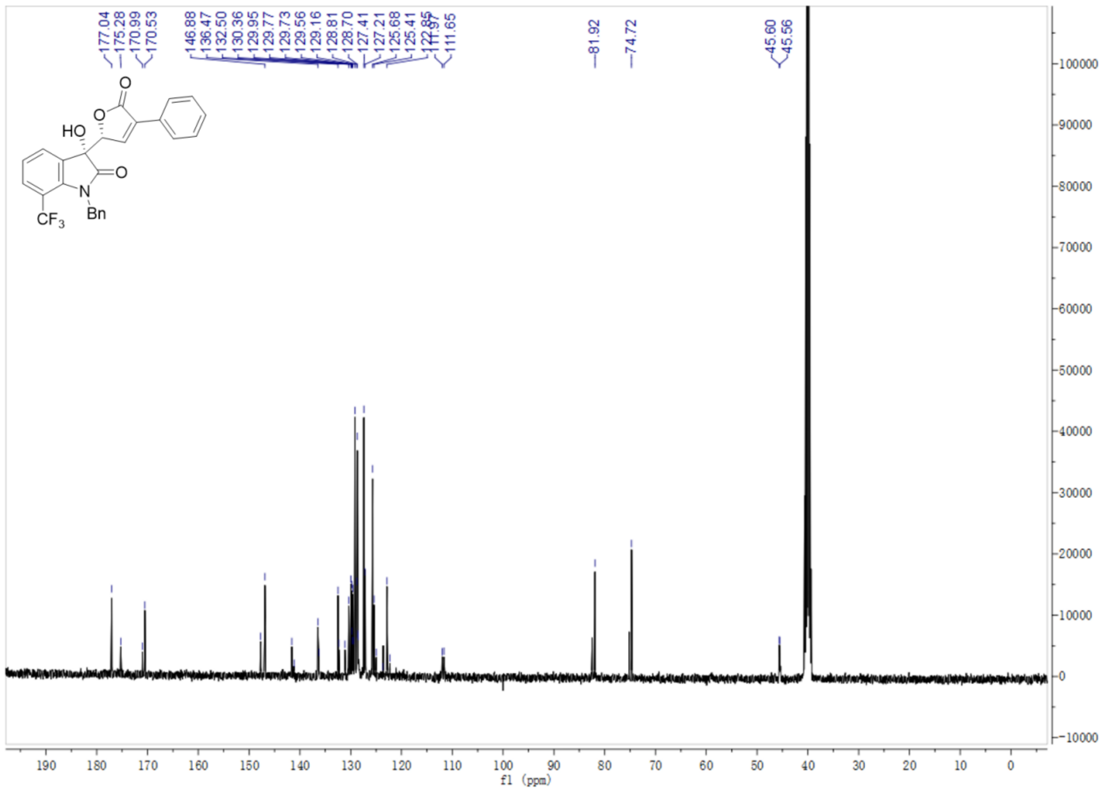

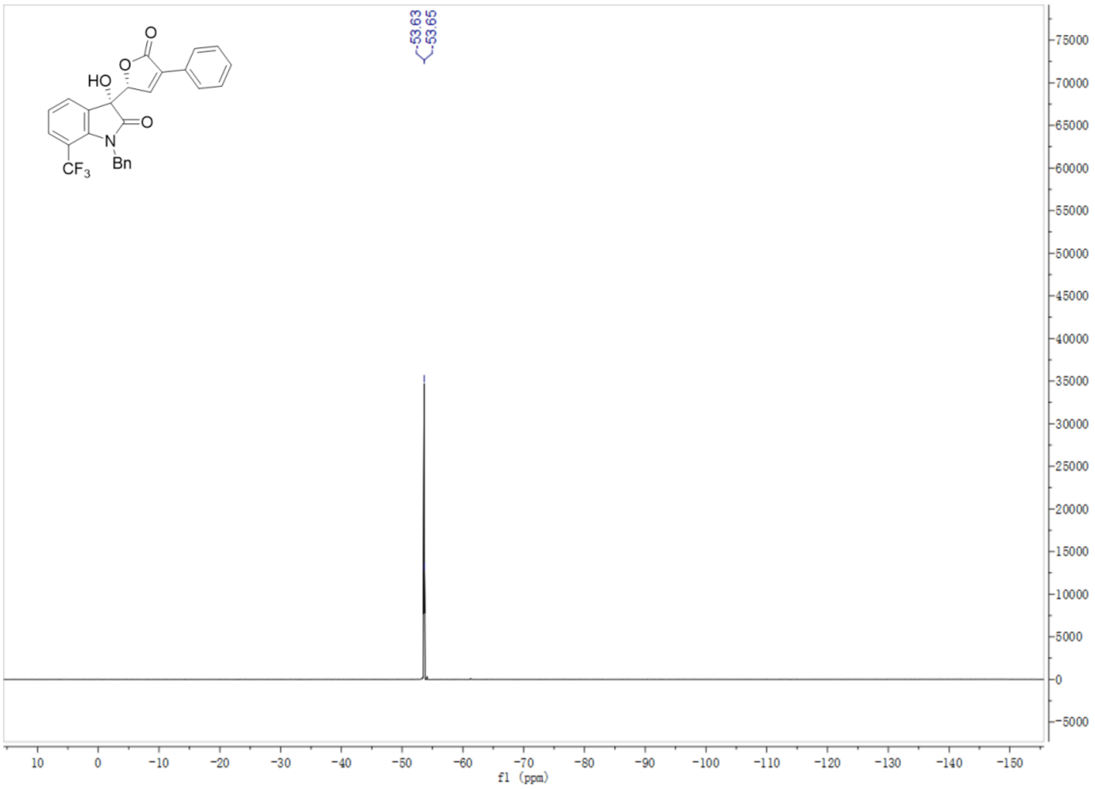

-53.63
-53.65
HO
O
O
O
N
Bn
CF3
f1 (ppm)

^{1}H-NMR and ^{13}C-NMR for **3r:**

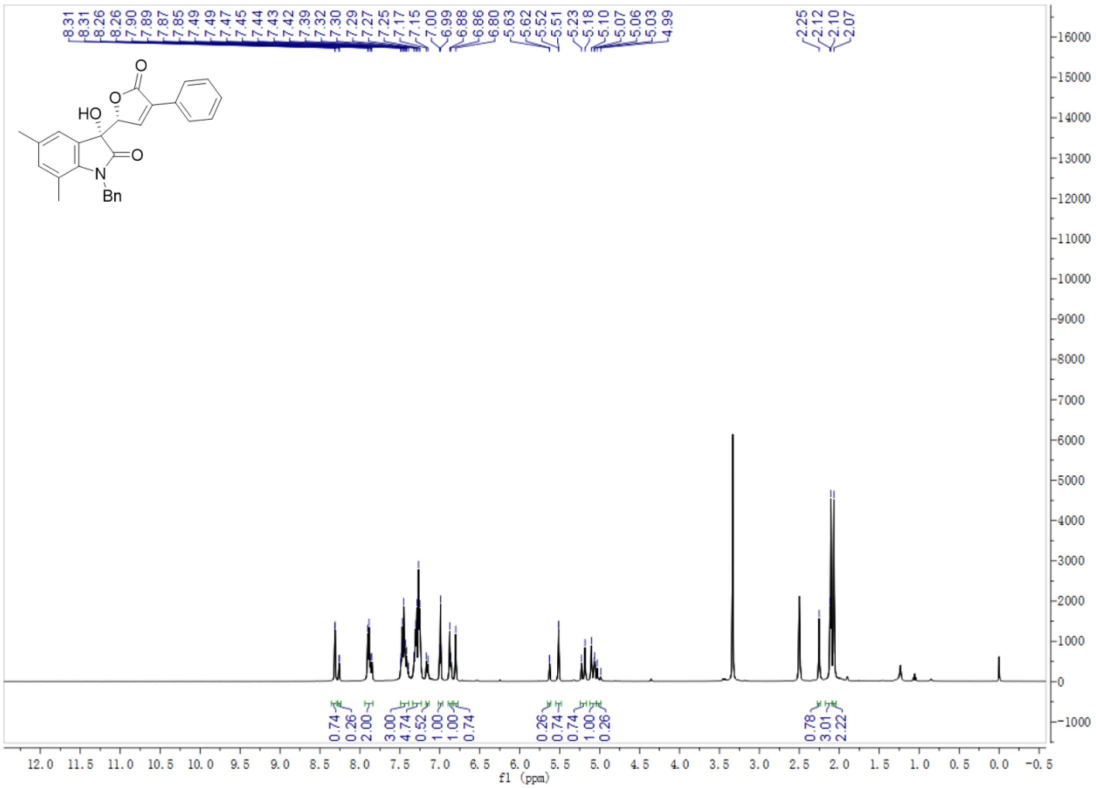

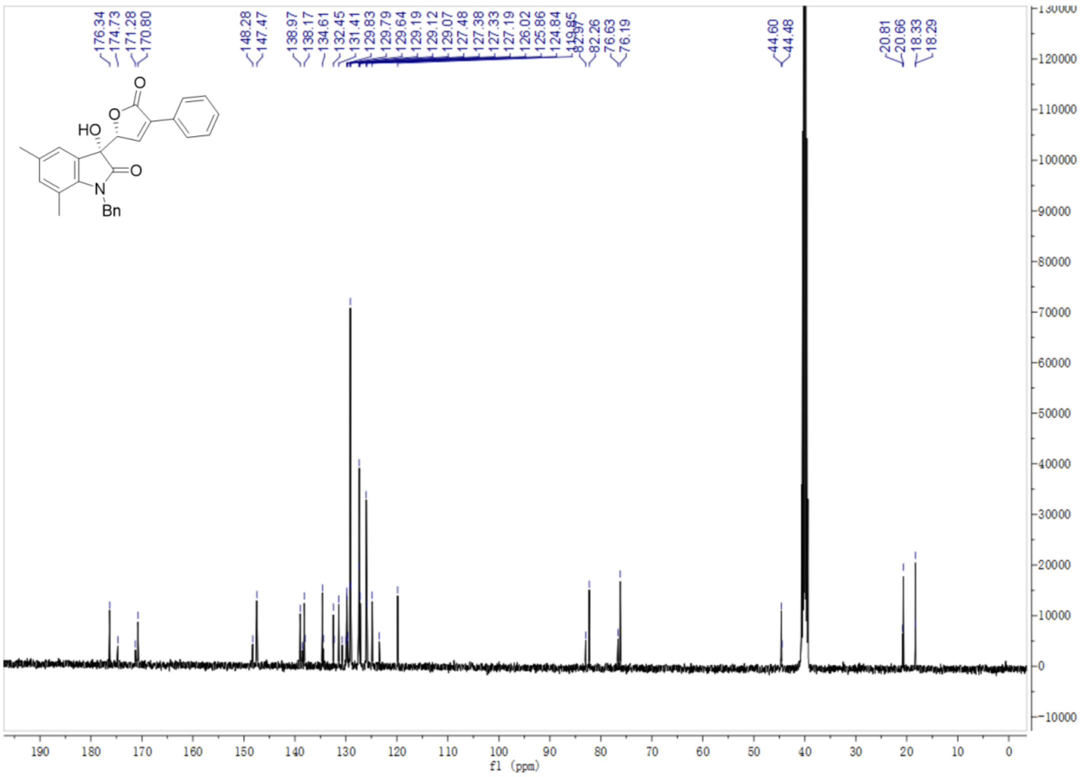

^{1}H-NMR and ^{13}C-NMR for **3s:**

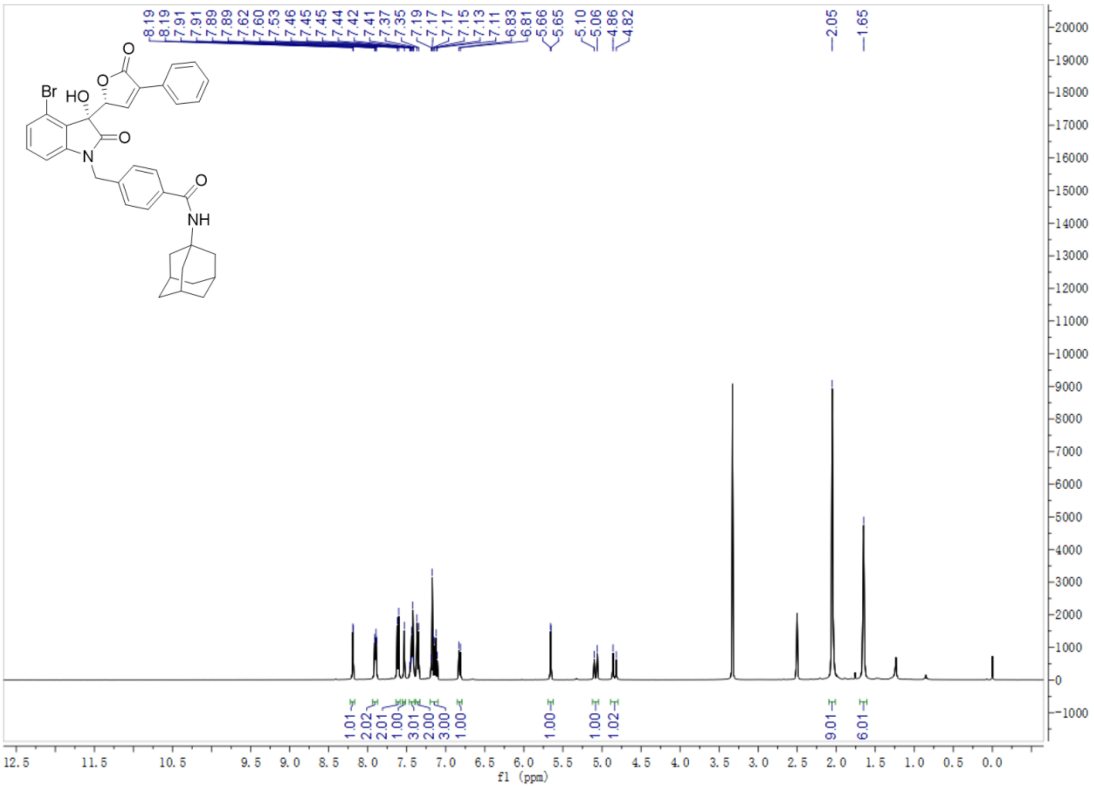

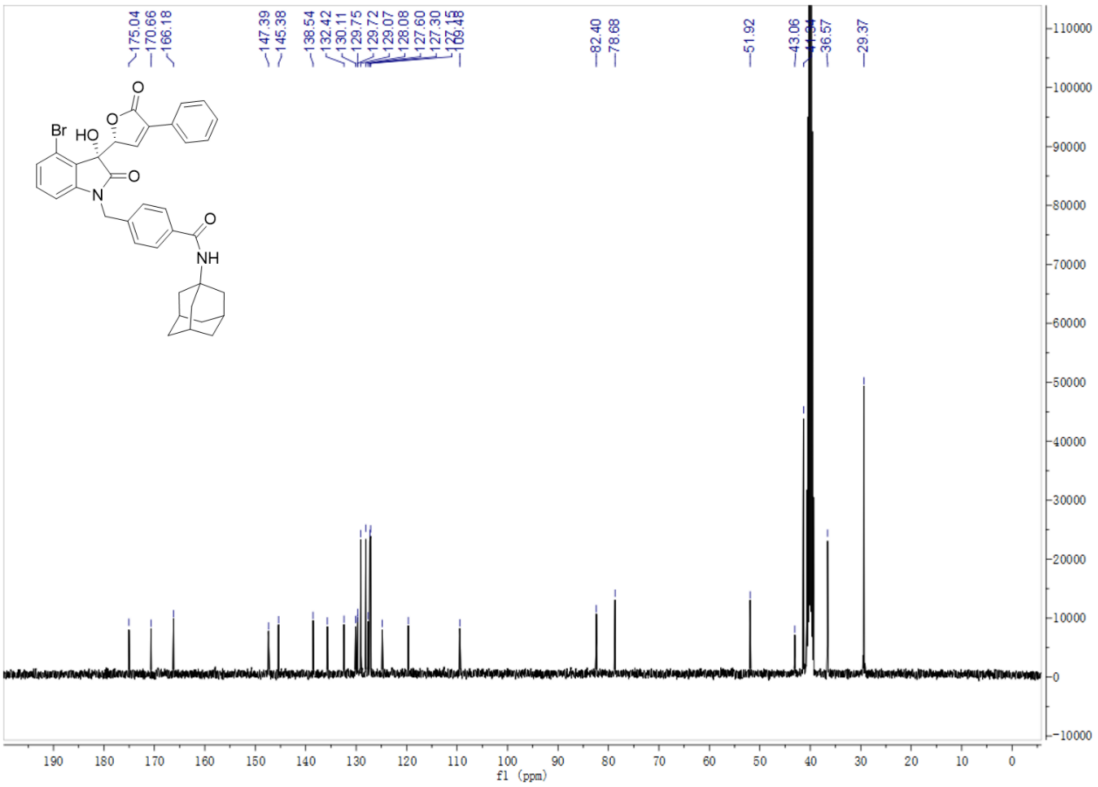

^{1}H-NMR and ^{13}C-NMR for **3t:**

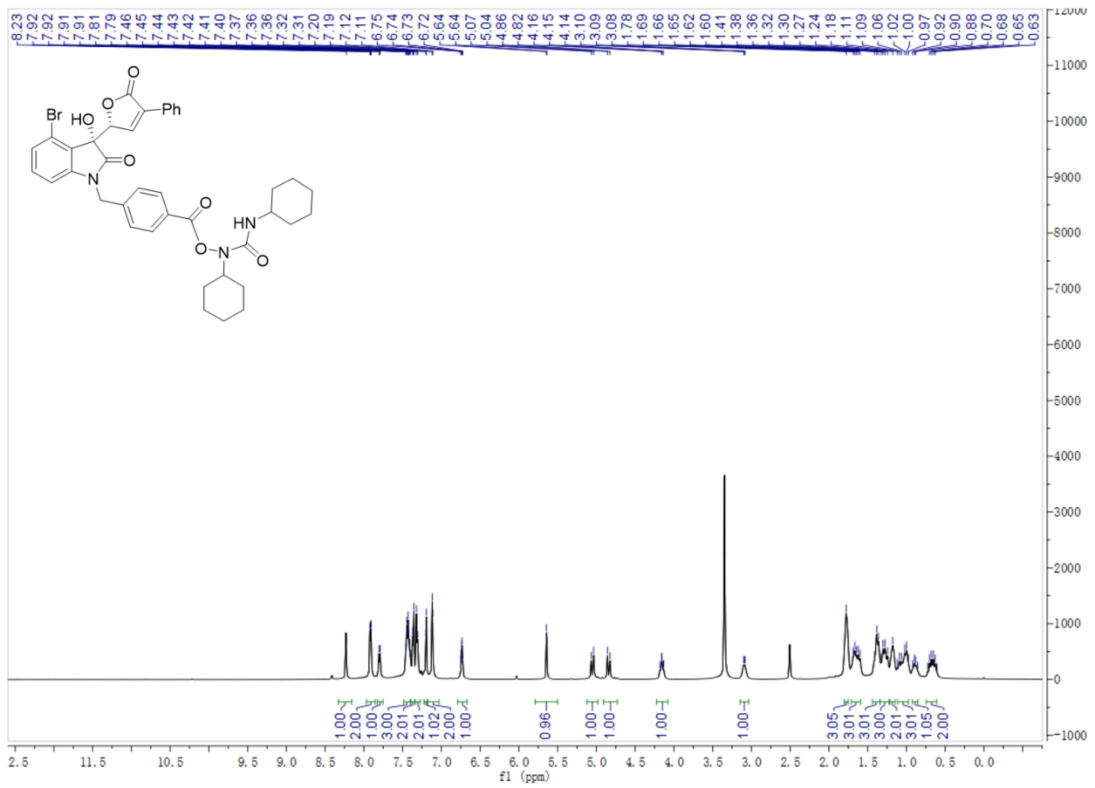

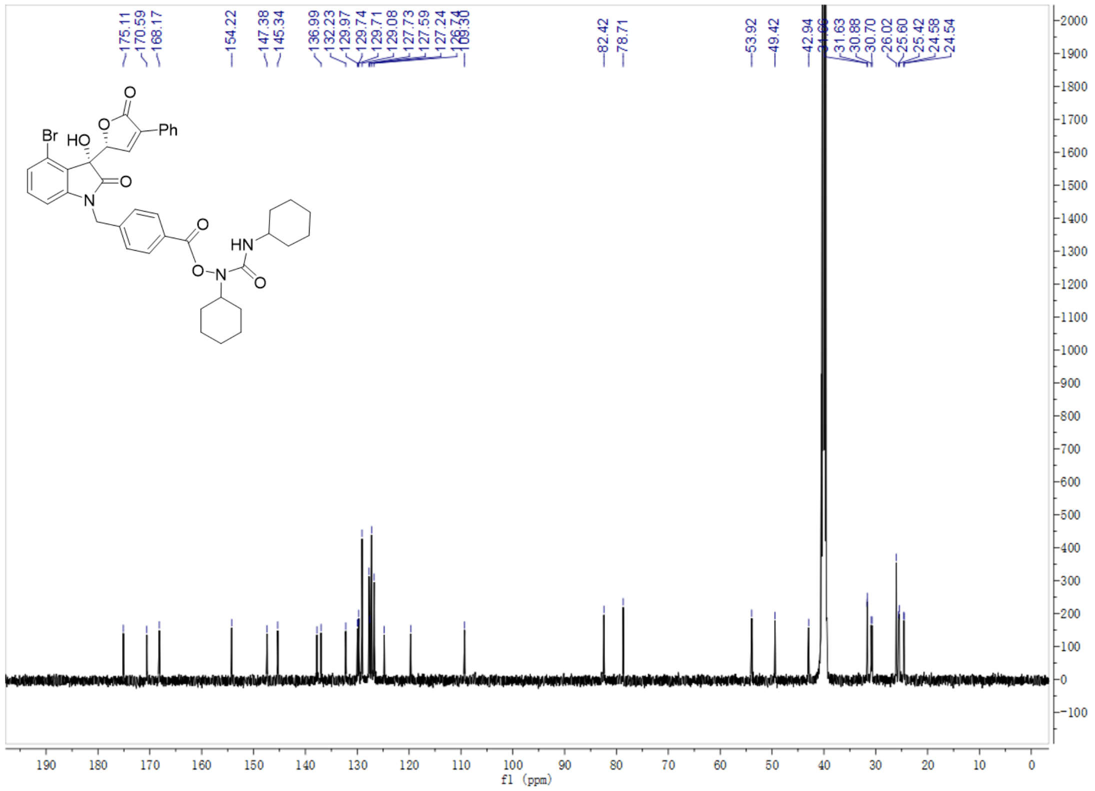

^{1}H-NMR and ^{13}C-NMR for **3u:**

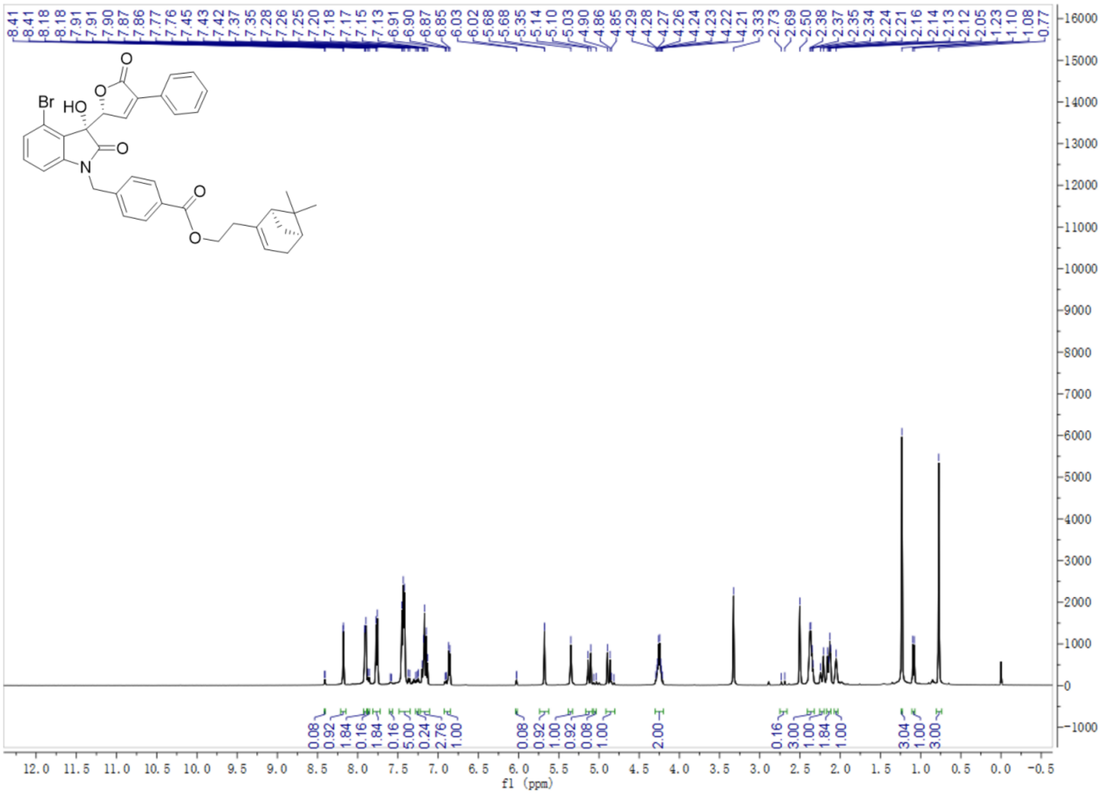

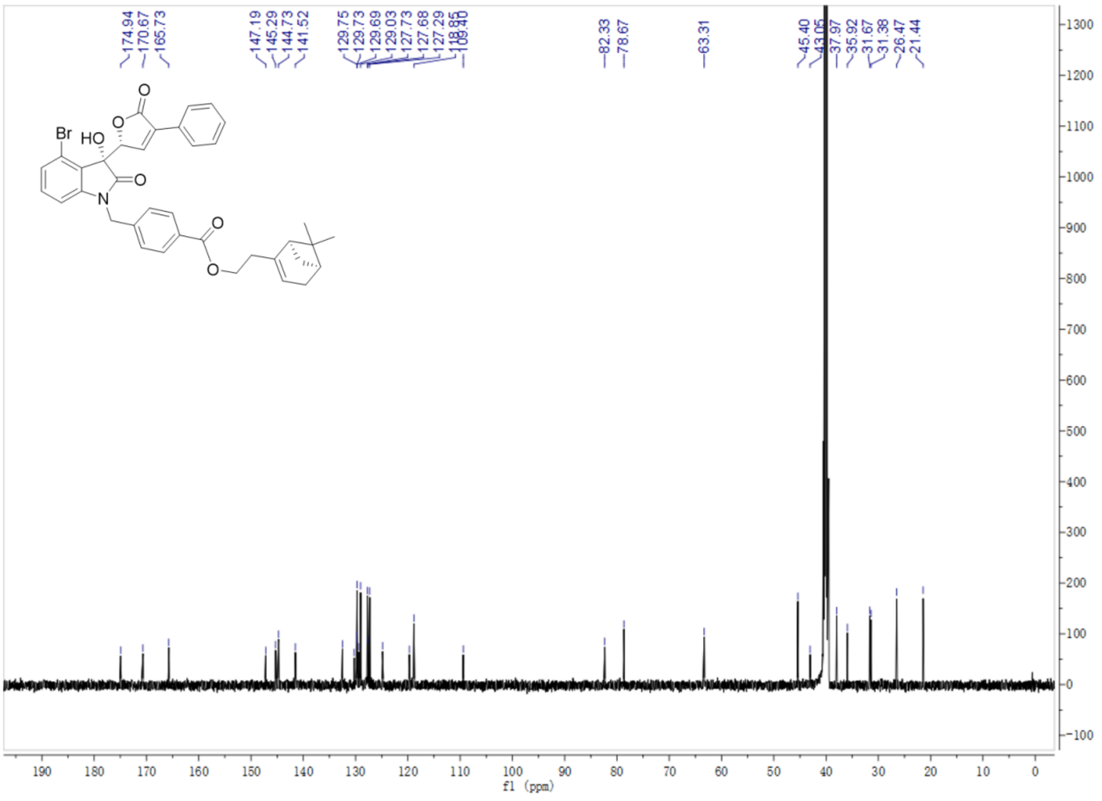

^{1}H-NMR, ^{13}C-NMR and ^{19}F NMR for **3v:**

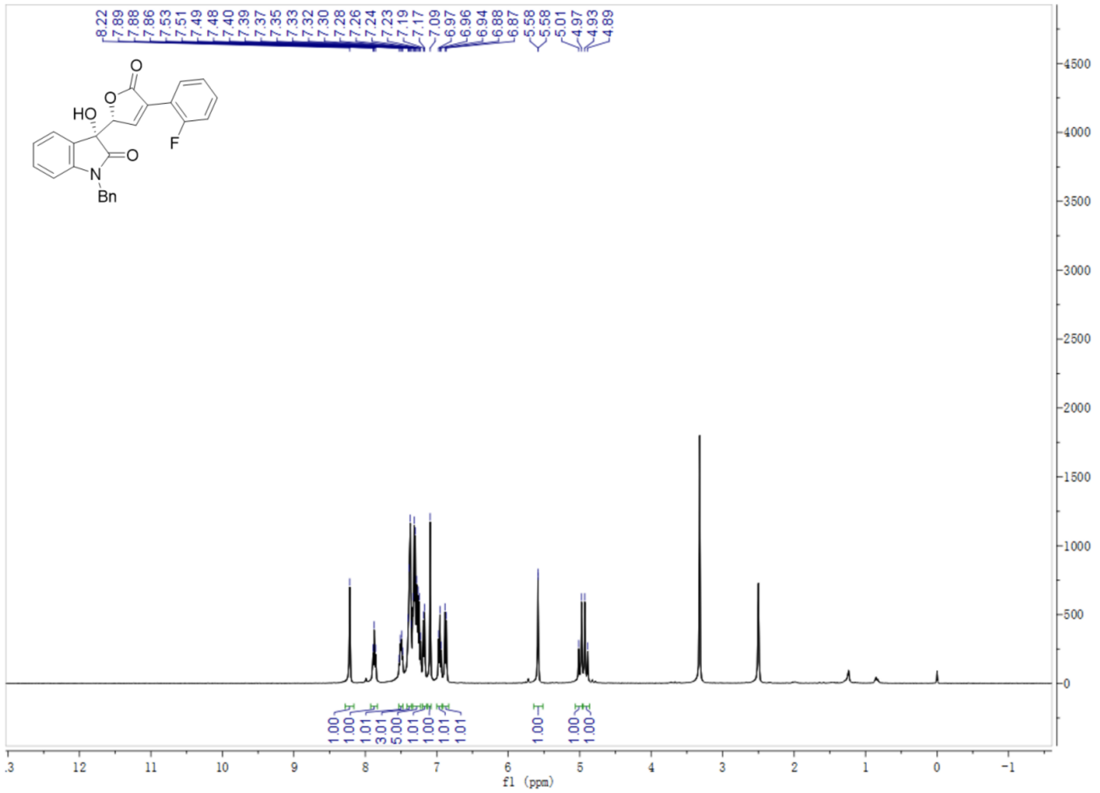

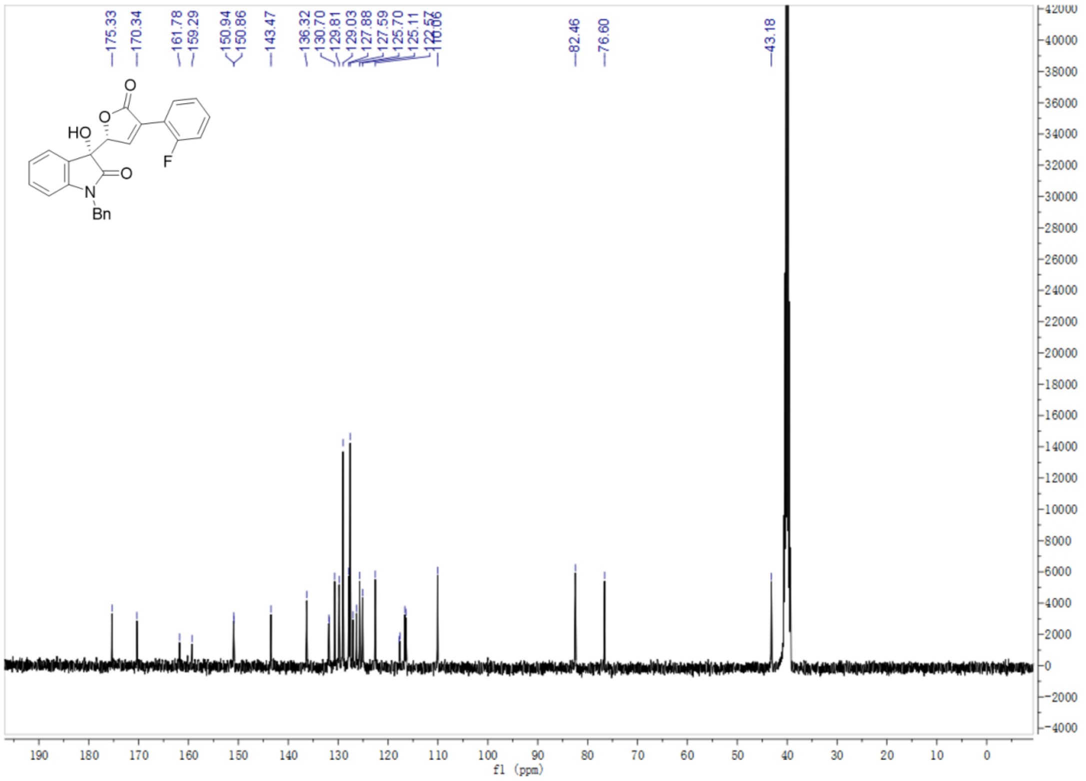

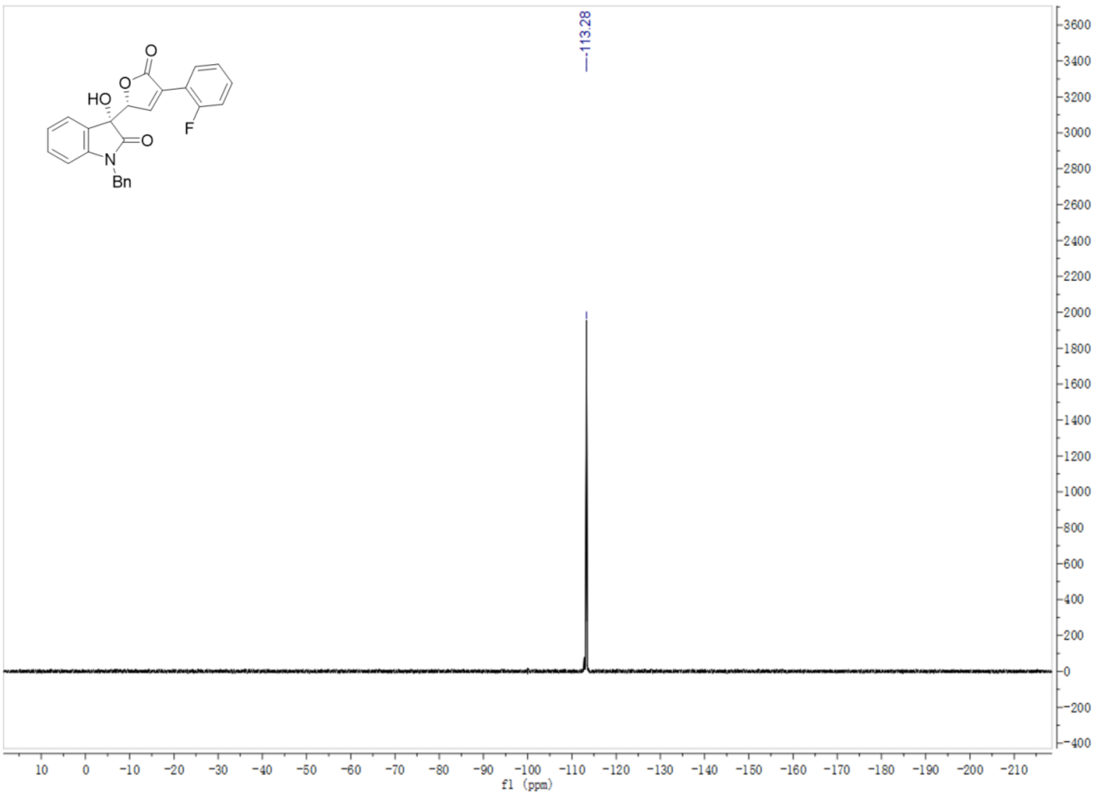

-113.28
HO
O
O
O
F
N
Bn
f1 (ppm)

^{1}H-NMR and ^{13}C-NMR for **3w:**

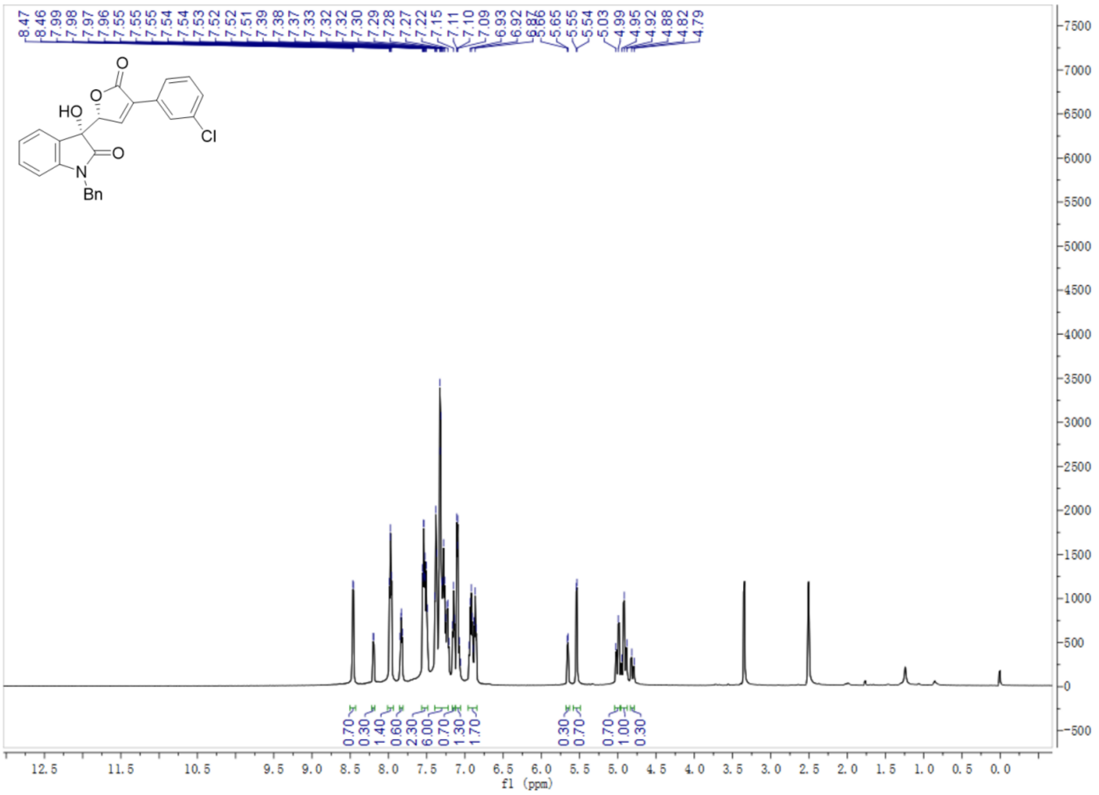

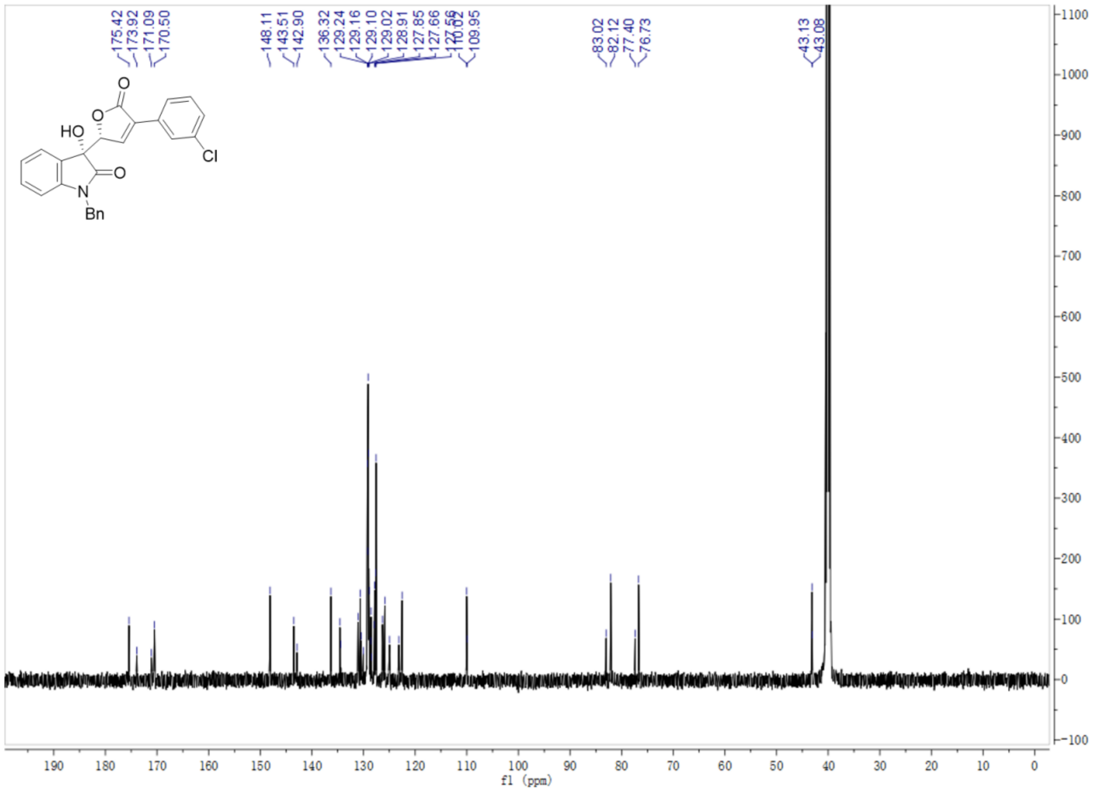

^{1}H-NMR and ^{13}C-NMR for **3x:**

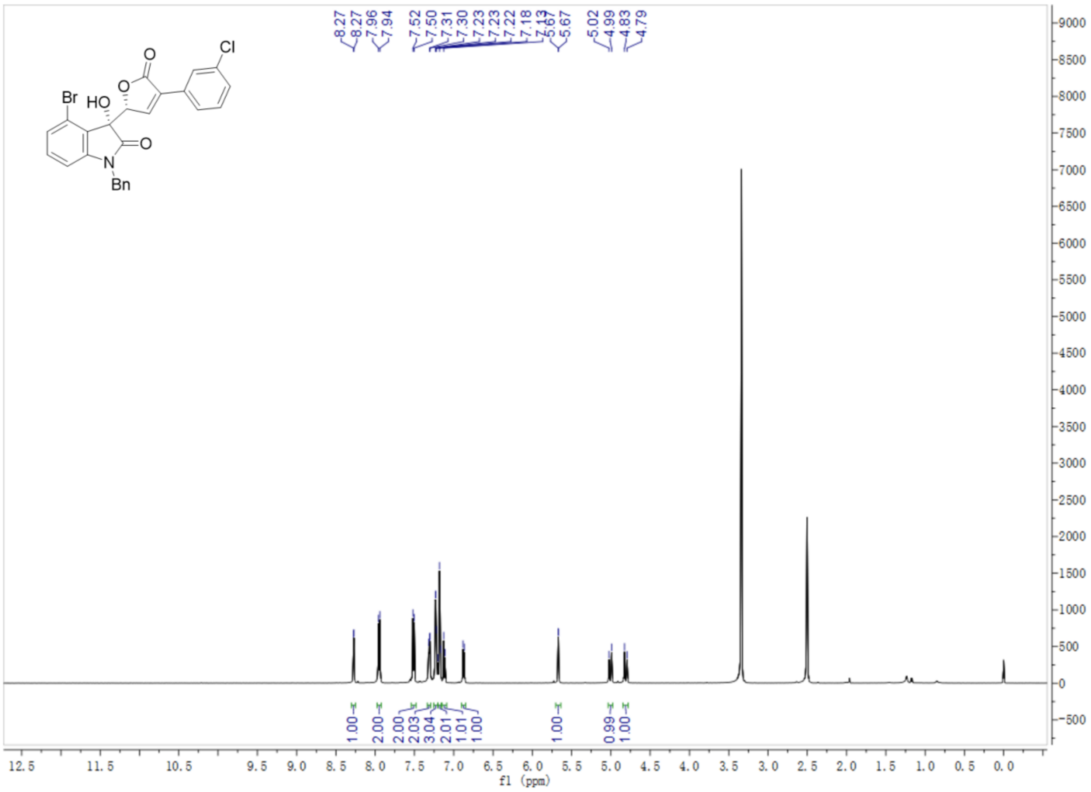

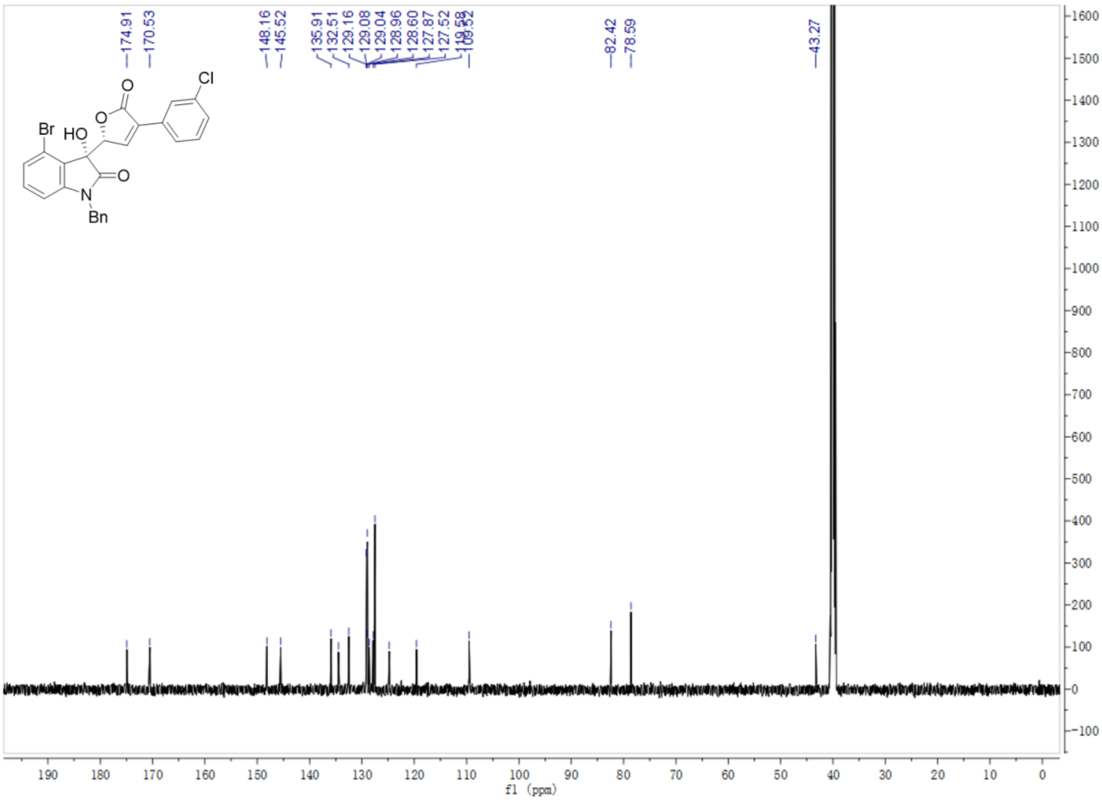

^{1}H-NMR, ^{13}C-NMR and ^{19}F NMR for **3y:**

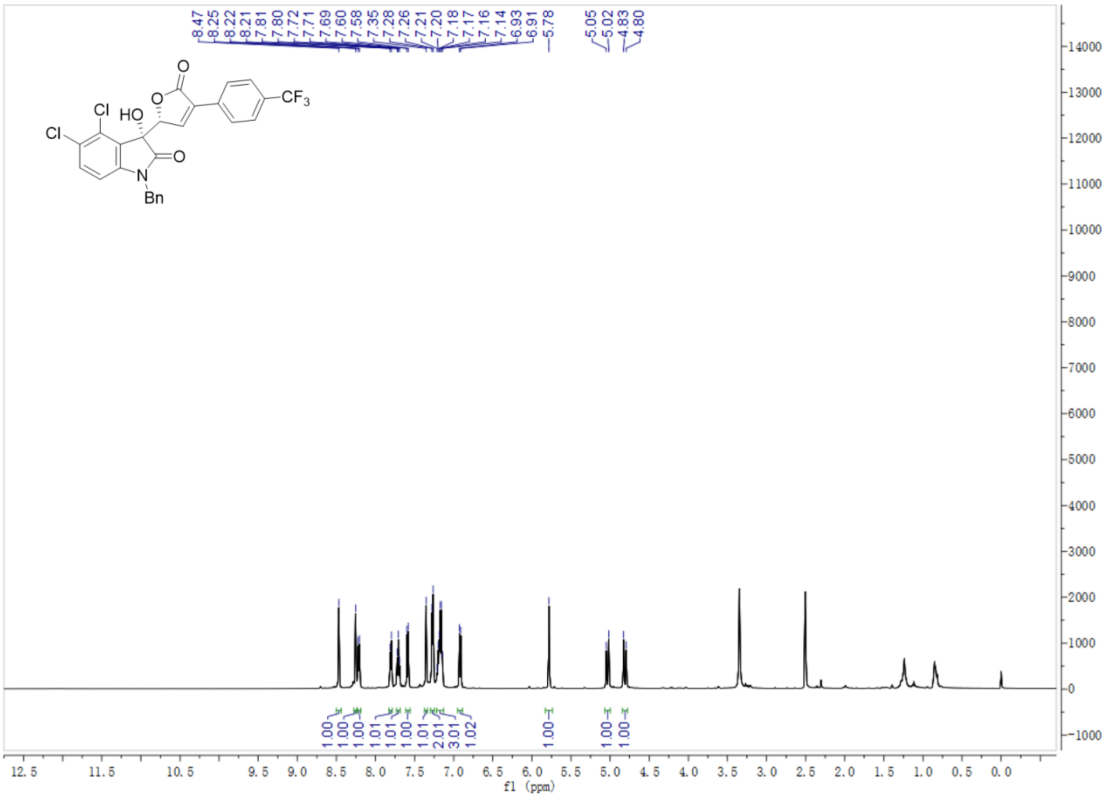

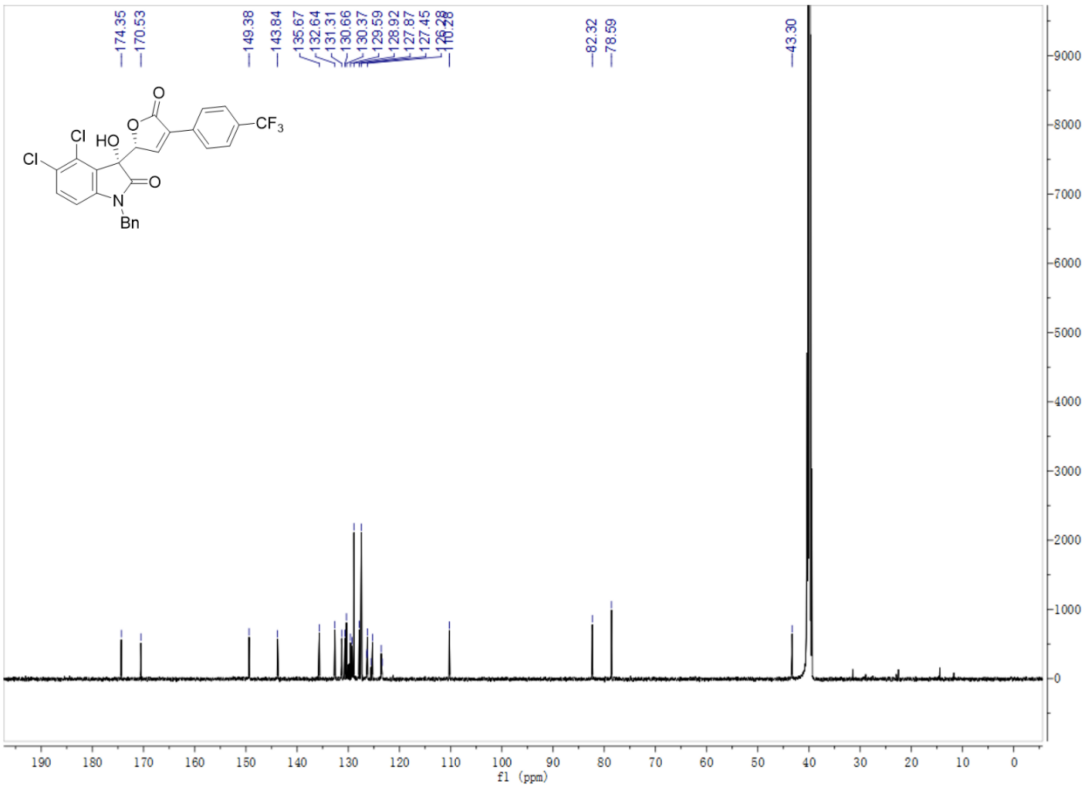

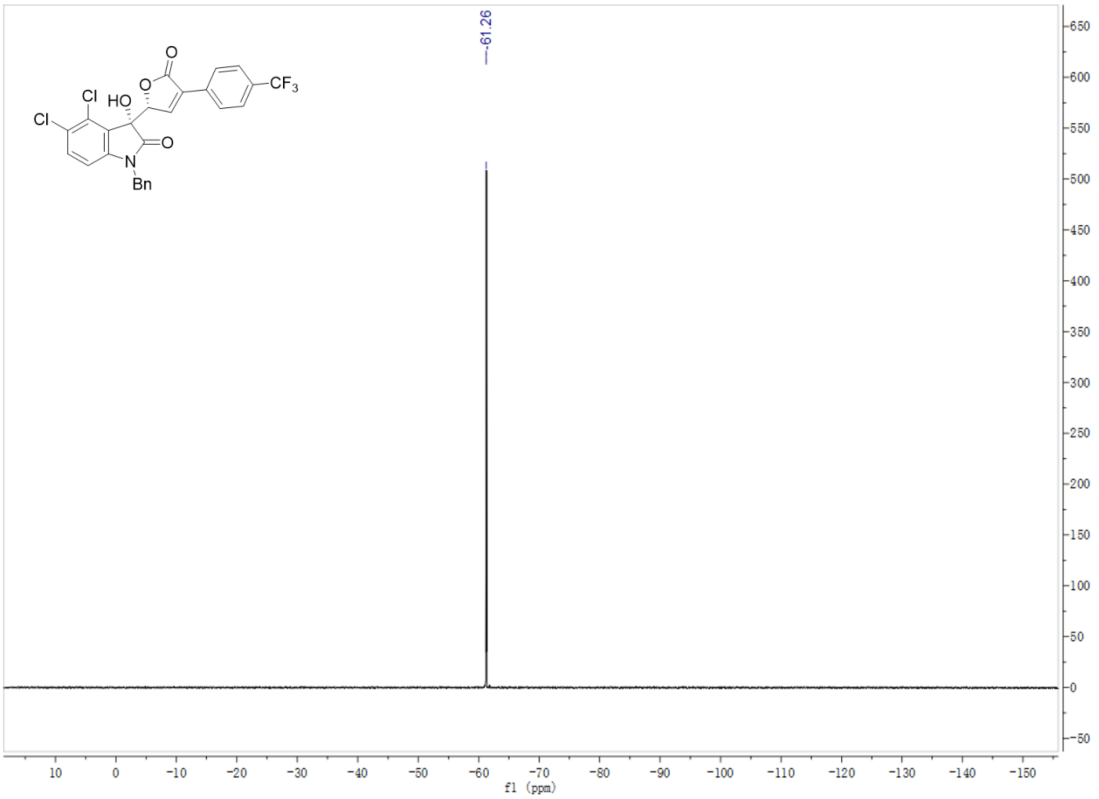
-61.26
Cl
Cl
HO
O
O
CF3
O
N
Bn
650
600
550
500
450
400
350
300
250
200
150
100
50
0
-50
10
0
-10
-20
-30
-40
-50
-60
-70
-80
-90
-100
-110
-120
-130
-140
-150
f1 (ppm)

^{1}H-NMR and ^{13}C-NMR for **3z:**

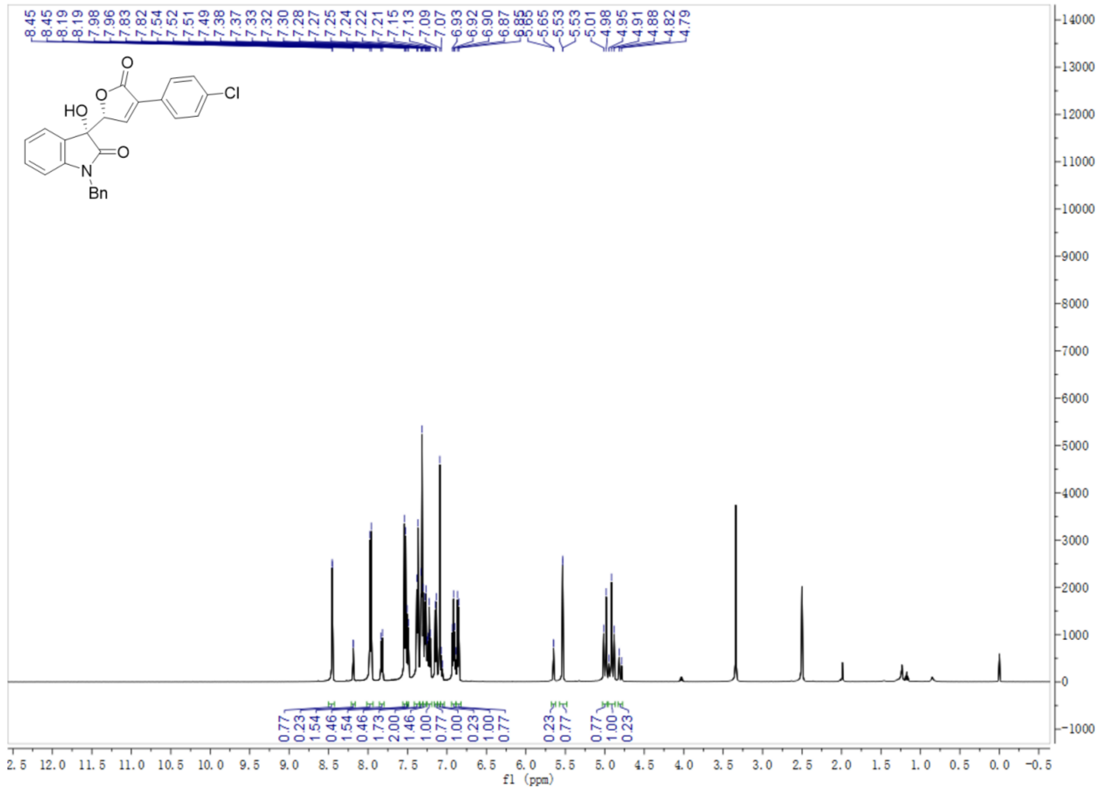

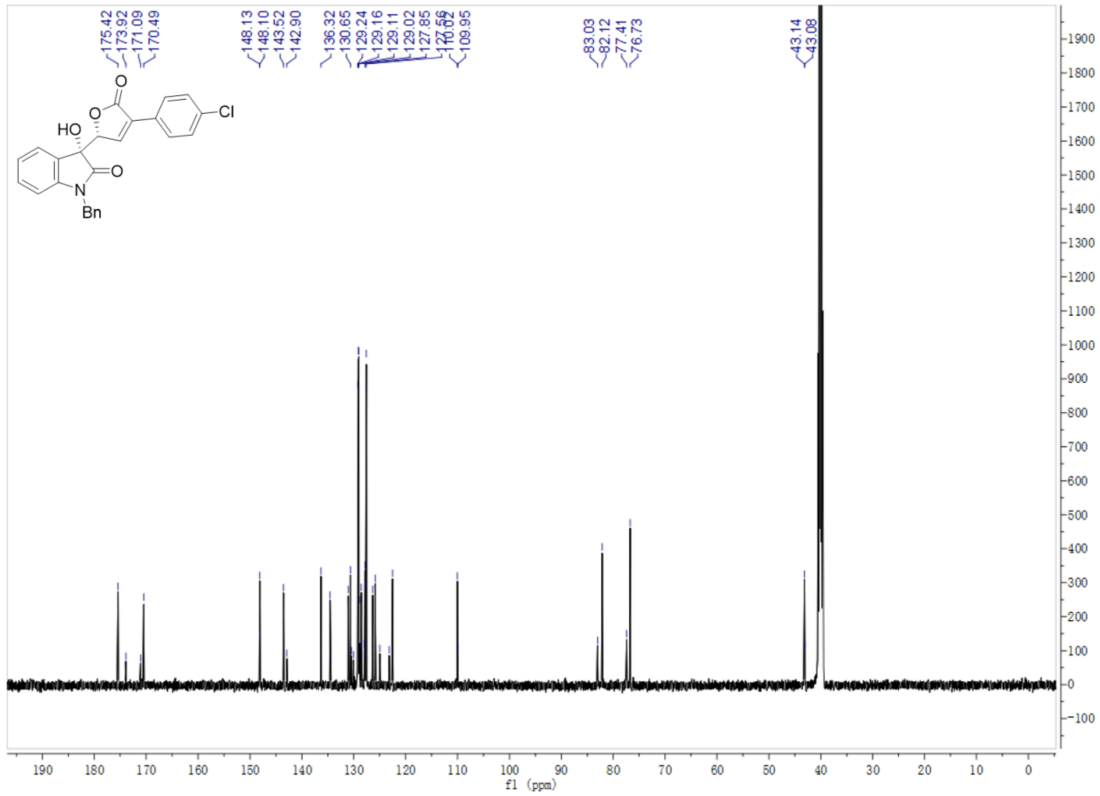

^{1}H-NMR and ^{13}C-NMR for **3aa:**

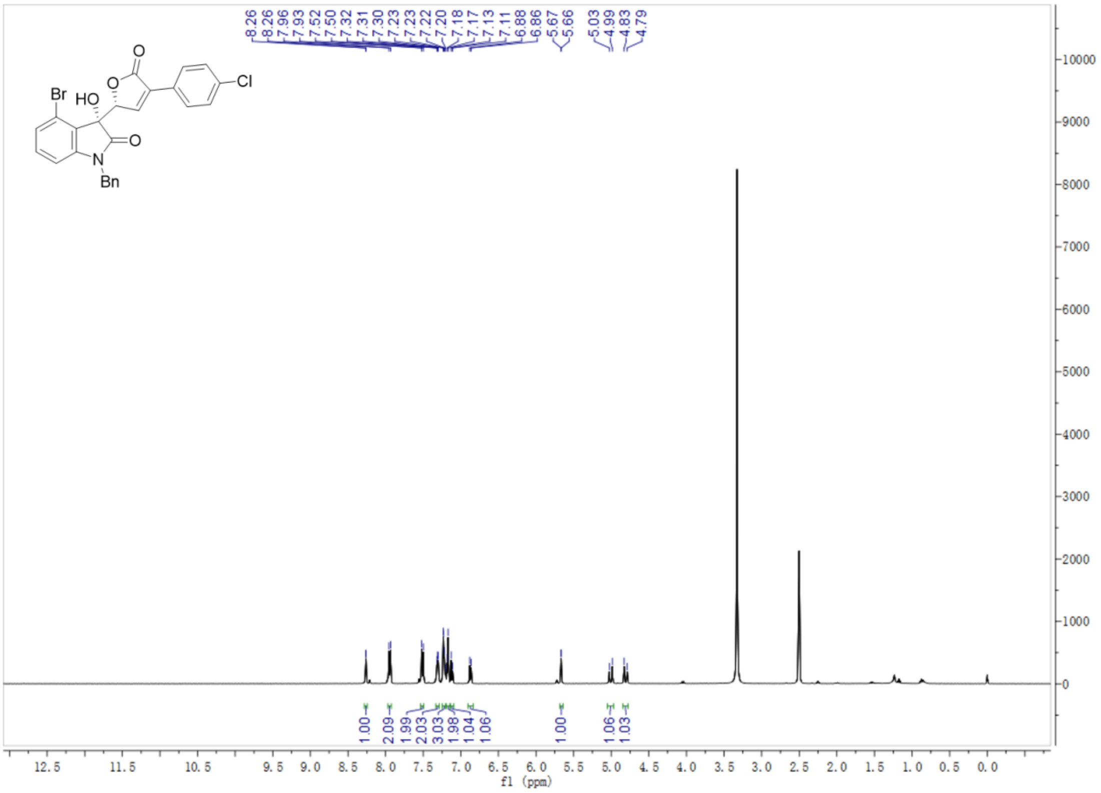

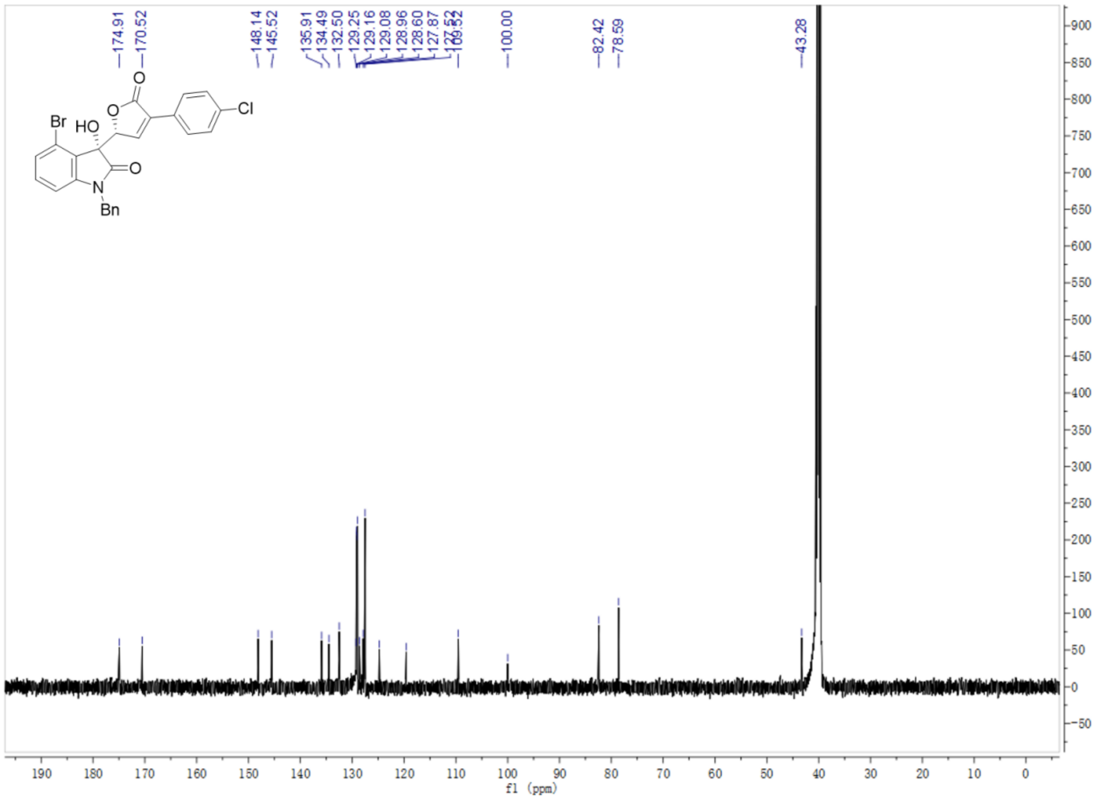

^{1}H-NMR and ^{13}C-NMR for **3ab:**

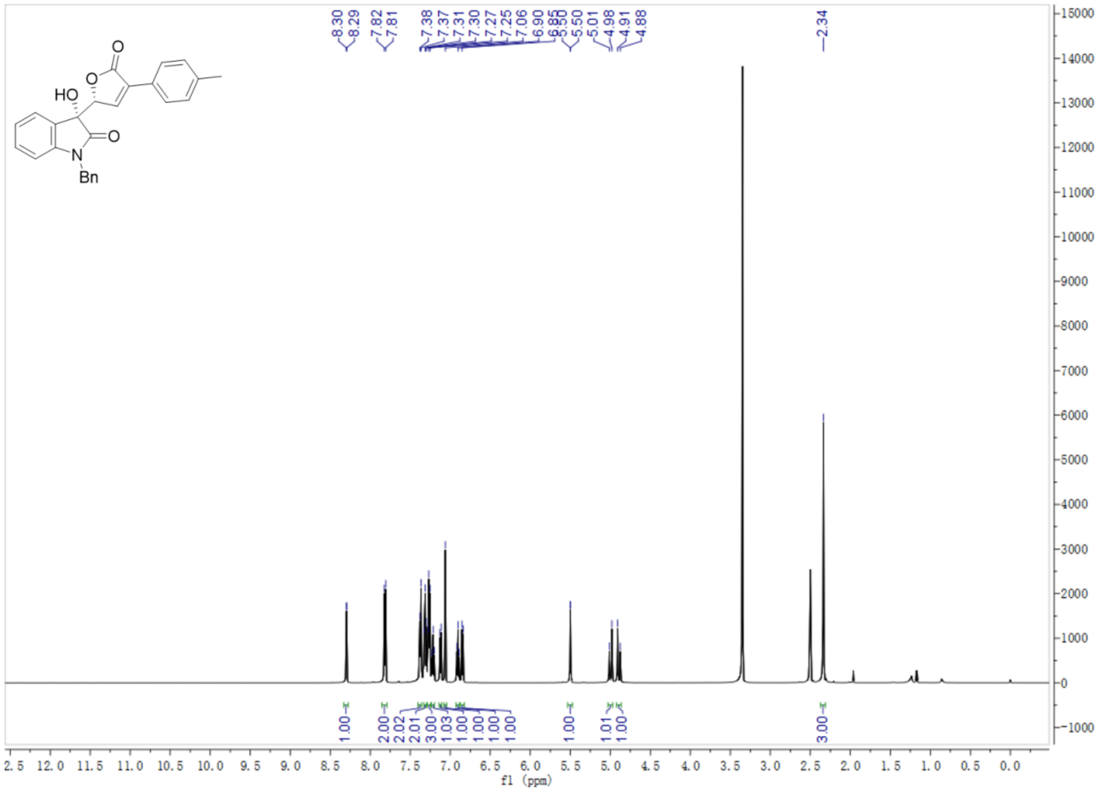

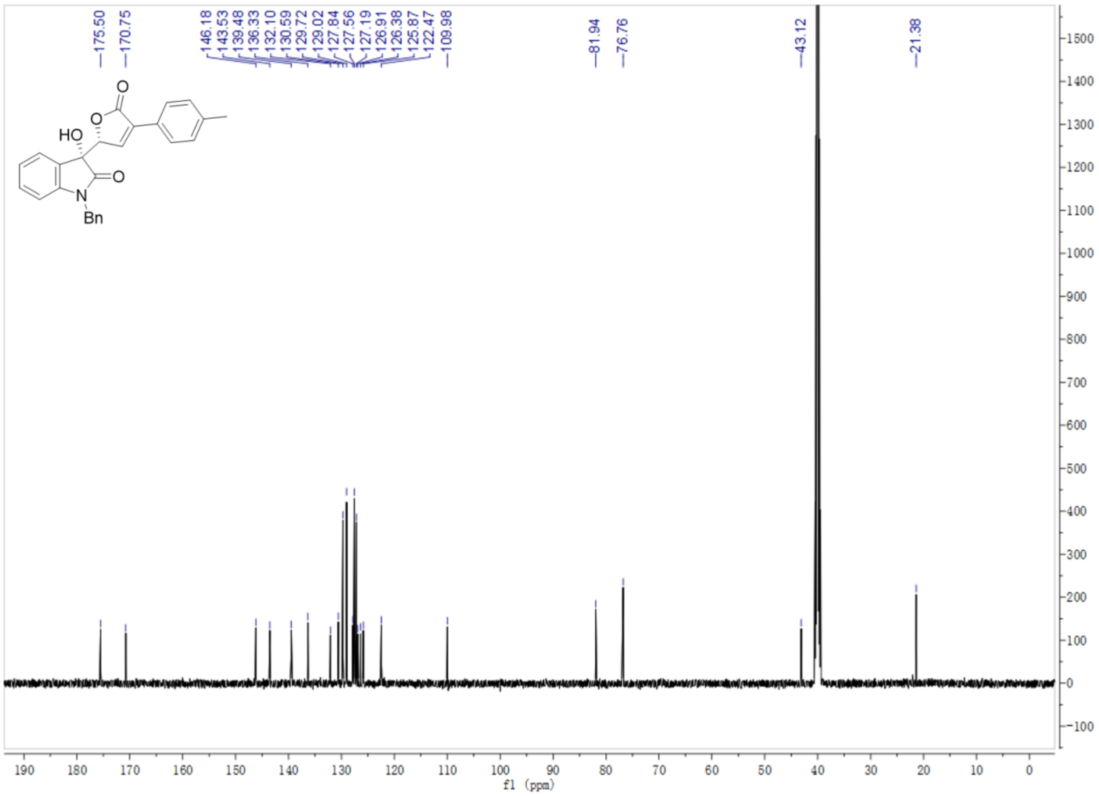

^{1}H-NMR and ^{13}C-NMR for **3ac:**

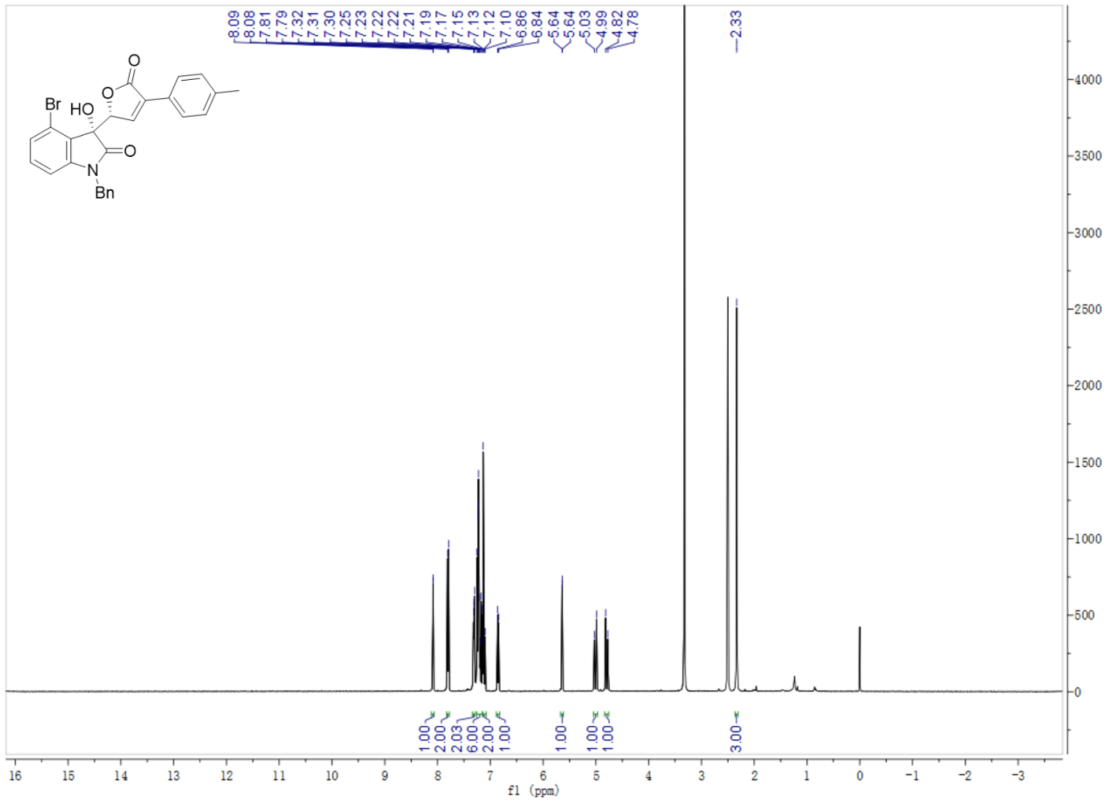

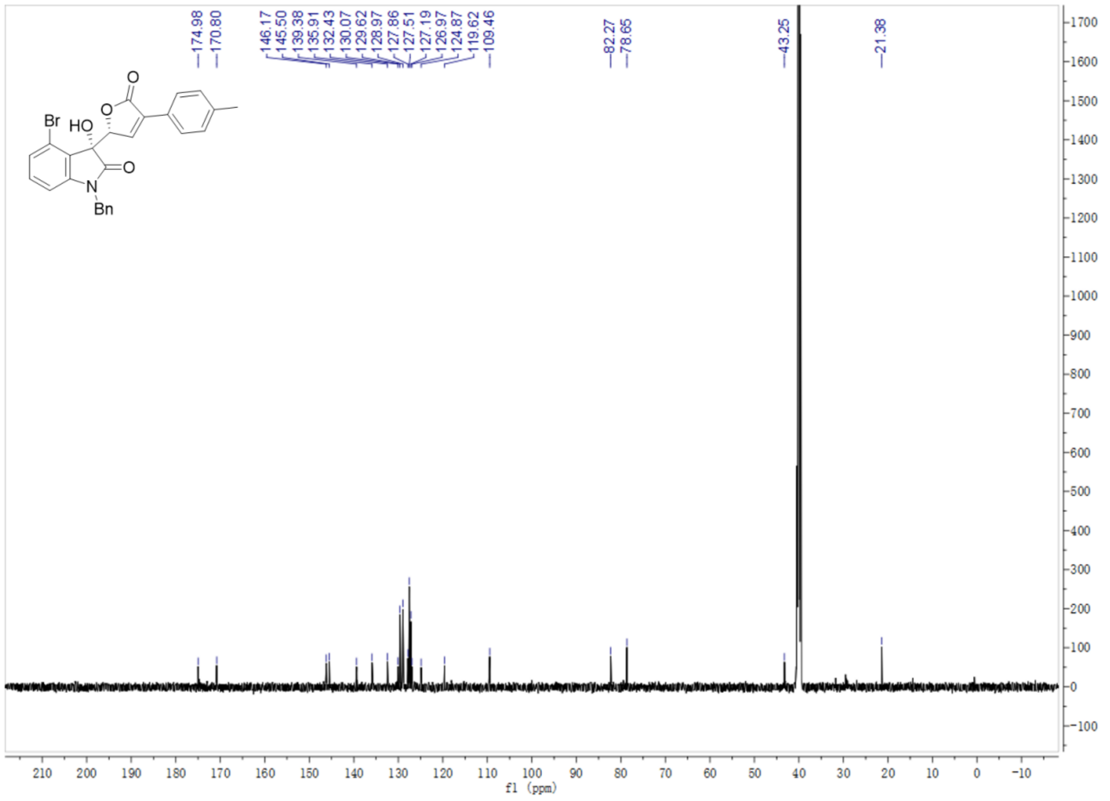

^{1}H-NMR and ^{13}C-NMR for **3ad:**

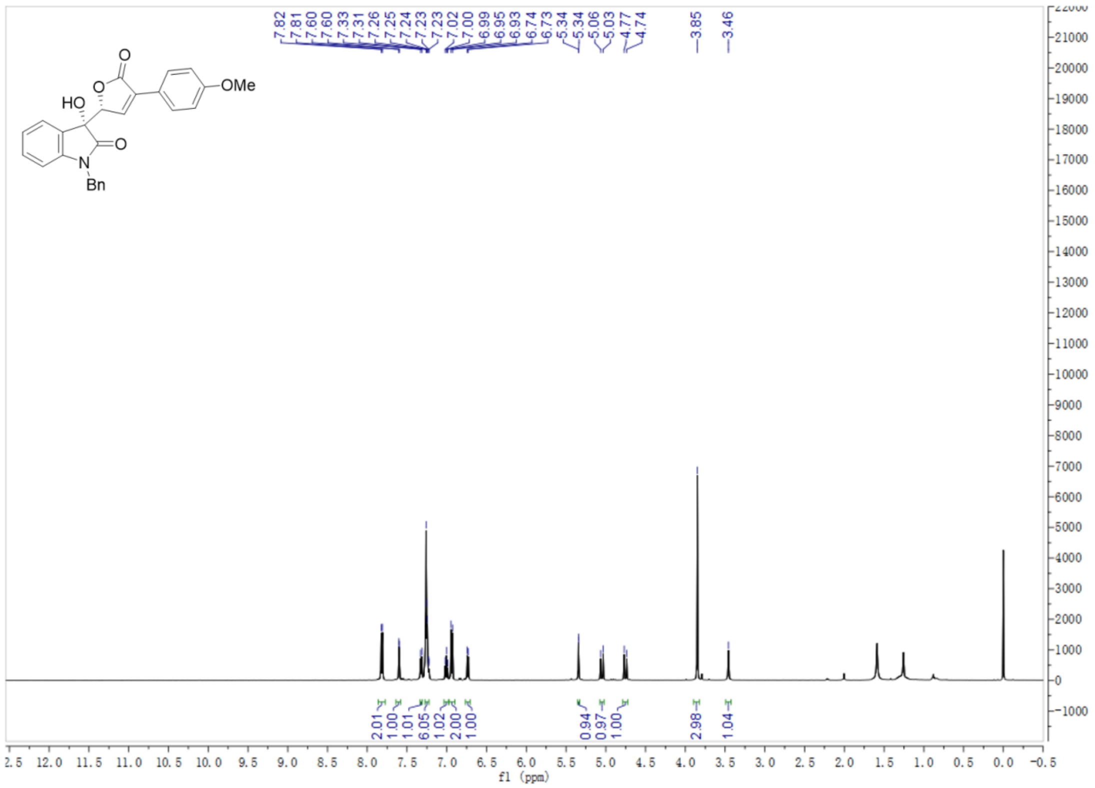

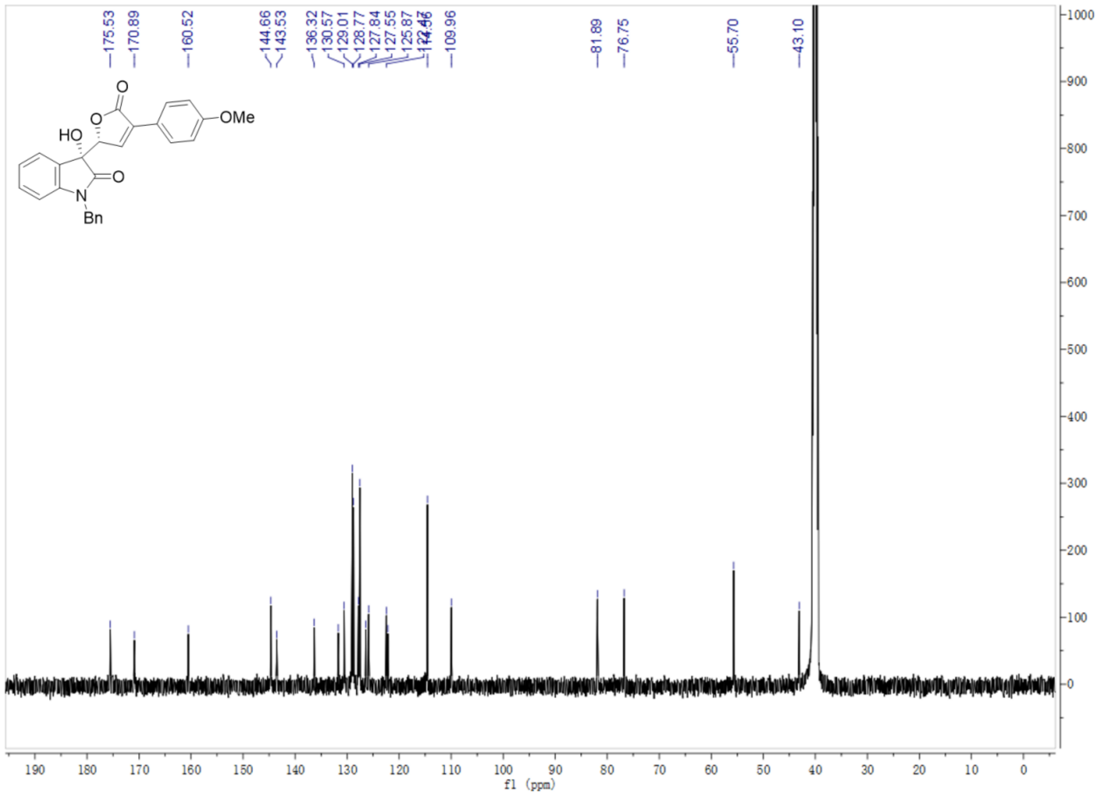

^{1}H-NMR and ^{13}C-NMR for **3ae:**

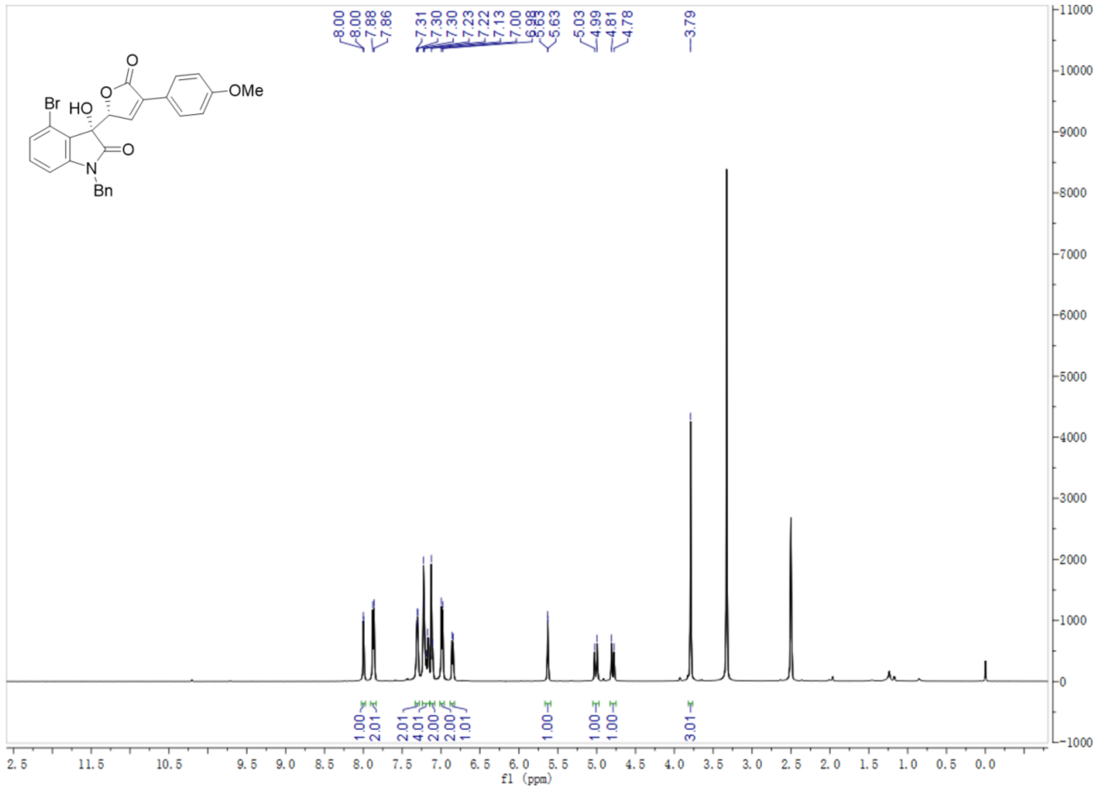

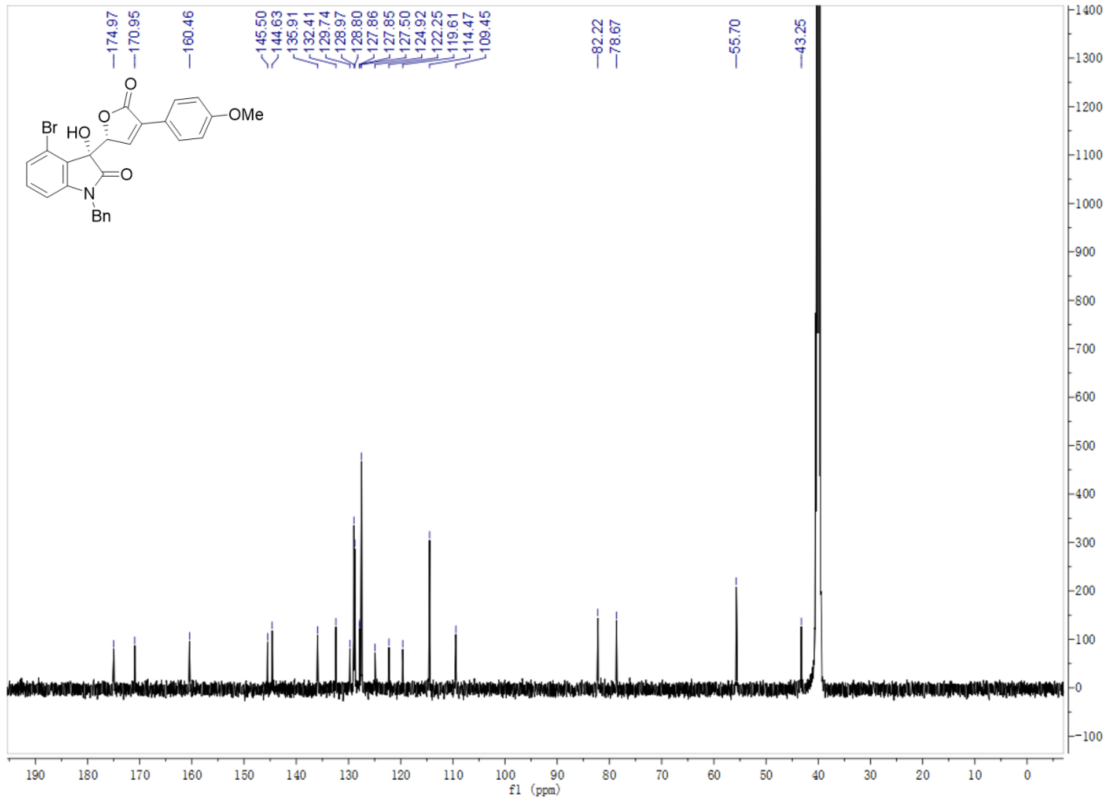

^{1}H-NMR and ^{13}C-NMR for **3af:**

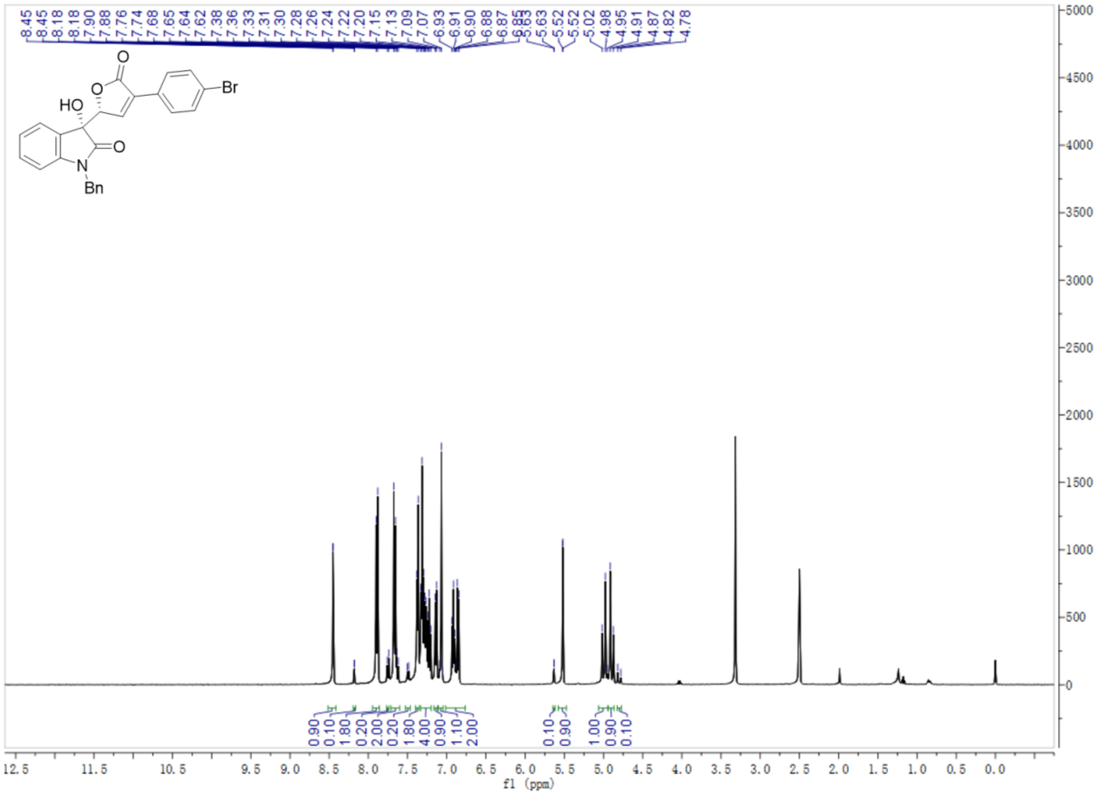

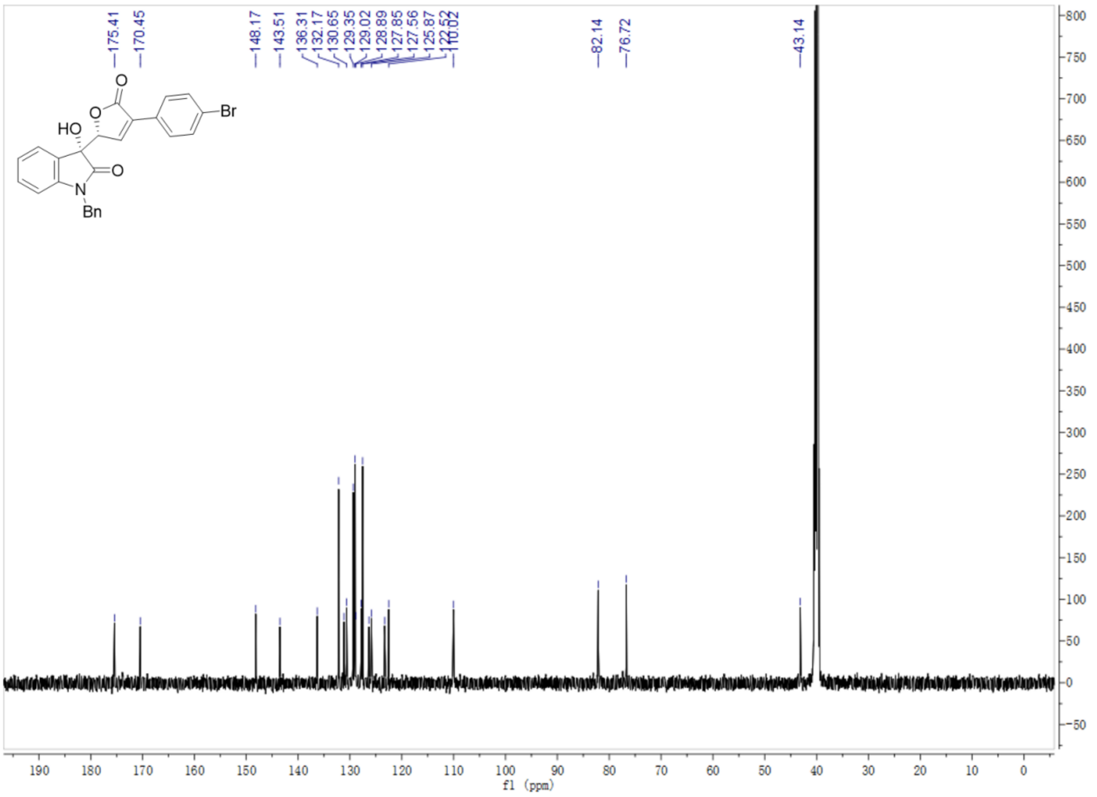

^{1}H-NMR, ^{13}C-NMR and [19] F NMR for **3ag:**

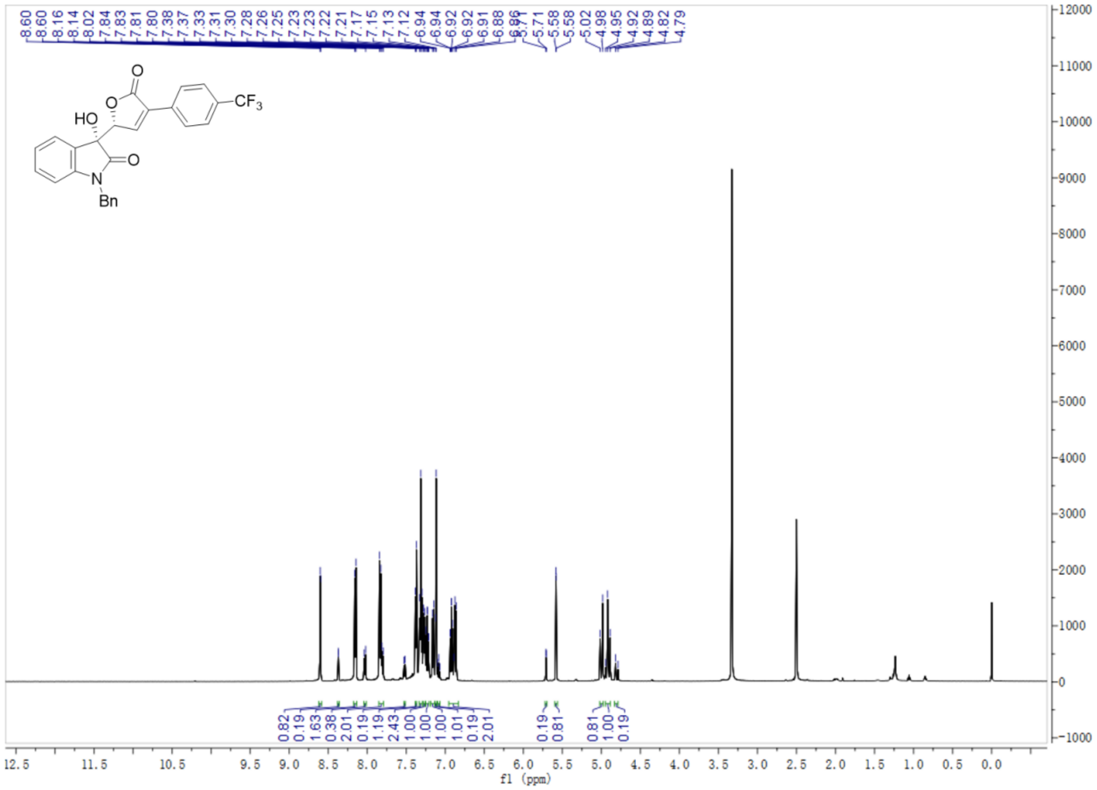

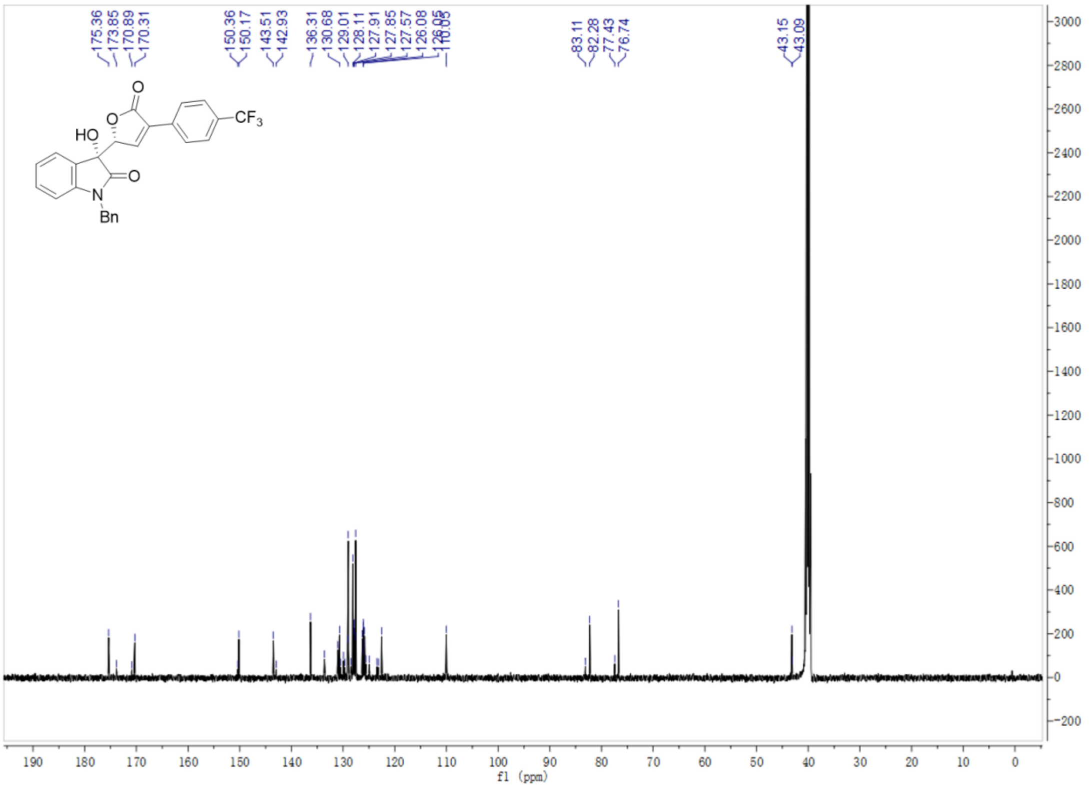

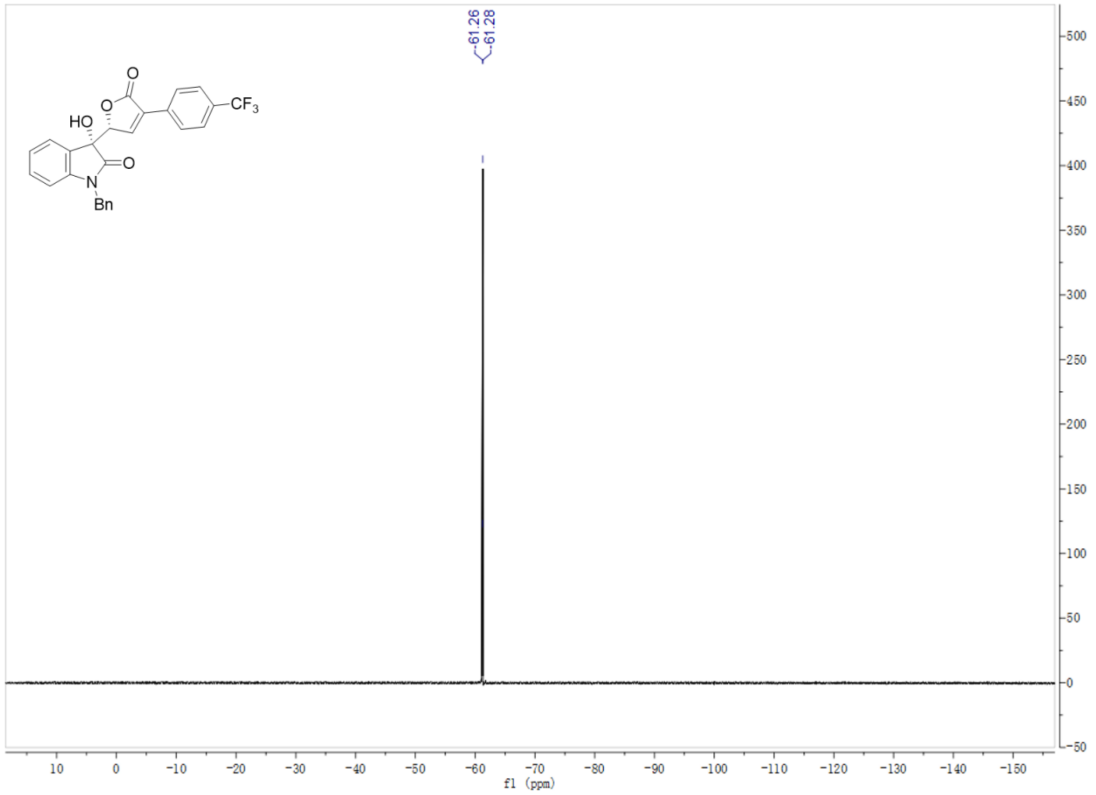
-61.26
-61.28
HO
O
O
O
CF3
N
Bn
f1 (ppm)

^{1}H-NMR and ^{13}C-NMR for **3ah:**

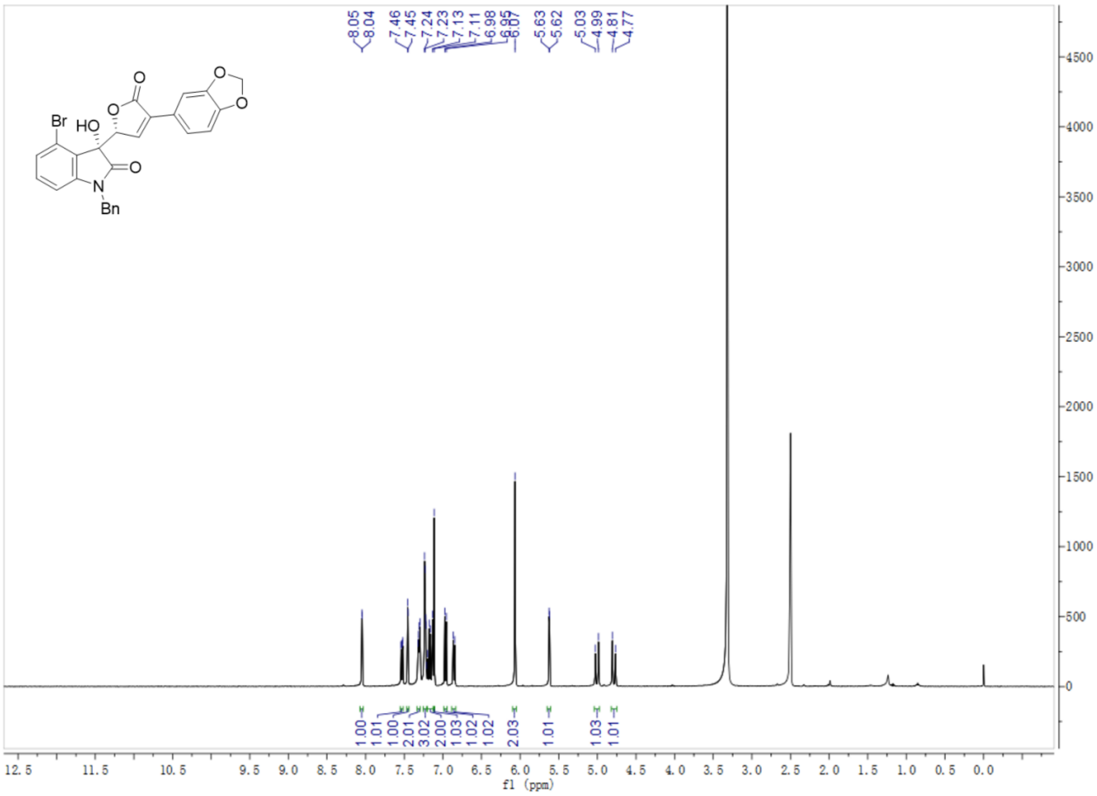

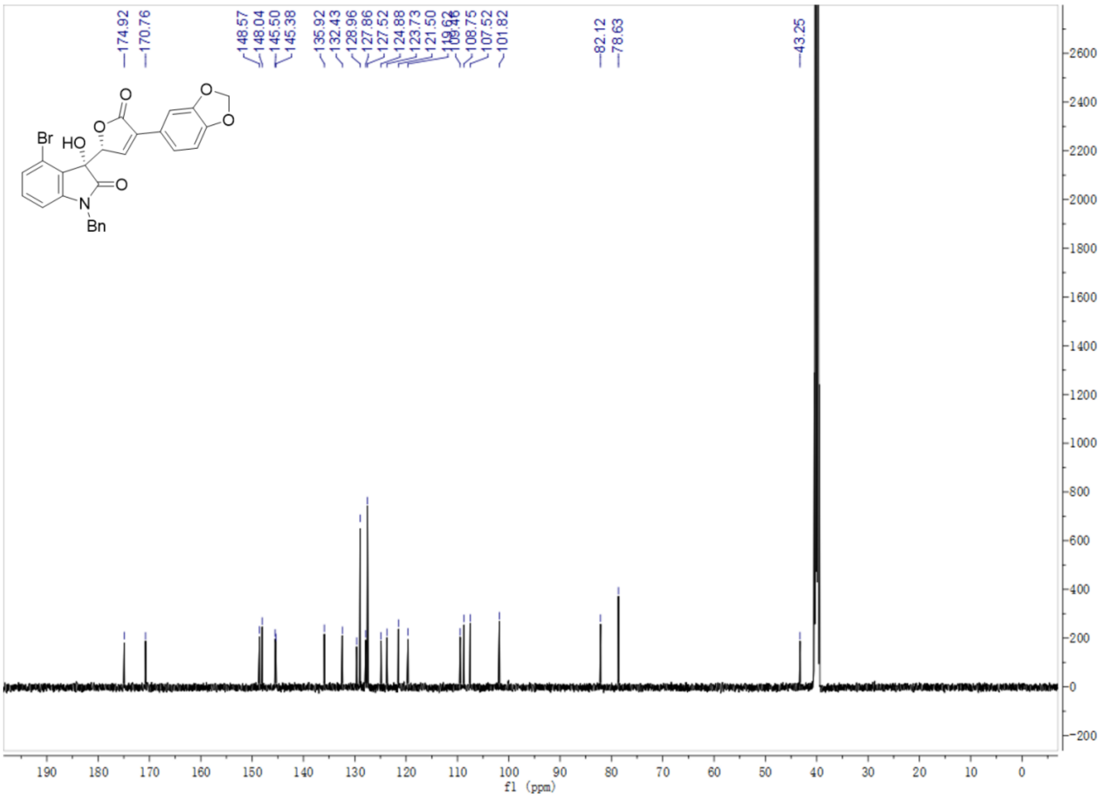

^{1}H-NMR and ^{13}C-NMR for **3ai:**

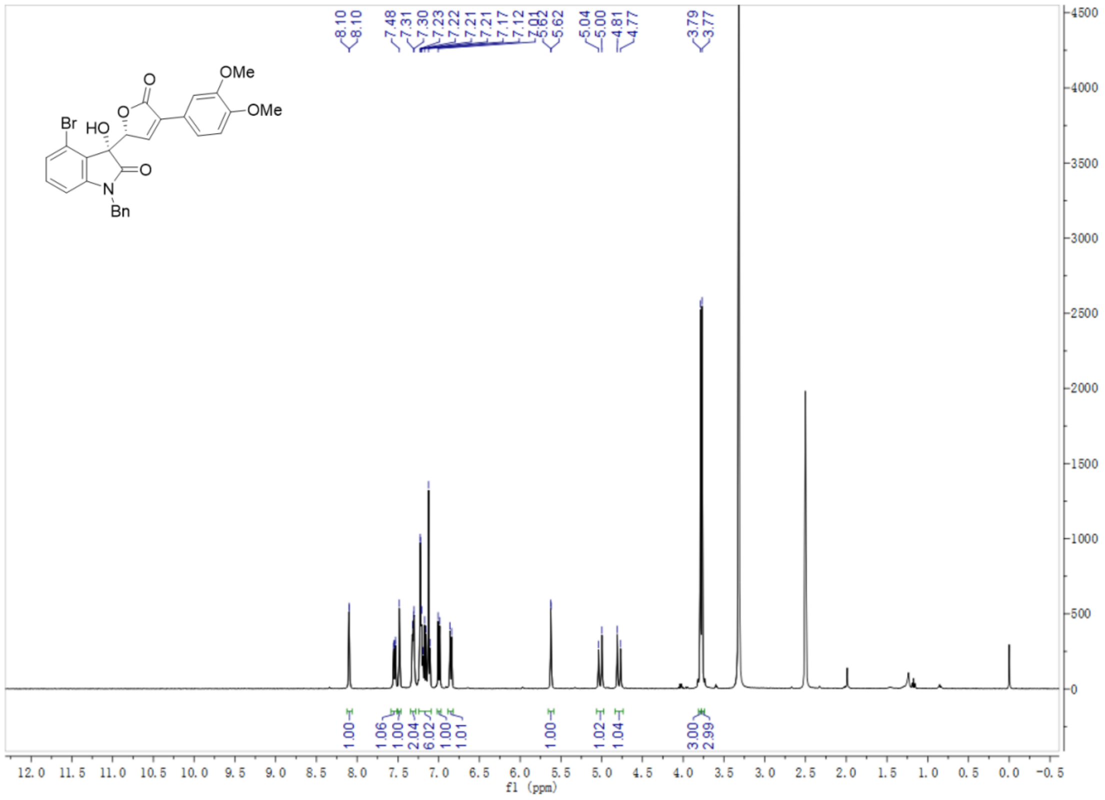

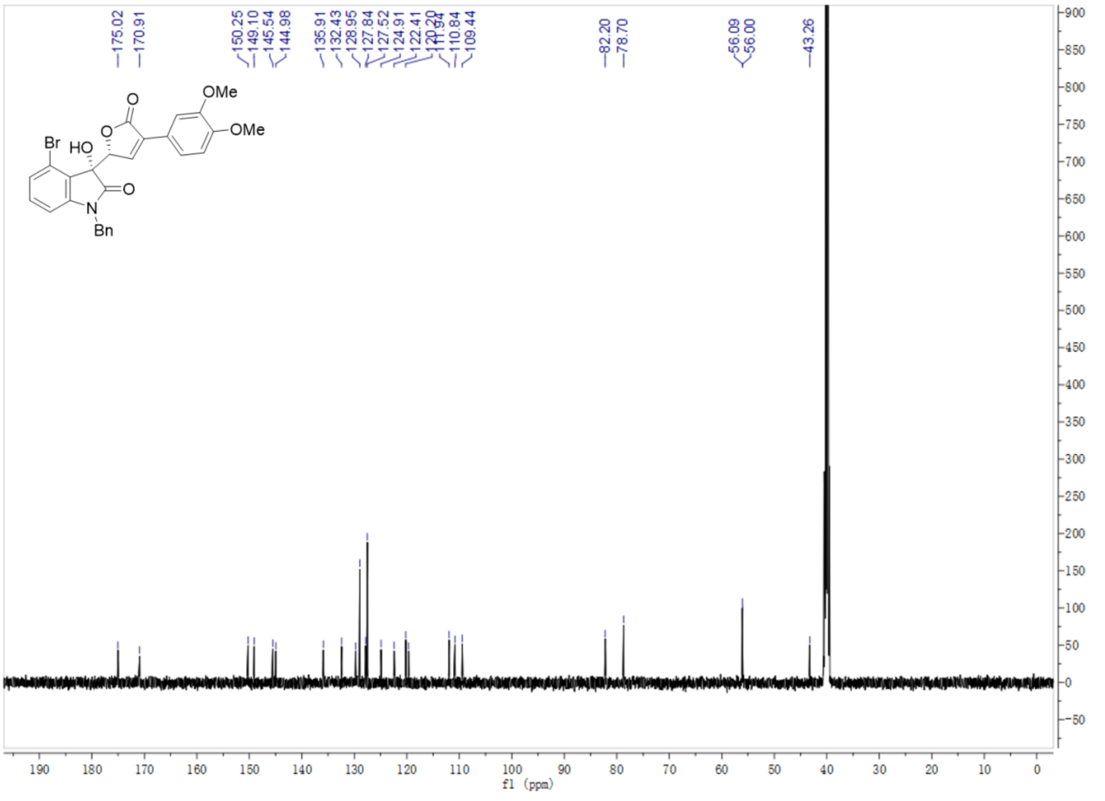

^{1}H-NMR and ^{13}C-NMR for **3aj:**

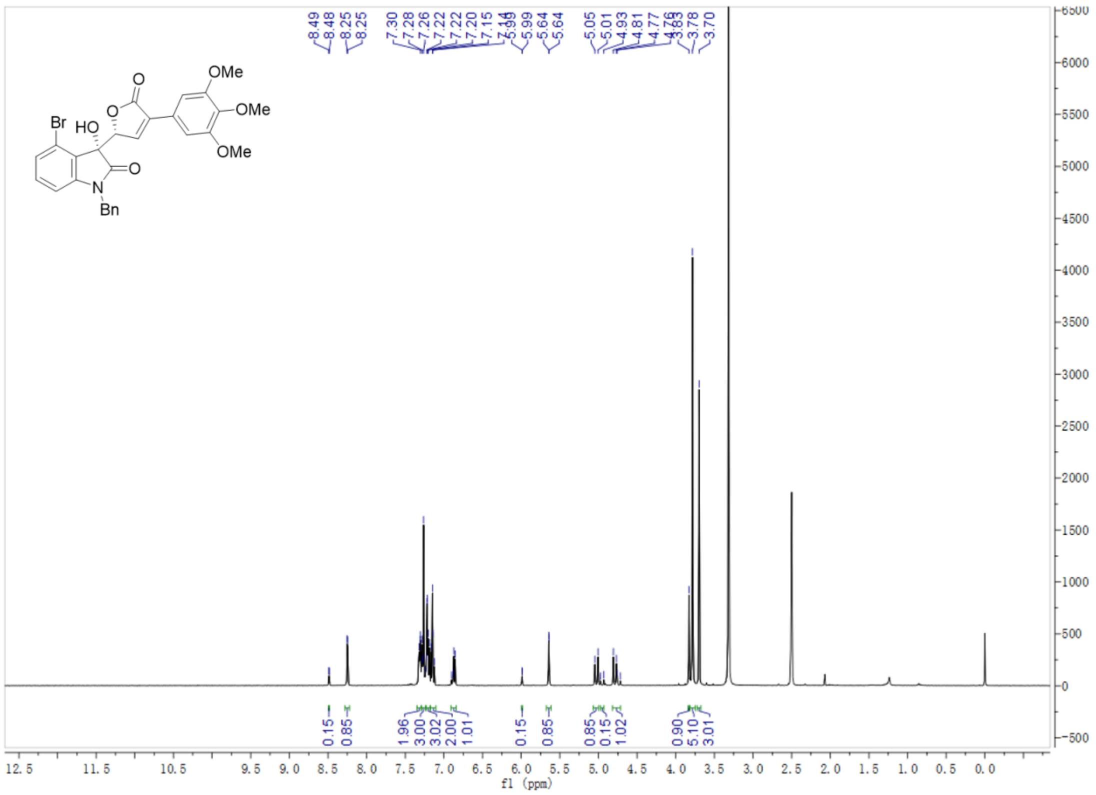

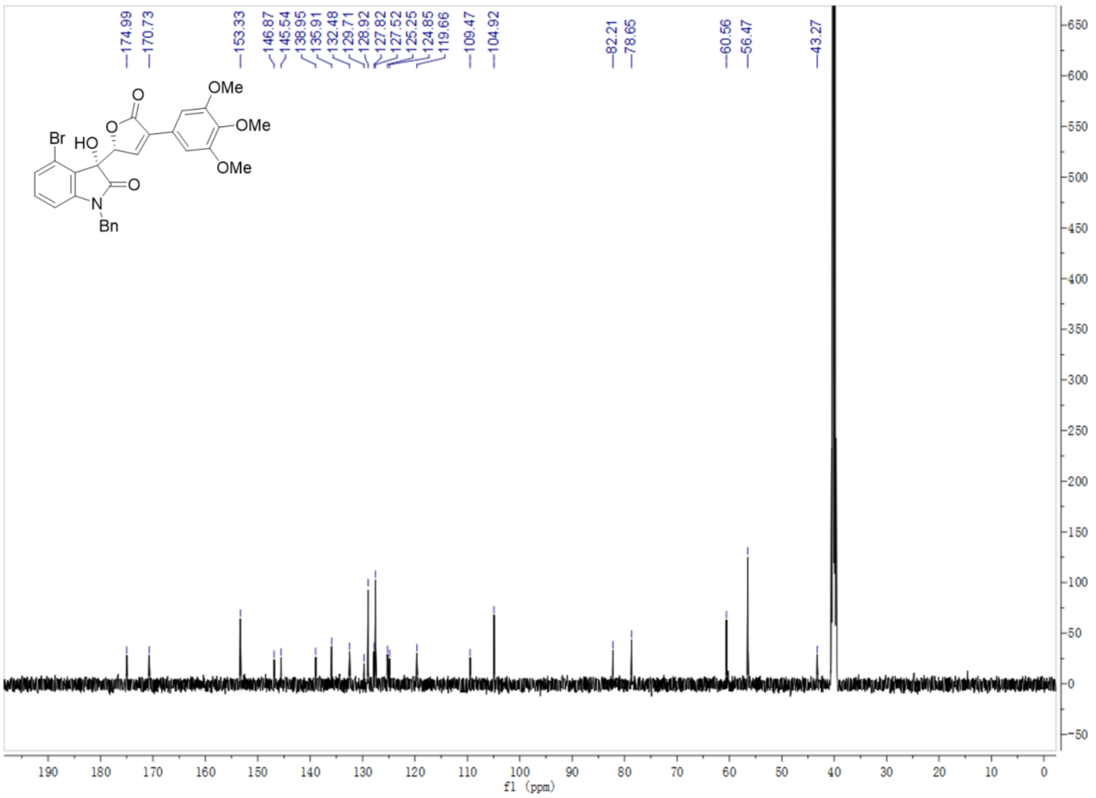

^{1}H-NMR and ^{13}C-NMR for **3ak:**

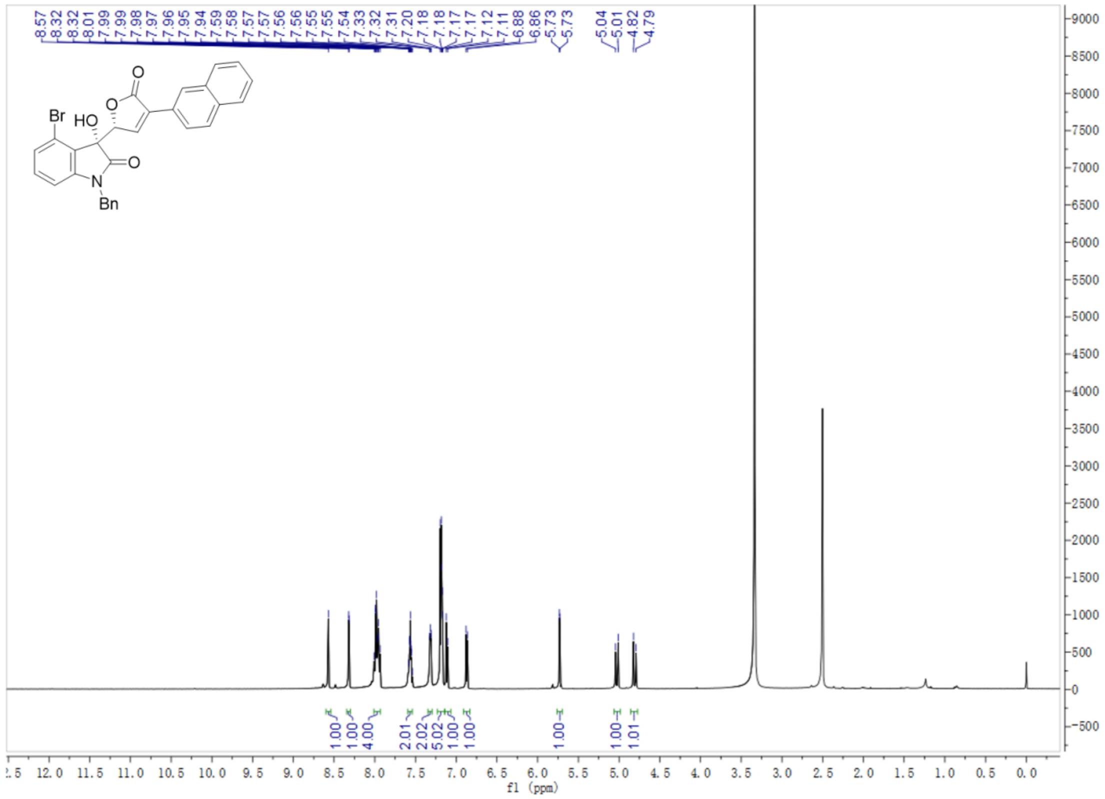

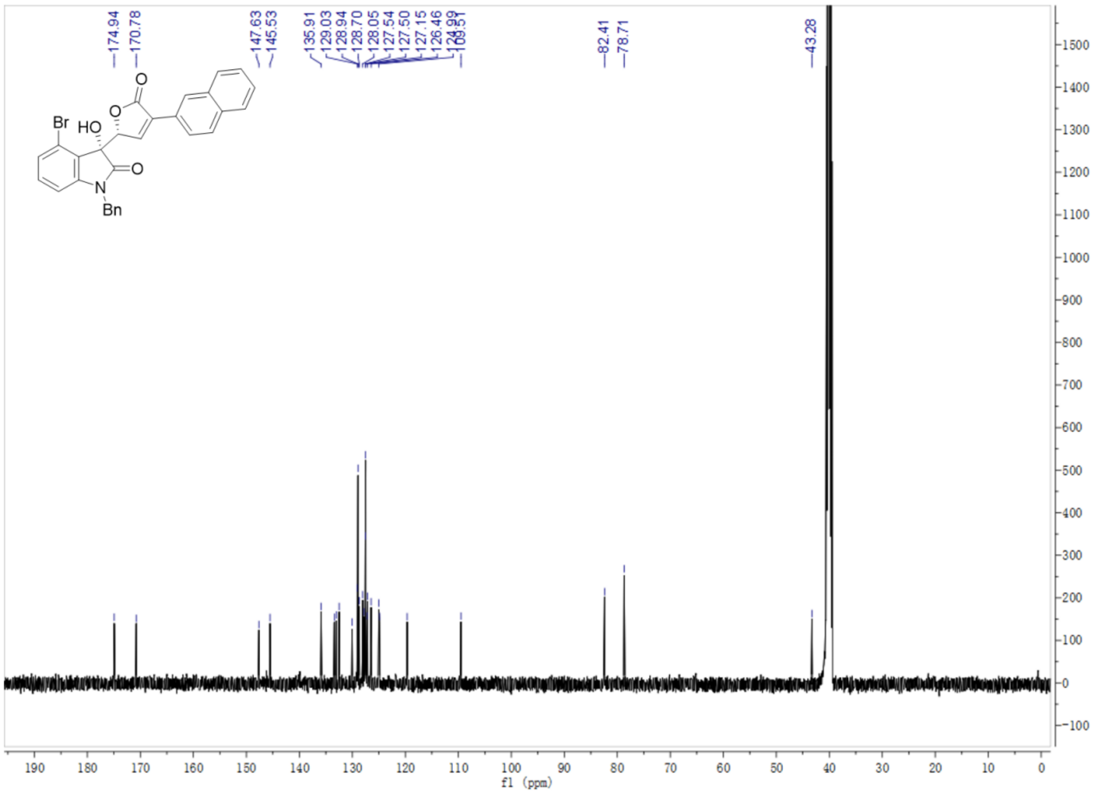

^{1}H-NMR and ^{13}C-NMR for **3al:**

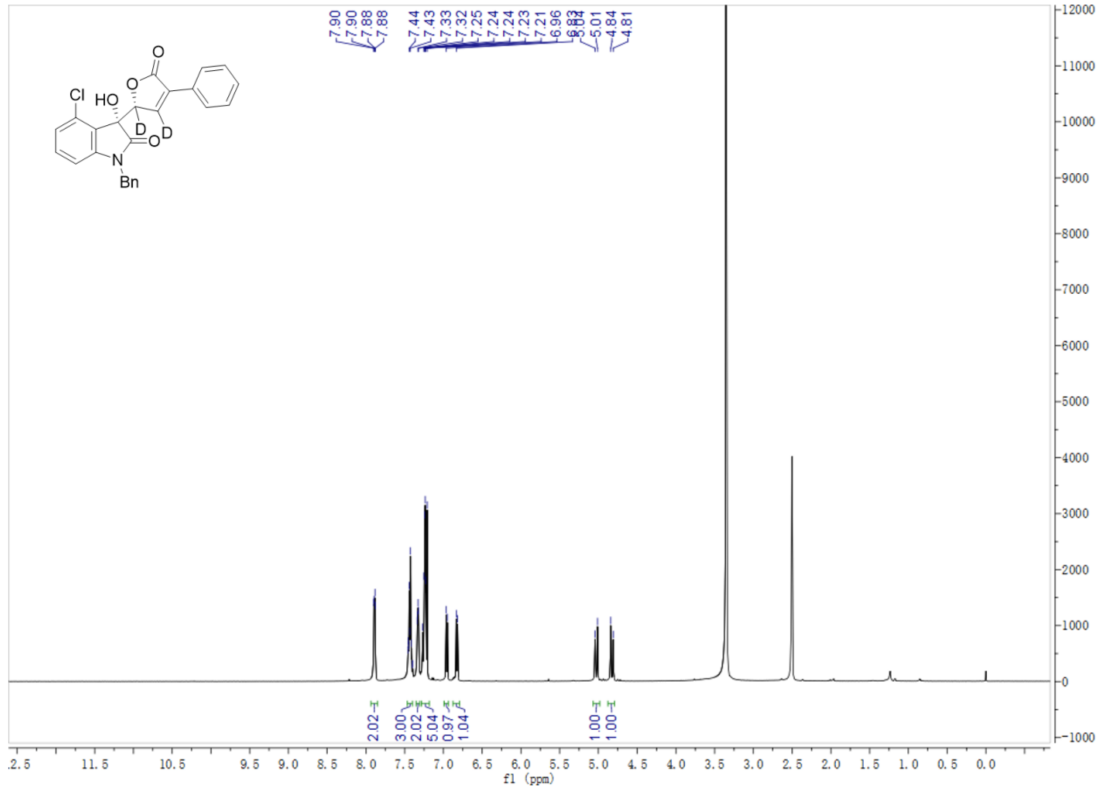

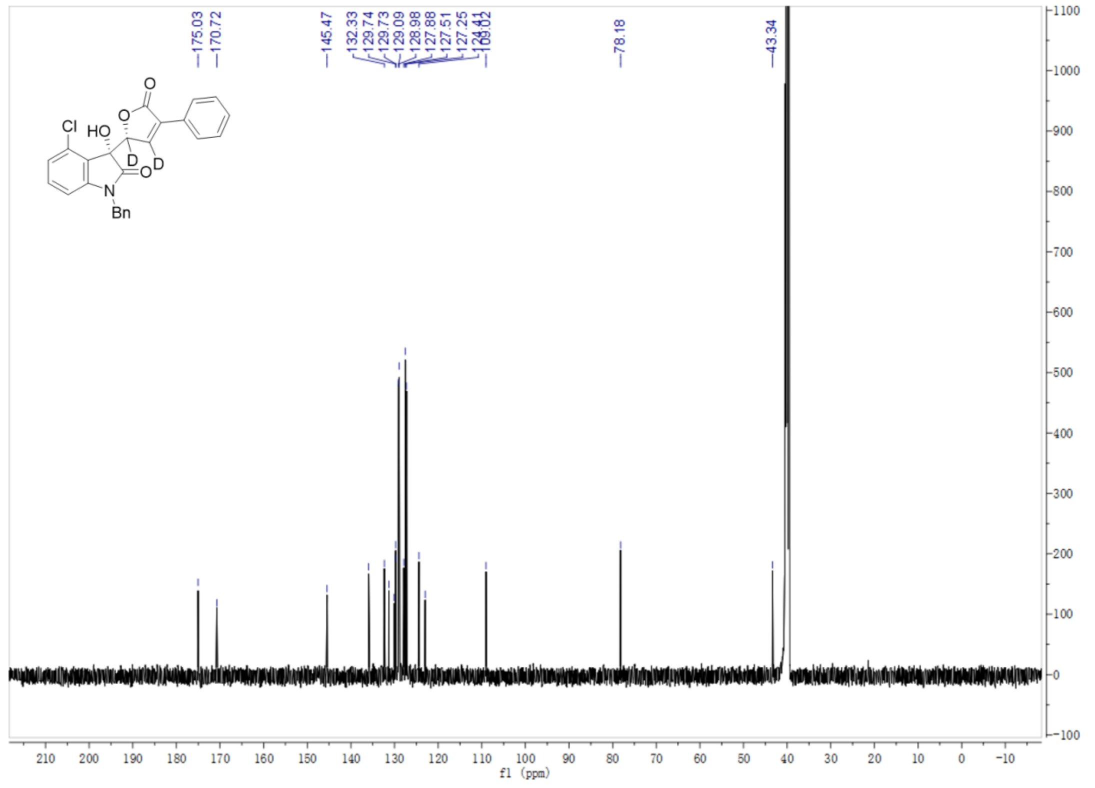

^{1}H-NMR and ^{13}C-NMR for **3am:**

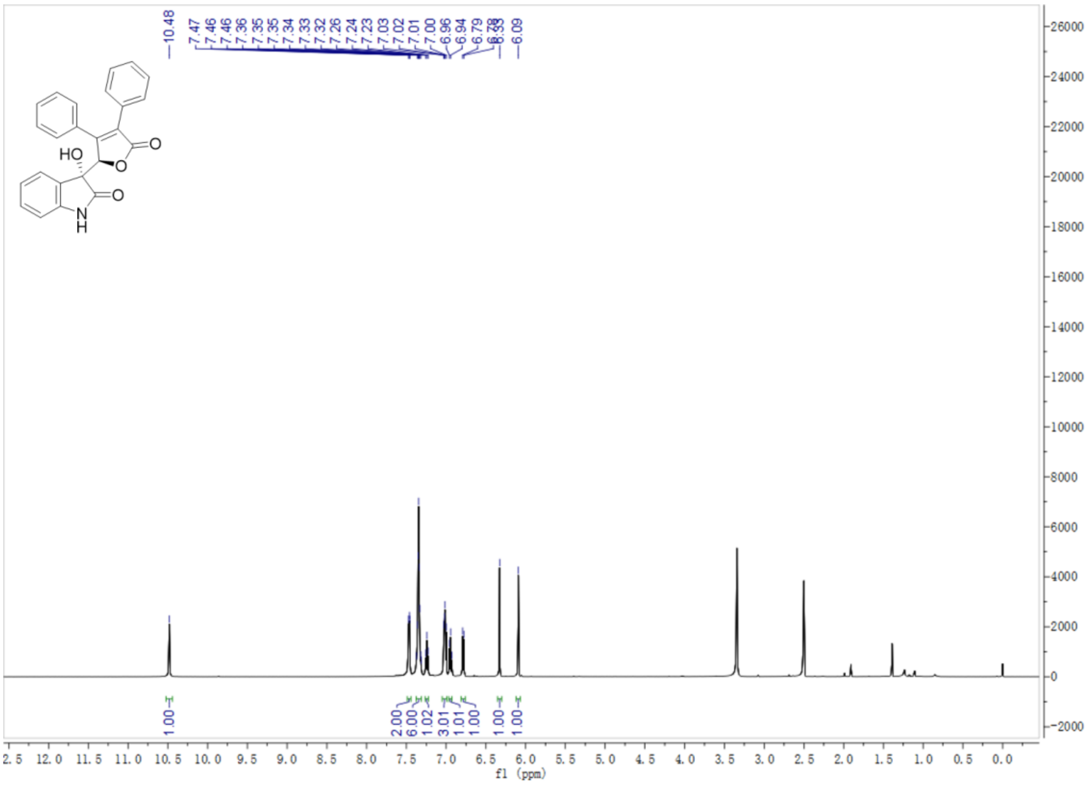

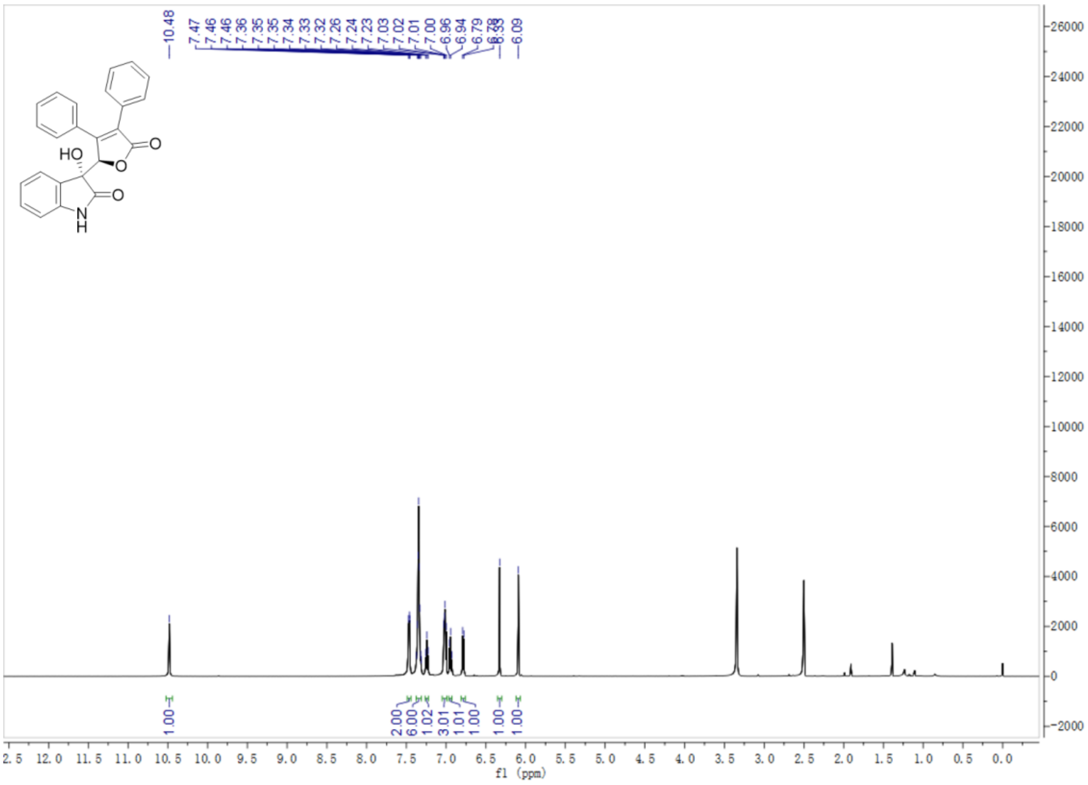

^{1}H-NMR and ^{13}C-NMR for **3an:**

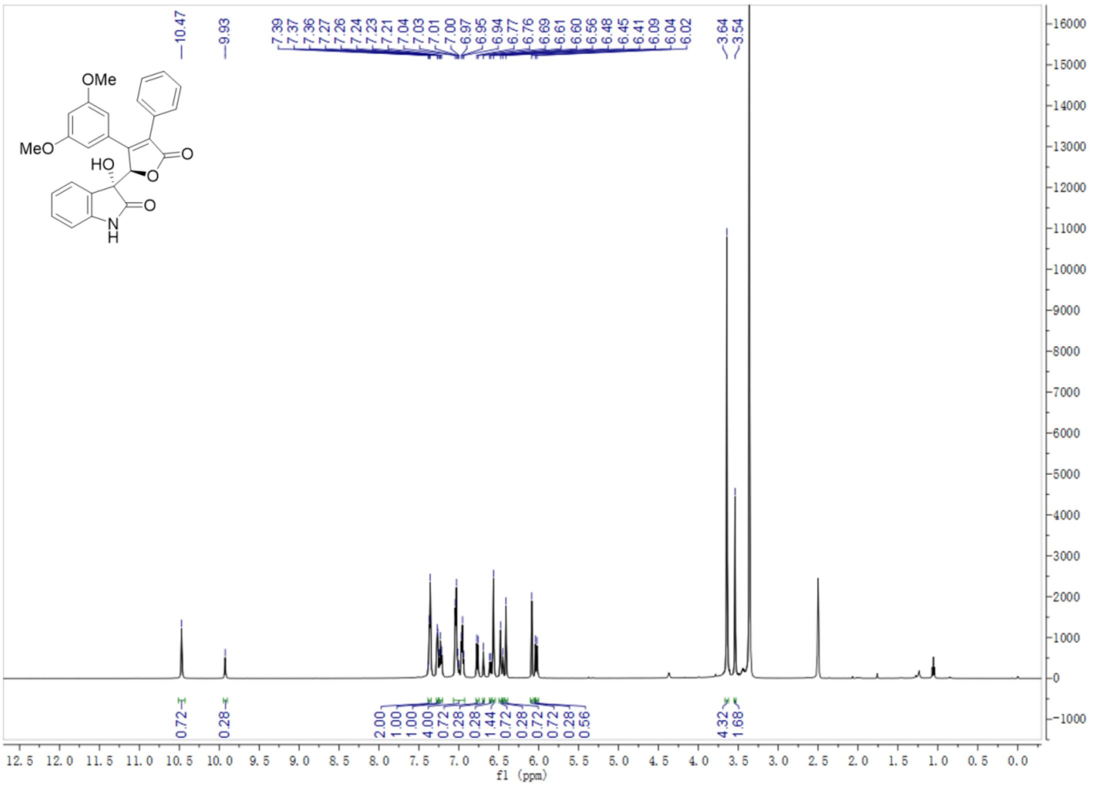

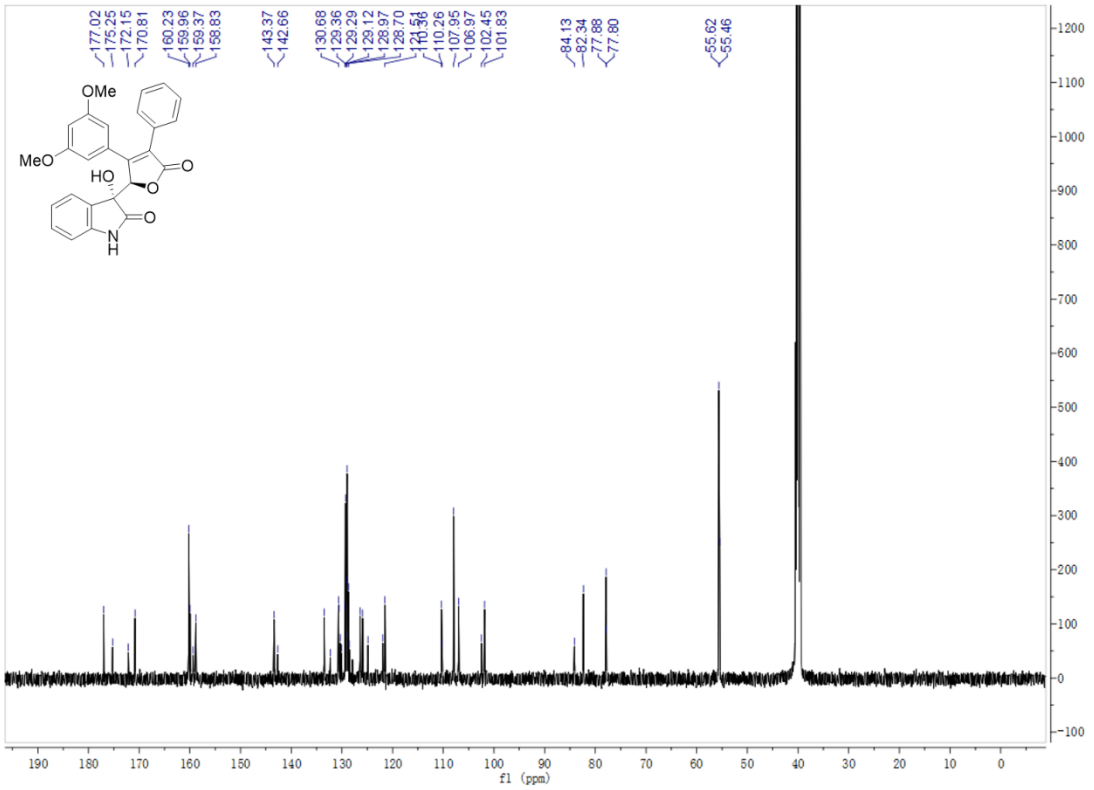

^{1}H-NMR and ^{13}C-NMR for **3ao:**

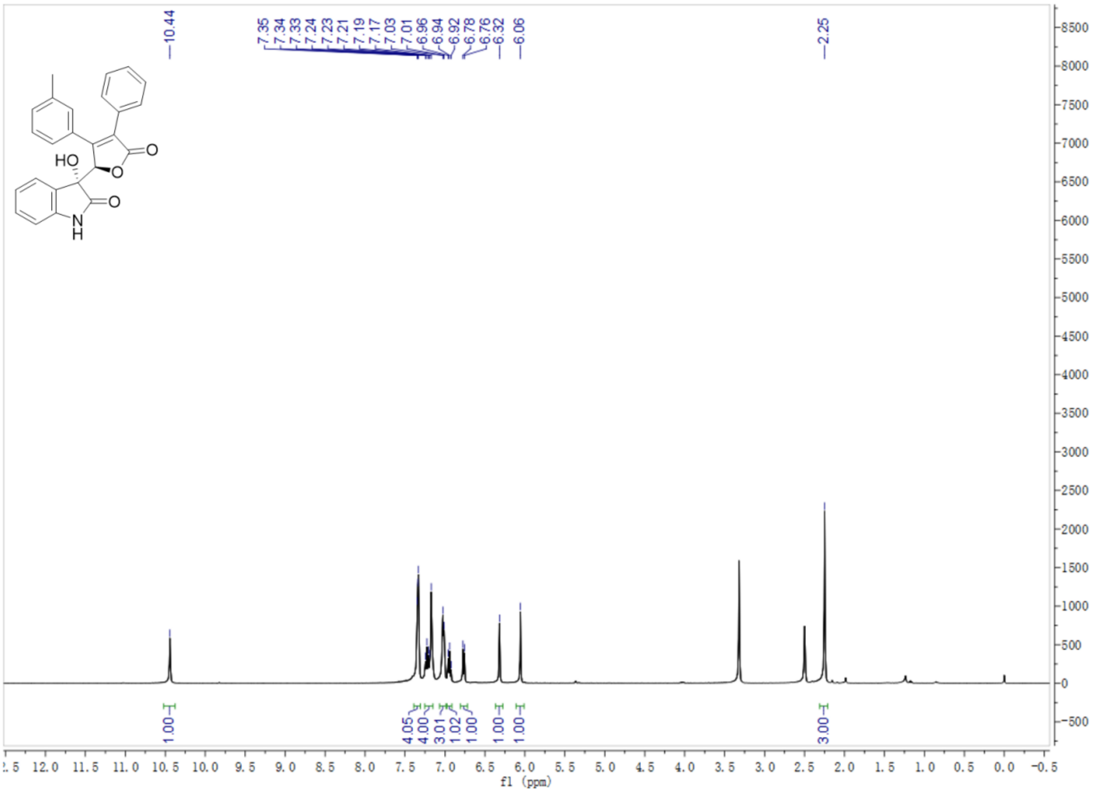

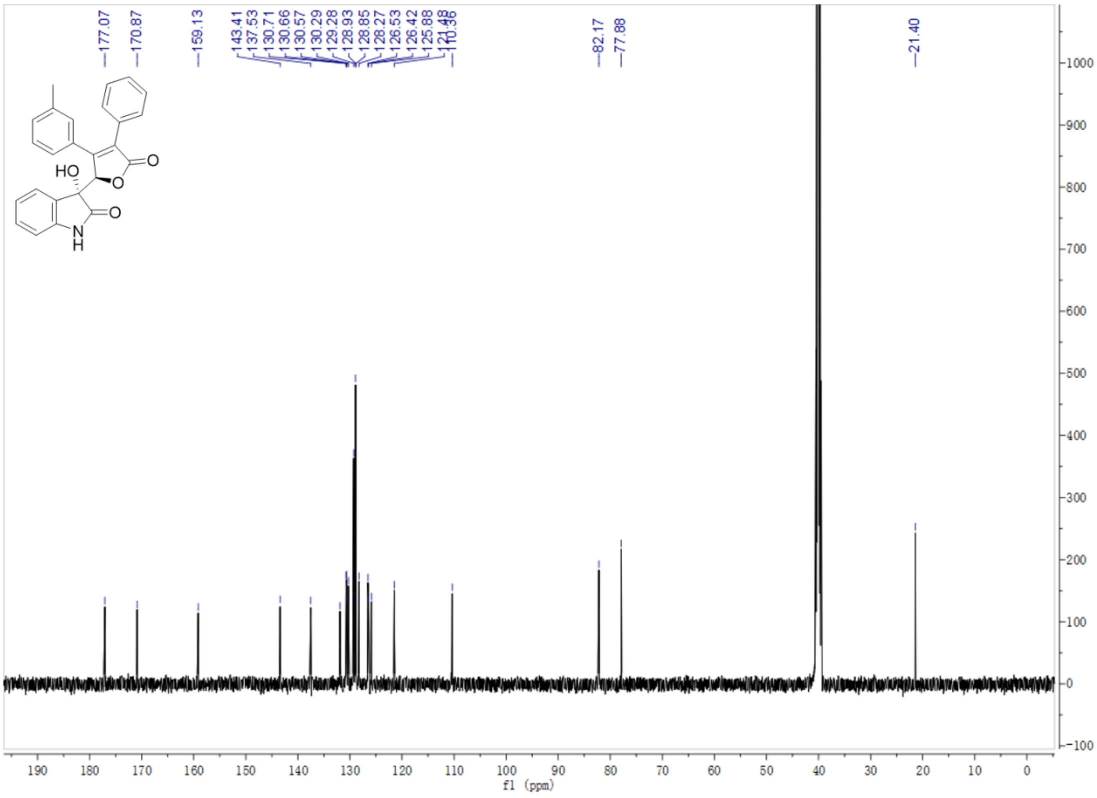

^{1}H-NMR and ^{13}C-NMR for **3ap:**

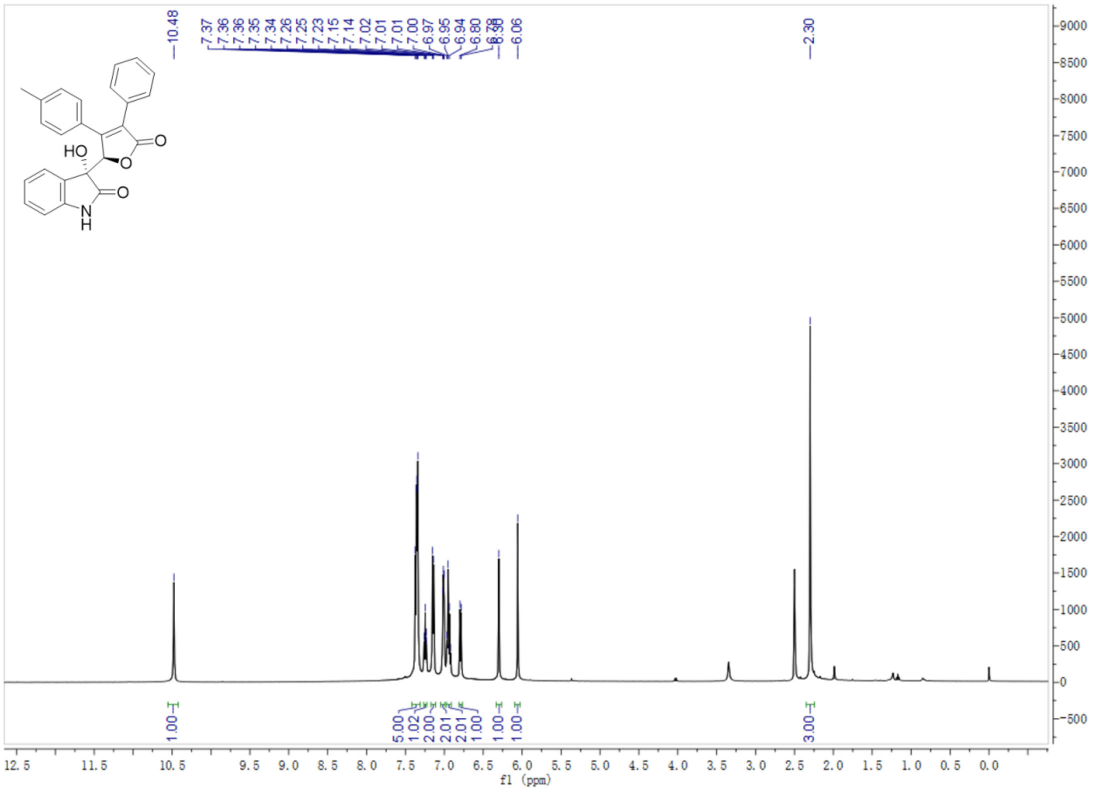

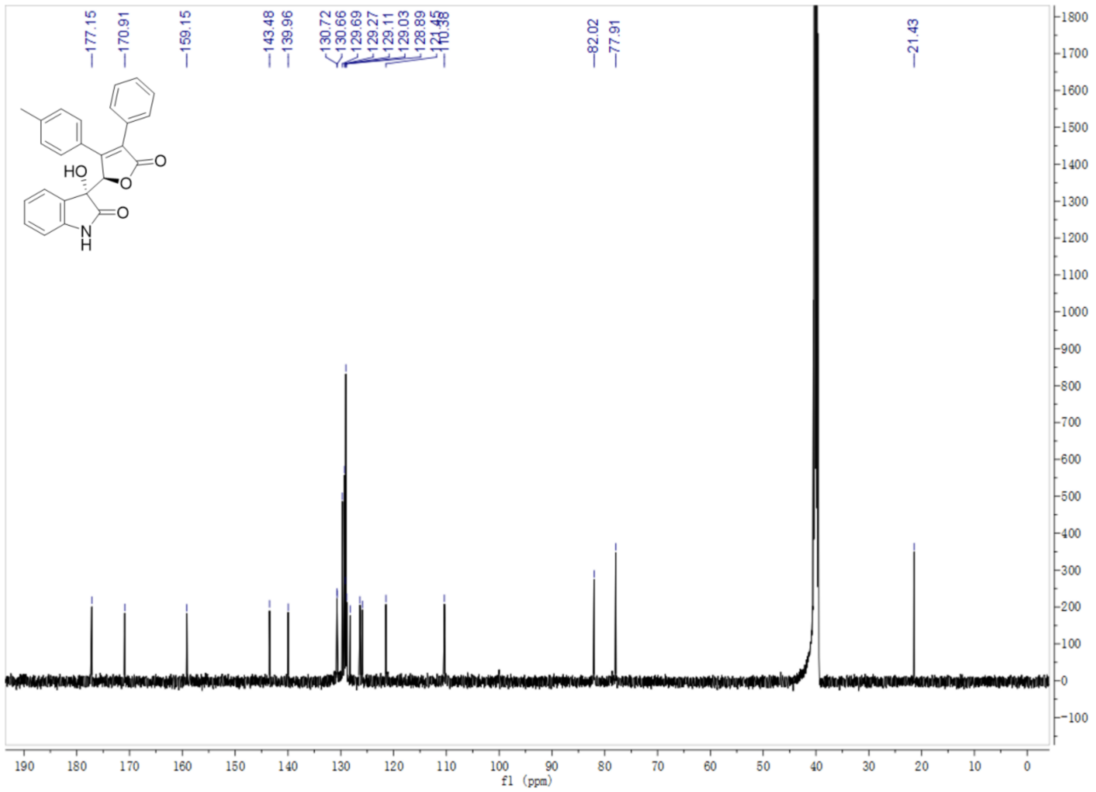

^{1}H-NMR and ^{13}C-NMR for **3aq:**

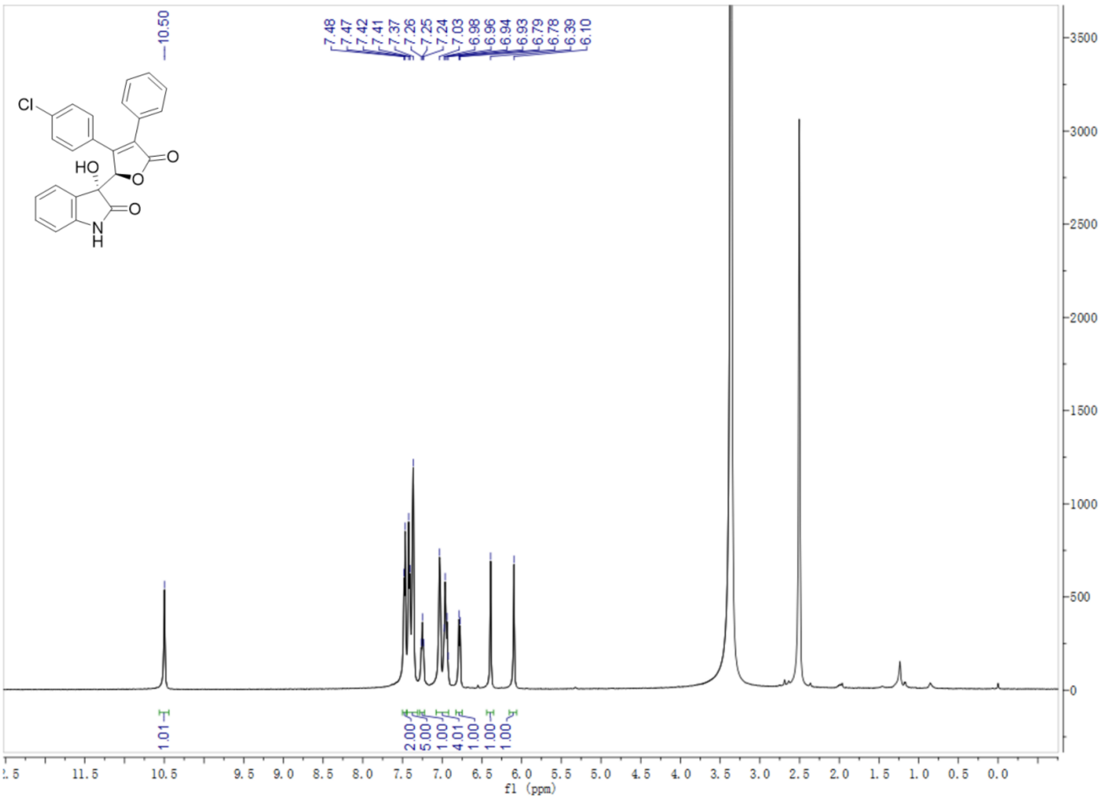

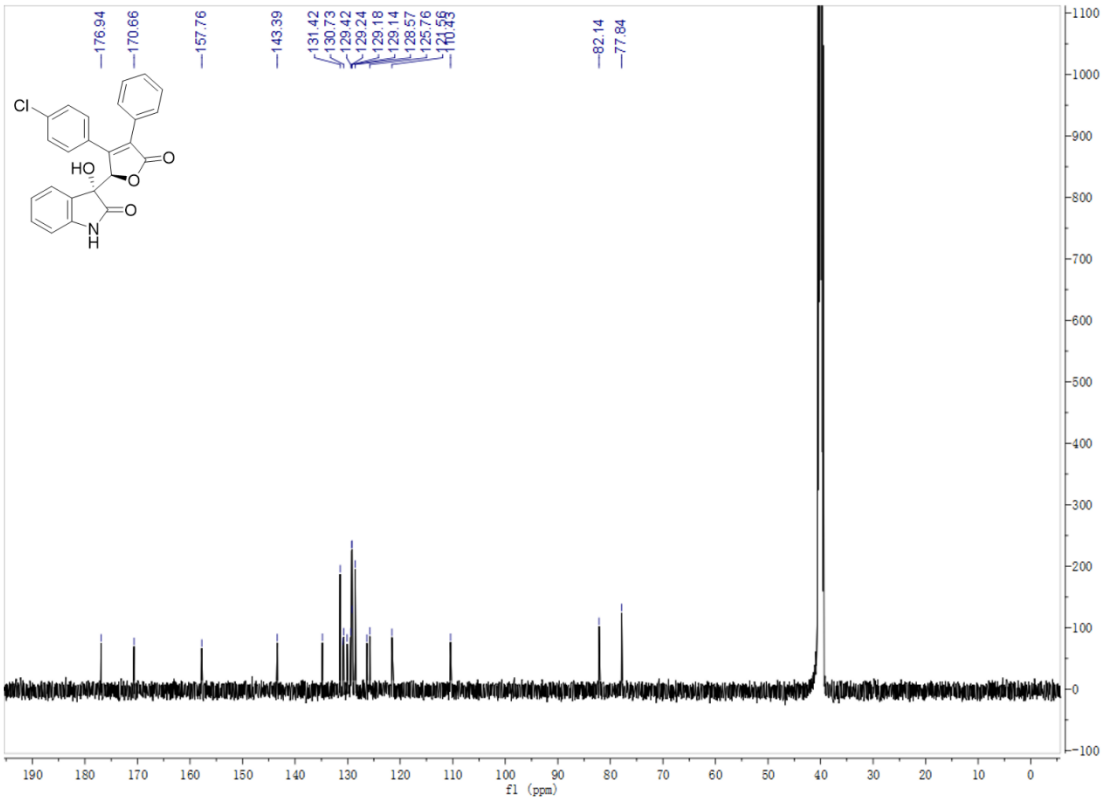

^{1}H-NMR and ^{13}C-NMR for **3ar:**

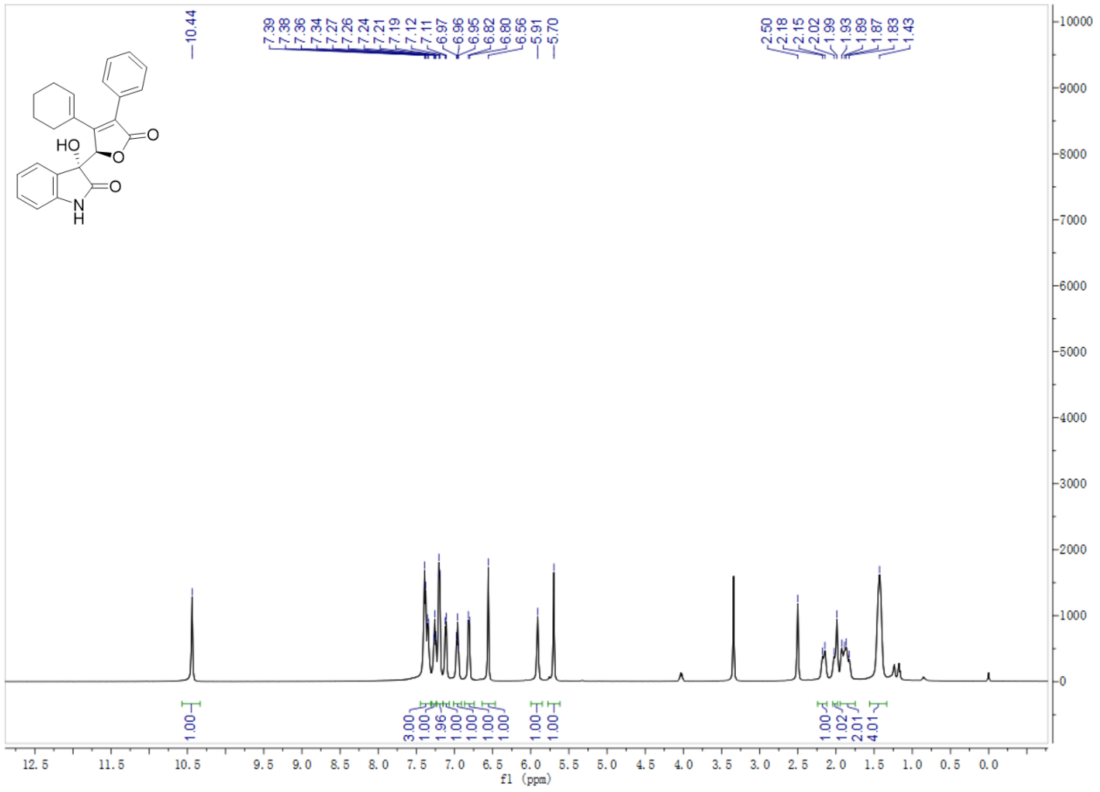

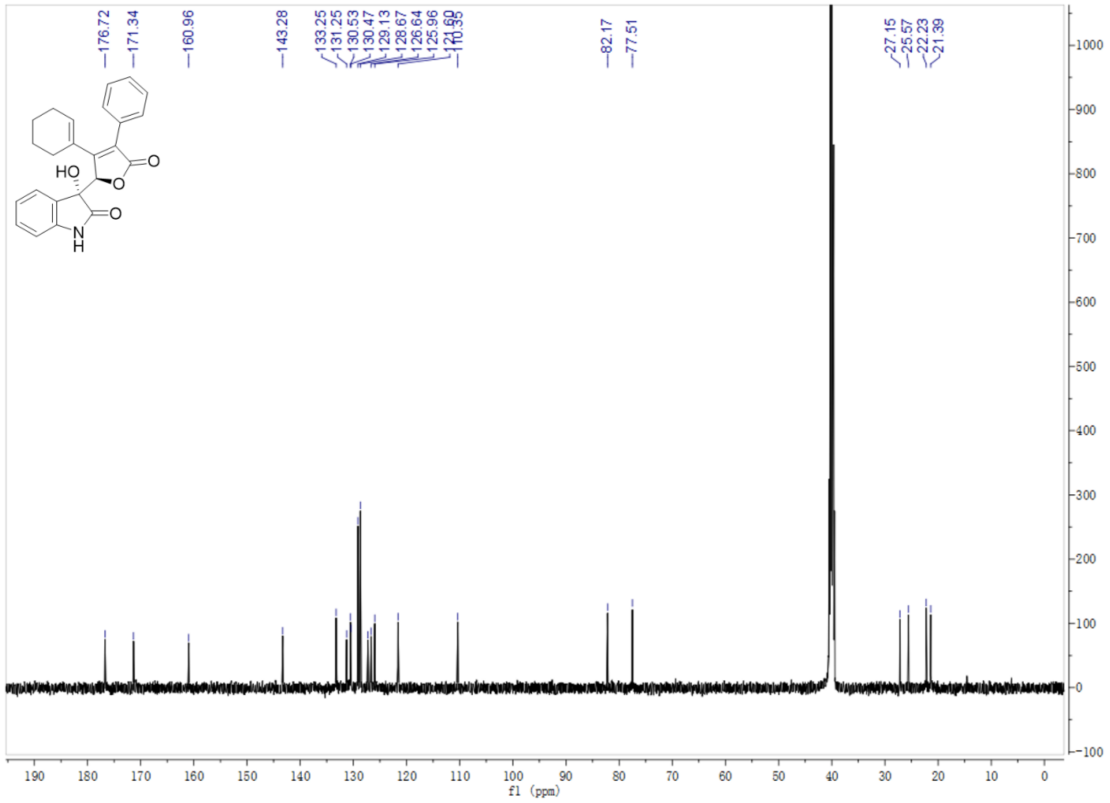

^{1}H-NMR and ^{13}C-NMR for **3as:**

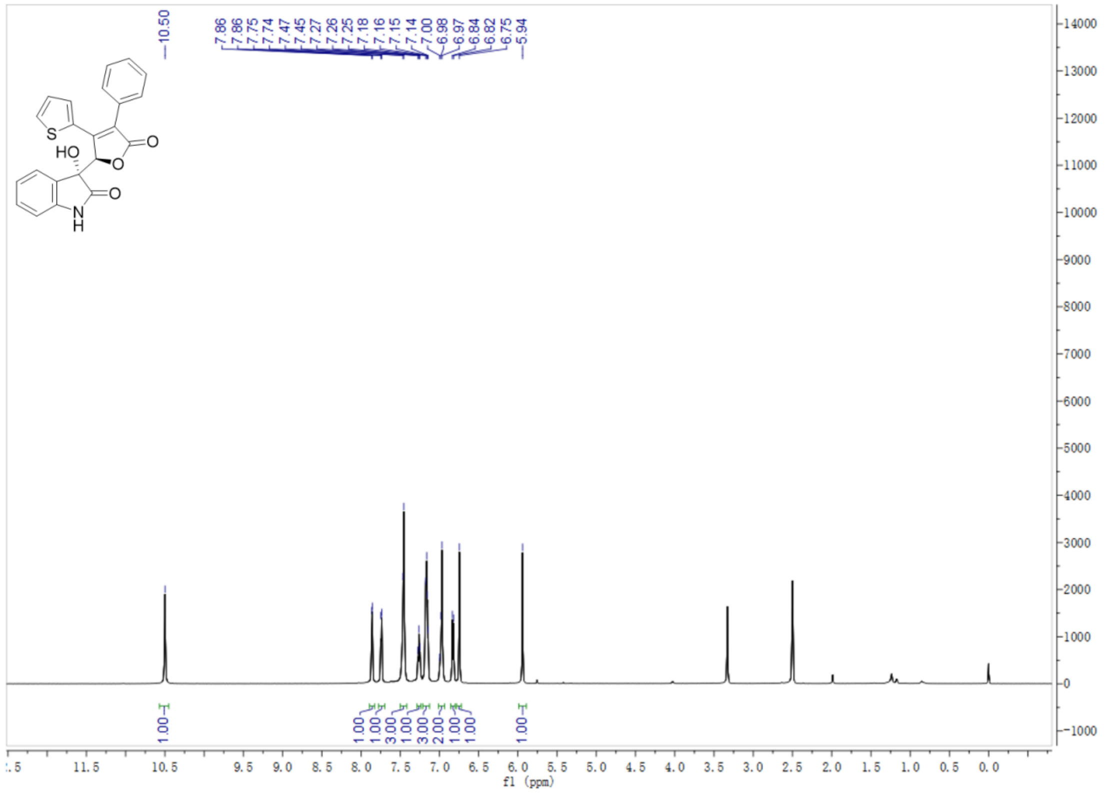

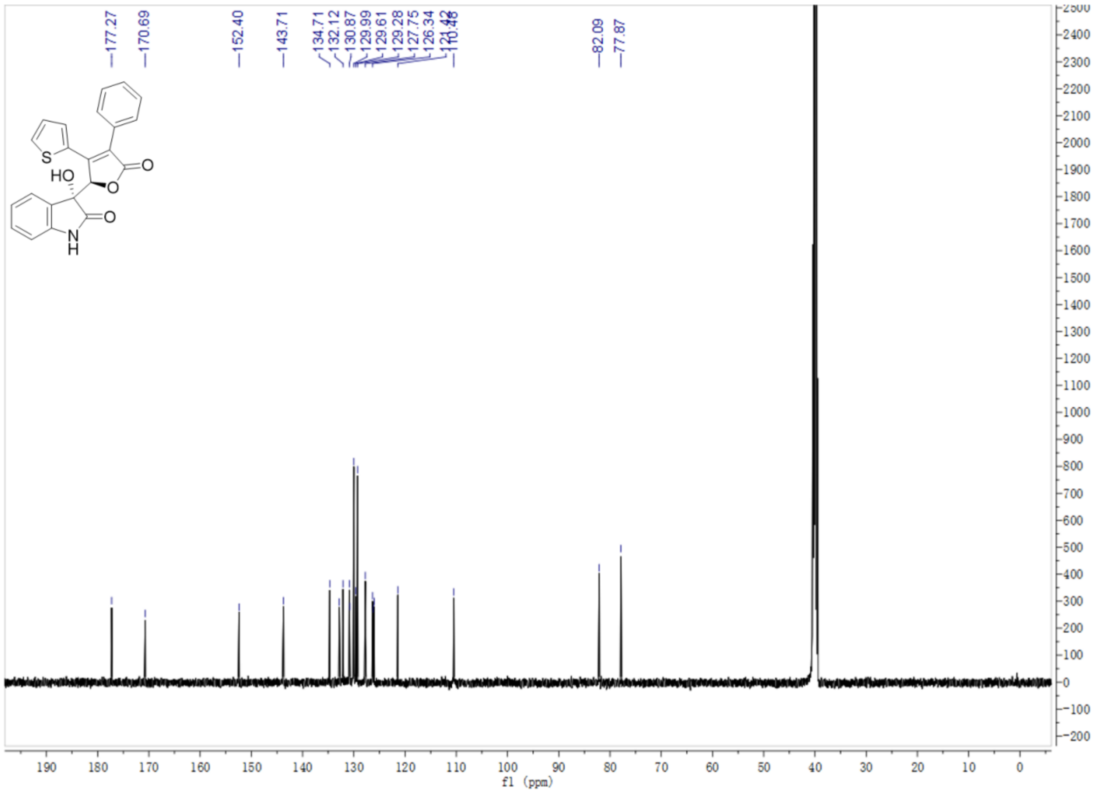

^{1}H-NMR and ^{13}C-NMR for **3at:**

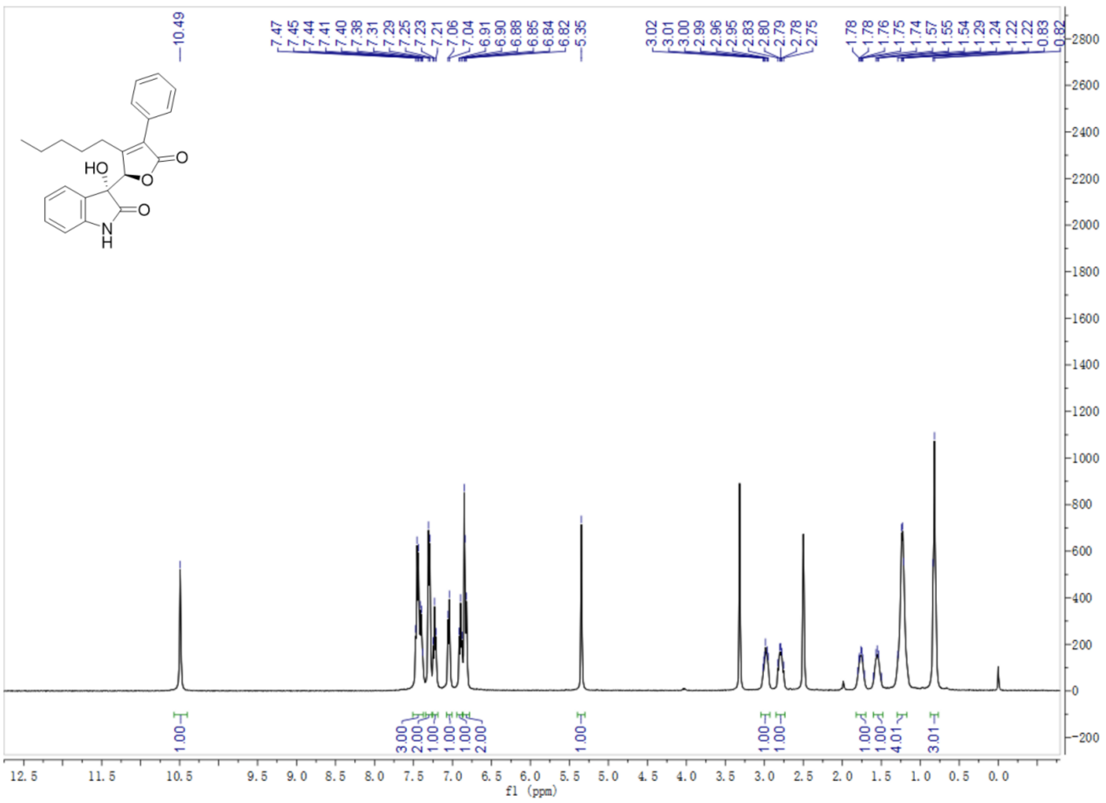

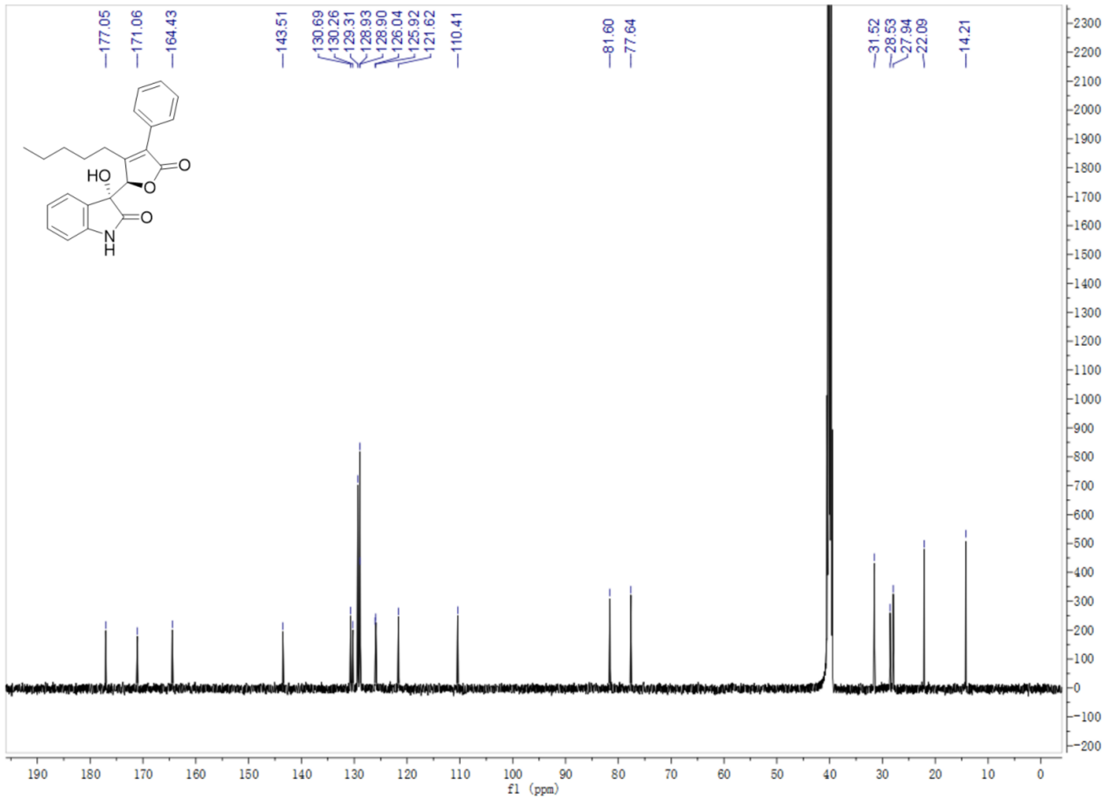

^{1}H-NMR and ^{13}C-NMR for **3au:**

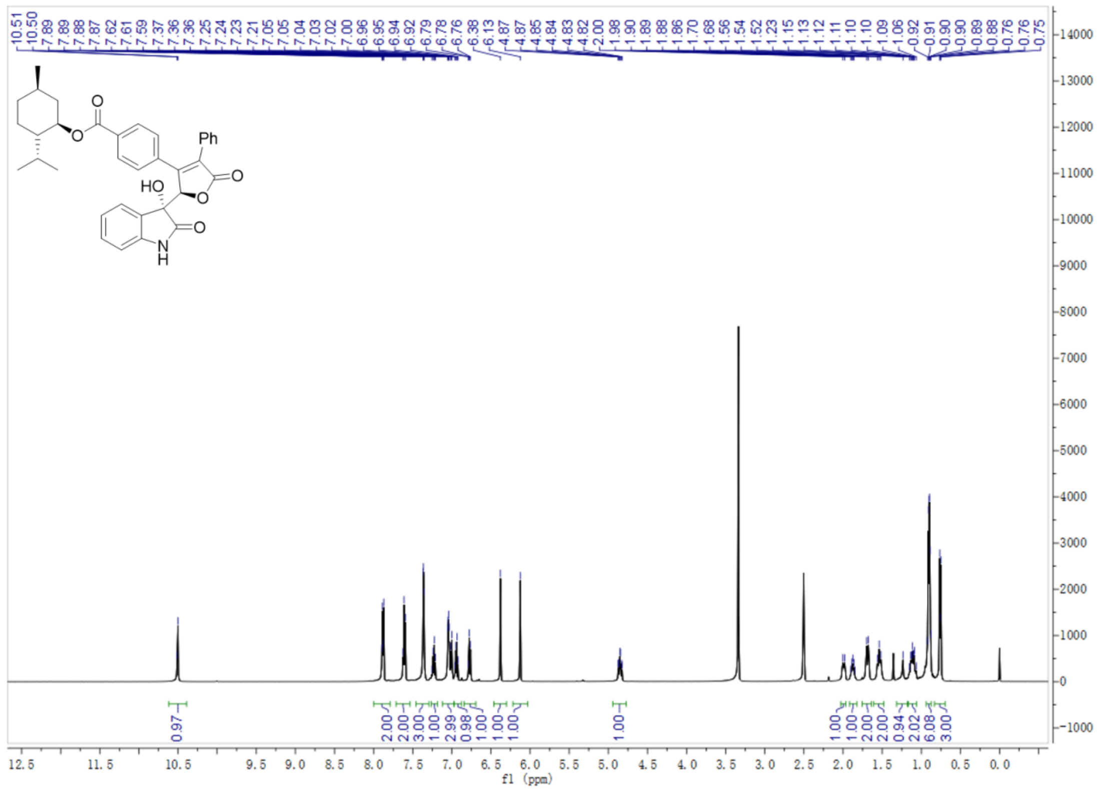

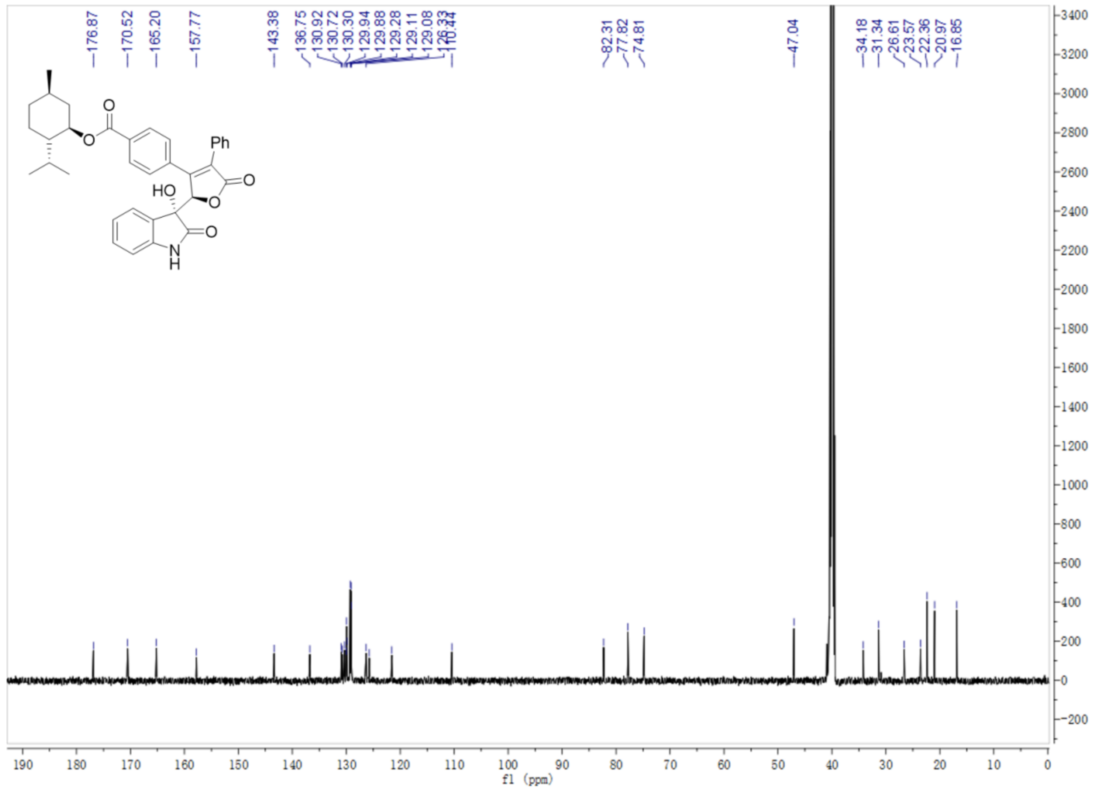

^{1}H-NMR and ^{13}C-NMR for **3av:**

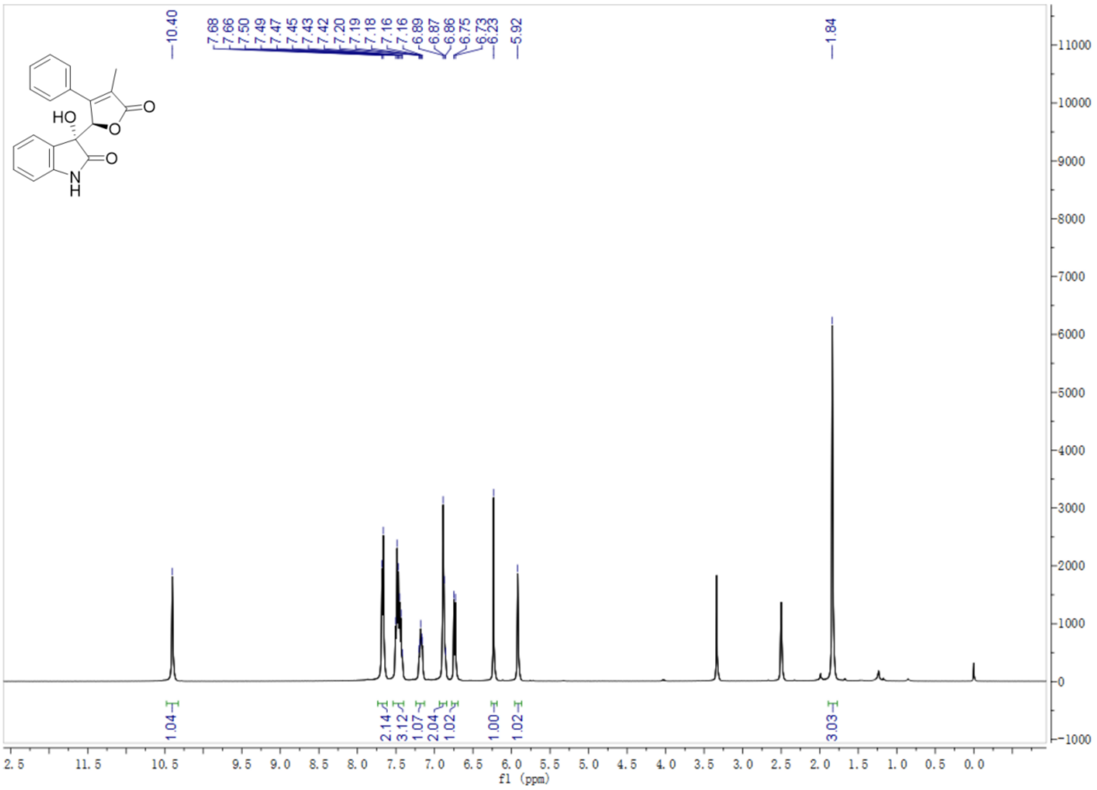

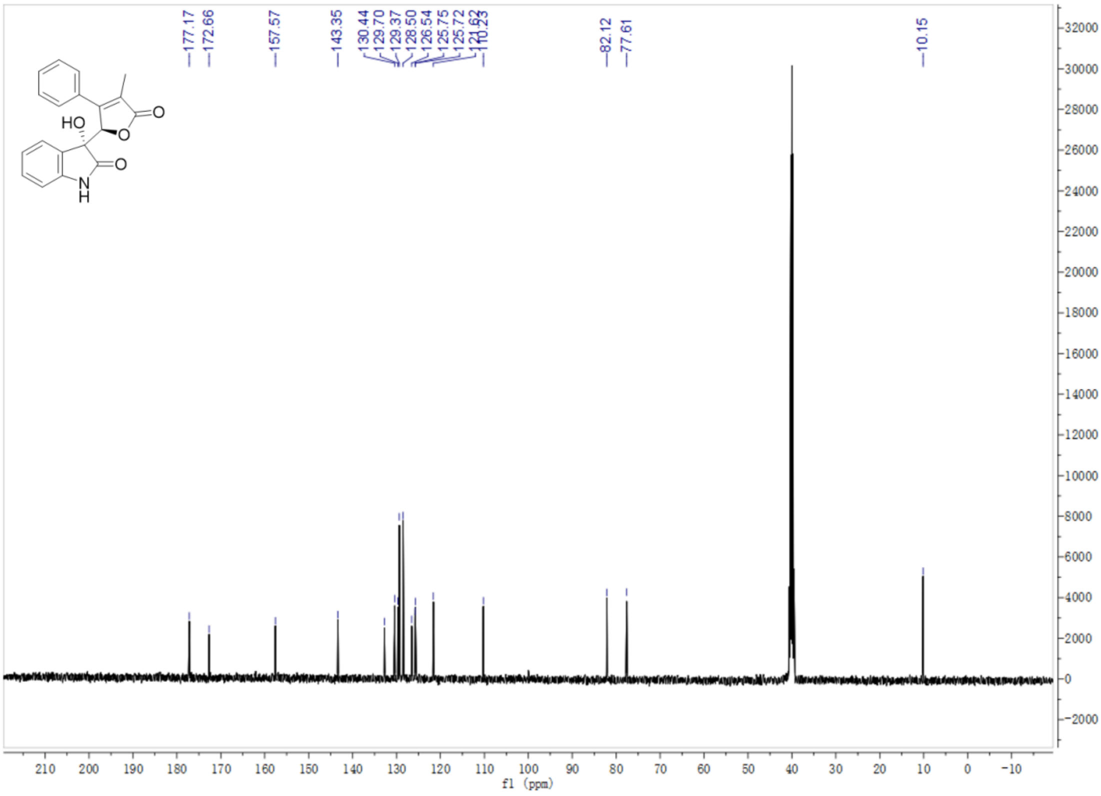

^{1}H-NMR and ^{13}C-NMR for **7a:**

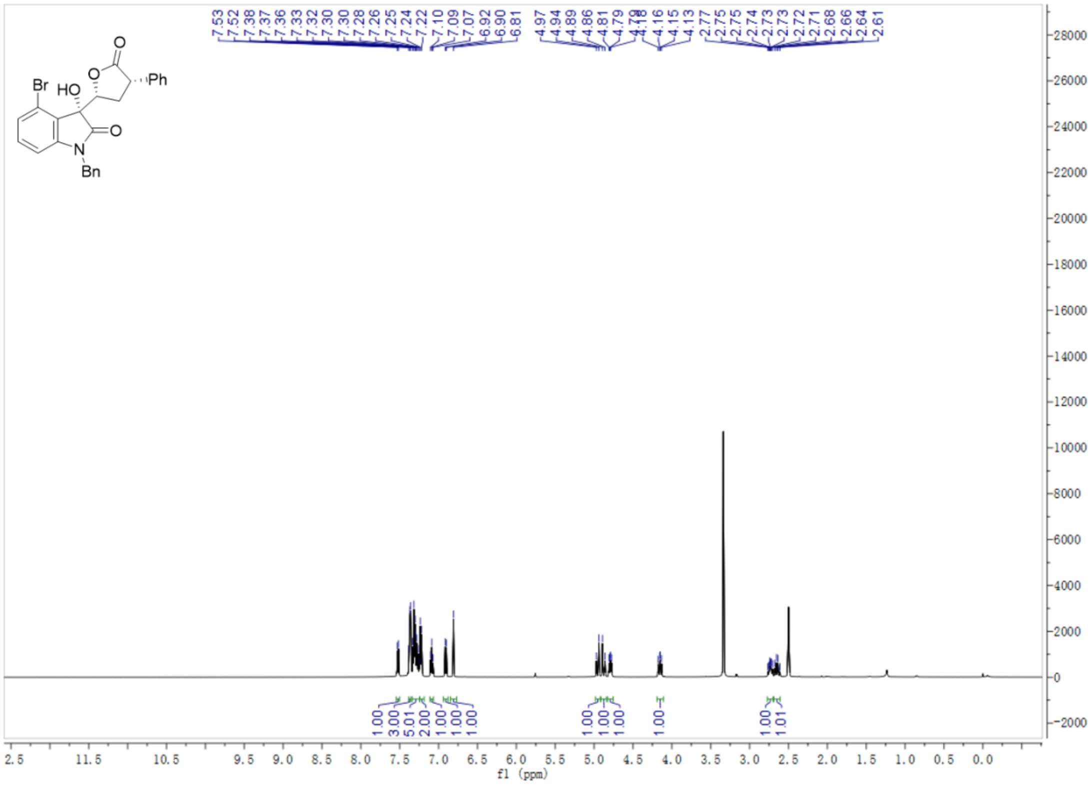

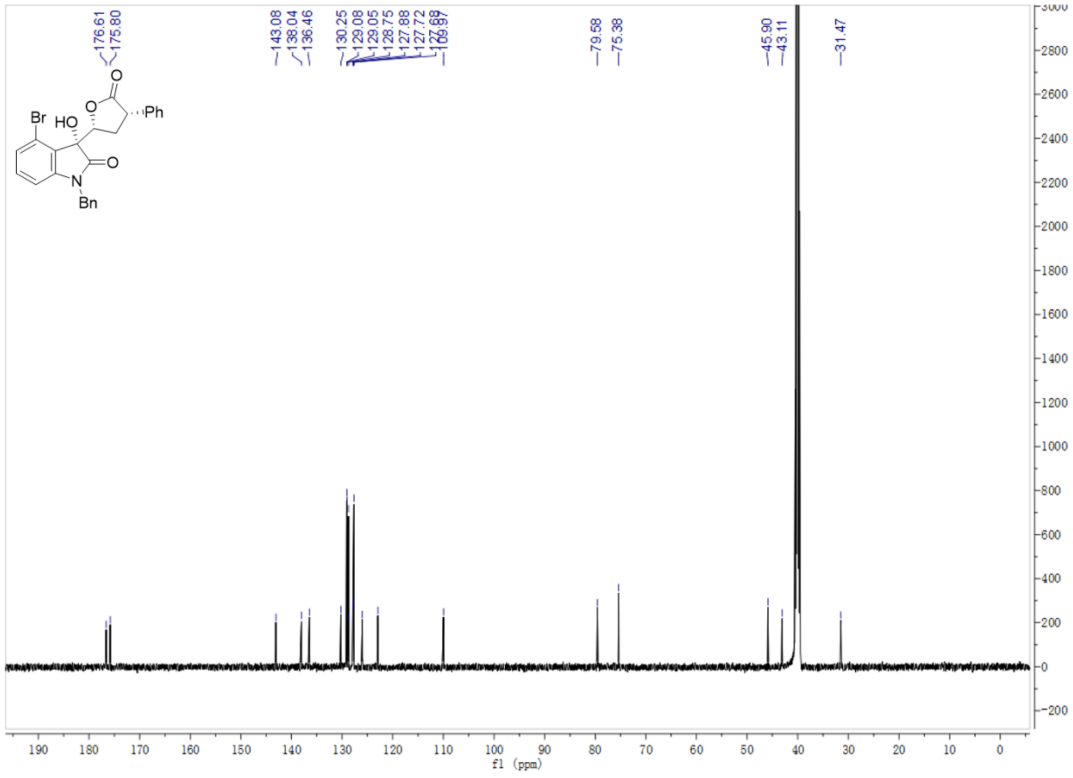

COSY spectrum for compound **7a**

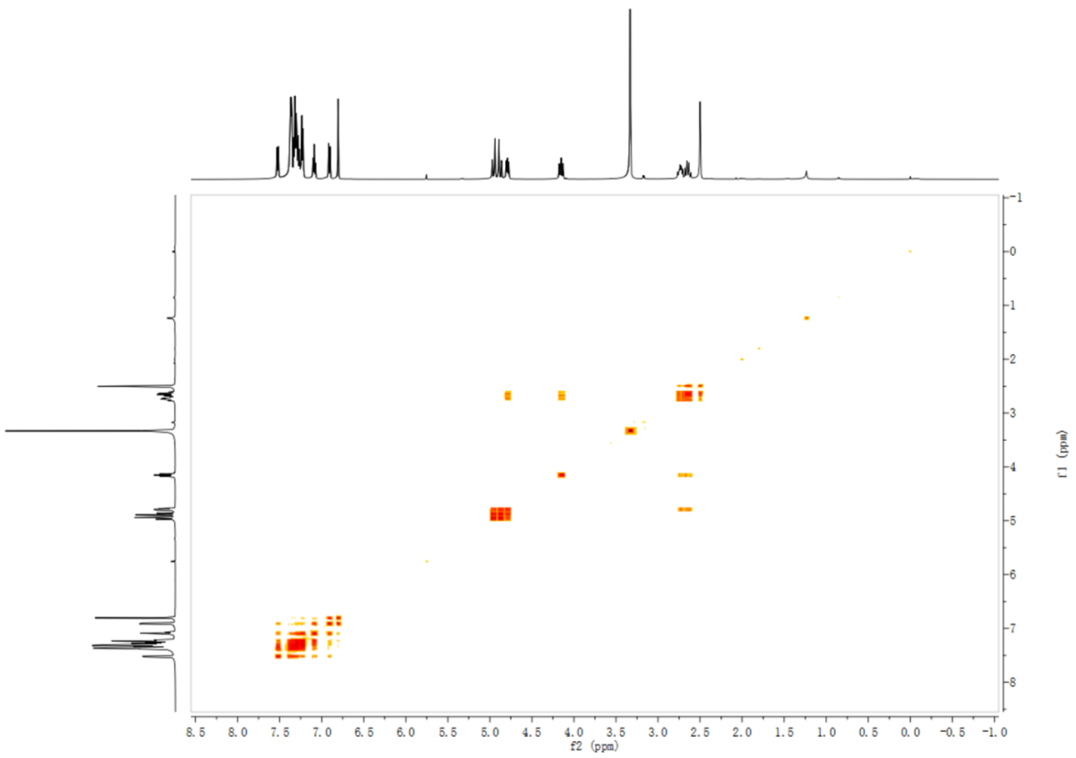

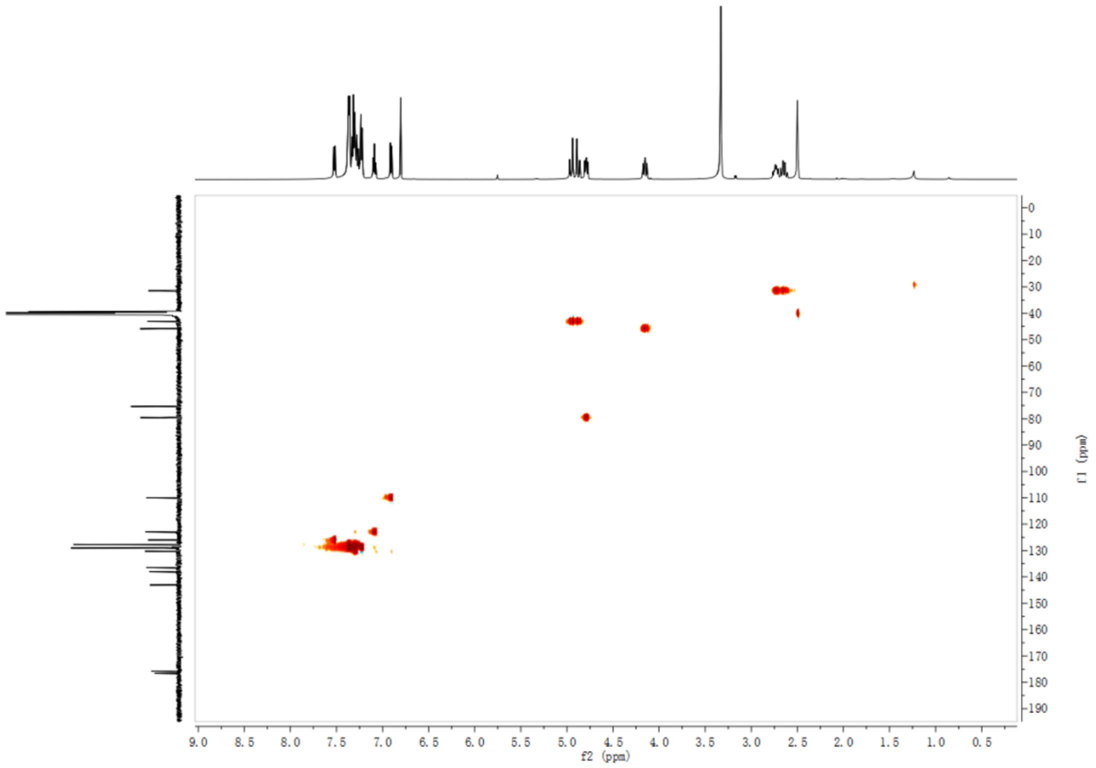

1D NOE spectrum for compound **7a**

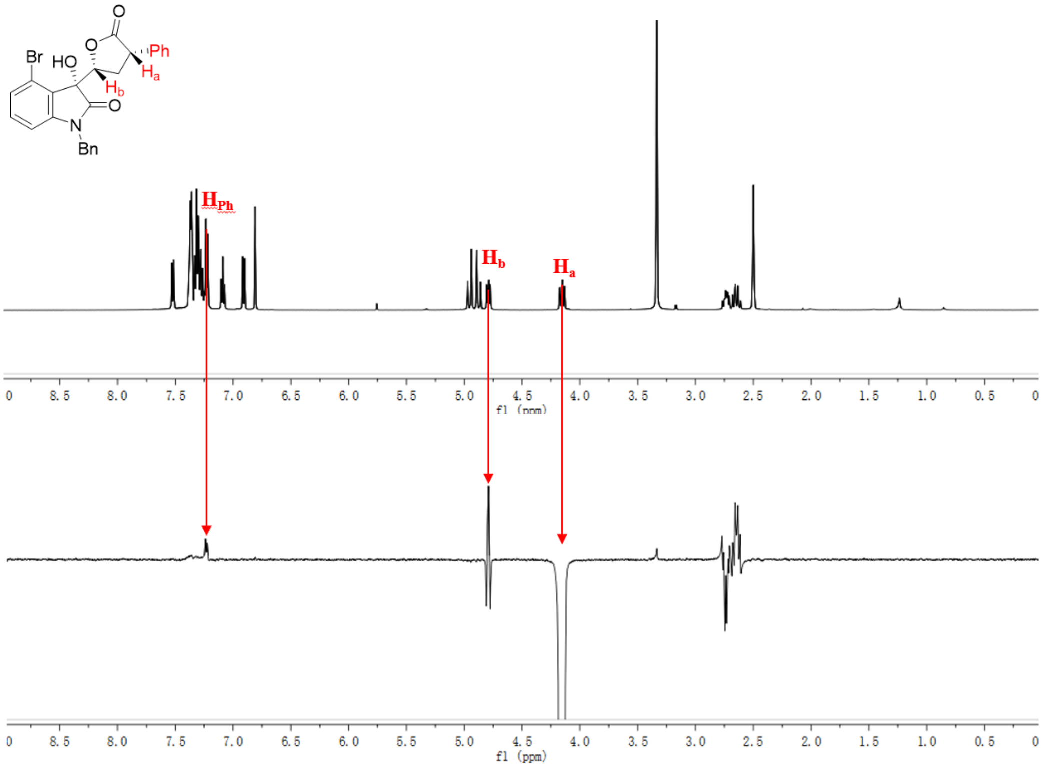

^{1}H-NMR and ^{13}C-NMR for **7b:**

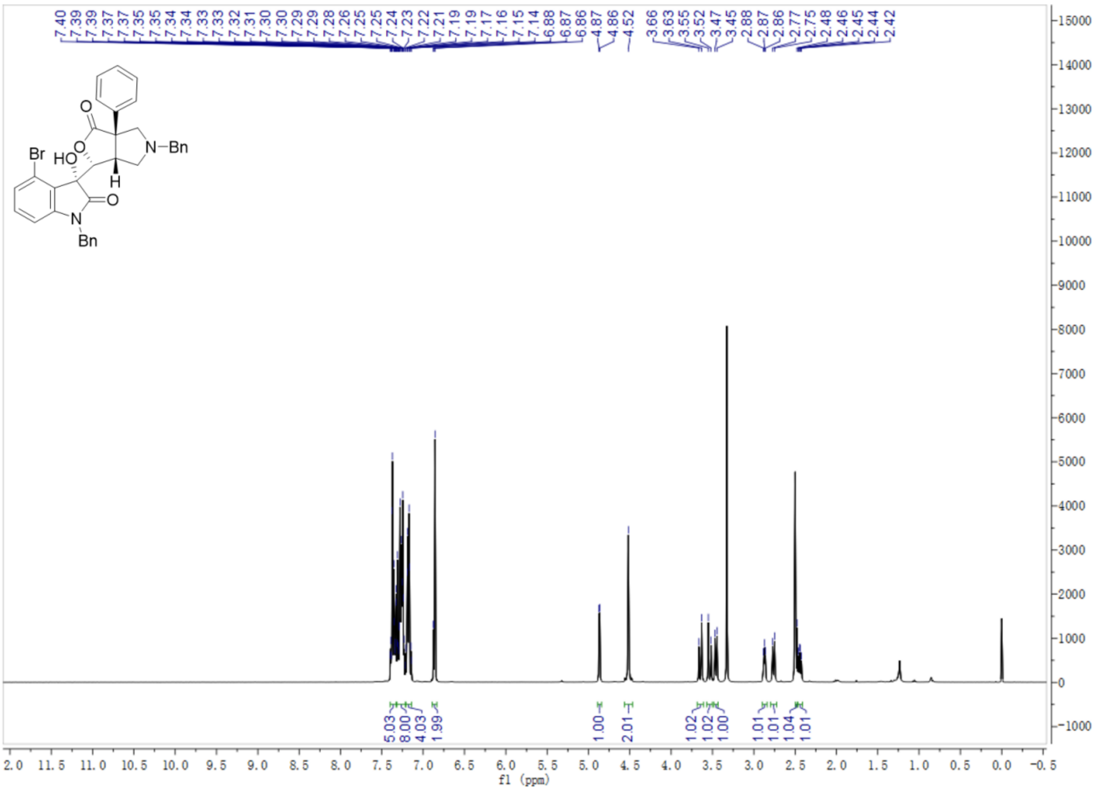

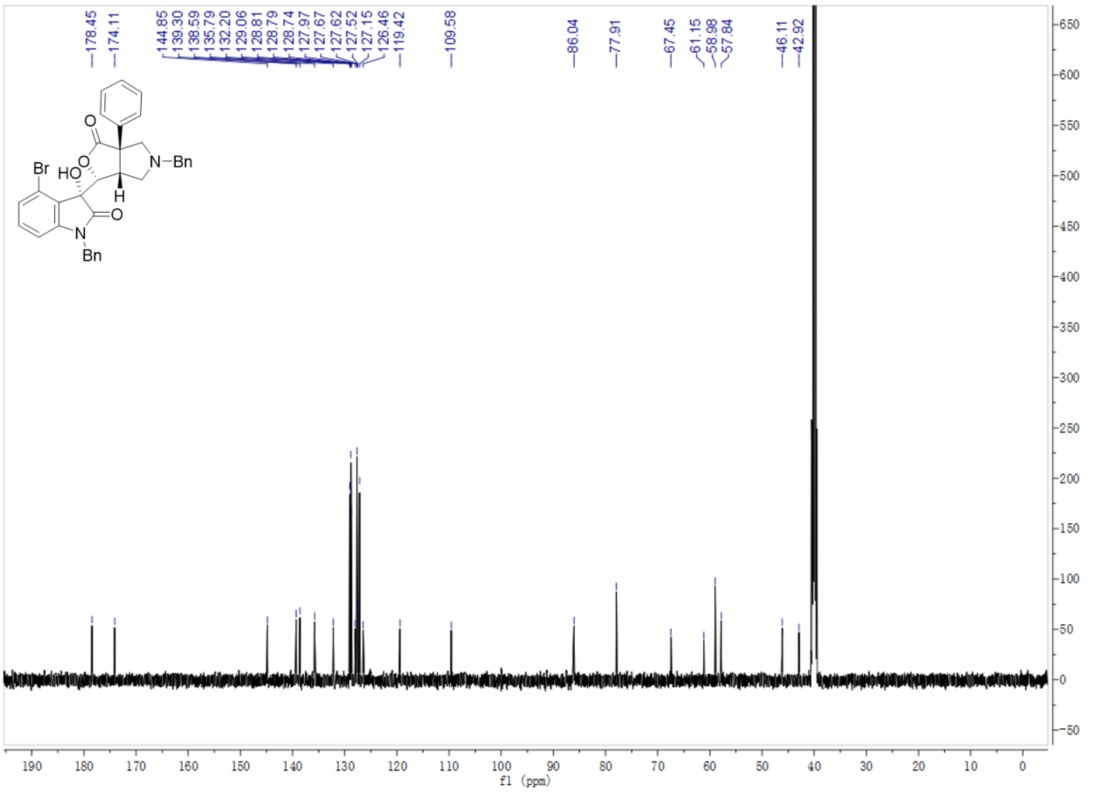

COSY spectrum for compound **7b:**

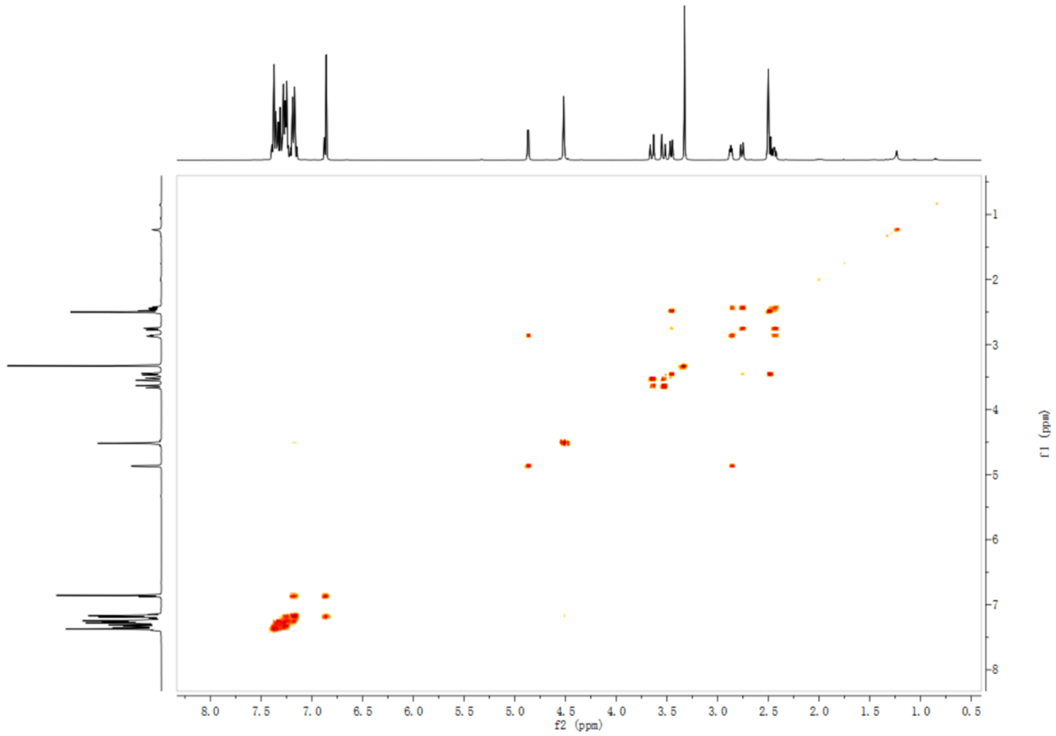

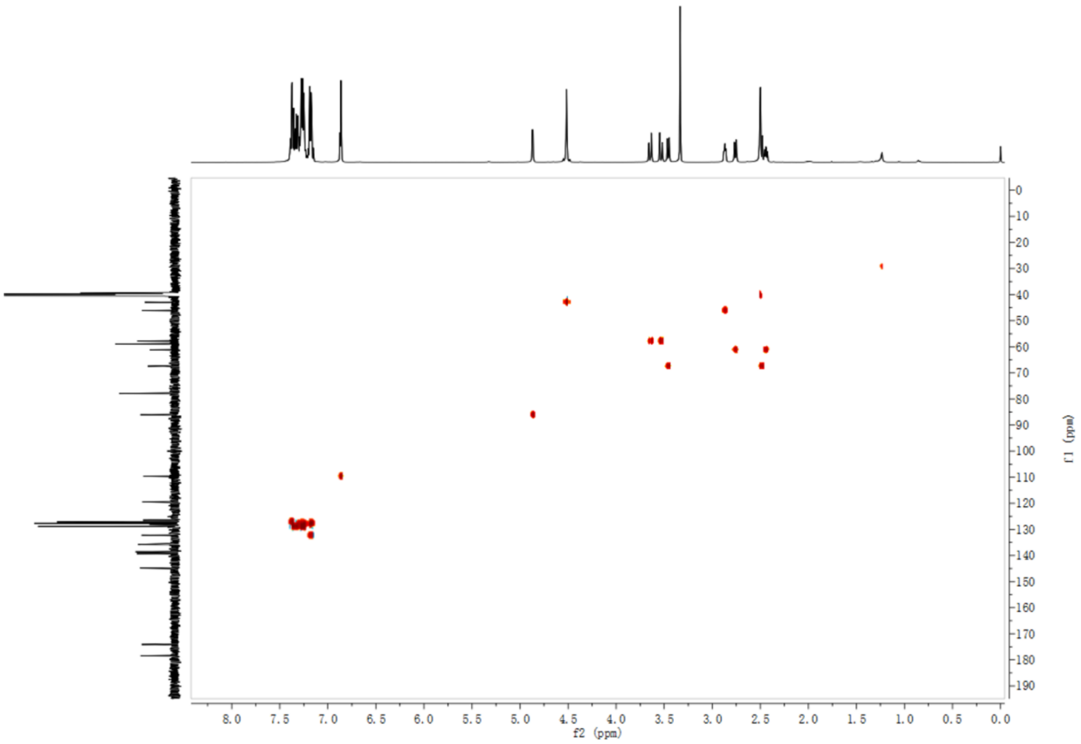

1D NOE spectrum for compound **7b:**

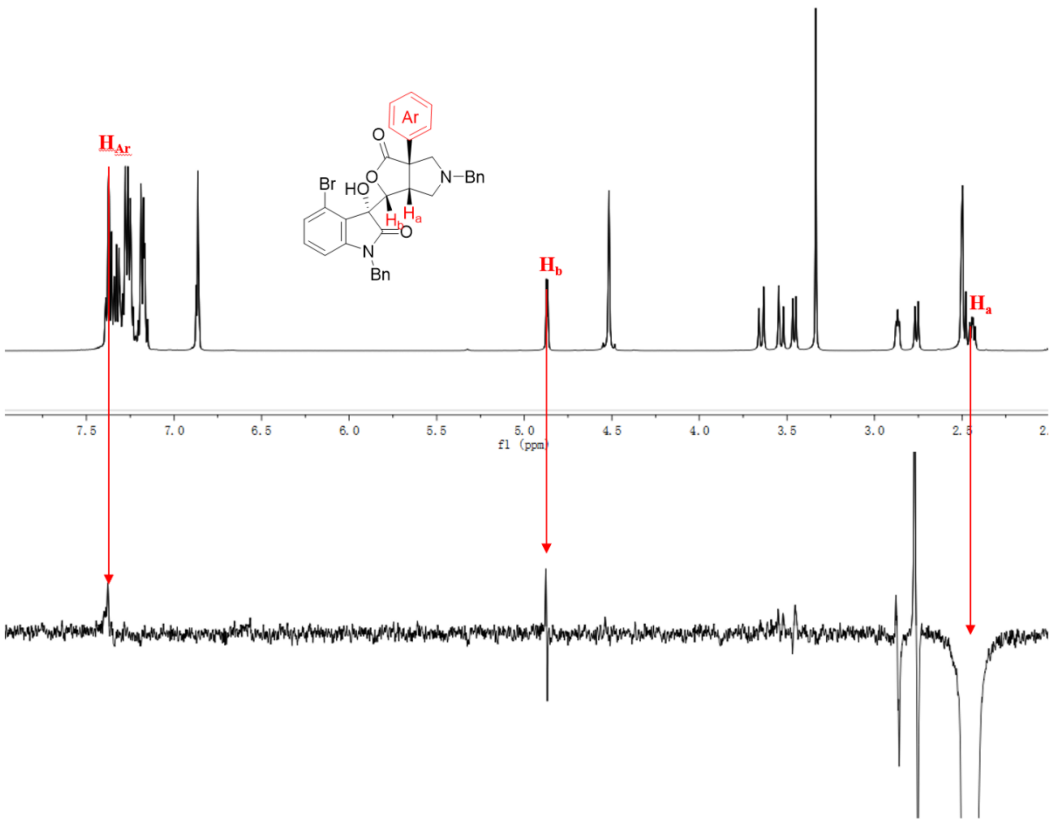

9. HPLC spectrums.

HPLC spectrum for **3a**:

Condition:hexane:2-propanol=80:20
Flow rate=1.0 mL/min, λ=254 nm, Chiral ADH

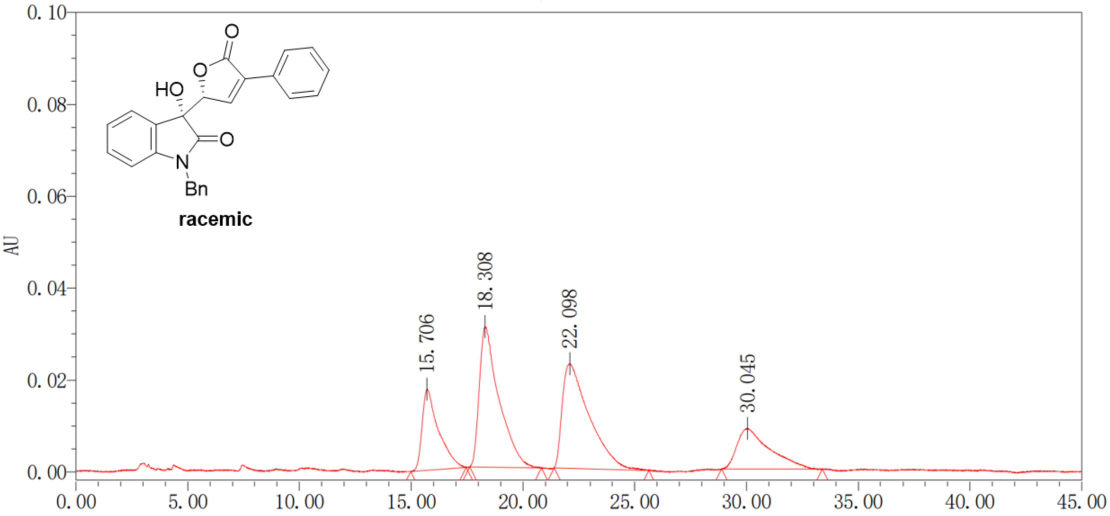

	Name	Ret. time (min)	Area (μV*s)	Height (μV)	%Area
1		15.706	898014	17635	16.07
2		18.308	1883100	30600	33.71
3		22.098	1904427	22734	34.09
4		30.045	901437	8817	16.13

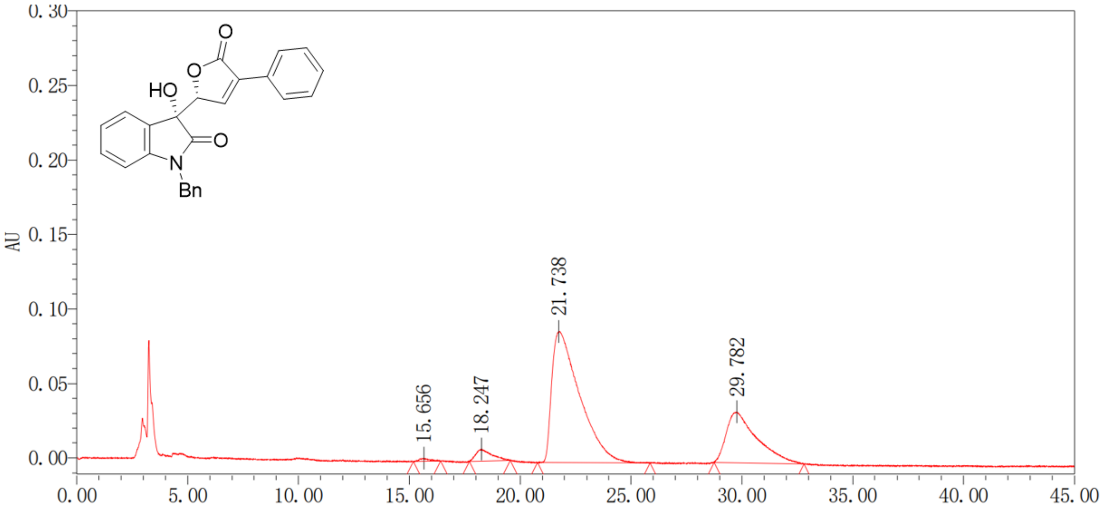

	Name	Ret.time (min)	Area (μV*s)	Height (μV)	%Area
1		15.656	56281	1886	0.49
2		18.247	400066	8011	3.52
3		21.738	7709623	88280	67.75
4		29.782	3213254	34053	28.24

HPLC spectrum for **3b:**

Condition:hexane:2-propanol=80:20

Flow rate=1.0 mL/min, λ=254 nm, Chiral IC

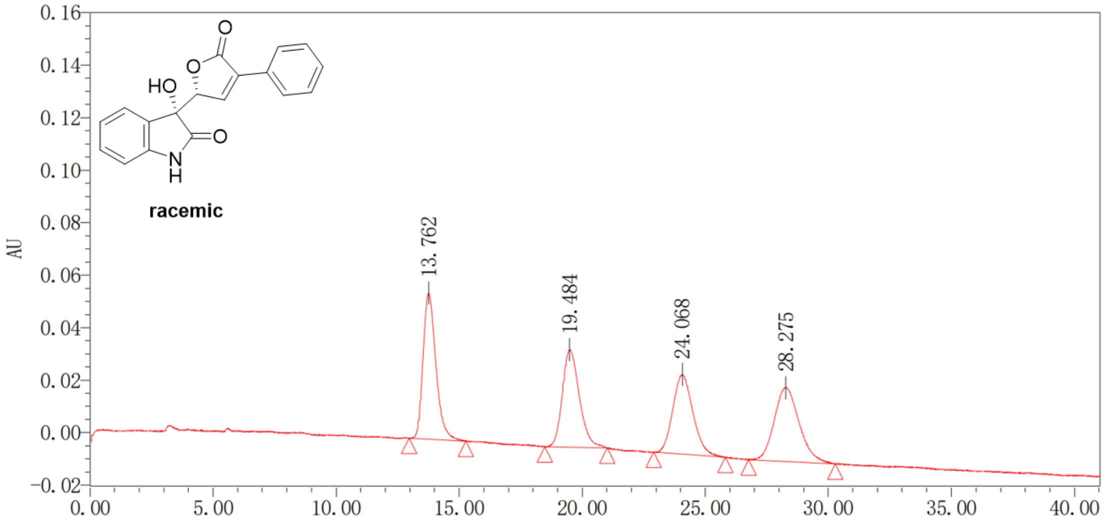

	Name	Ret.time (min)	Area (μV*s)	Height (μV)	%Area
**1**		13.762	1995878	55697	26.63
**2**		19.484	1786591	37101	23.84
**3**		24.068	1729568	30375	23.07
**4**		28.275	1983521	28157	26.46

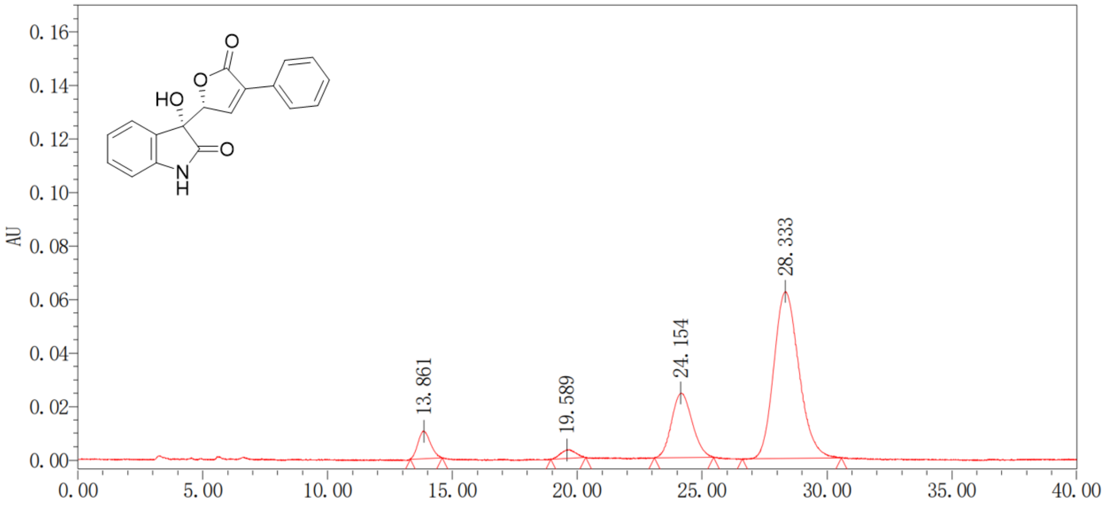

	Name	Ret.time (min)	Area (μV*s)	Height (μV)	%Area
**1**		13.861	353288	10258	5.69
**2**		19.589	137834	3234	2.22
**3**		24.154	1369603	24127	22.05
**4**		28.333	4350207	62356	70.04

HPLC spectrum for **3c:**

Condition:hexane:2-propanol=70:30
Flow rate=1.0 mL/min, λ=254 nm, Chiral IC

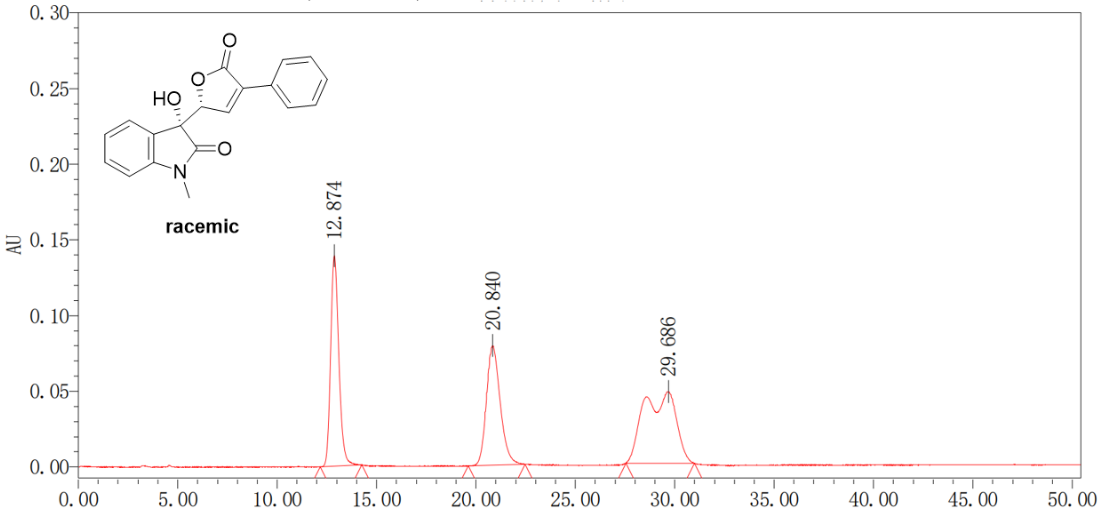

	Name	Ret.time (min)	Area (μV*s)	Height (μV)	%Area
**1**		12.874	3933263	139236	29.83
**2**		20.840	3856339	78959	29.25
**3**		29.686	5395478	47441	40.92

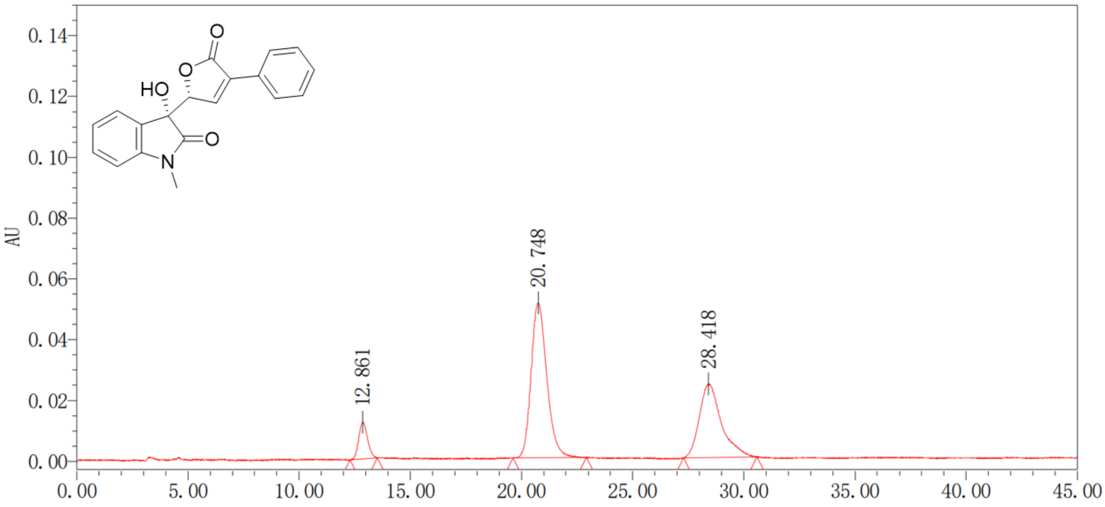

	Name	Ret.time (min)	Area (μV*s)	Height (μV)	%Area
**1**		12.861	332323	12110	7.38
**2**		20.748	250698	50994	55.68
**3**		28.418	1663440	24169	36.94

HPLC spectrum for **3d:**

Condition:hexane:2-propanol=70:30
Flow rate=1.0 mL/min, λ=254 nm, Chiral ODH

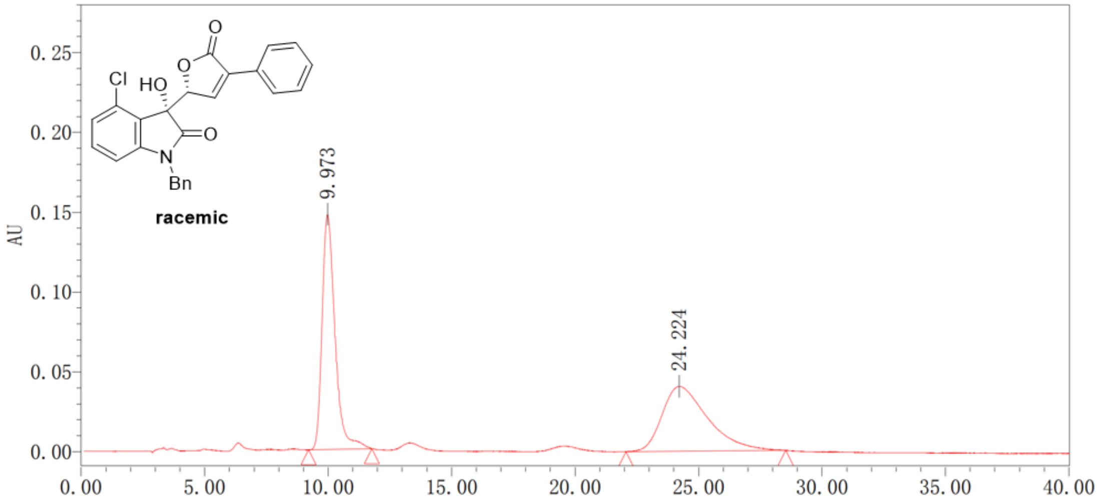

	Name	Ret.time (min)	Area (μV*s)	Height (μV)	%Area
1		9.973	5325807	147250	50.49
2		24.224	5223442	40727	49.51

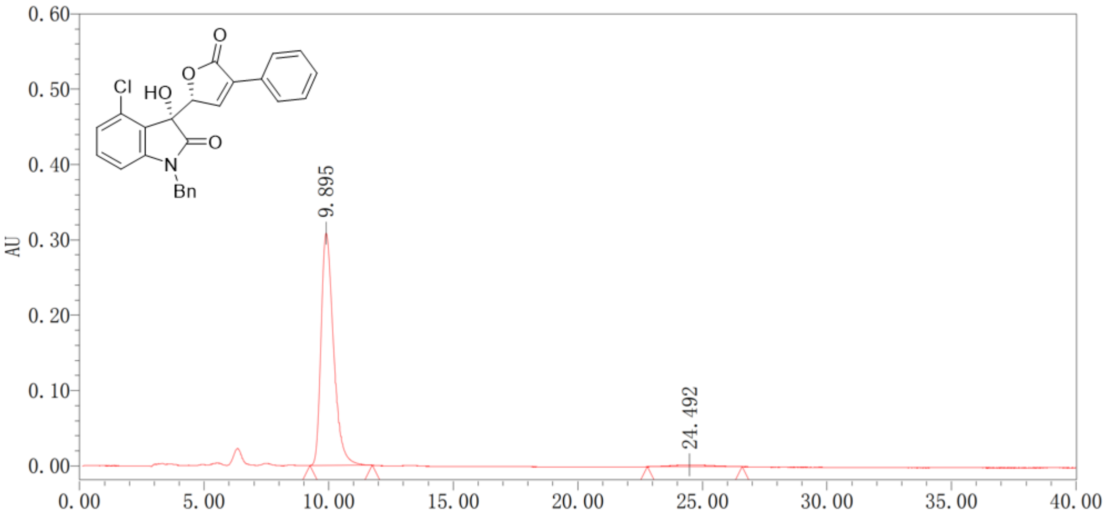

	Name	Ret.time (min)	Area (μV*s)	Height (μV)	%Area
1		9.895	10651359	308438	97.43
2		24.492	280791	2518	2.57

HPLC spectrum for **3e:**

Condition:hexane:2-propanol=80:20
Flow rate=1.0 mL/min, λ=254 nm, Chiral ADH

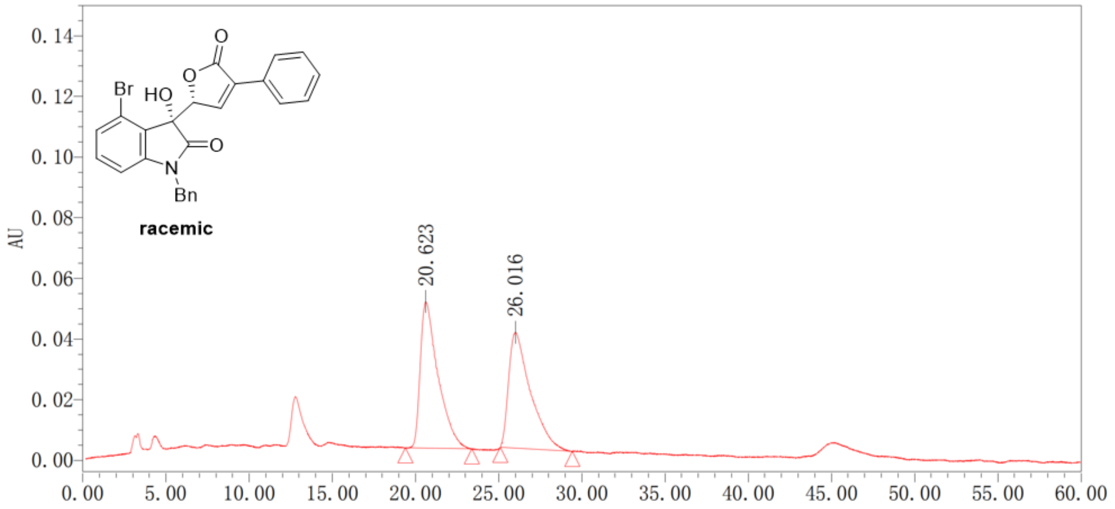

	Name	Ret.time (min)	Area (μV*s)	Height (μV)	%Area
**1**		20.623	3581624	48324	50.90
**2**		26.016	3455553	38151	49.10

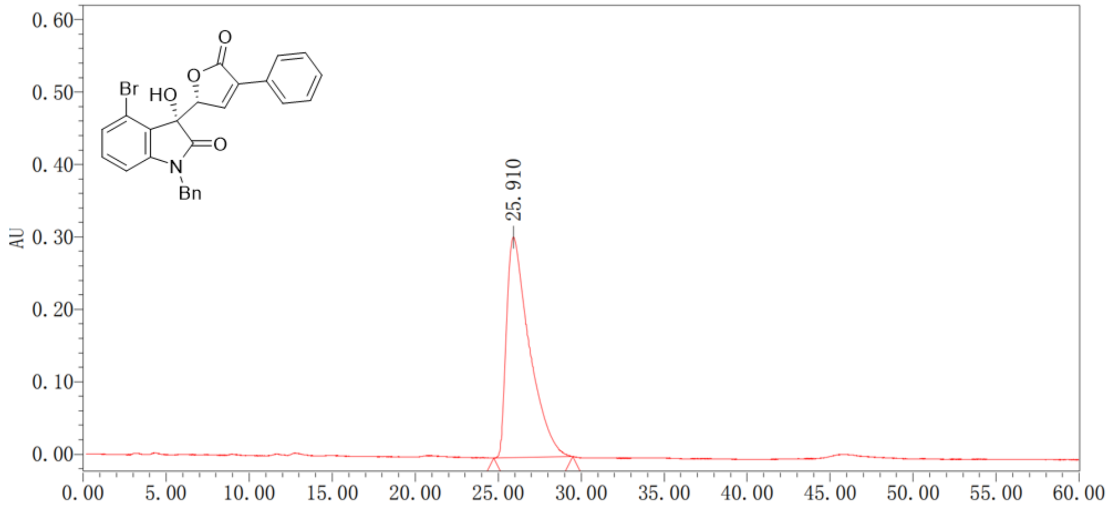

	Name	Ret.time (min)	Area (μV*s)	Height (μV)	%Area
**1**		25.910	29036293	304234	100.00
**2**					

HPLC spectrum for **3f**:

Condition:hexane:2-propanol=80:20
Flow rate=1.0 mL/min, λ=254 nm, Chiral ADH

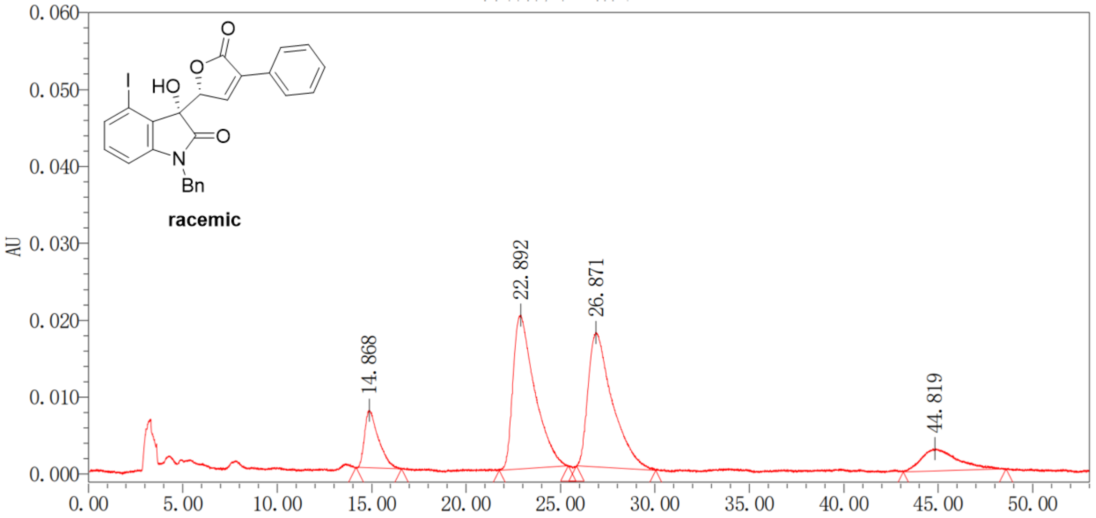

	Name	Ret.time (min)	Area (μV*s)	Height (μV)	%Area
1		14.868	382999	7471	9.82
2		22.892	1587403	19998	40.71
3		26.871	1557498	17503	39.94
4		44.819	371351	2879	9.52

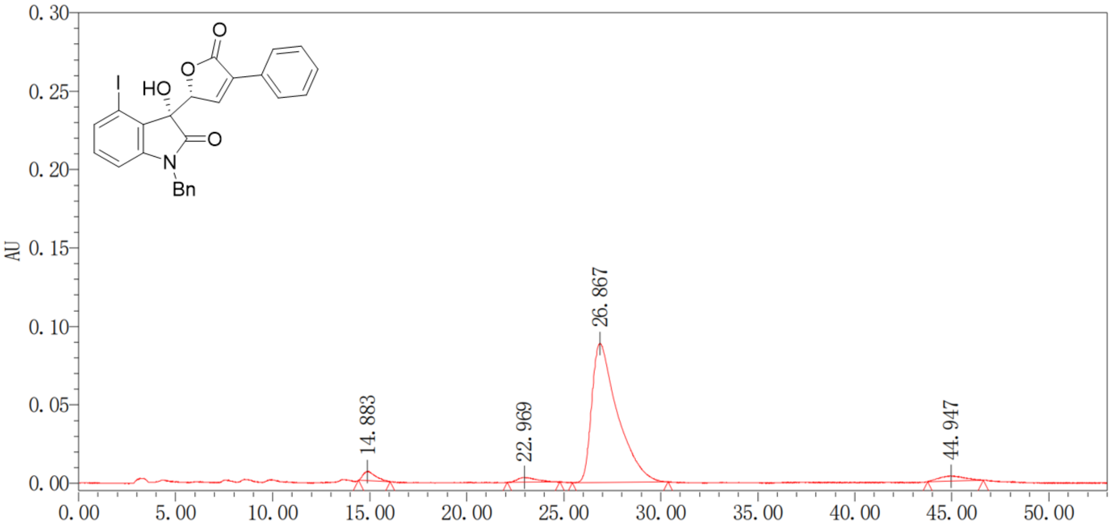

	Name	Ret.time (min)	Area (μV*s)	Height (μV)	%Area
1		14.883	265444	6035	2.96
2		22.969	210747	3071	2.35
3		26.867	8206622	88630	91.49
4		44.947	286745	3187	3.20

HPLC spectrum for **3g:**

Condition:hexane:2-propanol=80:20
Flow rate=1.0 mL/min, λ=254 nm, Chiral ADH

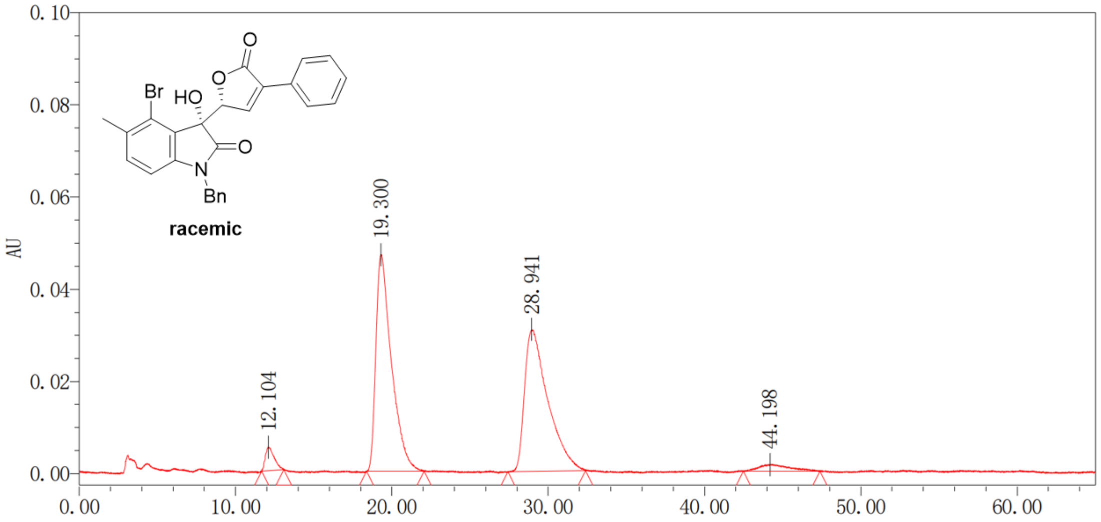

	Name	Ret.time (min)	Area (μV*s)	Height (μV)	%Area
1		12.104	201446	5162	2.95
2		19.300	3234842	47006	47.34
3		28.941	3202204	30835	46.87
4		44.198	194116	1459	2.84

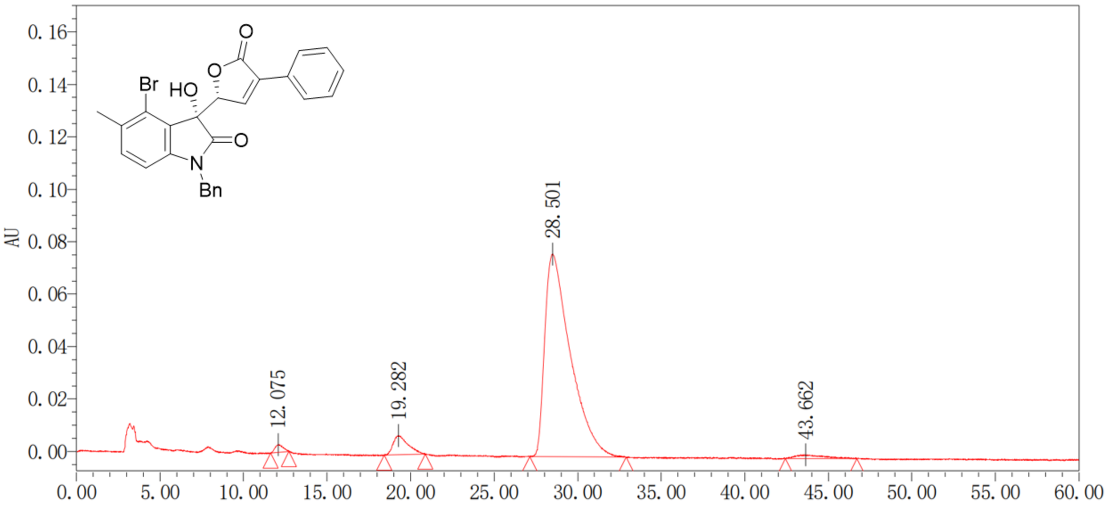

	Name	Ret.time (min)	Area (μV*s)	Height (μV)	%Area
1		12.075	99729	3001	1.12
2		19.282	446337	7241	5.01
3		28.501	8206493	77255	92.10
4		43.662	157875	1358	1.77

HPLC spectrum for **3h:**

Condition:hexane:2-propanol=80:20
Flow rate=1.0 mL/min, λ=254 nm, Chiral ADH

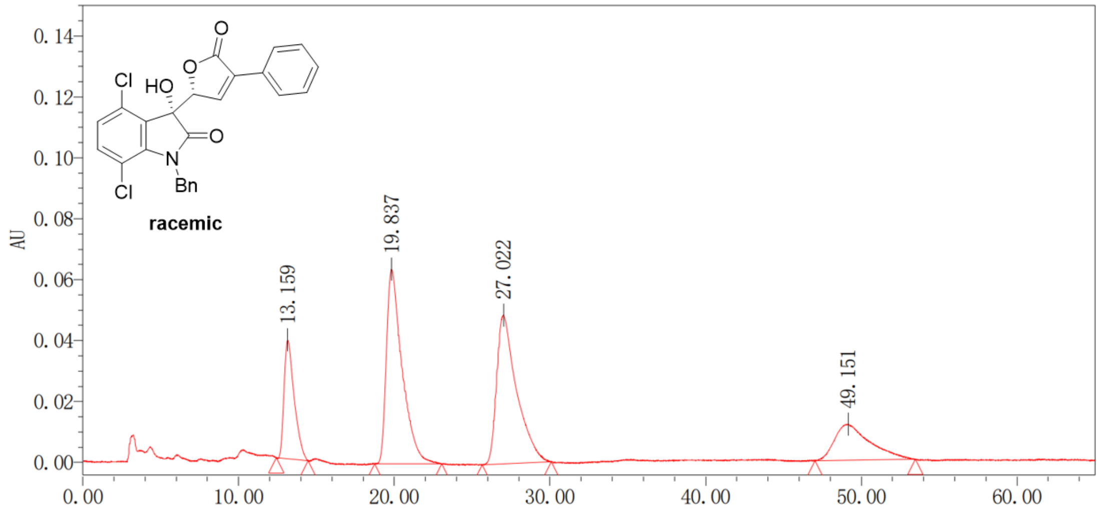

	Name	Ret.time (min)	Area (μV*s)	Height (μV)	%Area
**1**		13.159	1754195	39031	14.05
**2**		19.837	4478795	63815	35.87
**3**		27.022	4391200	48939	35.17
**4**		49.151	1861643	11861	14.91

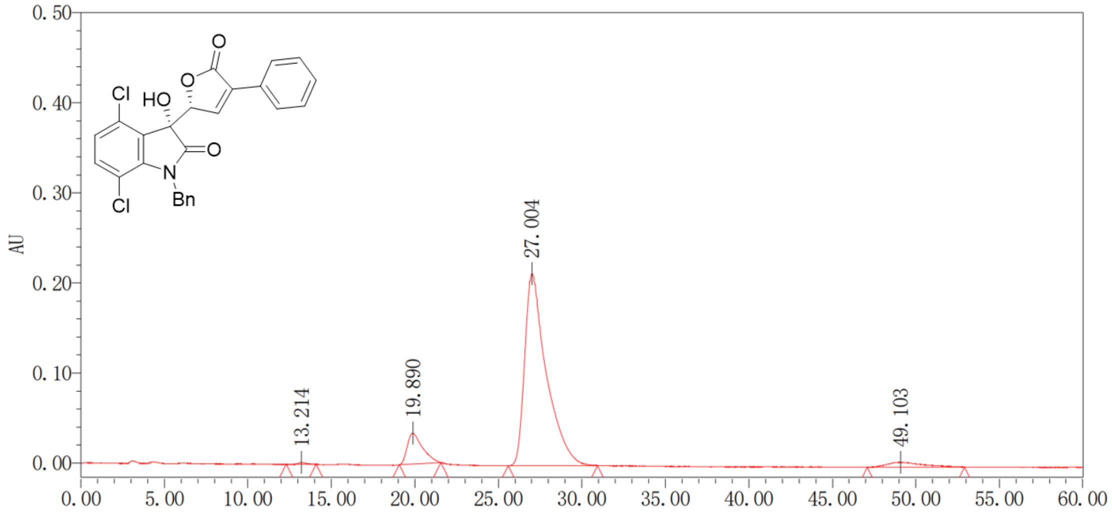

	Name	Ret.time (min)	Area (μV*s)	Height (μV)	%Area
**1**		13.214	79577	2130	0.36
**2**		19.890	2144448	34226	9.60
**3**		27.004	19322651	213066	86.47
**4**		49.103	799020	5436	3.58

HPLC spectrum for **3i:**

Condition:hexane:2-propanol=80:20
Flow rate=1.0 mL/min, λ=254 nm, Chiral IC

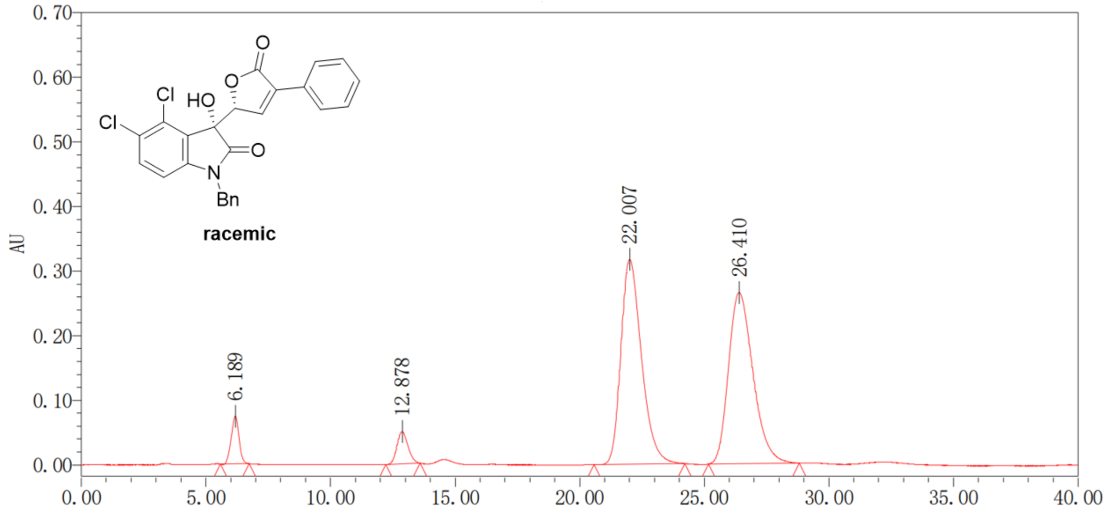

	Name	Ret.time (min)	Area (μV*s)	Height (μV)	%Area
**1**		6.189	1605526	73754	4.06
**2**		12.878	1560668	50073	3.95
**3**		22.007	18295569	316949	46.31
**4**		26.410	18044107	265020	45.67

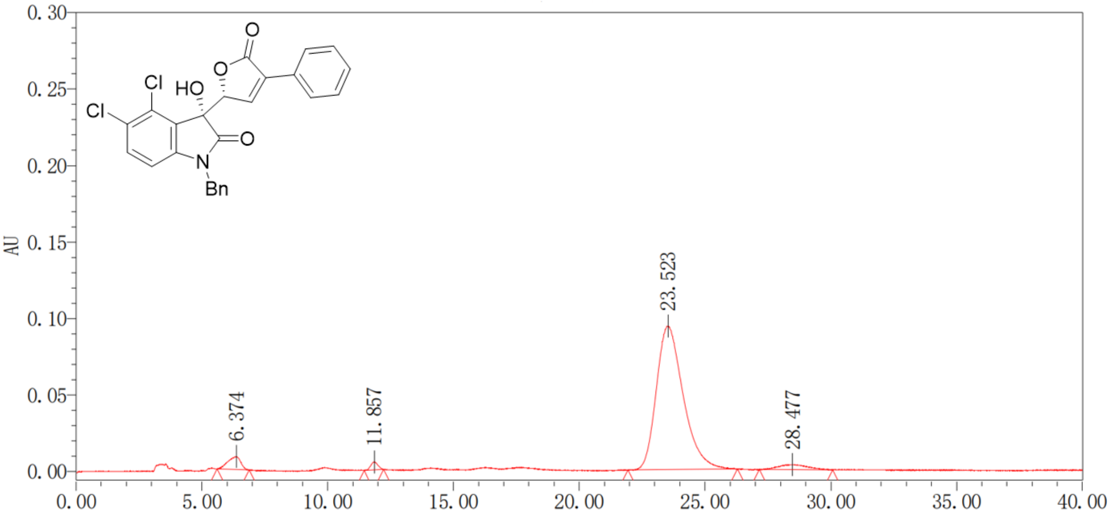

	Name	Ret.time (min)	Area (μV*s)	Height (μV)	%Area
**1**		6.374	296979	8519	3.87
**2**		11.857	97183	5064	1.27
**3**		23.523	7026119	93896	91.51
**4**		28.477	258104	3281	3.36

HPLC spectrum for **3j:**

Condition:hexane:2-propanol=80:20
Flow rate=1.0 mL/min, λ=254 nm, Chiral ADH

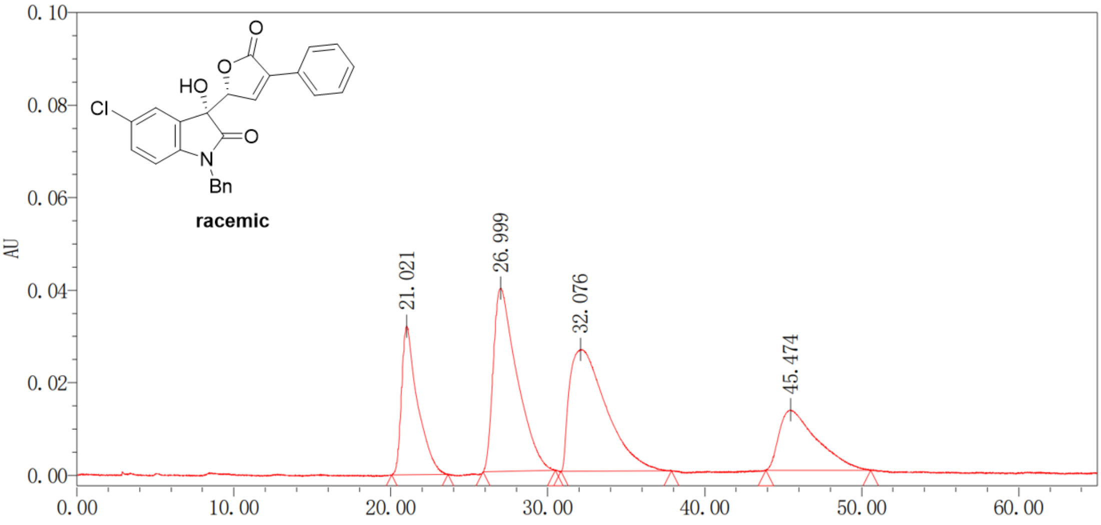

	Name	Ret.time (min)	Area (μV*s)	Height (μV)	%Area
**1**		21.021	2284802	32129	17.62
**2**		26.999	4194540	39641	32.35
**3**		32.076	4321015	26349	33.32
**4**		45.474	2167085	13033	16.71

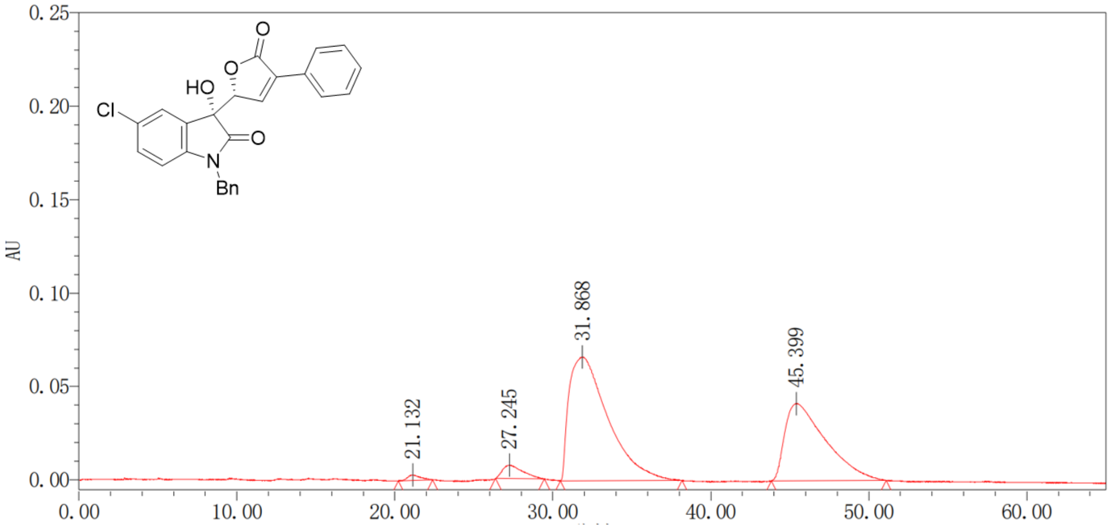

	Name	Ret.time (min)	Area (μV*s)	Height (μV)	%Area
**1**		21.132	160088	2850	0.82
**2**		27.245	628885	7118	3.21
**3**		31.868	11358355	66225	58.03
**4**		45.399	7426251	41451	37.94

HPLC spectrum for **3k:**

Condition:hexane:2-propanol=70:30
Flow rate=1.0 mL/min, λ=254 nm, Chiral ODH

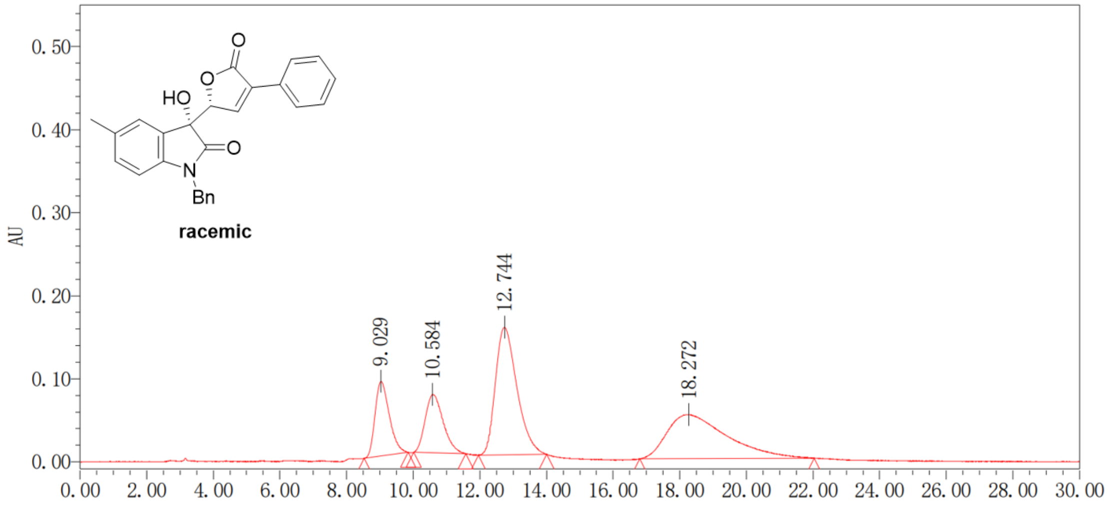

	Name	Ret.time (min)	Area (μV*s)	Height (μV)	%Area
1		9.029	2656566	89845	14.13
2		10.584	2635536	70040	14.02
3		12.744	6863818	153676	36.51
4		18.272	6641717	53065	35.33

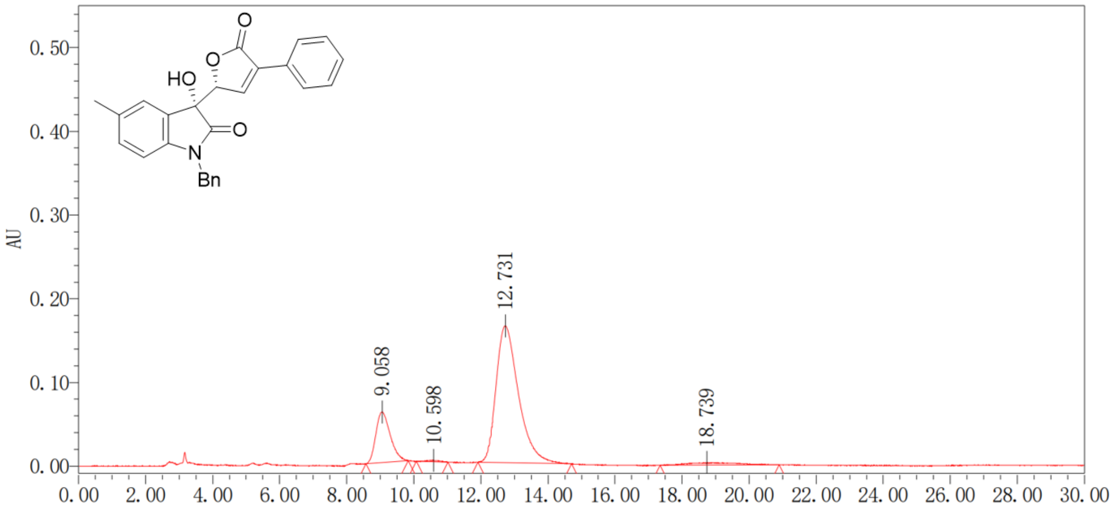

	Name	Ret.time (min)	Area (μV*s)	Height (μV)	%Area
1		9.058	1782633	60609	18.42
2		10.598	41403	1559	0.43
3		12.731	7553198	163499	78.05
4		18.739	300712	2880	3.11

HPLC spectrum for **3l:**

Condition:hexane:2-propanol=80:20
Flow rate=1.0 mL/min, λ=254 nm, Chiral ADH

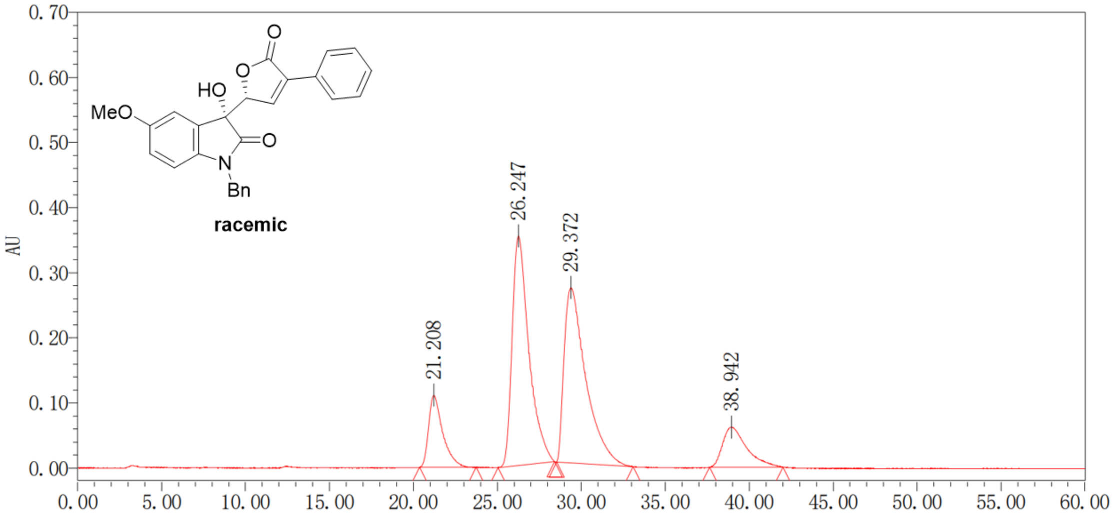

	Name	Ret.time (min)	Area (μV*s)	Height (μV)	%Area
**1**		21.208	6499387	110919	10.61
**2**		26.247	24442638	352444	39.91
**3**		29.372	24218422	269074	39.54
**4**		38.942	6083084	61652	9.93

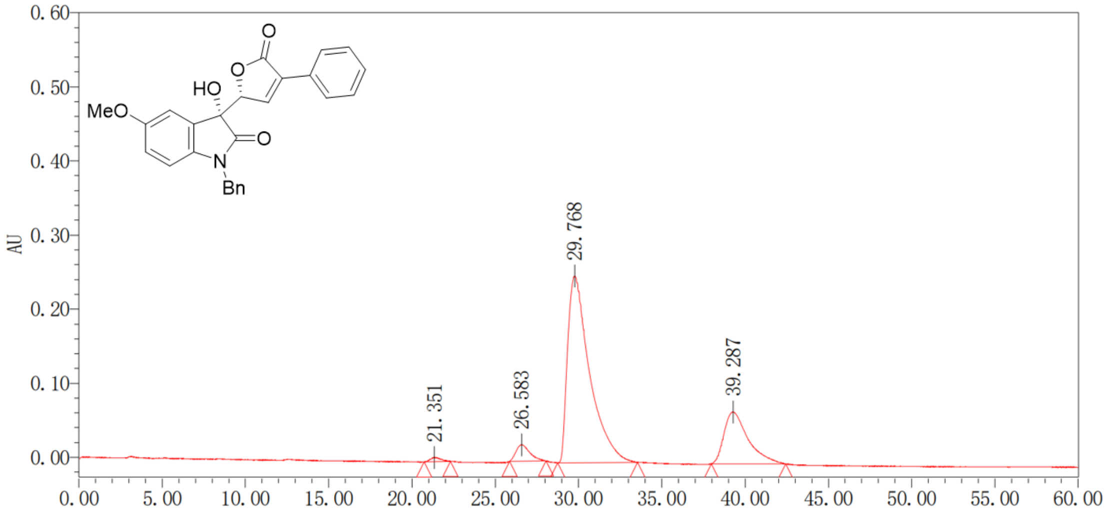

	Name	Ret.time (min)	Area (μV*s)	Height (μV)	%Area
**1**		21.351	237477	5640	0.75
**2**		26.583	1281365	22302	4.03
**3**		29.768	23311813	251986	73.30
**4**		39.287	6974602	69964	21.93

HPLC spectrum for **3m:**

Condition:hexane:2-propanol=75:25
Flow rate=1.0 mL/min, λ=254 nm, Chiral ADH

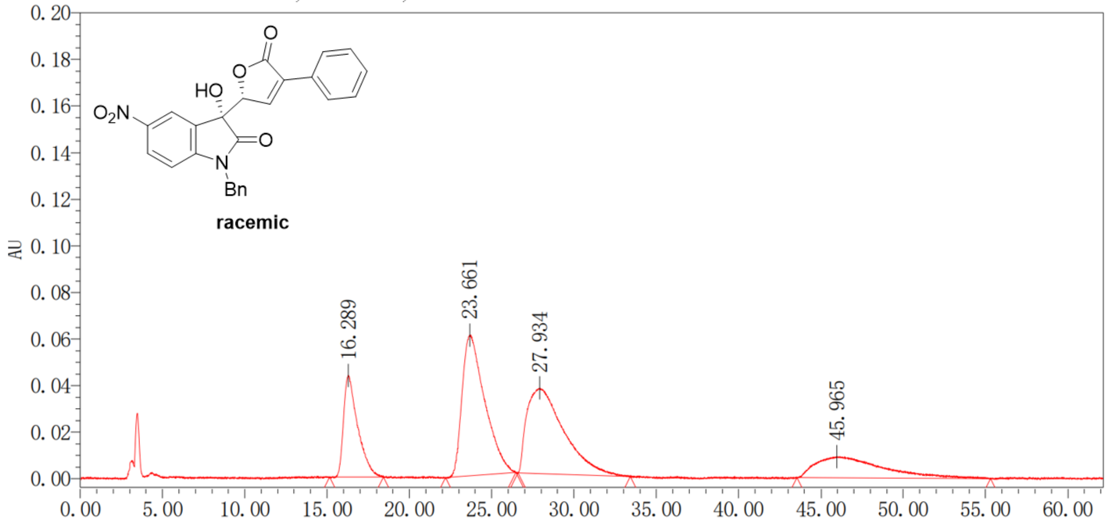

	Name	Ret. time (min)	Area (μV*s)	Height (μV)	%Area
1		16.289	2595029	43550	15.50
2		23.661	5778619	60495	34.52
3		27.934	5735374	36809	34.26
4		45.965	2631007	9246	15.72

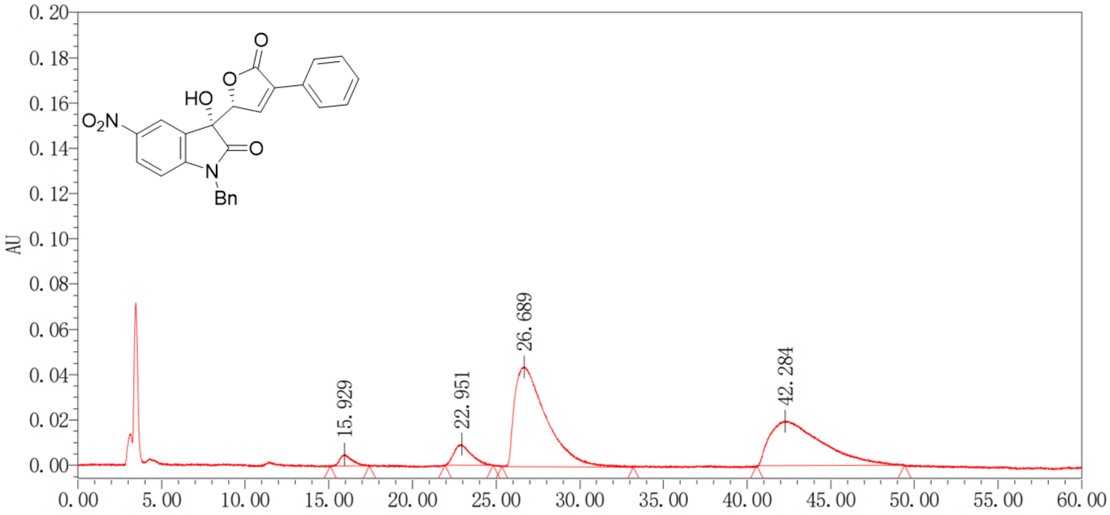

	Name	Ret. time (min)	Area (μV*s)	Height (μV)	%Area
1		15.929	253827	5258	2.31
2		22.951	657677	9291	5.98
3		26.689	5813672	44225	52.90
4		42.284	4265694	19639	38.81

HPLC spectrum for **3n:**

Condition:hexane:2-propanol=80:20
Flow rate=1.0 mL/min, λ=254 nm, Chiral ODH

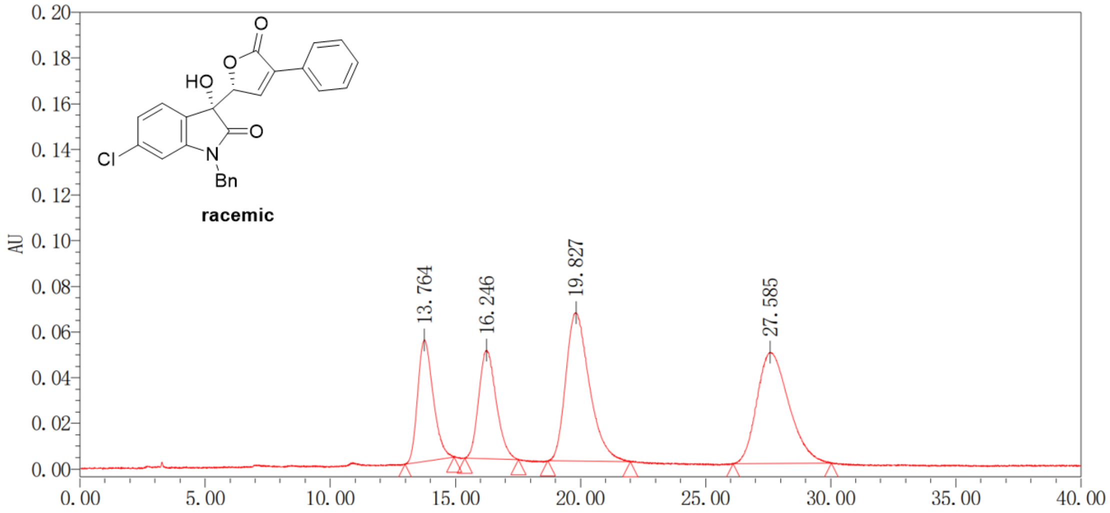

	Name	Ret.time (min)	Area (μV*s)	Height (μV)	%Area
**1**		13.764	2269195	53268	17.13
**2**		16.246	2337317	47610	17.64
**3**		19.827	4296385	65129	32.43
**4**		27.585	4344370	48794	32.79

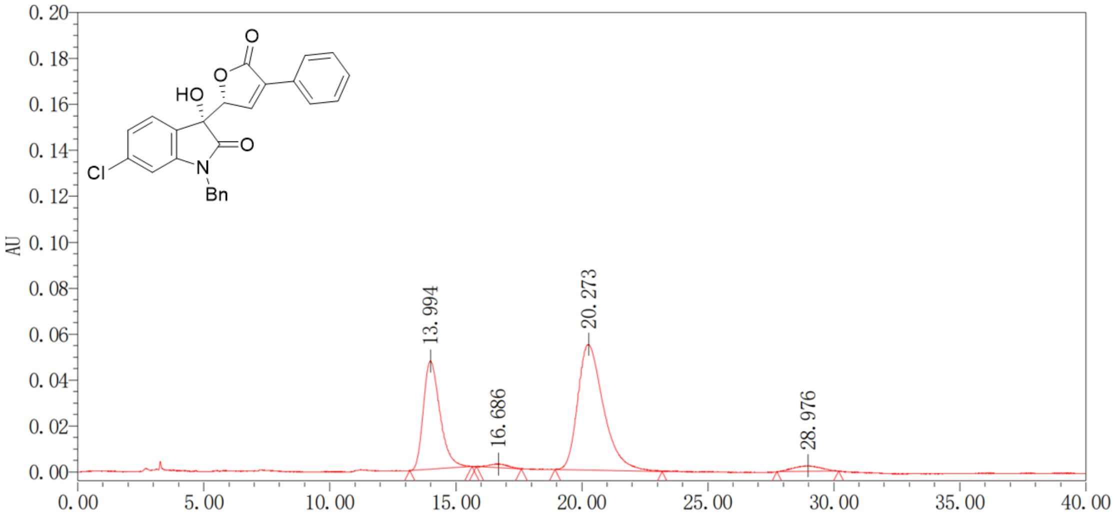

	Name	Ret.time (min)	Area (μV*s)	Height (μV)	%Area
**1**		13.994	2159354	47215	34.15
**2**		16.686	79364	1587	1.26
**3**		20.273	3899060	54783	61.67
**4**		28.976	185183	2424	2.93

HPLC spectrum for **3o:**

Condition:hexane:2-propanol=90:10
Flow rate=1.0 mL/min, λ=254 nm, Chiral ODH

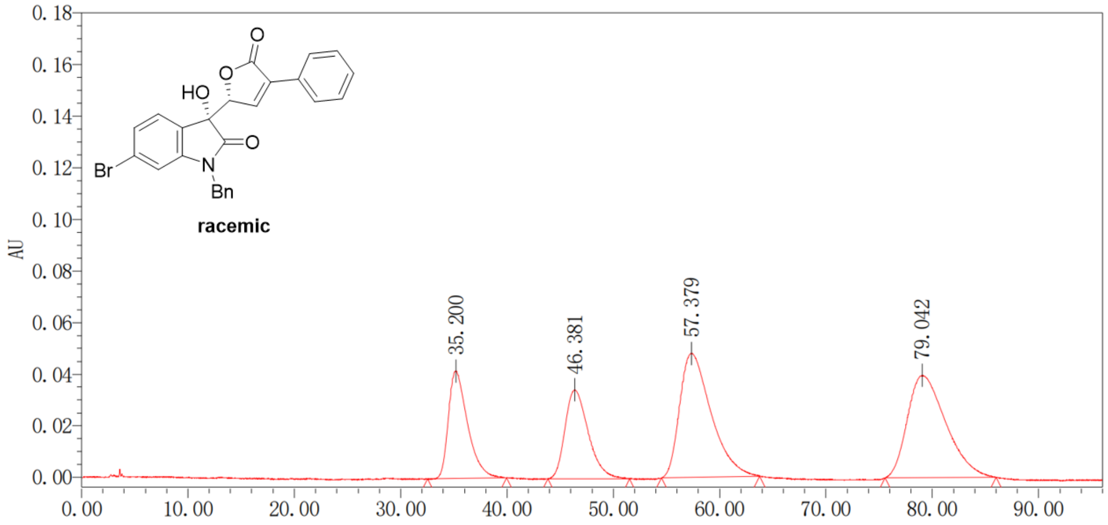

	Name	Ret.time (min)	Area (μV*s)	Height (μV)	%Area
**1**		35.200	5261457	41687	17.35
**2**		46.381	5196825	34428	17.14
**3**		57.379	9866378	48070	32.53
**4**		79.042	10002467	39675	32.98

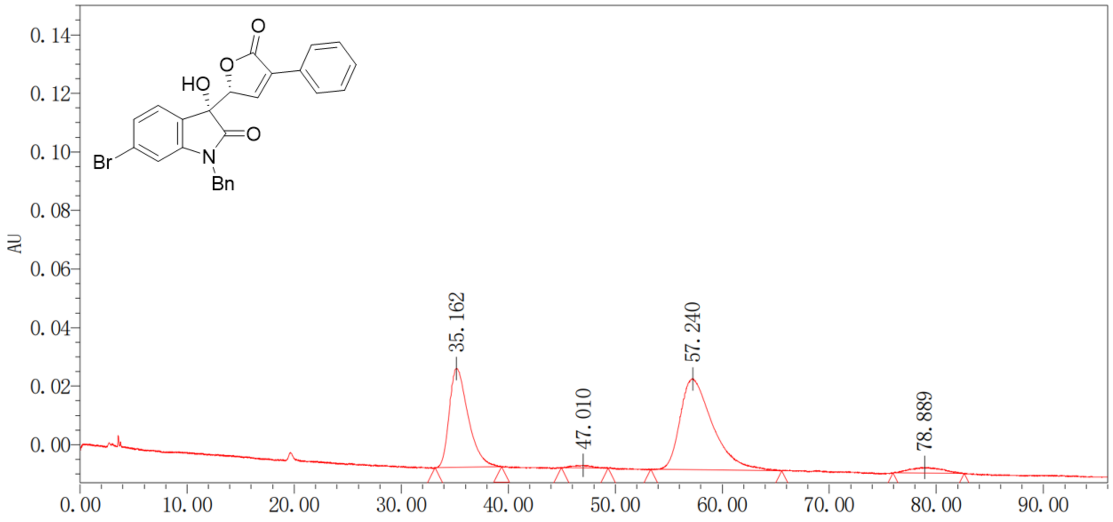

	Name	Ret.time (min)	Area (μV*s)	Height (μV)	%Area
**1**		35.162	4194761	33865	37.24
**2**		47.010	110727	914	0.98
**3**		57.240	6559535	31024	58.24
**4**		78.889	398149	1863	3.53

HPLC spectrum for **3p:**

Condition:hexane:2-propanol=85:15
Flow rate=1.0 mL/min, λ=254 nm, Chiral IA

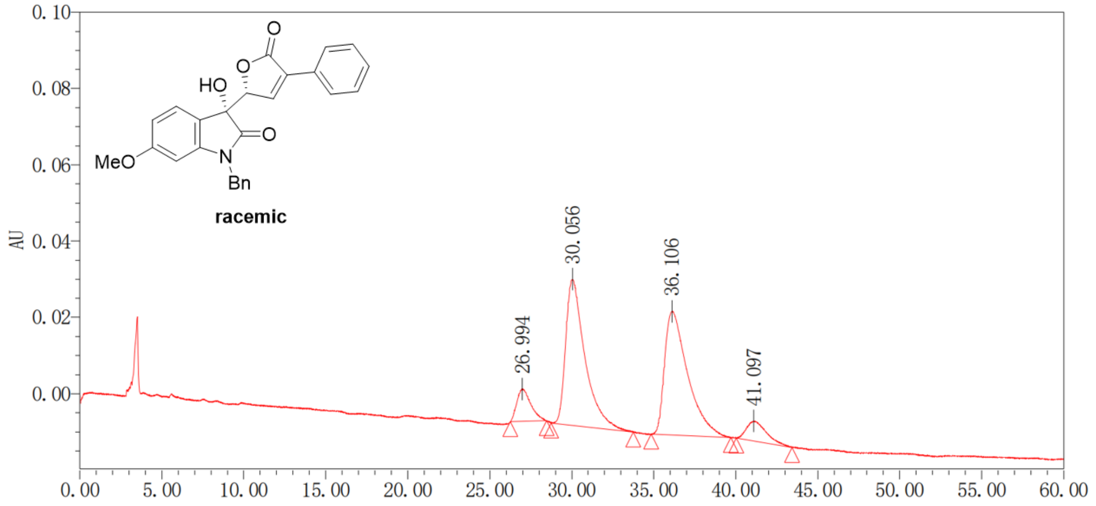

	Name	Ret.time (min)	Area (μV*s)	Height (μV)	%Area
1		26.994	501958	8480	6.91
2		30.056	3150495	38323	43.34
3		36.106	3175081	32454	43.68
4		41.097	441360	5231	6.07

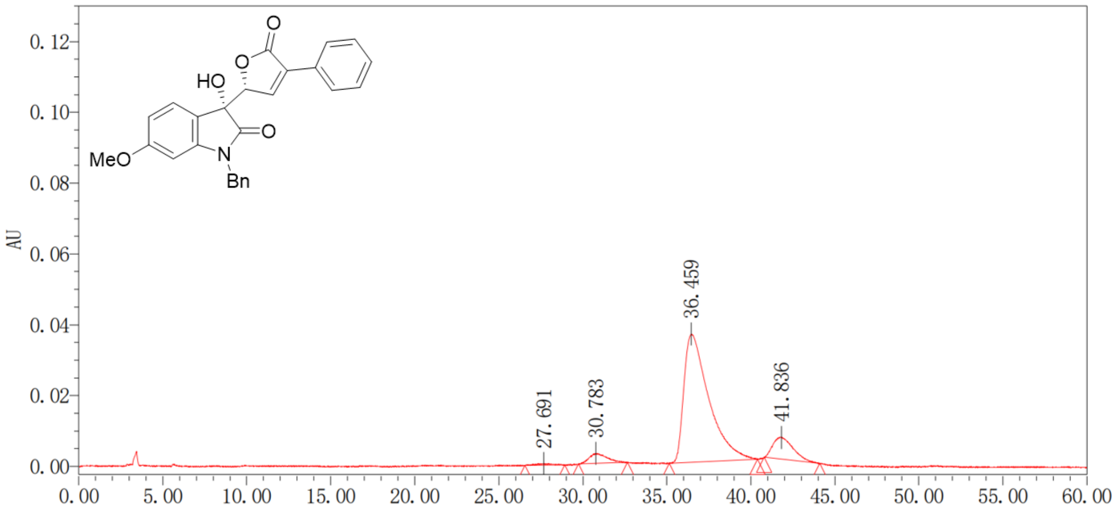

	Name	Ret.time (min)	Area (μV*s)	Height (μV)	%Area
1		27.691	27024	443	0.61
2		30.783	204951	2772	4.60
3		36.459	3712563	36224	83.27
4		41.836	513951	6159	11.53

HPLC spectrum for **3q:**

Condition:hexane:2-propanol=90:10
Flow rate=1.0 mL/min, λ=254 nm, Chiral ADH

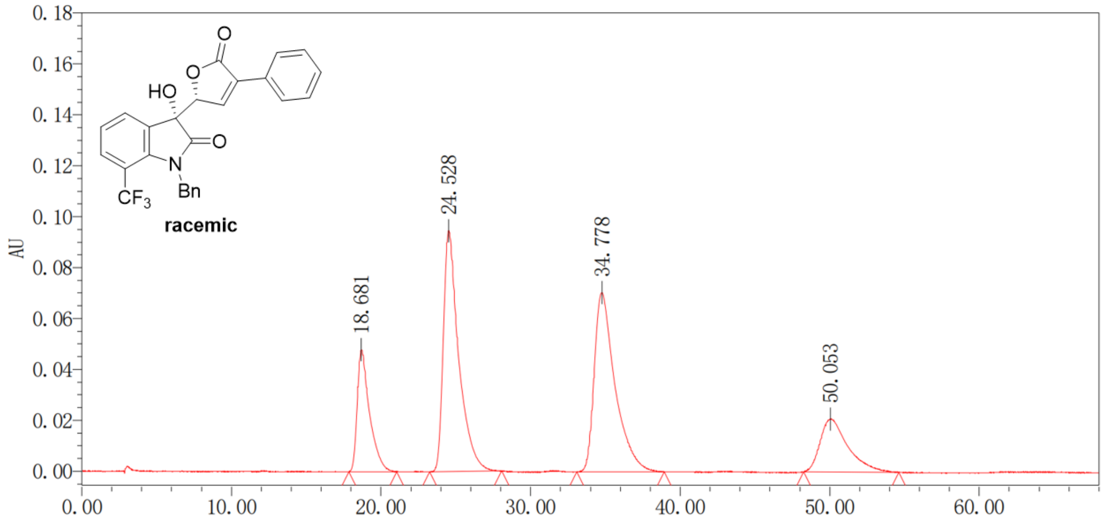

	Name	Ret.time (min)	Area (μV*s)	Height (μV)	%Area
**1**		18.681	2775184	47852	14.56
**2**		24.528	6834381	94663	35.87
**3**		34.778	6768459	70475	35.52
**4**		50.053	2677670	20822	14.05

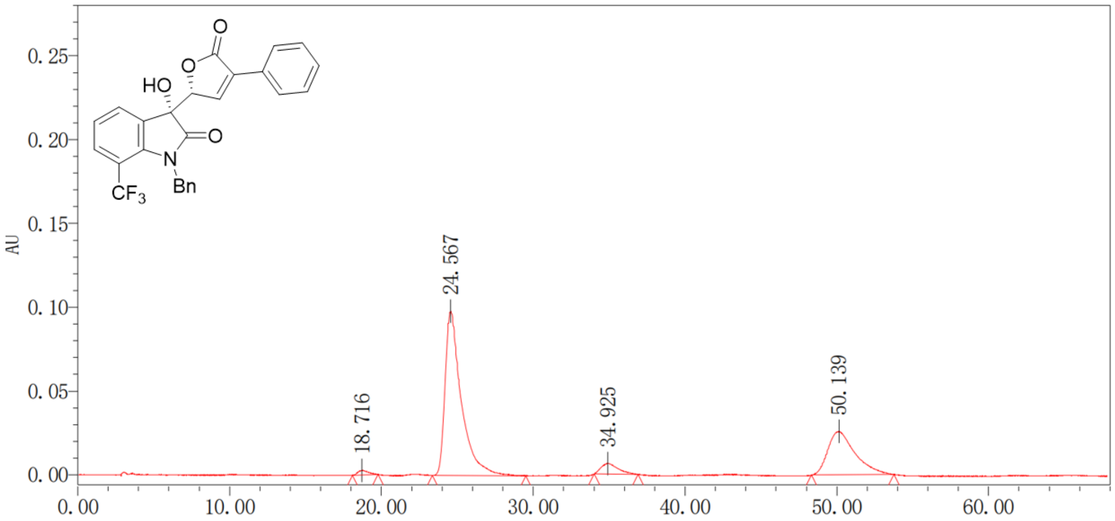

	Name	Ret.time (min)	Area (μV*s)	Height (μV)	%Area
**1**		18.716	132524	2752	1.18
**2**		24.567	7372822	98212	65.76
**3**		34.925	490948	6342	4.38
**4**		50.139	3215619	25865	28.68

HPLC spectrum for **3r:**

Condition:hexane:2-propanol=80:20
Flow rate=1.0 mL/min, λ=254 nm, Chiral ADH

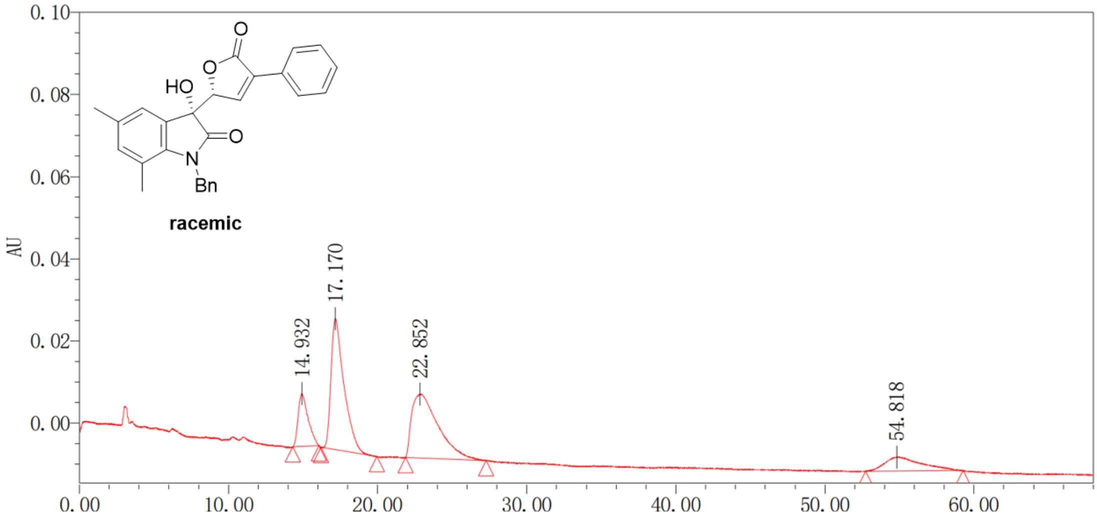

	Name	Ret.time (min)	Area (μV*s)	Height (μV)	%Area
1		14.932	593544	12850	11.91
2		17.170	1914680	31859	38.41
3		22.852	1908898	15534	38.30
4		54.818	567283	3357	11.38

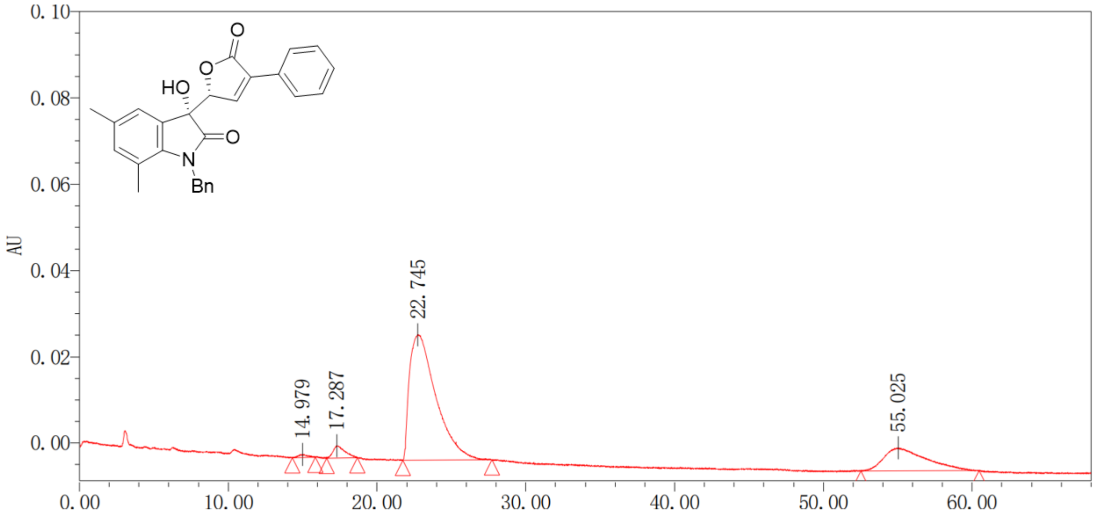

	Name	Ret.time (min)	Area (μV*s)	Height (μV)	%Area
1		14.979	29543	710	0.62
2		17.287	157261	2855	3.28
3		22.745	3566541	29130	74.30
4		55.025	1047069	5331	21.81

HPLC spectrum for **3s:**

Condition:hexane:2-propanol=70:30
Flow rate=1.0 mL/min, λ=254 nm, Chiral IA

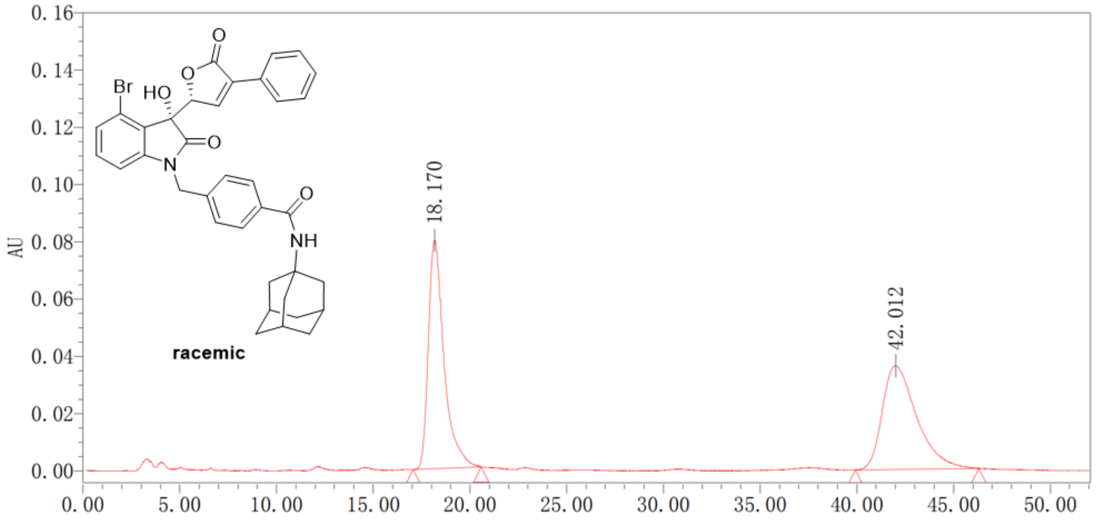

	Name	Ret.time (min)	Area (μV*s)	Height (μV)	%Area
1		18.170	4511351	79822	50.27
2		42.012	4462338	36193	49.73

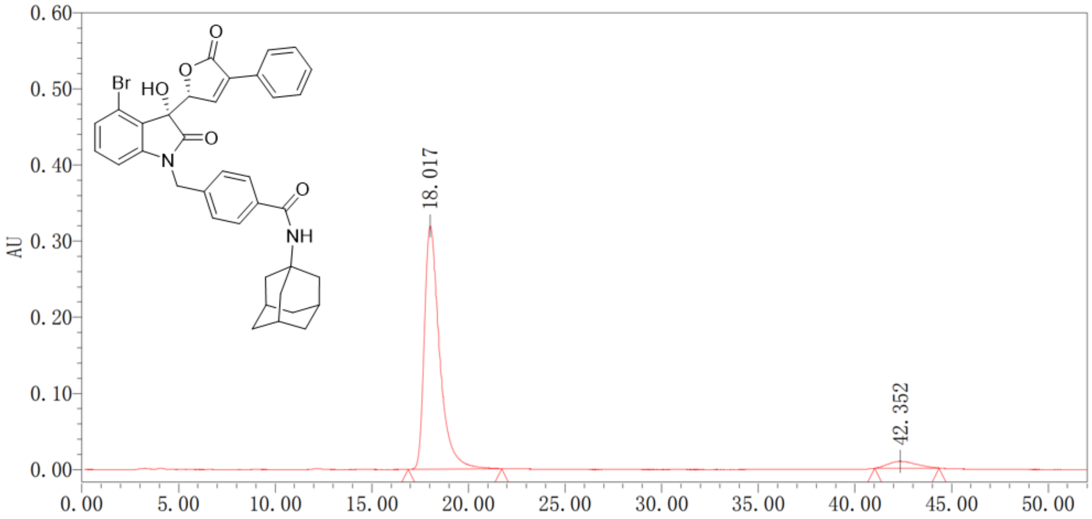

	Name	Ret.time (min)	Area (μV*s)	Height (μV)	%Area
1		18.017	17450414	319346	95.04
2		42.352	911465	8938	4.96

HPLC spectrum for **3v:**

Condition:hexane:2-propanol=85:15
Flow rate=1.0 mL/min, λ=254 nm, Chiral IC

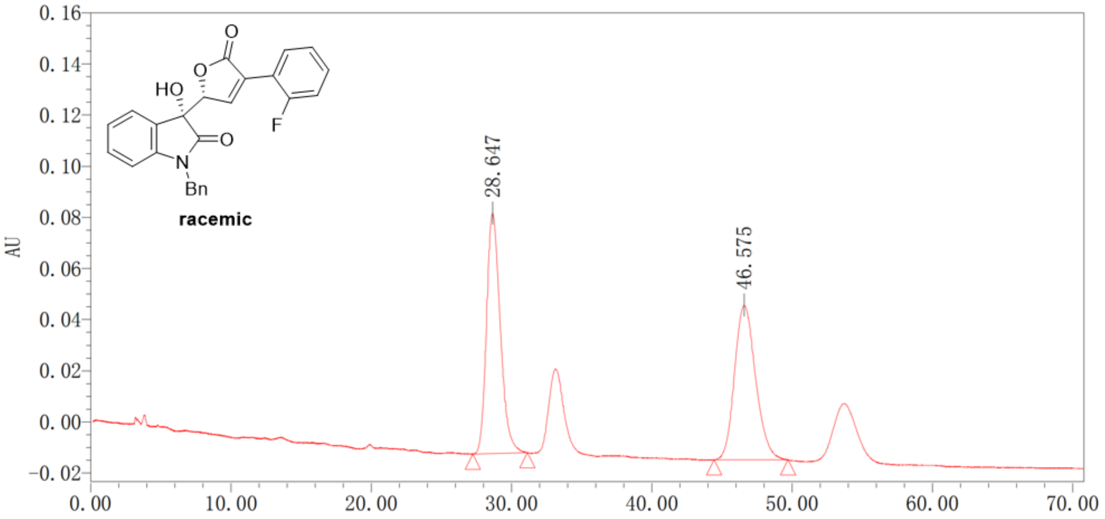

	Name	Ret.time (min)	Area (μV*s)	Height (μV)	%Area
**1**		28.647	6400392	93999	50.33
**2**		46.575	6316453	60537	49.67

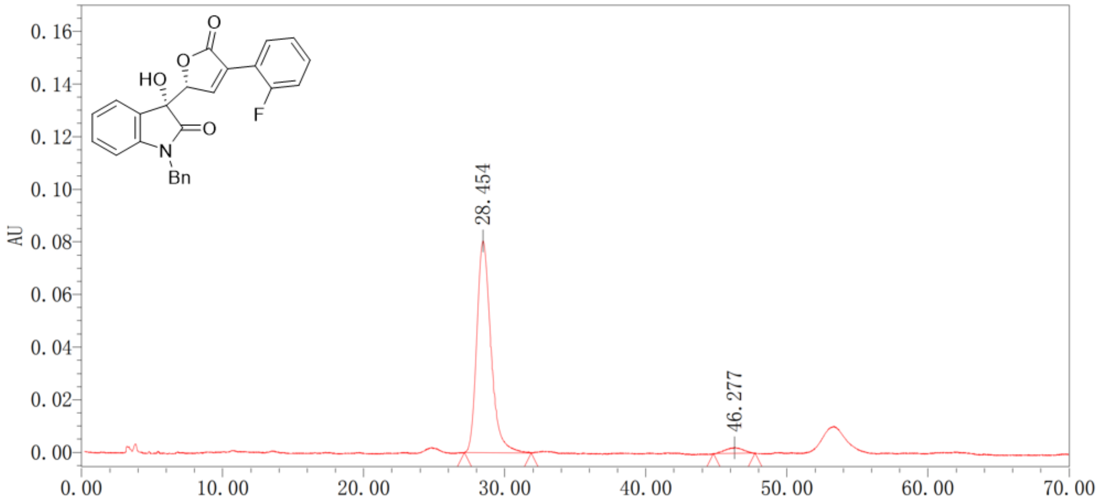

	Name	Ret.time (min)	Area (μV*s)	Height (μV)	%Area
**1**		28.454	5555651	80585	96.80
**2**		46.277	183806	2209	3.20

HPLC spectrum for **3w:**

Condition:hexane:2-propanol=75:25
Flow rate=1.0 mL/min, λ=254 nm, Chiral ODH

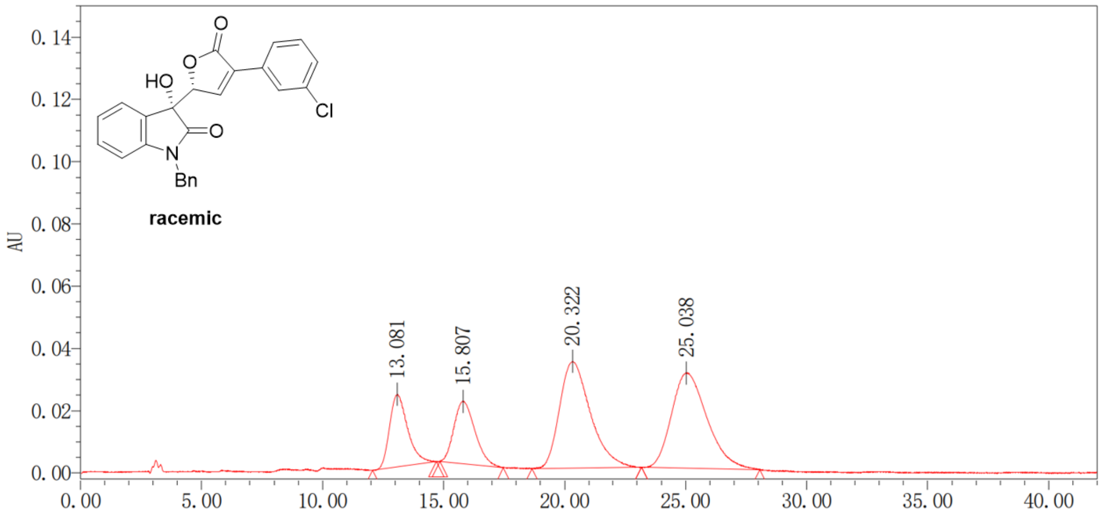

	Name	Ret.time (min)	Area (μV*s)	Height (μV)	%Area
1		13.081	1213994	23266	14.13
2		15.807	1207554	20069	14.13
3		20.322	3026952	34240	35.22
4		25.038	3144827	30670	36.60

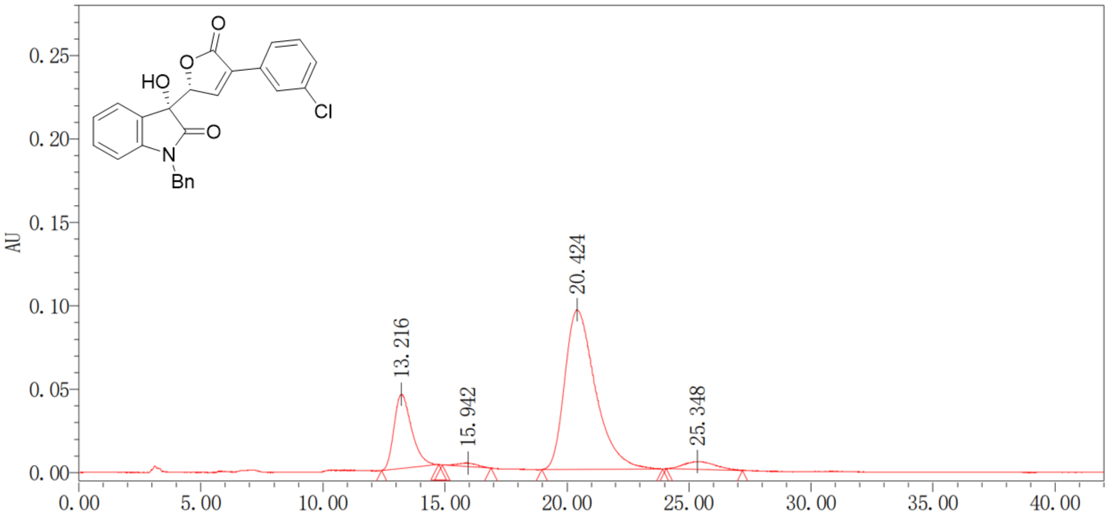

	Name	Ret.time (min)	Area (μV*s)	Height (μV)	%Area
1		13.216	2248908	44463	20.16
2		15.942	120507	2175	1.08
3		20.424	8376258	95565	75.10
4		25.348	407175	4593	3.65

HPLC spectrum for **3x:**

Condition:hexane:2-propanol=80:20
Flow rate=1.0 mL/min, λ=254 nm, Chiral IC

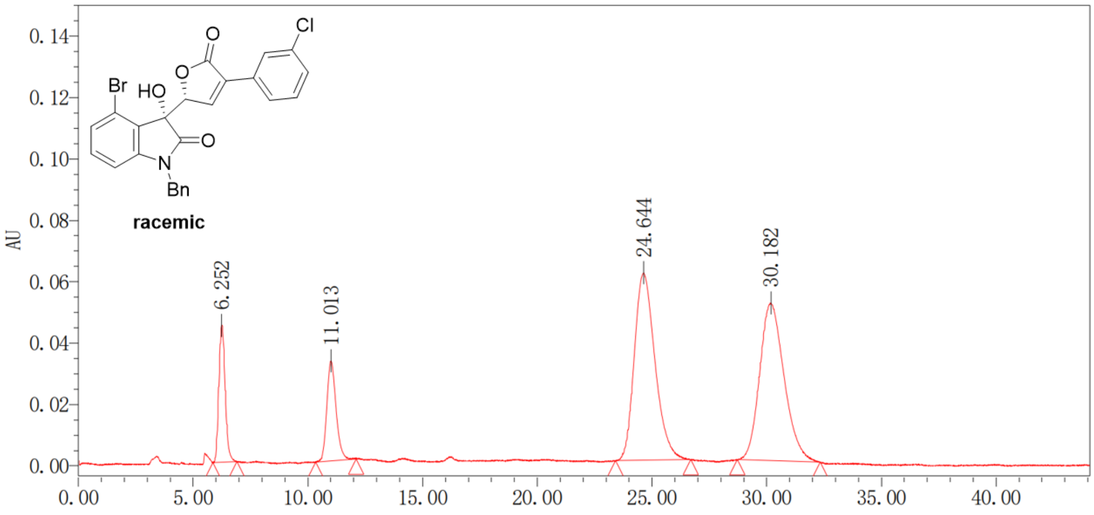

	Name	Ret.time (min)	Area (μV*s)	Height (μV)	%Area
1		6.252	872370	44631	9.28
2		11.013	901491	32553	9.59
3		24.644	3798378	61052	40.42
4		30.182	3825277	51341	40.71

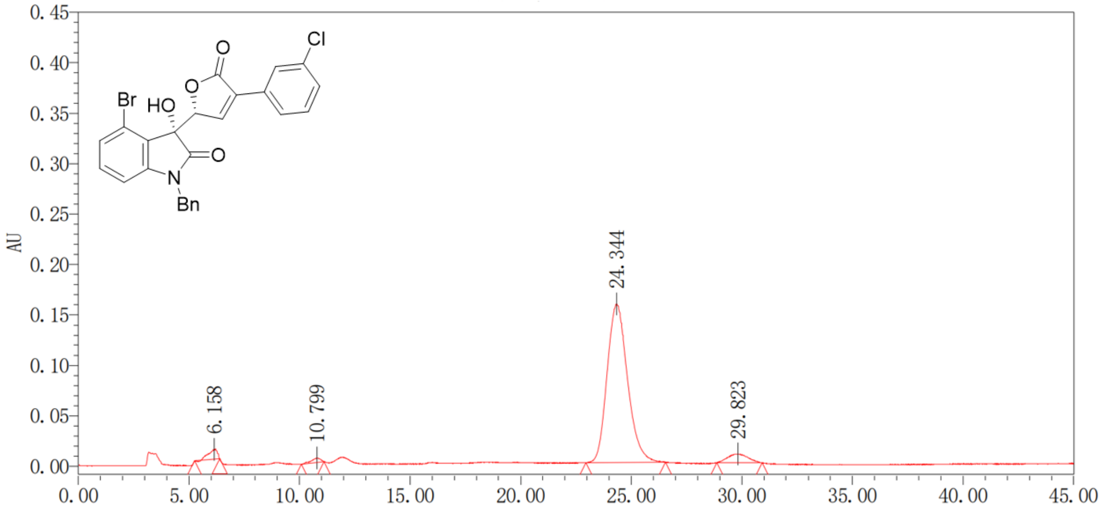

	Name	Ret.time (min)	Area (μV*s)	Height (μV)	%Area
1		6.158	299073	9906	2.68
2		10.799	129289	4332	1.16
3		24.344	10178711	157058	91.22
4		29.823	551566	8669	4.94

HPLC spectrum for **3y:**

Condition:hexane:2-propanol=93:7
Flow rate=1.0 mL/min, λ=254 nm, Chiral IC

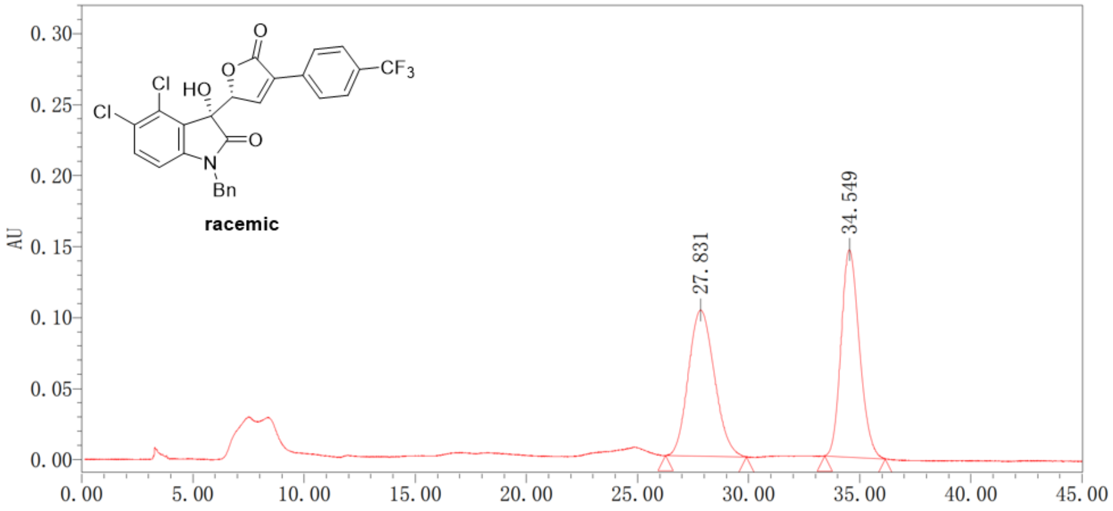

	Name	Ret.time (min)	Area (μV*s)	Height (μV)	%Area
1		27.831	8353877	102896	49.88
2		34.549	8394282	146199	50.12

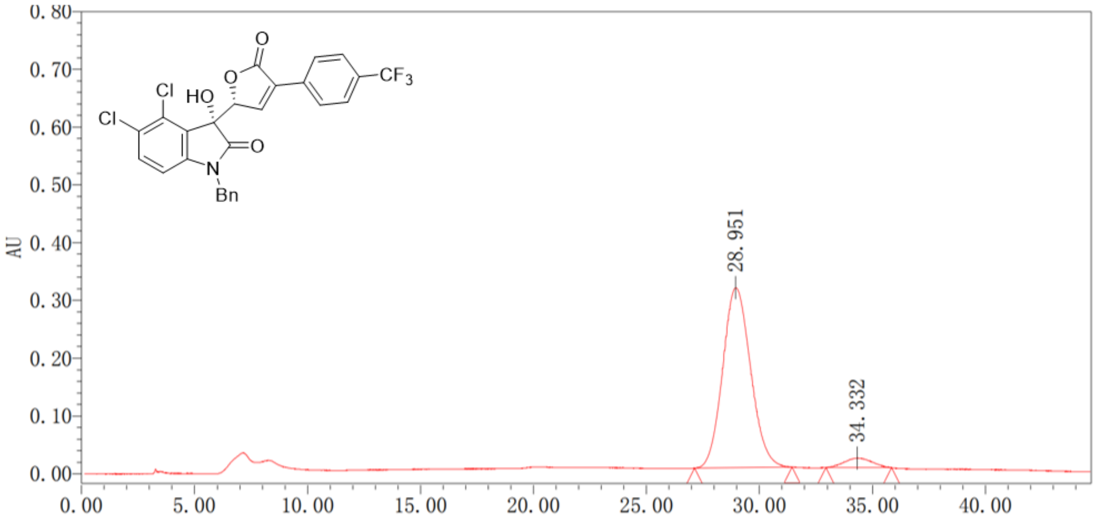

	Name	Ret.time (min)	Area (μV*s)	Height (μV)	%Area
1		28.951	26835623	311602	95.00
2		34.332	1411029	16544	5.00

HPLC spectrum for **3z:**

Condition:hexane:2-propanol=75:25
Flow rate=1.0 mL/min, λ=254 nm, Chiral ODH

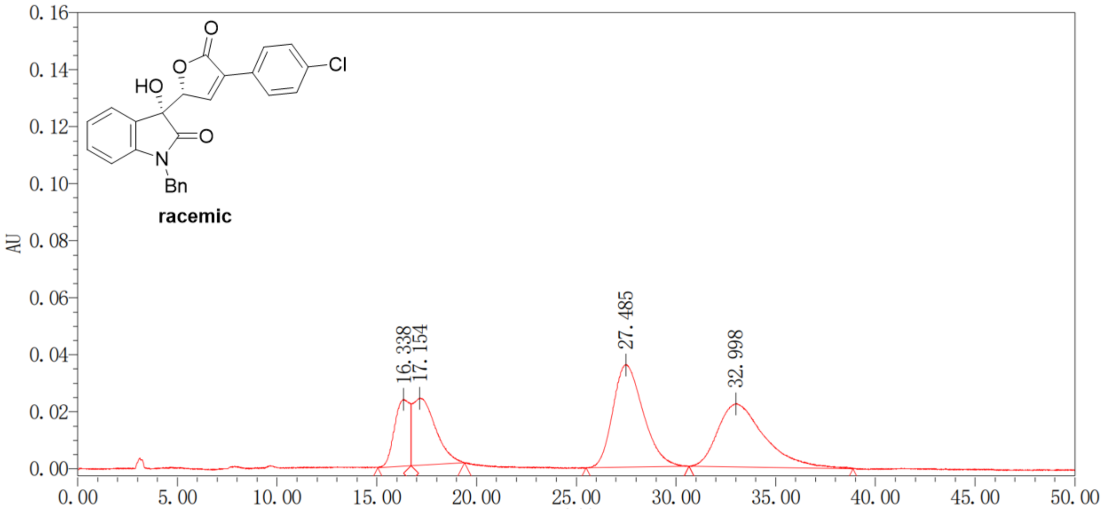

	Name	Ret. time (min)	Area (μV*s)	Height (μV)	%Area
**1**		16.338	1259041	23361	11.94
**2**		17.154	1865992	23461	17.69
**3**		27.485	3809866	35960	36.13
**4**		32.998	3610821	22150	34.24

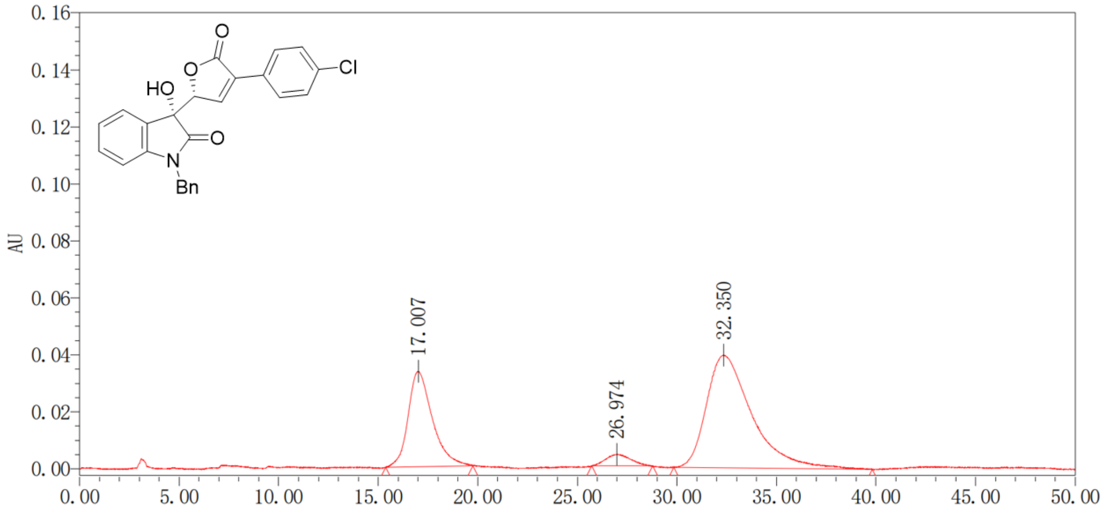

	Name	Ret. time (min)	Area (μV*s)	Height (μV)	%Area
**1**		17.007	2808129	33400	29.74
**2**		26.974	344613	4035	3.65
**3**		32.350	6289923	39562	66.61

HPLC spectrum for **3aa:**

Condition:hexane:2-propanol=80:20
Flow rate=1.0 mL/min, λ=254 nm, Chiral IC

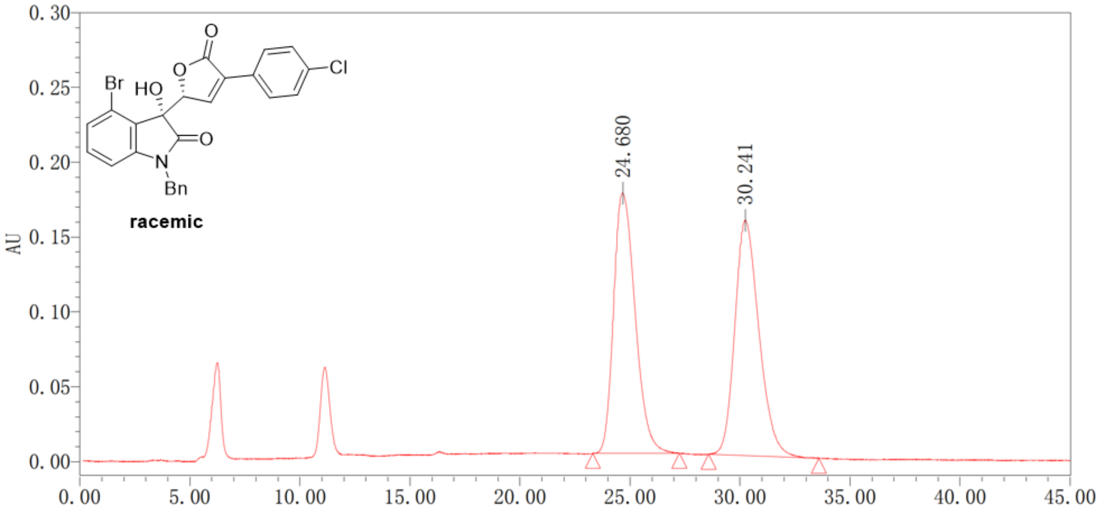

	Name	Ret.time (min)	Area (μV*s)	Height (μV)	%Area
1		24.680	11863203	173892	49.65
2		30.241	12030568	157221	50.35

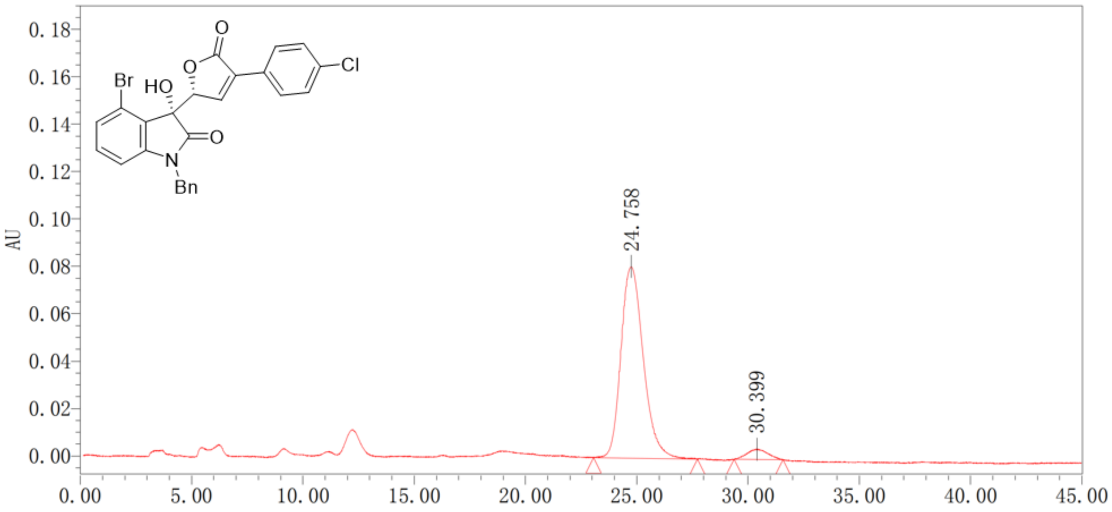

	Name	Ret.time (min)	Area (μV*s)	Height (μV)	%Area
1		24.758	5651081	80891	95.31
2		30.399	277931	4361	4.69

HPLC spectrum for **3ab:**

Condition:hexane:2-propanol=82:18
Flow rate=1.0 mL/min, λ=254 nm, Chiral ADH

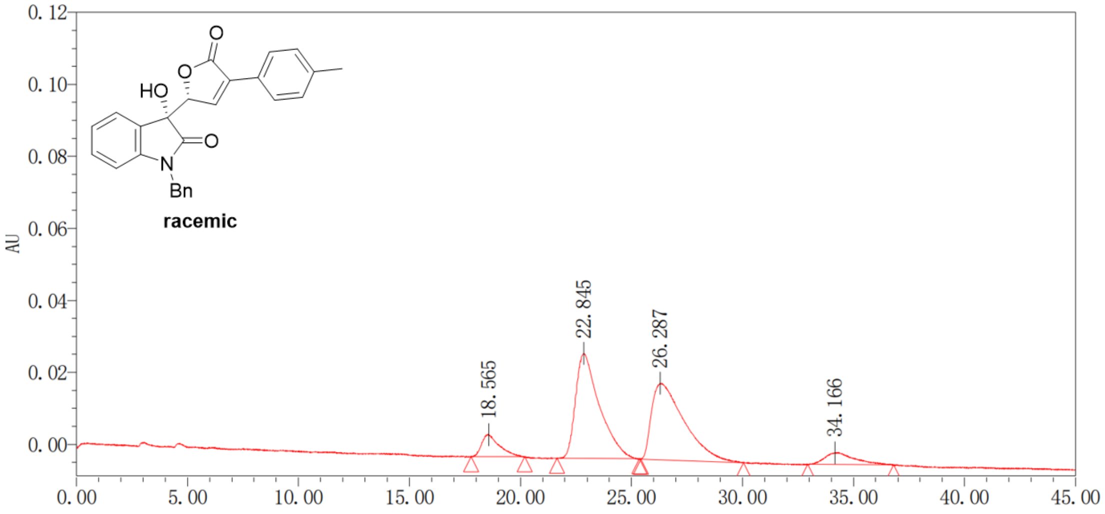

	Name	Ret. time (min)	Area (μV*s)	Height (μV)	%Area
1		18.565	341047	6150	6.82
2		22.845	2190845	29118	43.80
3		26.287	2161444	21197	43.21
4		34.166	309144	3324	6.18

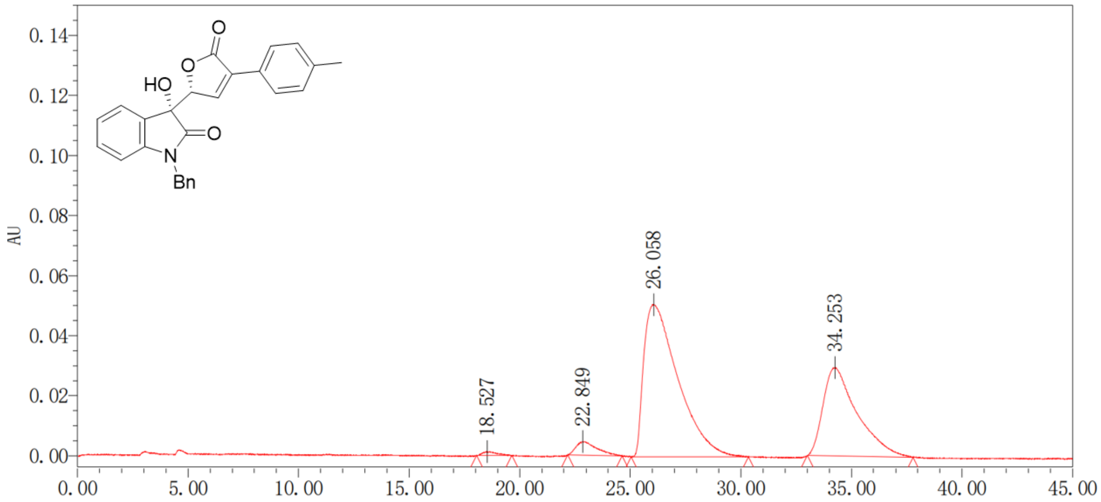

	Name	Ret. time (min)	Area (μV*s)	Height (μV)	%Area
1		18.527	60935	1367	0.68
2		22.849	296867	4534	3.32
3		26.058	5463238	50675	61.01
4		34.253	3133754	29450	35.00

HPLC spectrum for **3ac:**

Condition:hexane:2-propanol=75:25
Flow rate=1.0 mL/min, λ=254 nm, Chiral ADH

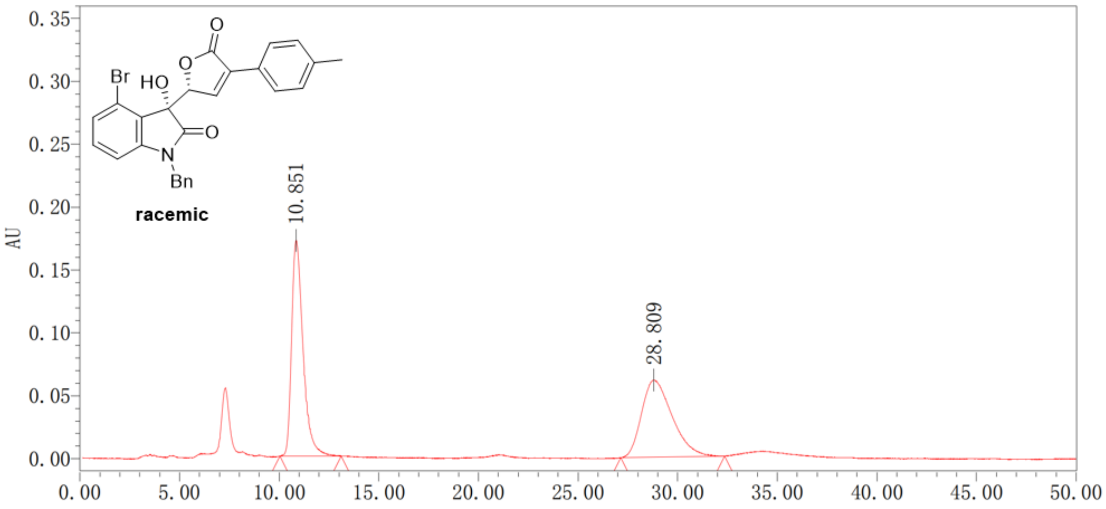

	Name	Ret.time (min)	Area (μV*s)	Height (μV)	%Area
1		10.851	6777547	171582	50.90
2		28.809	6538518	61274	49.10

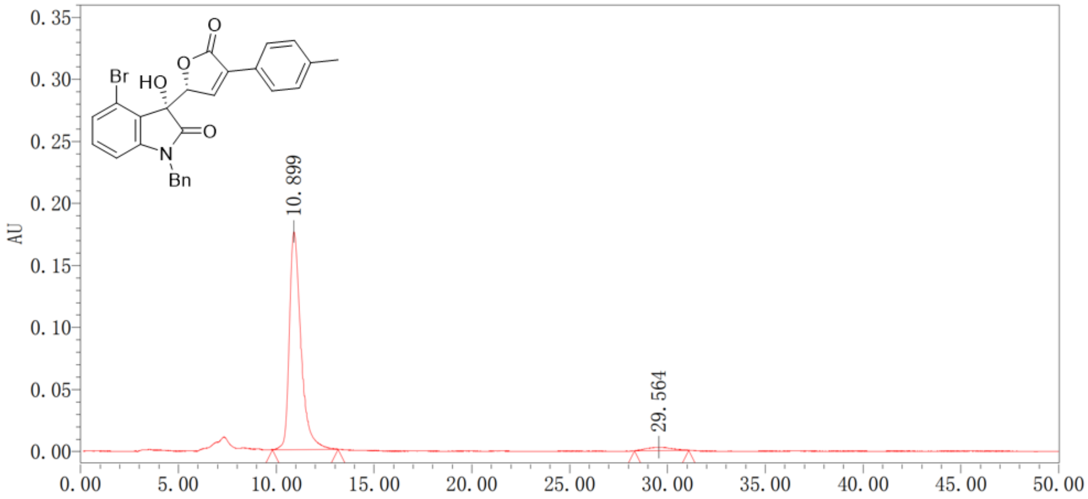

	Name	Ret.time (min)	Area (μV*s)	Height (μV)	%Area
1		10.899	7177376	175577	96.93
2		29.564	227285	2656	3.07

HPLC spectrum for **3ad:**

Condition:hexane:2-propanol=70:30
Flow rate=1.0 mL/min, λ=254 nm, Chiral ODH

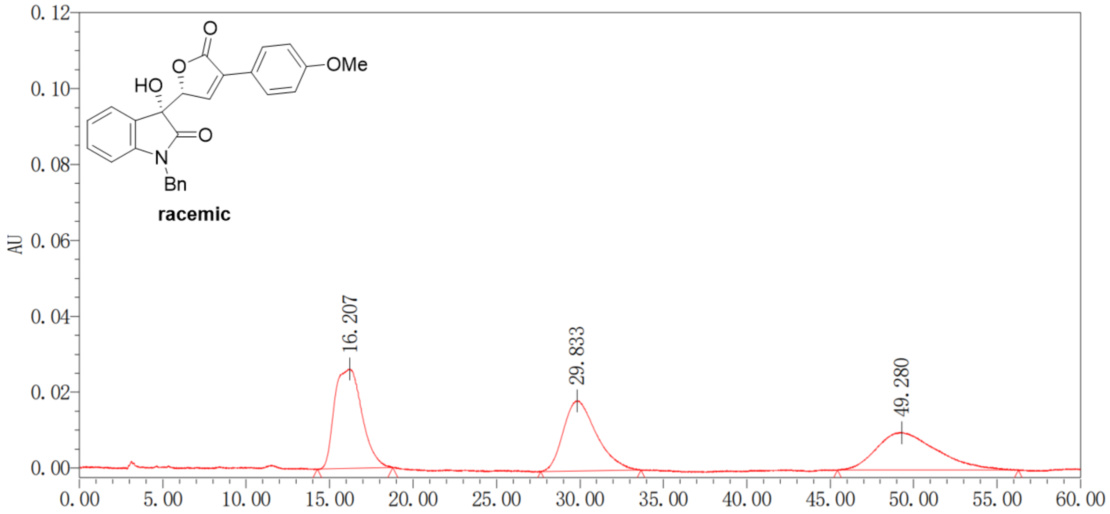

	Name	Ret. time (min)	Area (μV*s)	Height (μV)	%Area
**1**		16.207	3002950	26231	37.23
**2**		29.833	2552267	18466	31.64
**3**		49.280	2511371	9905	31.13

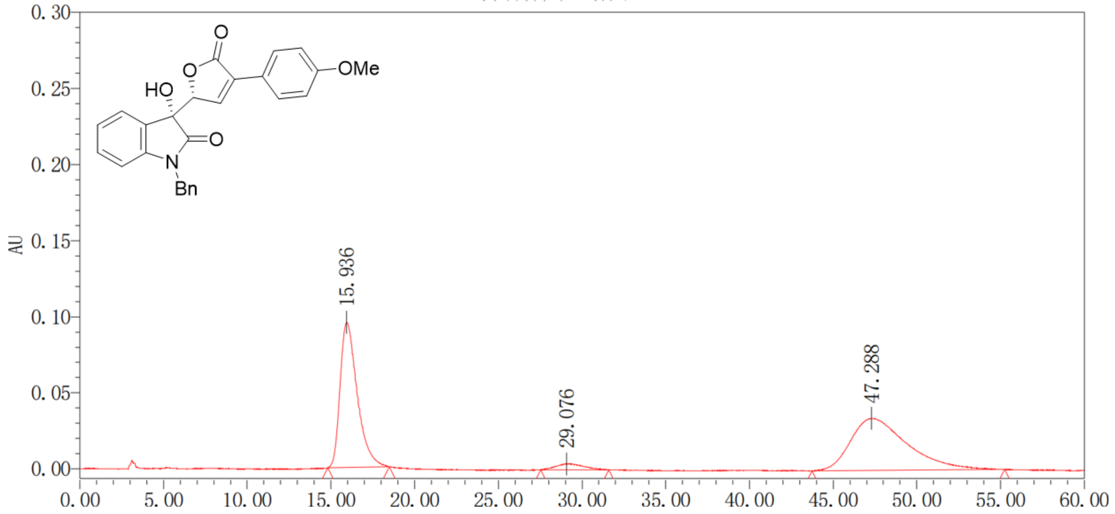

	Name	Ret. time (min)	Area (μV*s)	Height (μV)	%Area
**1**		15.936	6795778	95379	44.47
**2**		29.076	435295	3809	2.85
**3**		47.288	8052055	9905	52.69

HPLC spectrum for **3ae:**

Condition:hexane:2-propanol=75:25
Flow rate=1.0 mL/min, λ=254 nm, Chiral ODH

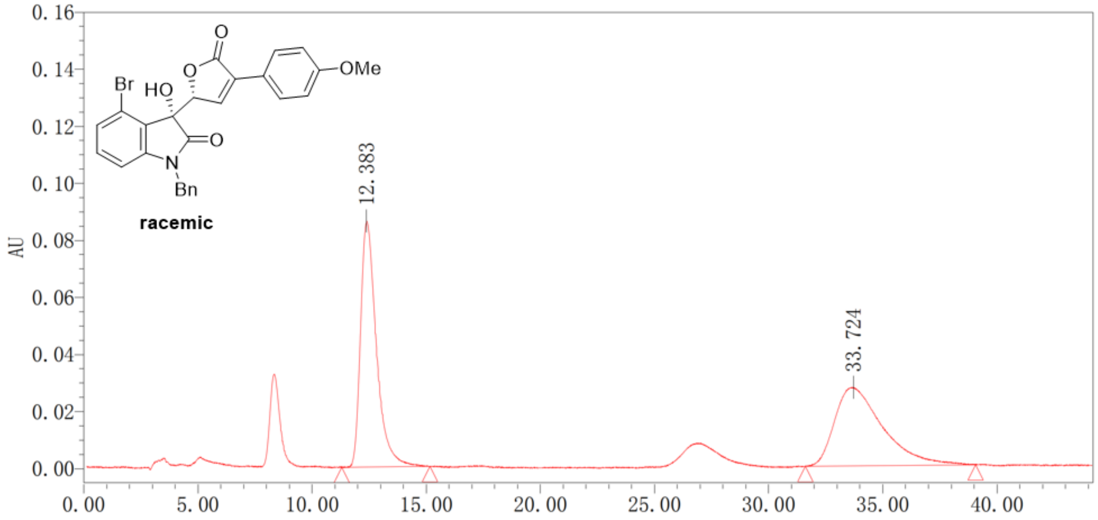

	Name	Ret.time (min)	Area (μV*s)	Height (μV)	%Area
**1**		12.383	4137432	86204	50.75
**2**		33.724	4015014	27513	49.25

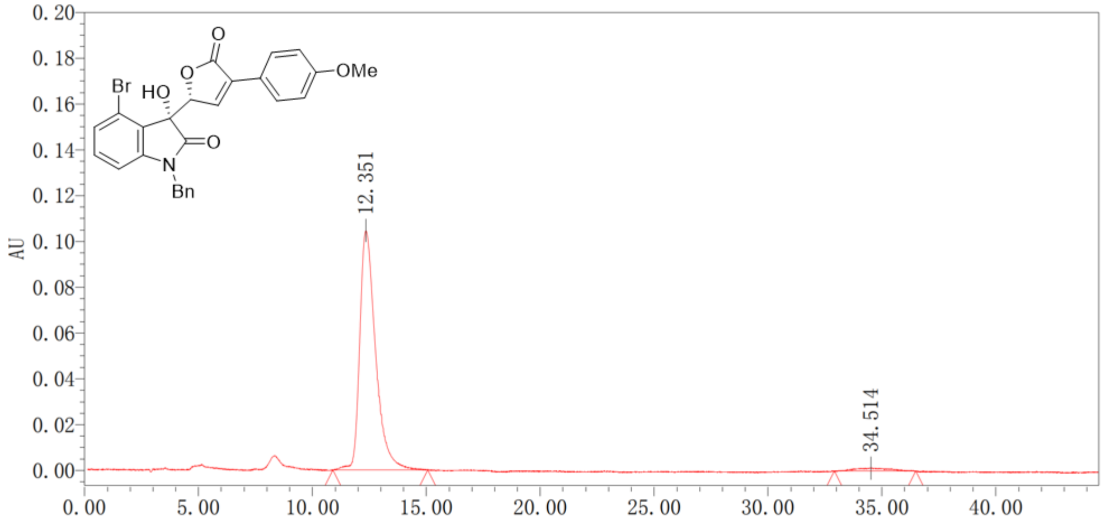

	Name	Ret.time (min)	Area (μV*s)	Height (μV)	%Area
**1**		12.351	5004075	104549	97.48
**2**		34.514	129414	1315	2.52

HPLC spectrum for **3af:**

Condition:hexane:2-propanol=85:15
Flow rate=1.0 mL/min, λ=254 nm, Chiral IC

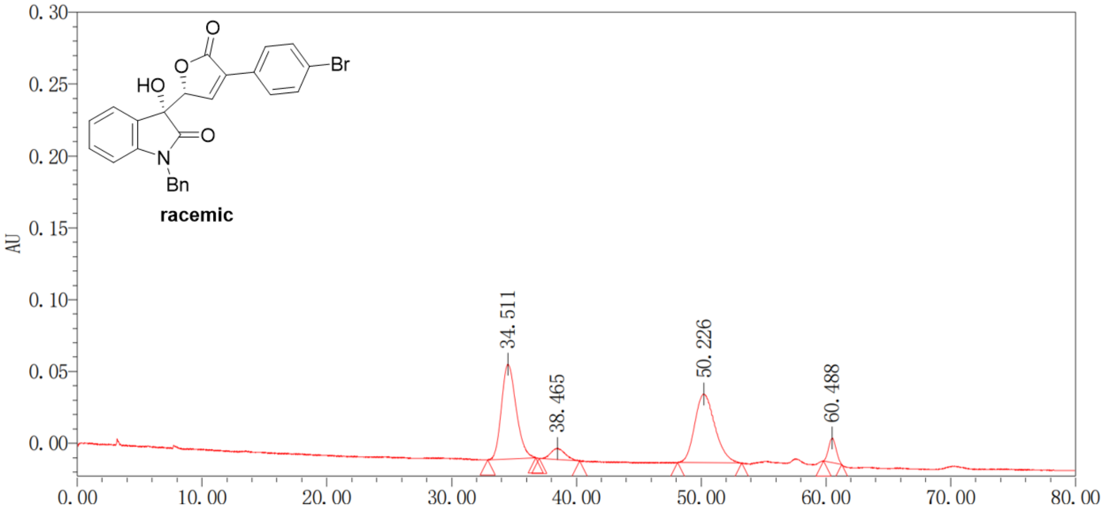

	Name	Ret. time (min)	Area (μV*s)	Height (μV)	%Area
1		34.511	5319379	65920	44.18
2		38.465	623526	7762	5.18
3		50.226	5392497	47844	44.79
4		60.488	704113	17014	5.85

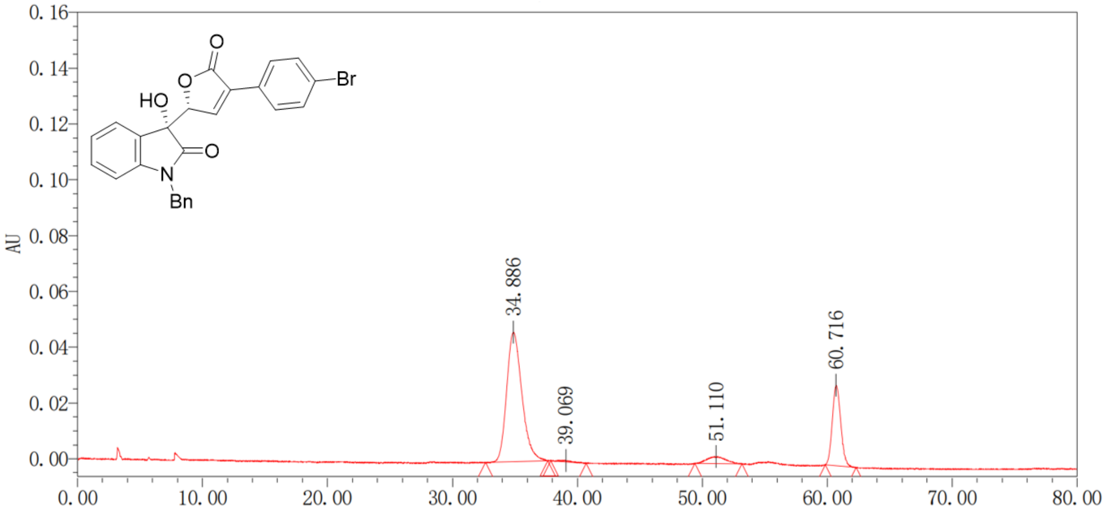

	Name	Ret. time (min)	Area (μV*s)	Height (μV)	%Area
1		34.886	3902724	46483	70.38
2		39.069	25536	497	0.46
3		51.110	273882	2587	4.94
4		60.716	1342868	28846	24.22

HPLC spectrum for **3ag:**

Condition:hexane:2-propanol=85:15
Flow rate=1.0 mL/min, λ=254 nm, Chiral ODH

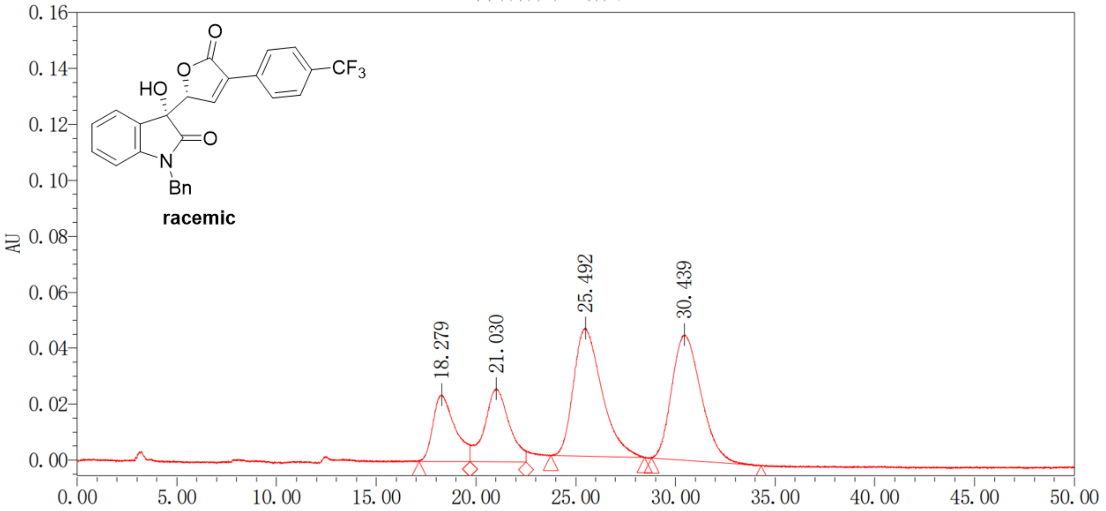

	Name	Ret. time (min)	Area (µV*s)	Height (µV)	%Area
1		18.279	1828493	23877	13.91
2		21.030	2244502	26279	17.07
3		25.492	4518832	45739	34.37
4		30.439	4556727	44834	34.66

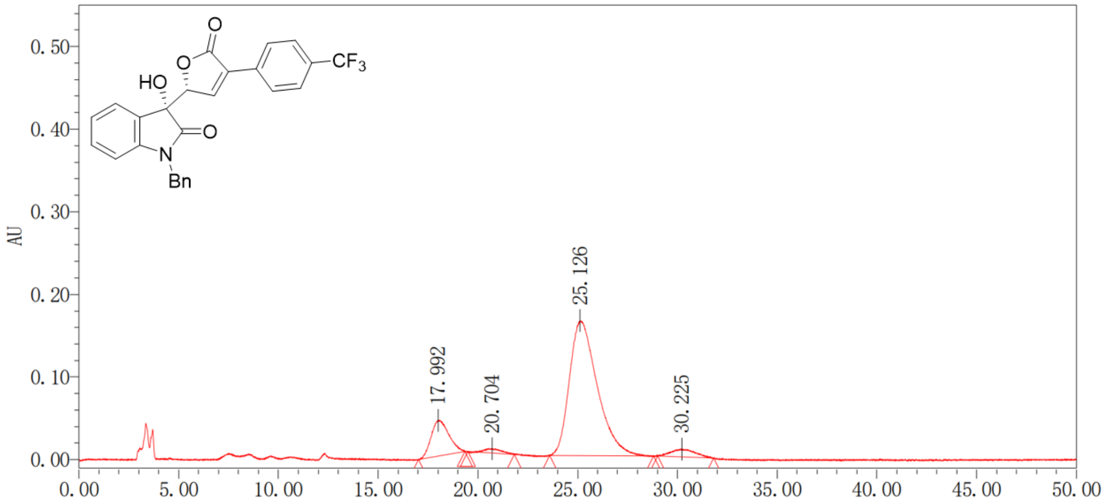

	Name	Ret. time (min)	Area (µV*s)	Height (µV)	%Area
1		17.992	2660161	43314	13.47
2		20.704	351966	5585	1.78
3		25.126	15959470	163484	80.81
4		30.225	776848	9322	3.93

HPLC spectrum for **3ah:**

Condition:hexane:2-propanol=75:25
Flow rate=1.0 mL/min, λ=254 nm, Chiral ADH

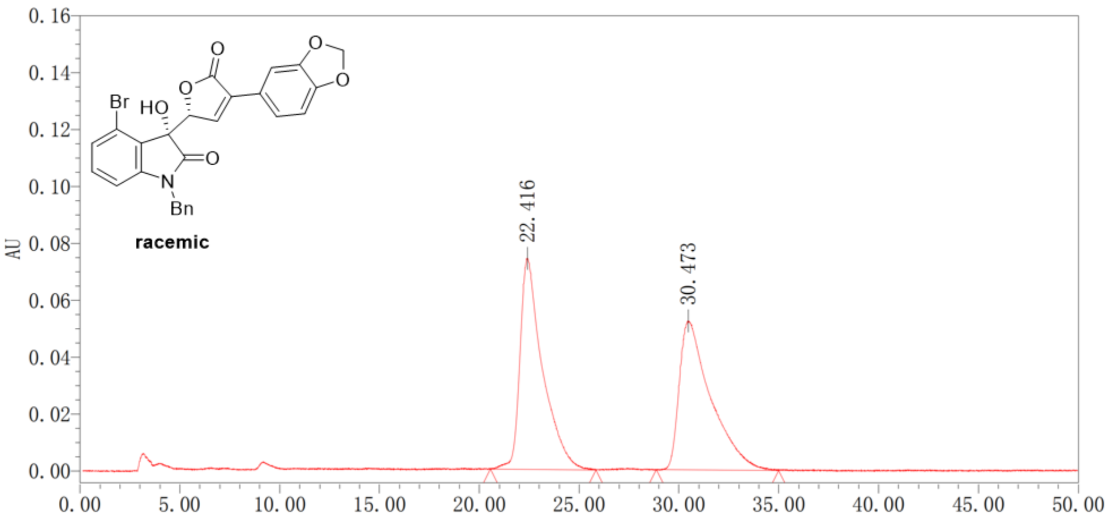

	Name	Ret.time (min)	Area (μV*s)	Height (μV)	%Area
1		22.416	5855994	74105	50.48
2		30.473	5744697	52368	49.52

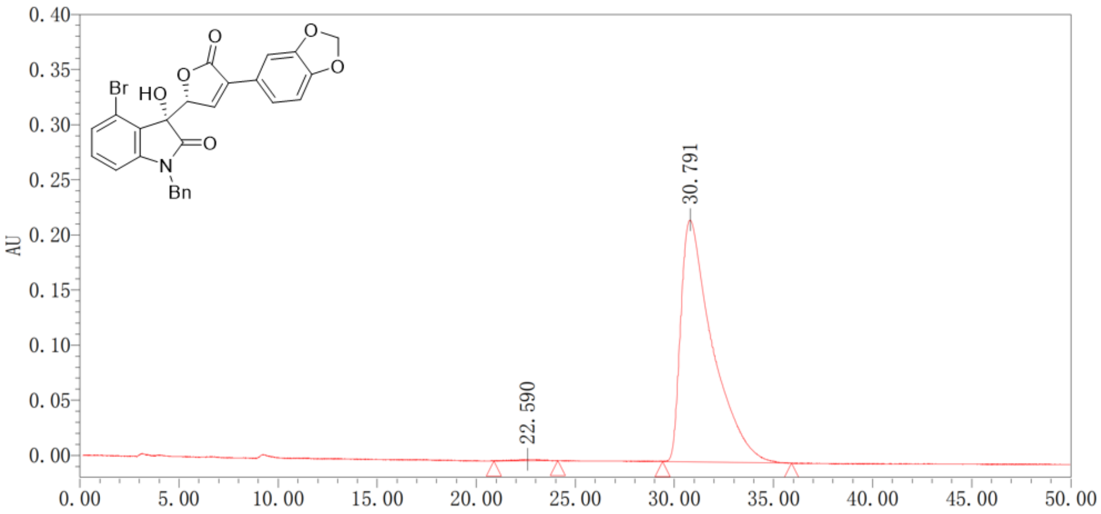

	Name	Ret.time (min)	Area (μV*s)	Height (μV)	%Area
1		22.590	104339	1313	0.43
2		30.791	24304813	219570	99.57

HPLC spectrum for **3ai:**

Condition:hexane:2-propanol=70:30
Flow rate=1.0 mL/min, λ=254 nm, Chiral ODH

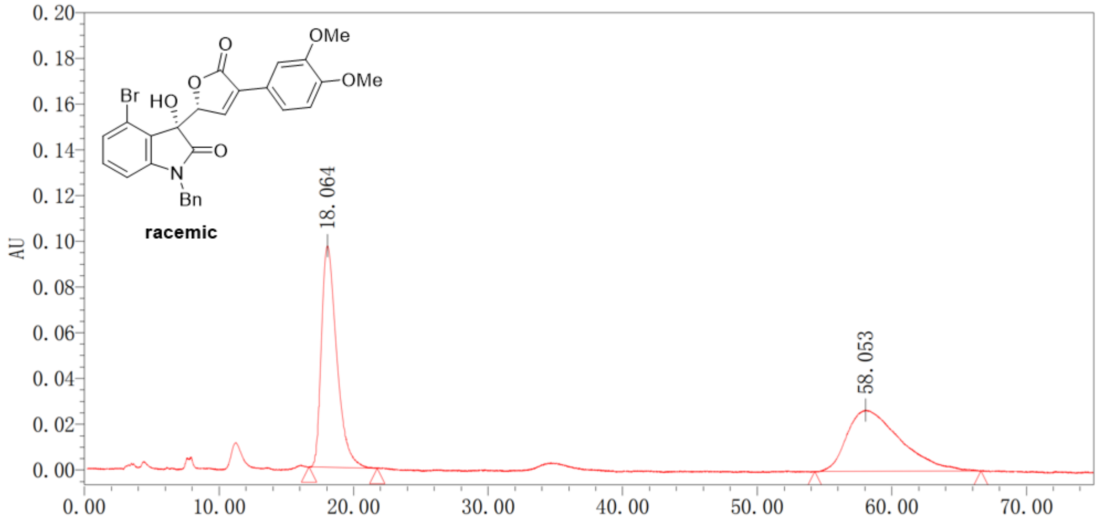

	Name	Ret.time (min)	Area (μV*s)	Height (μV)	%Area
1		18.064	7577633	96884	50.77
2		58.053	7347130	26799	49.23

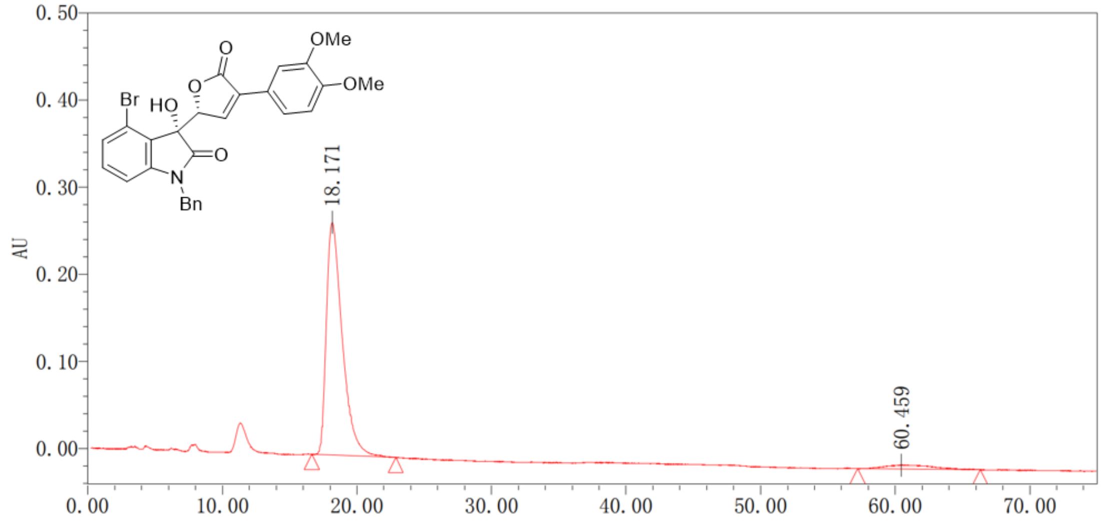

	Name	Ret.time (min)	Area (μV*s)	Height (μV)	%Area
1		18.171	21752886	266972	95.31
2		60.459	1069401	4325	4.69

HPLC spectrum for **3aj:**

Condition:hexane:2-propanol=70:30
Flow rate=1.0 mL/min, λ=254 nm, Chiral ODH

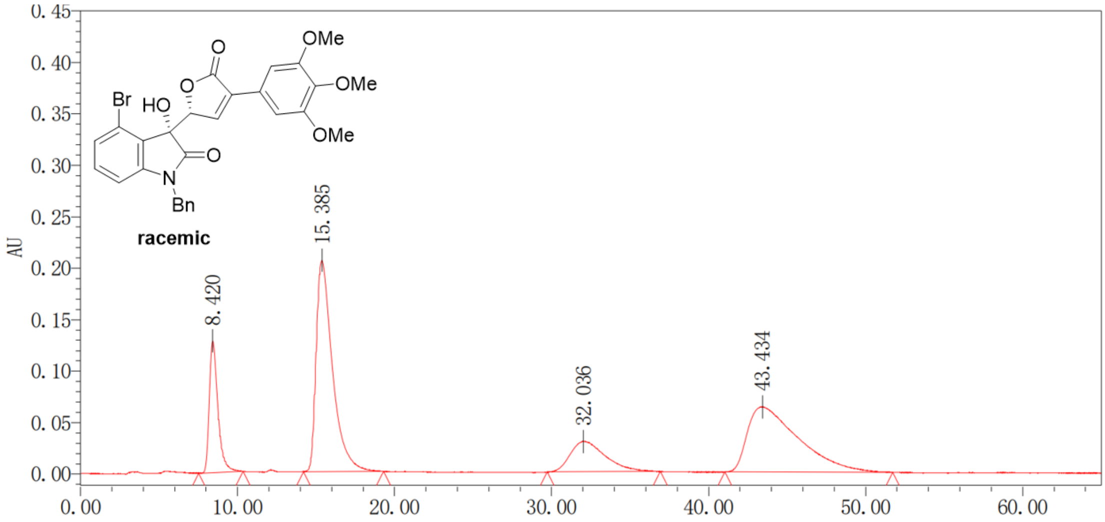

	Name	Ret.time (min)	Area (μV*s)	Height (μV)	%Area
**1**		8.420	4868311	127983	12.62
**2**		15.385	14797073	205316	38.36
**3**		32.036	4618304	29611	11.97
**4**		43.434	14290649	63361	37.05

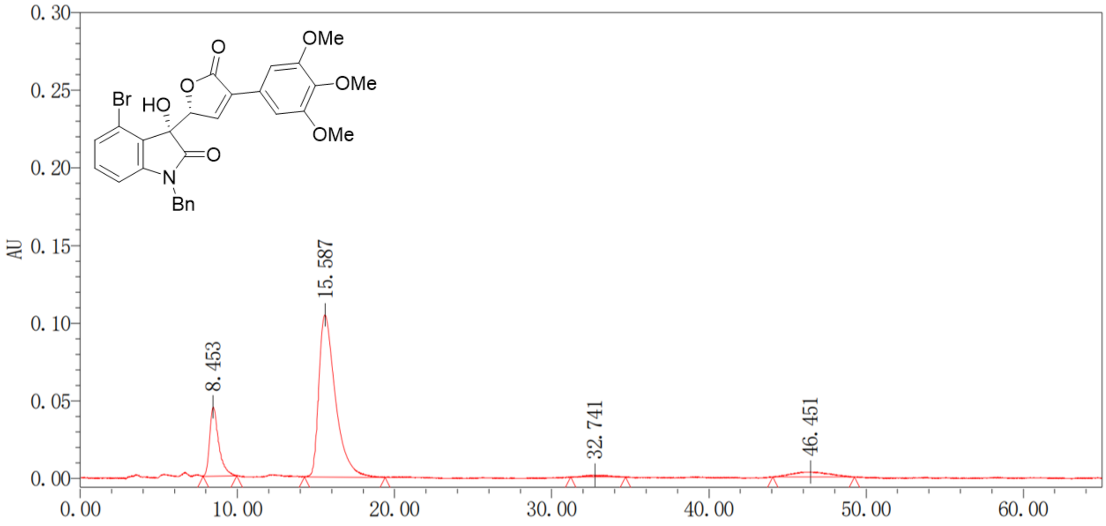

	Name	Ret.time (min)	Area (μV*s)	Height (μV)	%Area
**1**		8.453	1781729	44491	17.85
**2**		15.587	7606529	104388	76.20
**3**		32.741	123094	1177	1.23
**4**		46.451	471523	3097	4.72

HPLC spectrum for **3ak:**

Condition:hexane:2-propanol=78:22
Flow rate=1.0 mL/min, λ=254 nm, Chiral ADH

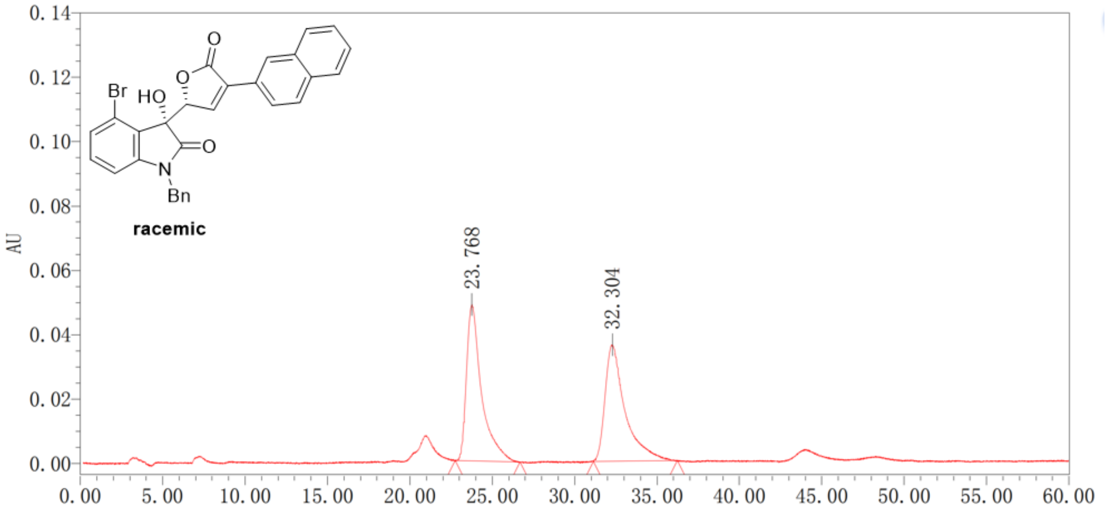

	Name	Ret.time (min)	Area (µV*s)	Height (µV)	%Area
1		23.768	3058532	48536	50.10
2		32.304	3046175	36049	49.90

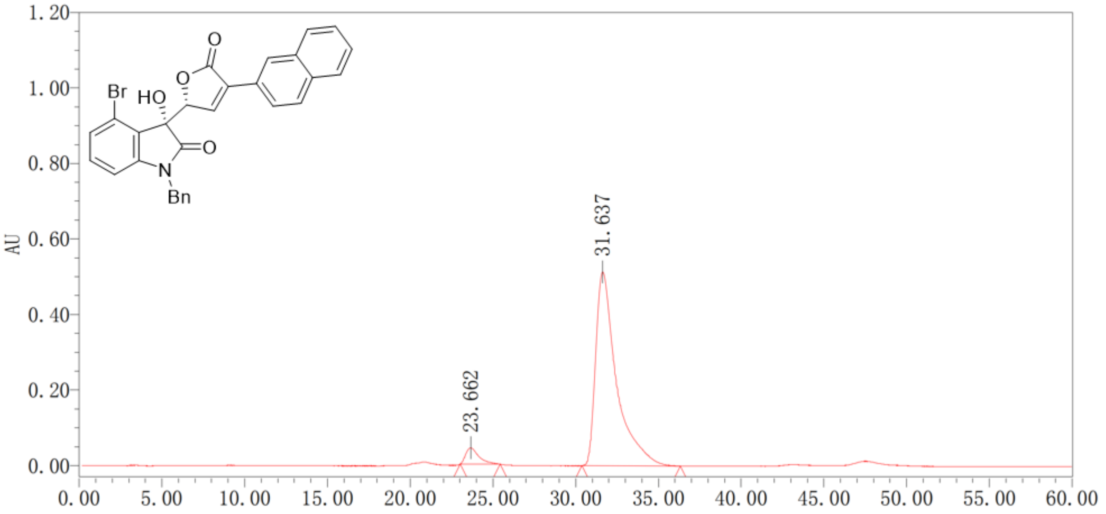

	Name	Ret.time (min)	Area (µV*s)	Height (µV)	%Area
1		23.662	2382911	43305	5.07
2		31.637	44588716	513643	94.93

HPLC spectrum for **3al:**

Condition:hexane:2-propanol=70:30
Flow rate=1.0 mL/min, λ=254 nm, Chiral ODH

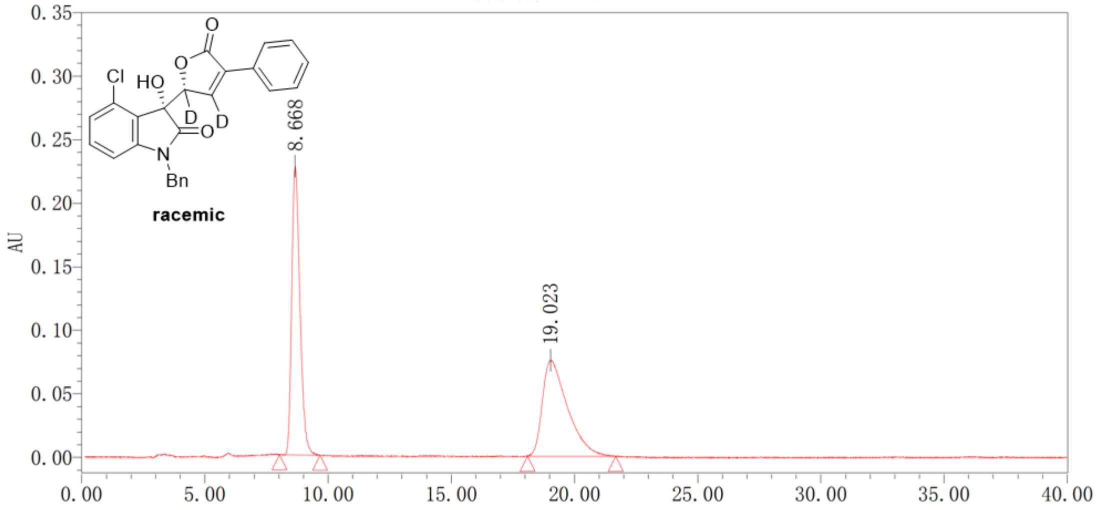

	Name	Ret.time (min)	Area (μV*s)	Height (μV)	%Area
**1**		8.668	5189419	227138	49.23
**2**		19.023	5352017	75380	50.77

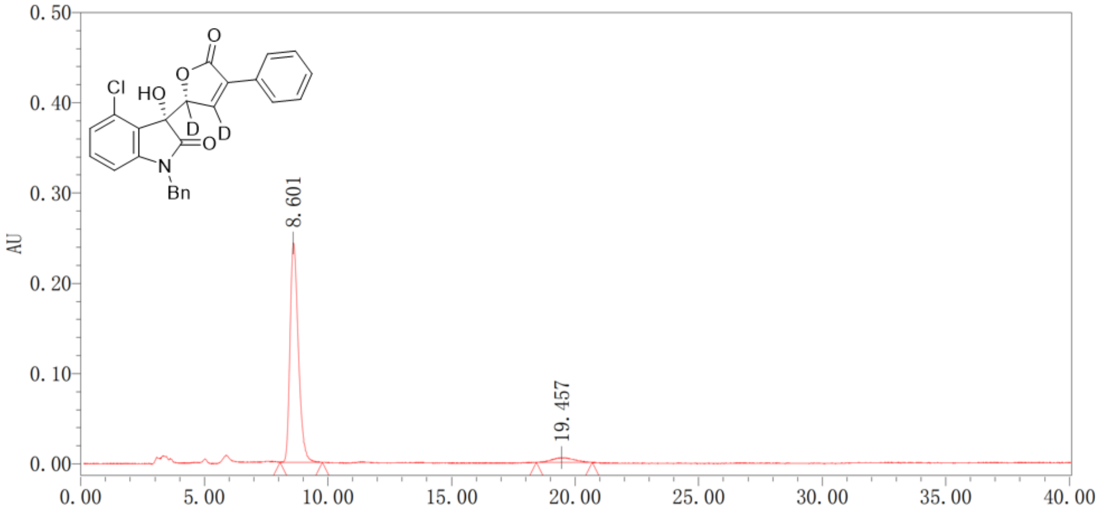

	Name	Ret.time (min)	Area (μV*s)	Height (μV)	%Area
**1**		8.601	5611297	242842	94.83
**2**		19.457	305958	5102	5.17

HPLC spectrum for **3am:**

Condition:hexane:2-propanol=85:15
Flow rate=1.0 mL/min, λ=254 nm, Chiral IA

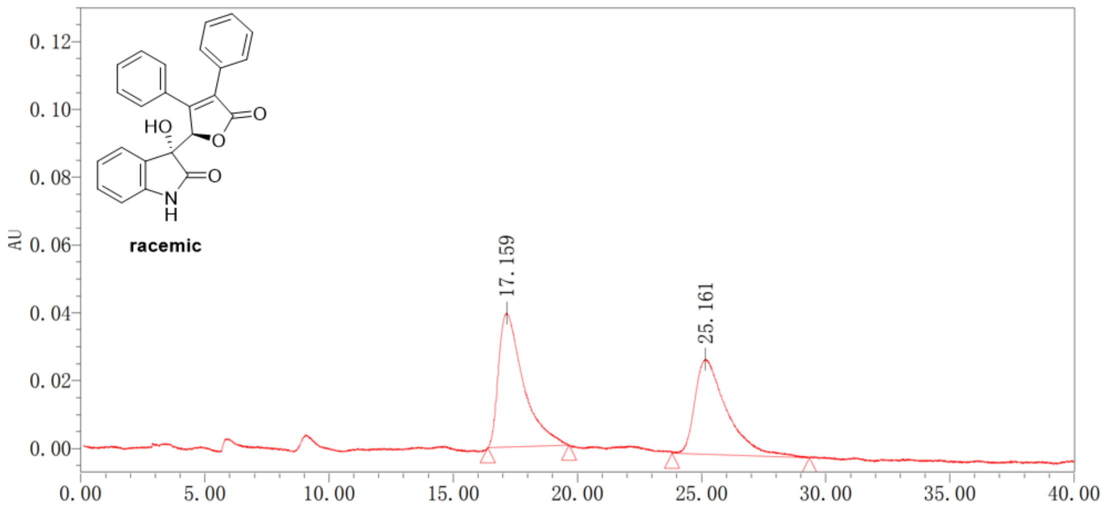

	Name	Ret.time (min)	Area (μV*s)	Height (μV)	%Area
1		17.159	2752048	39607	50.55
2		25.161	2691870	28076	49.45

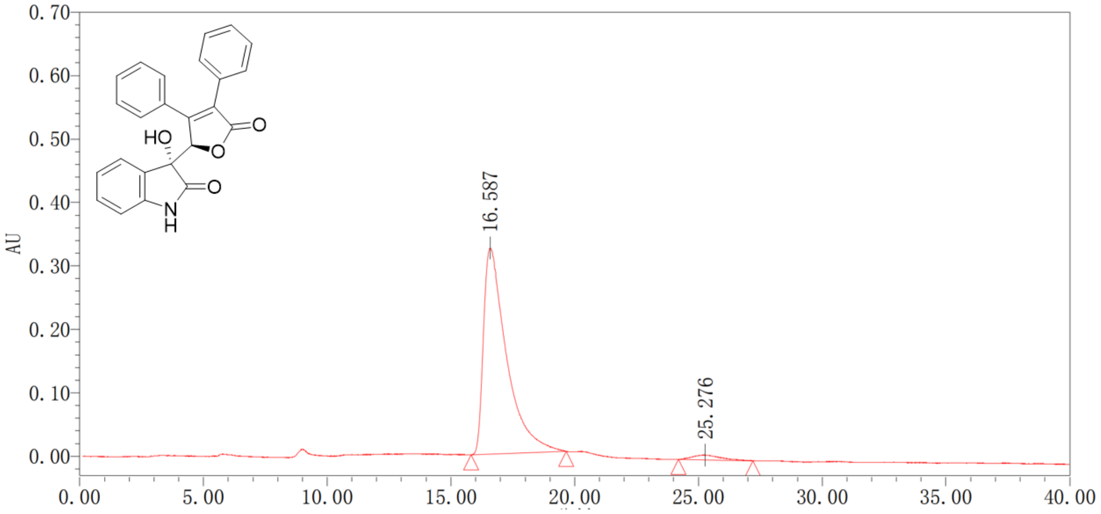

	Name	Ret.time (min)	Area (μV*s)	Height (μV)	%Area
1		16.587	21102326	324760	97.34
2		25.276	576624	7445	2.66

HPLC spectrum for **3an:**

Condition:hexane:2-propanol=80:20
Flow rate=1.0 mL/min, λ=254 nm, Chiral ADH

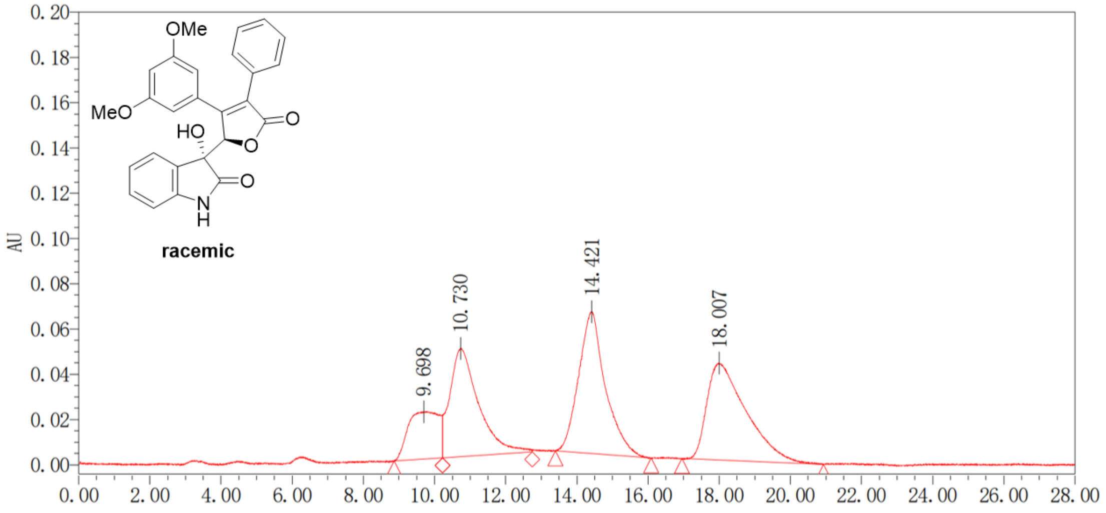

	Name	Ret.time (min)	Area (μV*s)	Height (μV)	%Area
1		9.698	1270537	20998	12.08
2		10.730	2674737	47805	25.43
3		14.421	3268503	62746	31.07
4		18.007	3305820	42836	31.43

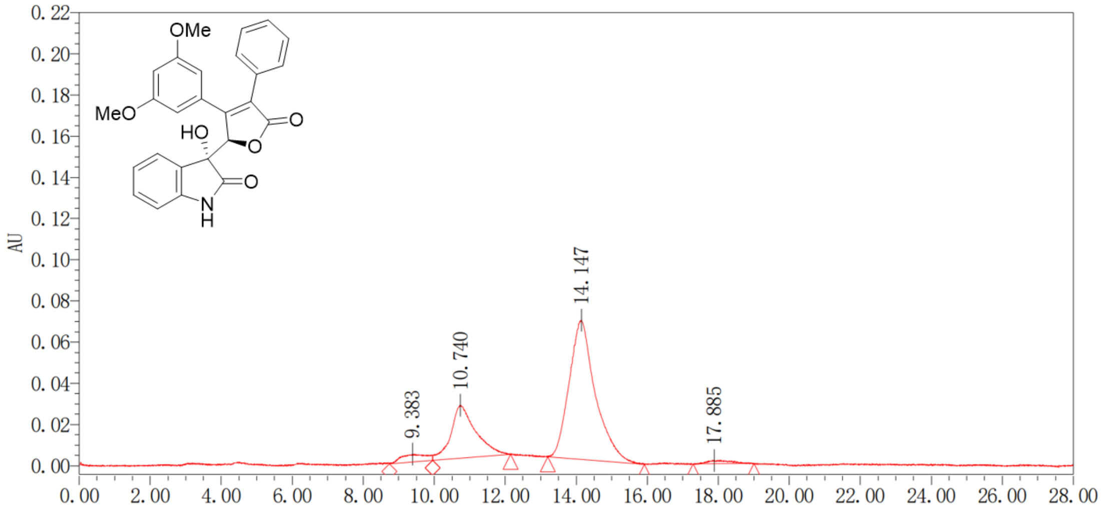

	Name	Ret.time (min)	Area (μV*s)	Height (μV)	%Area
1		9.383	185642	3849	3.73
2		10.740	1250397	25665	25.13
3		14.147	3469623	67745	69.72
4		17.885	70926	1564	1.43

HPLC spectrum for **3ao:**

Condition:hexane:2-propanol=80:20
Flow rate=1.0 mL/min, λ=254 nm, Chiral IA

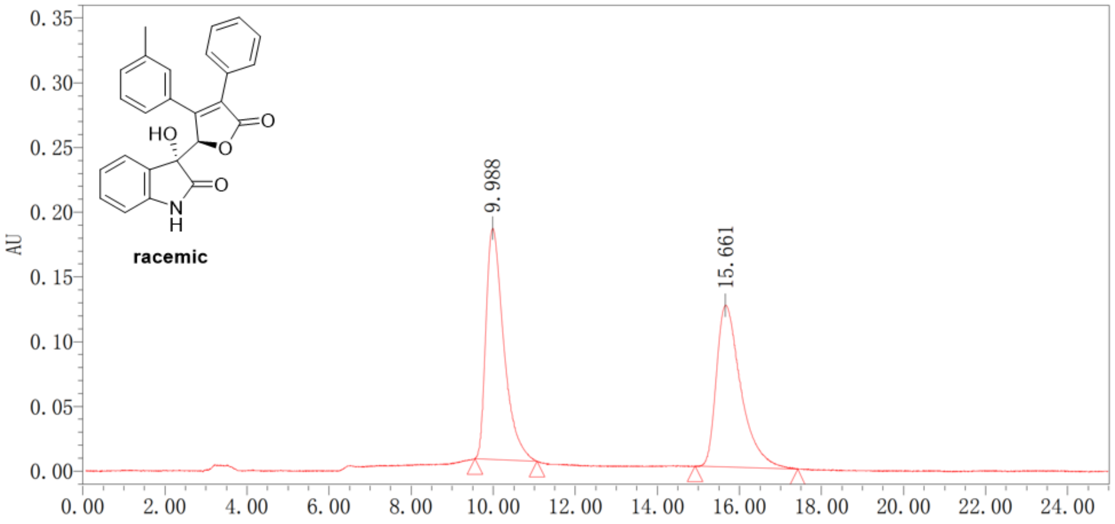

	Name	Ret.time (min)	Area (μV*s)	Height (μV)	%Area
**1**		9.988	5341136	178585	50.77
**2**		15.661	5178529	125223	49.23

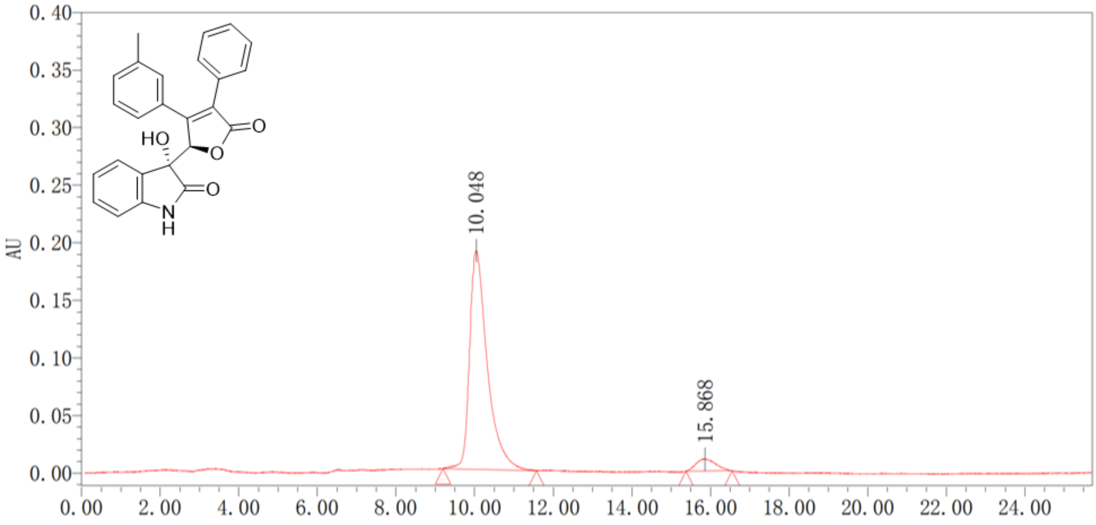

	Name	Ret.time (min)	Area (μV*s)	Height (μV)	%Area
**1**		10.048	5963088	189922	94.29
**2**		15.868	360785	10445	5.71

HPLC spectrum for **3ap:**

Condition:hexane:2-propanol=80:20
Flow rate=1.0 mL/min, λ=254 nm, Chiral IA

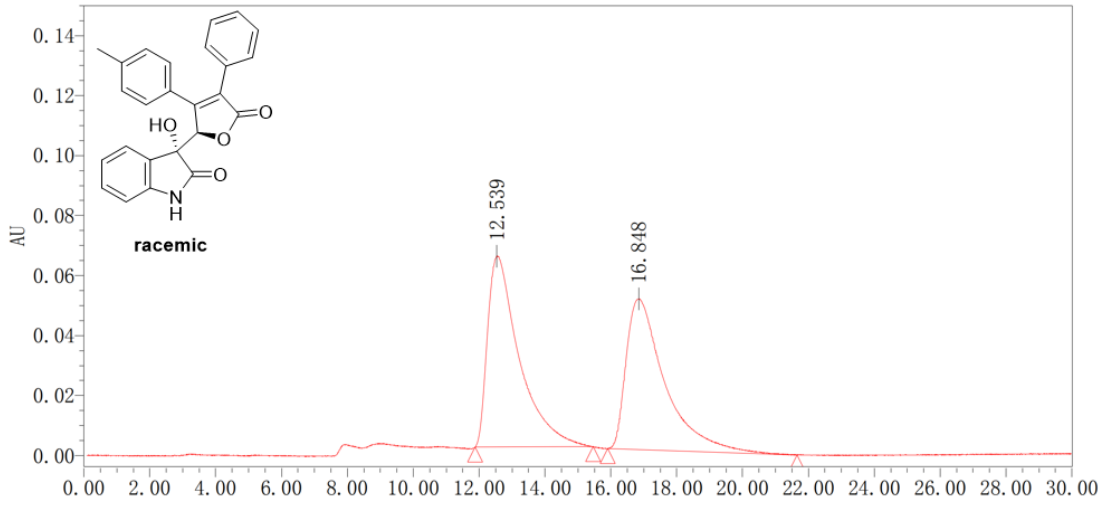

	Name	Ret.time (min)	Area (μV*s)	Height (μV)	%Area
1		12.539	4268803	63621	50.98
2		16.848	4104600	50297	49.02

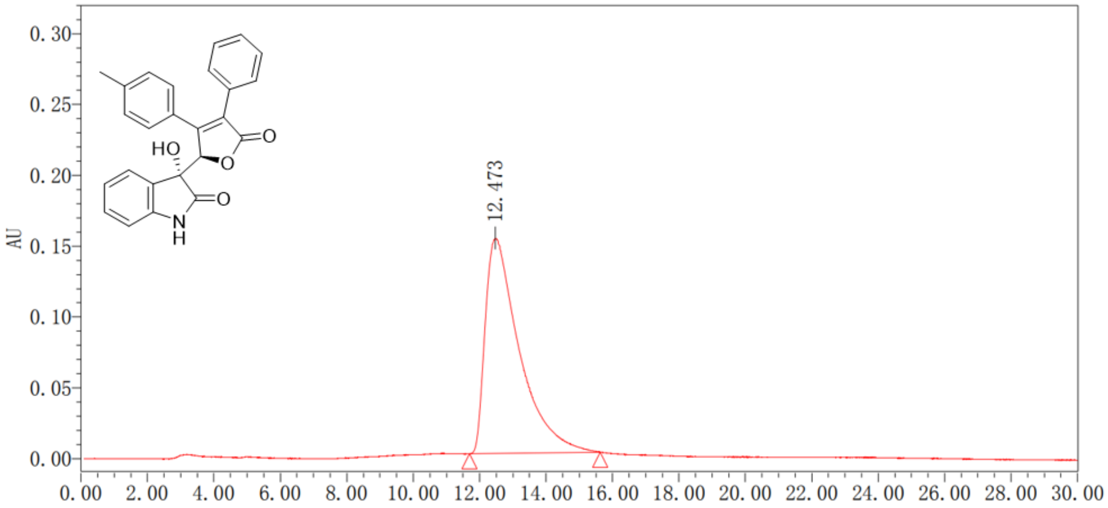

	Name	Ret.time (min)	Area (μV*s)	Height (μV)	%Area
1		12.473	10862416	151987	100.00
2					

HPLC spectrum for **3aq:**

Condition:hexane:2-propanol=80:20
Flow rate=1.0 mL/min, λ=254 nm, Chiral IA

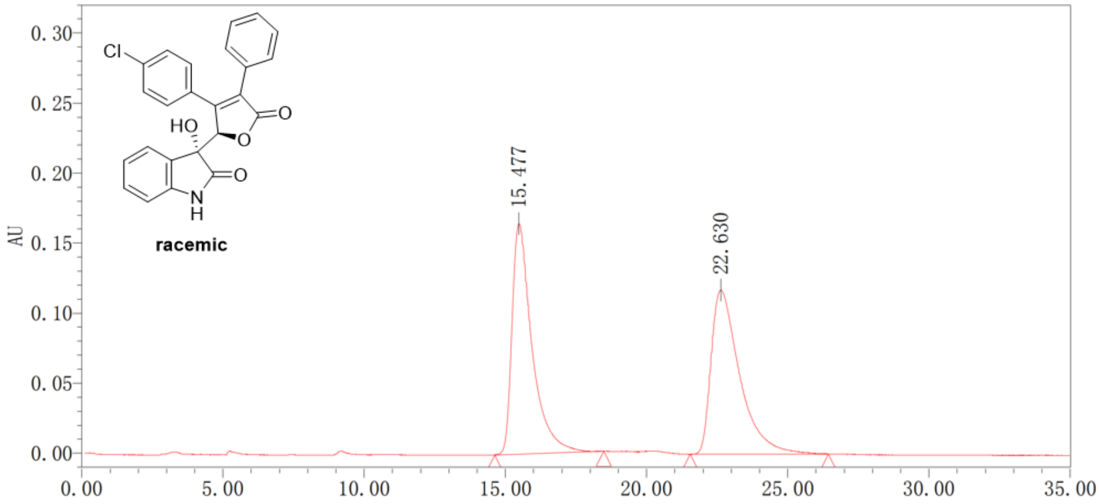

	Name	Ret.time (min)	Area (μV*s)	Height (μV)	%Area
**1**		15.477	8104577	164618	49.55
**2**		22.630	8250691	117332	50.45

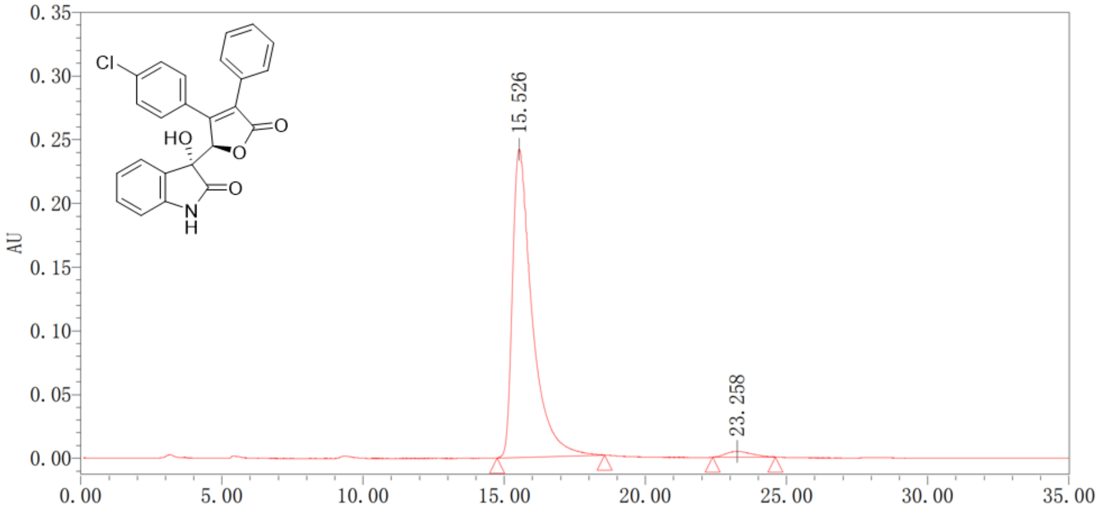

	Name	Ret.time (min)	Area (μV*s)	Height (μV)	%Area
**1**		15.526	11767787	241884	97.62
**2**		23.258	287341	4555	2.38

HPLC spectrum for **3ar:**

Condition:hexane:2-propanol=80:20
Flow rate=1.0 mL/min, λ=254 nm, Chiral IA

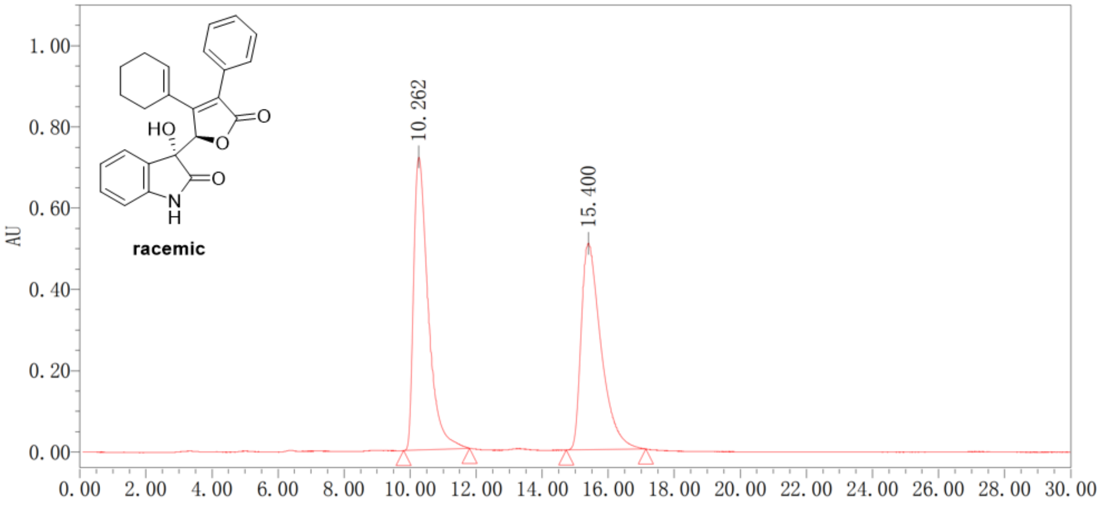

	Name	Ret.time (min)	Area (μV*s)	Height (μV)	%Area
1		10.262	21981804	721302	50.71
2		15.400	21370371	508305	49.29

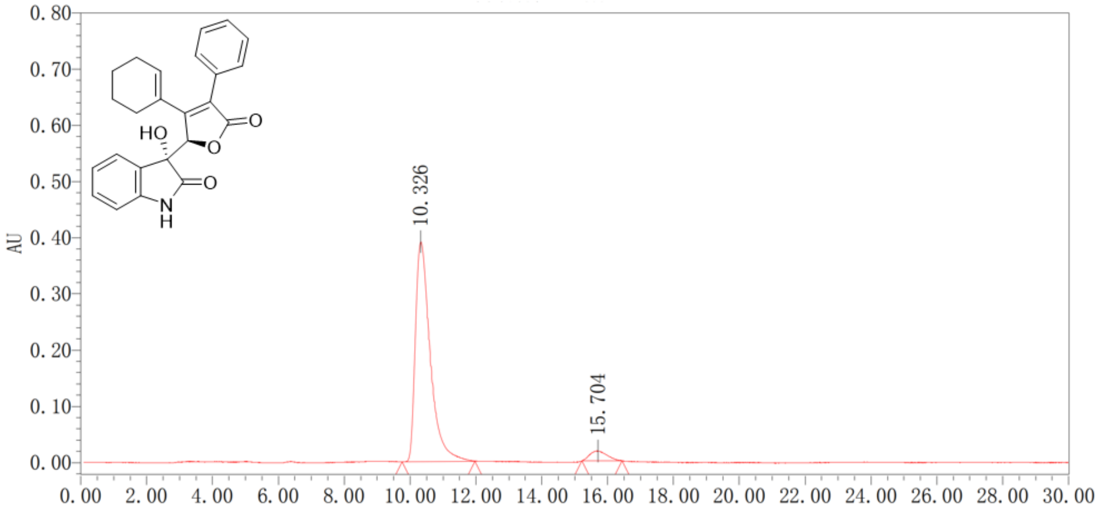

	Name	Ret.time (min)	Area (μV*s)	Height (μV)	%Area
1		10.326	12164369	391102	94.97
2		15.704	644670	17711	5.03

HPLC spectrum for **3as:**

Condition:hexane:2-propanol=80:20
Flow rate=1.0 mL/min, λ=254 nm, Chiral IG

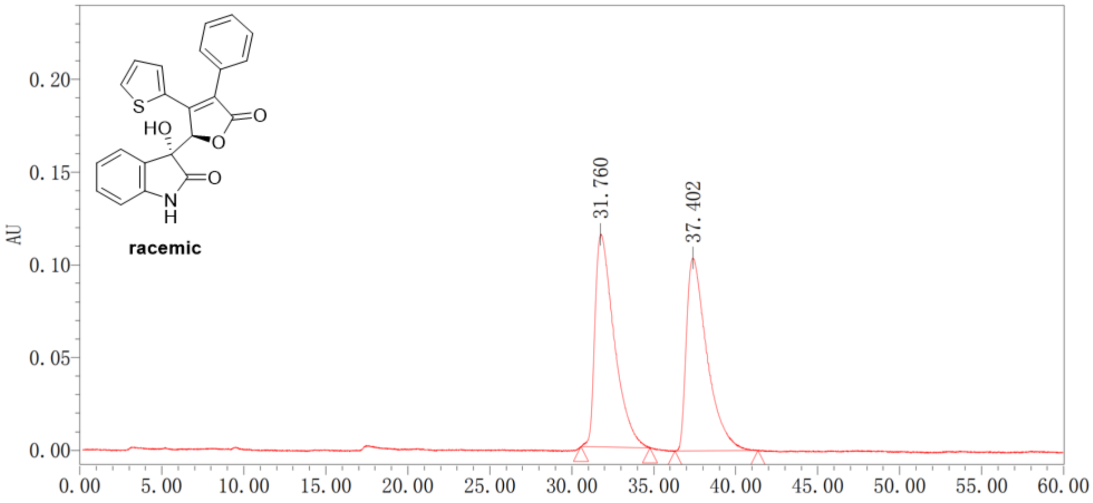

	Name	Ret.time (min)	Area (μV*s)	Height (μV)	%Area
1		31.760	9351773	114782	50.75
2		37.402	9075343	104027	49.25

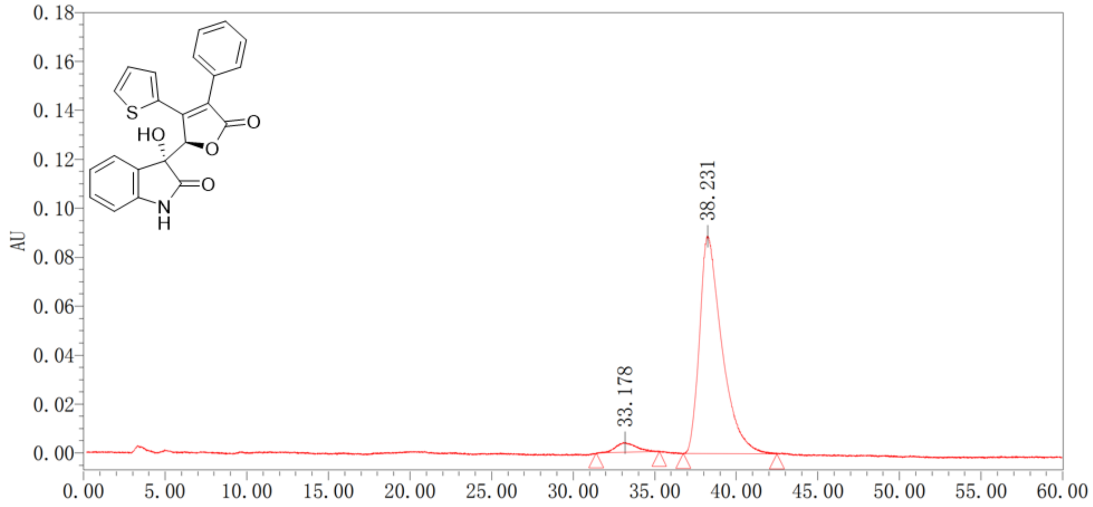

	Name	Ret.time (min)	Area (μV*s)	Height (μV)	%Area
1		33.178	347238	4026	3.93
2		38.231	8499074	88798	96.07

HPLC spectrum for **3at:**

Condition:hexane:2-propanol=80:20
Flow rate=1.0 mL/min, λ=254 nm, Chiral IA

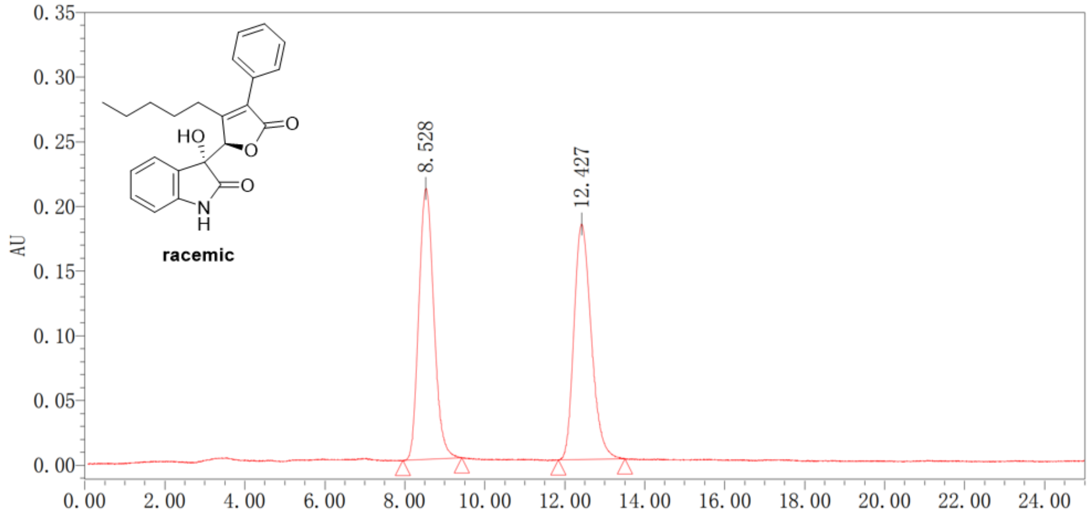

	Name	Ret.time (min)	Area (μV*s)	Height (μV)	%Area
**1**		8.528	5406918	209428	50.00
**2**		12.427	5406262	181765	50.00

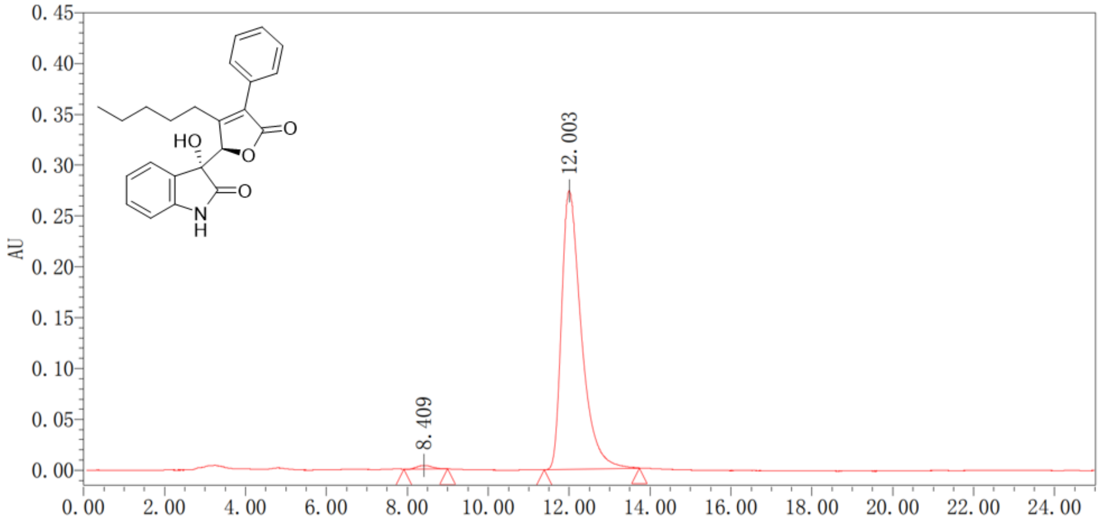

	Name	Ret.time (min)	Area (μV*s)	Height (μV)	%Area
**1**		8.409	95719	3637	1.01
**2**		12.003	9345855	273922	98.99

HPLC spectrum for **3av:**

Condition:hexane:2-propanol=80:20
Flow rate=1.0 mL/min, λ=254 nm, Chiral IA

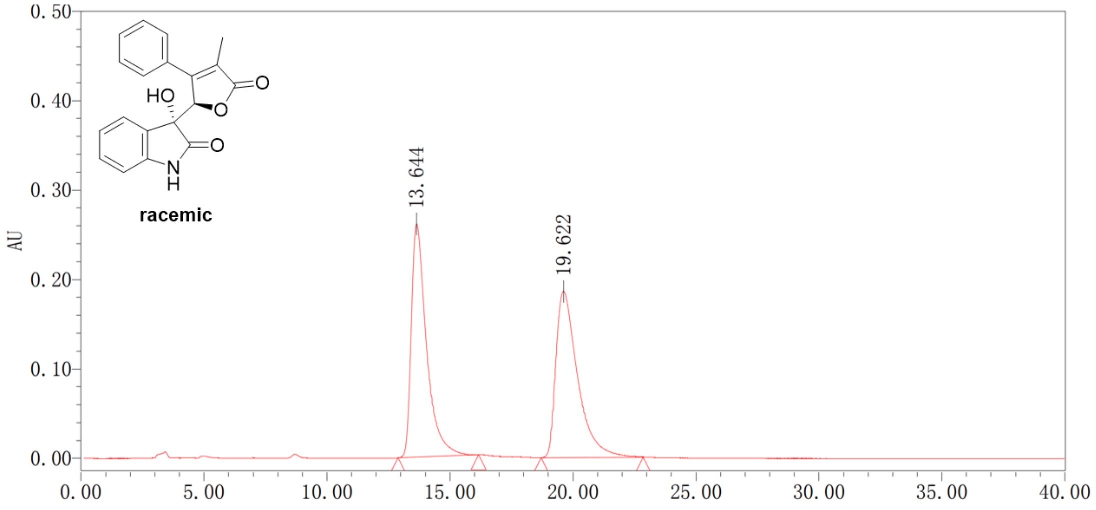

	Name	Ret.time (min)	Area (μV*s)	Height (μV)	%Area
1		13.644	11147310	260794	49.60
2		19.622	11325615	185801	50.40

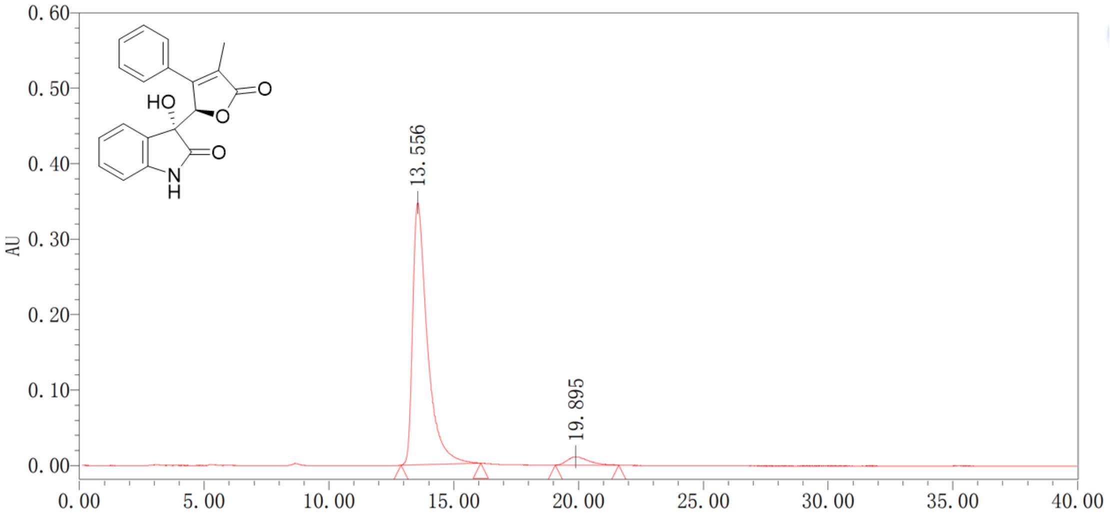

	Name	Ret.time (min)	Area (μV*s)	Height (μV)	%Area
1		13.556	14063091	347256	95.61
2		19.895	645492	10984	4.39

HPLC spectrum for **3k** with different ee of **L10:**

Condition:hexane:2-propanol=70:30
Flow rate=1.0 mL/min, λ=254 nm, Chiral ODH

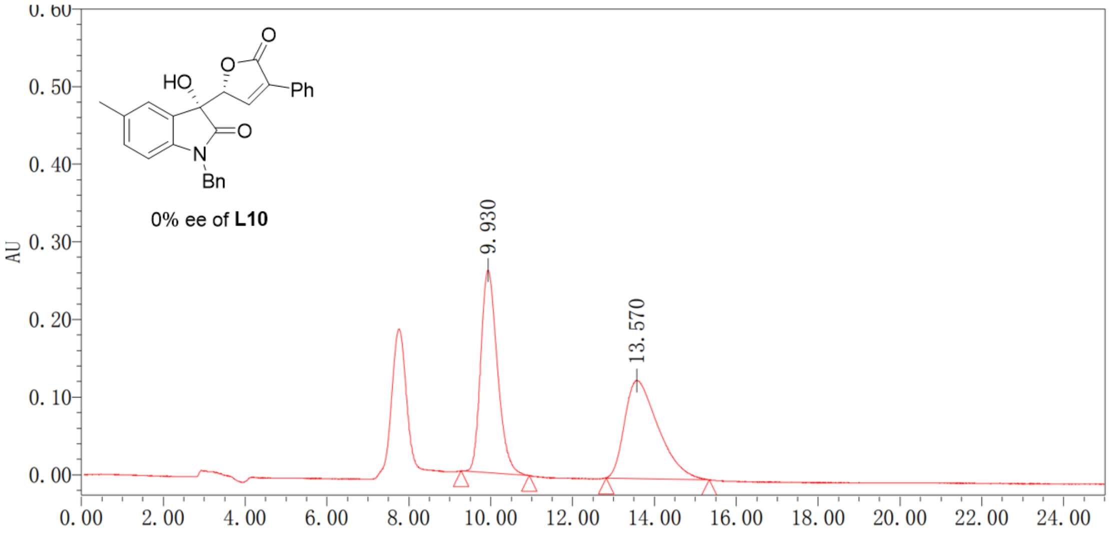

	Name	Ret.time (min)	Area (μV*s)	Height (μV)	%Area
**1**		9.930	7336974	260892	50.47
**2**		13.570	7199766	126428	49.53

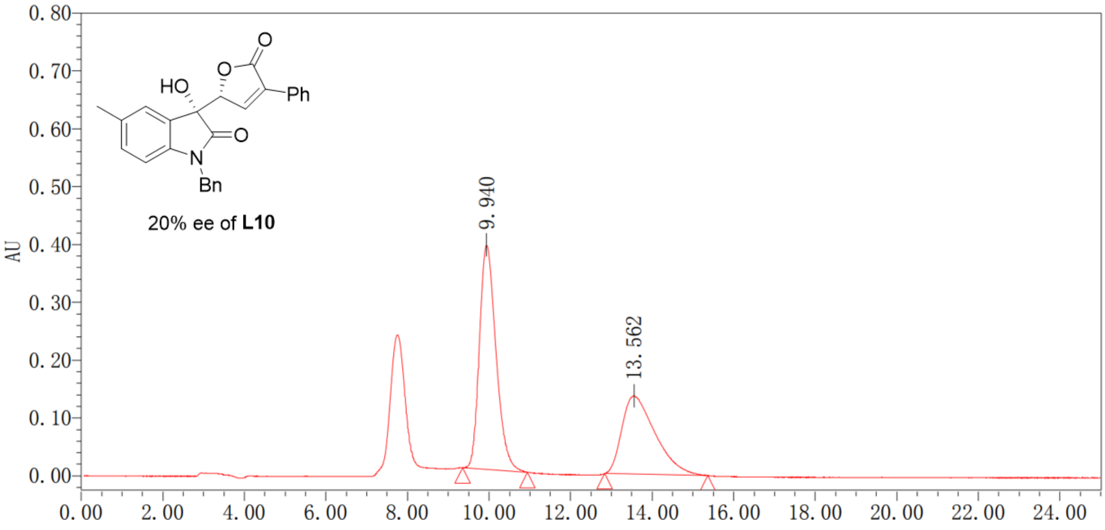

	Name	Ret.time (min)	Area (μV*s)	Height (μV)	%Area
**1**		9.940	10852533	387579	58.55
**2**		13.562	7683834	135493	41.45

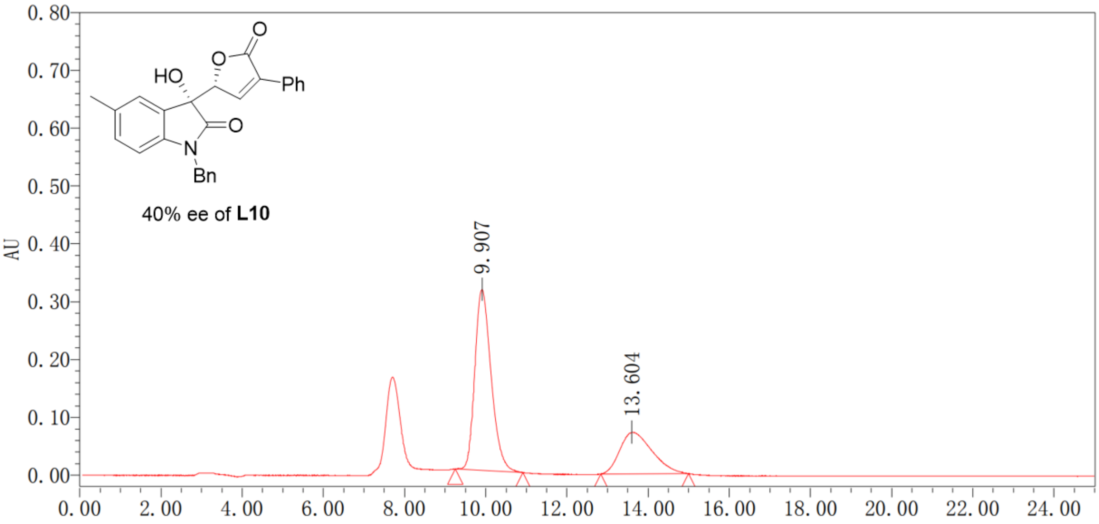

	Name	Ret.time (min)	Area (μV*s)	Height (μV)	%Area
1		9.907	8681337	312943	68.63
2		13.604	3967796	72056	31.37

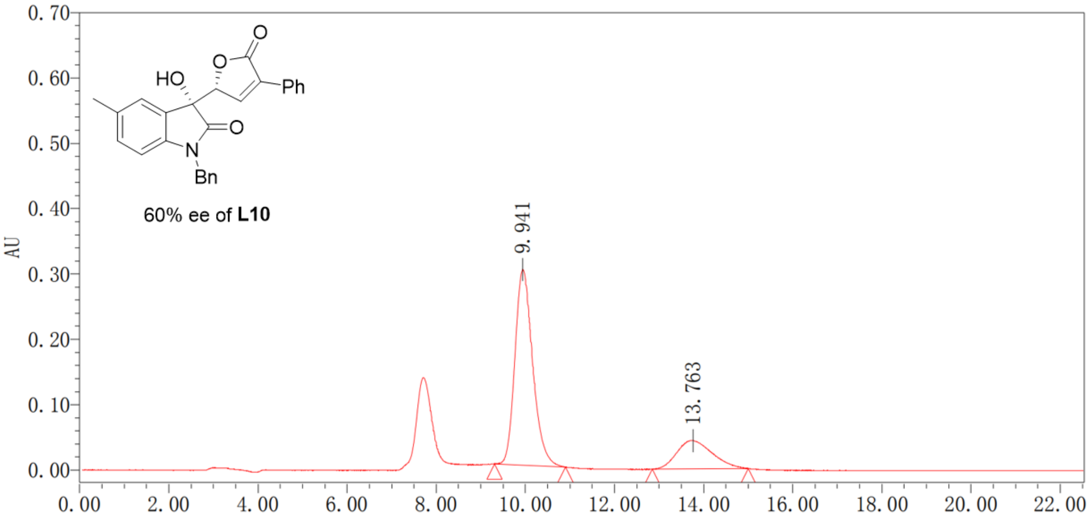

	Name	Ret.time (min)	Area (μV*s)	Height (μV)	%Area
1		9.941	8278891	299202	77.47
2		13.763	2407977	43558	22.53

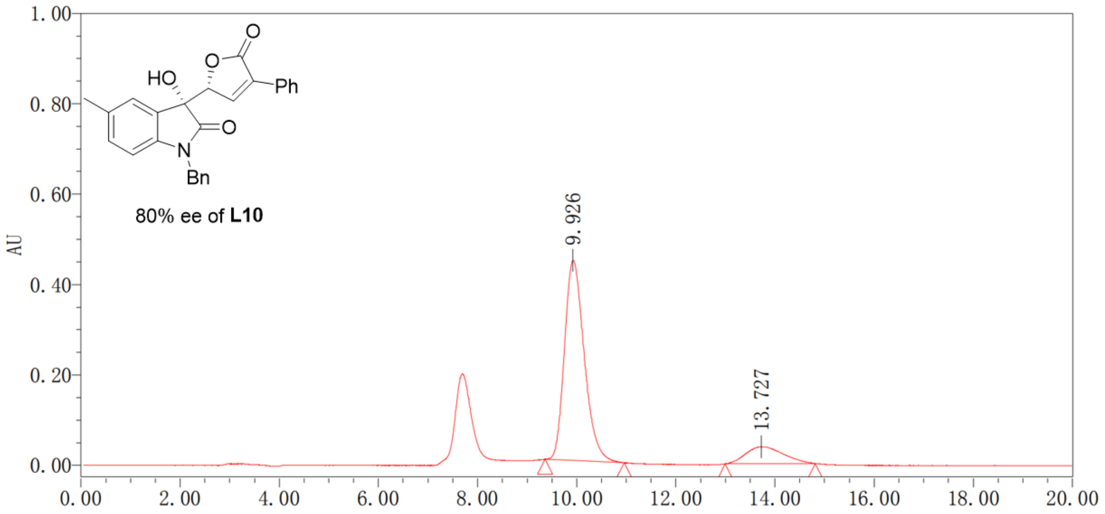

	Name	Ret.time (min)	Area (μV*s)	Height (μV)	%Area
**1**		9.926	12207077	442699	86.15
**2**		13.727	1962736	37670	13.85

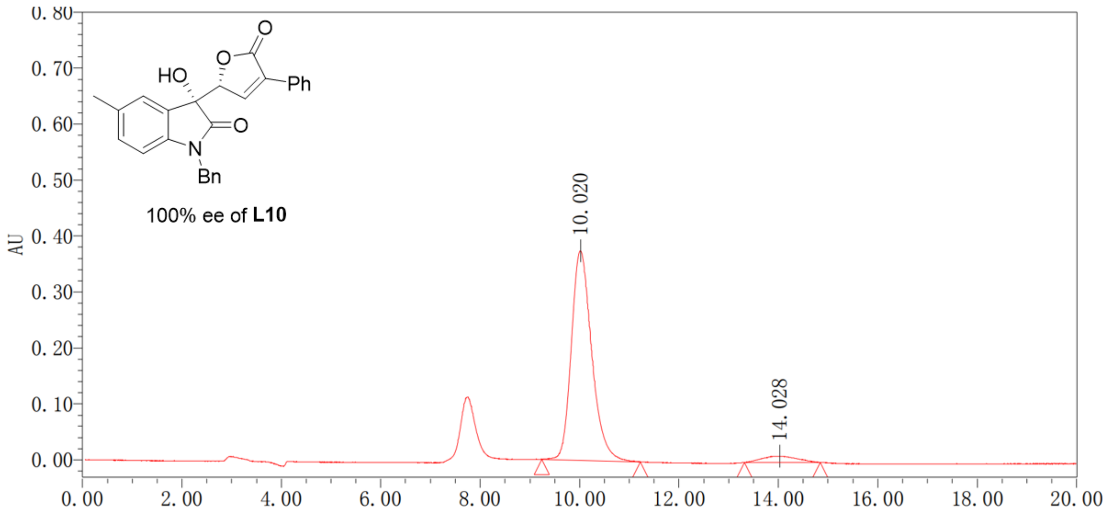

	Name	Ret.time (min)	Area (μV*s)	Height (μV)	%Area
**1**		10.020	10637589	373868	95.26
**2**		14.028	529239	11111	4.74

HPLC spectrum for **7a:**

Condition:hexane:2-propanol=80:20
Flow rate=1.0 mL/min, λ=254 nm, Chiral ODH

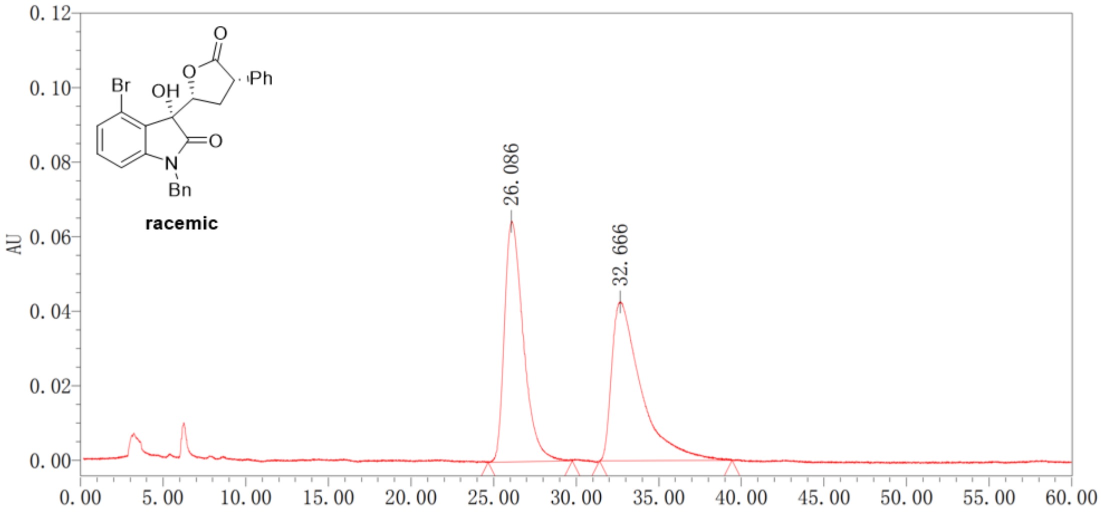

	Name	Ret.time (min)	Area (μV*s)	Height (μV)	%Area
1		26.086	5116528	64579	49.23
2		32.666	5276637	42577	50.77

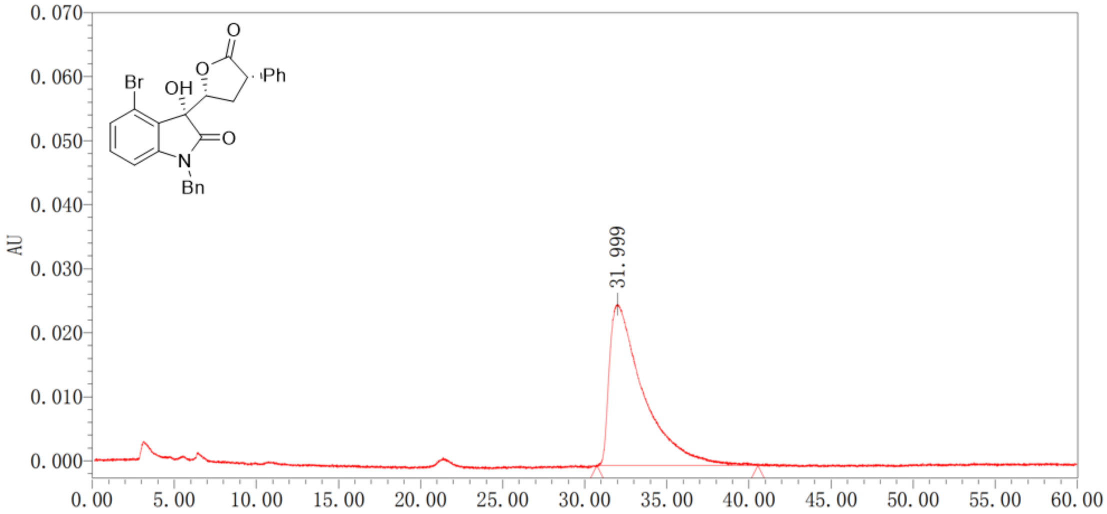

	Name	Ret.time (min)	Area (μV*s)	Height (μV)	%Area
1		31.999	3609982	25175	100.00
2					

HPLC spectrum for **7b:**

Condition:hexane:2-propanol=75:25
Flow rate=1.0 mL/min, λ=254 nm, Chiral ODH

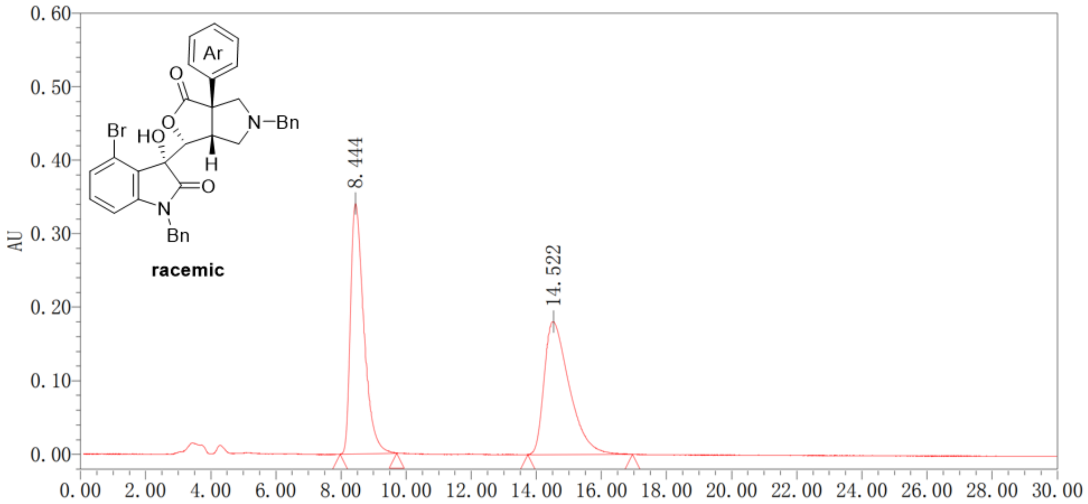

	Name	Ret.time (min)	Area (μV*s)	Height (μV)	%Area
1		8.444	9681662	340683	49.93
2		14.522	9709632	180734	50.07

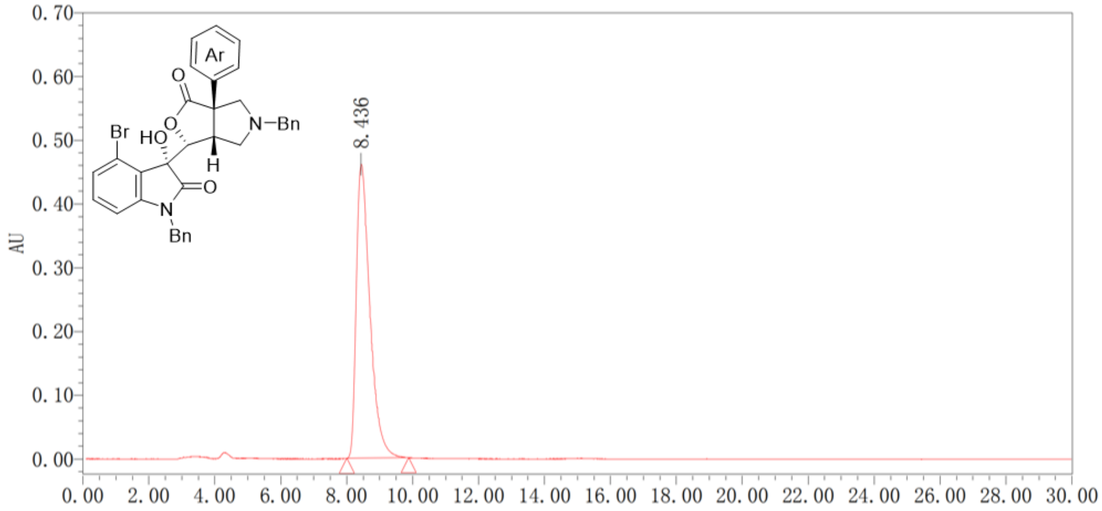

	Name	Ret.time (min)	Area (μV*s)	Height (μV)	%Area
1		8.436	13180978	461049	100
2					

10. Computational study

Method

DFT-calculations were performed with Gaussian 09 program[5], and the geometry optimization and frequency of structures with m062x[6]/6-31G(d)/Lanl2dz (6-31G(d) is applied to nonmetal elements such as C, H, O, N and Lanl2dz is used for rhodium element).[2] Intrinsic reaction coordinate calculations was conducted to ensure TS is connect with starting materials and products. Solvent effect with self-consistent reaction field (SCRF) method is based on SMD model in ethylethanoate (ε=8.86). The 3D optimized structures were displayed by CYLview visualization program.

Table S11. The energetic parameters of the intermediates and transition states of the MMCR.

Structures	H	G	E	H (corrections)	G (corrections)
**$Rh_2(OAc)_4$**	-1132.27	-1132.339919	-	0.235779	0.235779
**PDA**	-607.268	-607.317144	-	0.171916	0.122354
**INT1**	-1630.07	-1630.171111	-1630.471697	0.39803	0.300586
**INT2**	-2115.88	-2116.001031	-1831.390496	0.544845	0.426489
**INT3**	-1485	-1485.091314	-1485.797317	0.408191	0.31689
**TS1**	-1484.94	-1485.032152	-1485.741655	0.406488	0.316229
**INT4**	-1560.75	-1560.846272	-1561.606962	0.424974	0.333504
**INT5**	-1076.52	-1076.591472	-1077.127062	0.296509	0.220124
**INT6**	-1076.49	-1076.568211	-1077.104287	0.297627	0.220782
**TS2**	-1076.48	-1076.556712	-1077.093352	0.295244	0.222719
**INT7a**	-1076.56	-1076.634885	-1077.175188	0.299397	0.226904
**INT7b**	-1076.56	-1076.633513	-1077.170437	0.299291	0.226494
**TS3**	-3475.05	-3475.23589	-3477.843441	1.355725	1.169702
**INT8**	-3475.45	-3475.631559	-3477.872084	1.360099	1.182632

Cartesian coordinates.

$Rh_2(OAc)_4$

0 1

Rh	0.00096400	-0.00391800	1.18972800
O	-1.53851100	1.38997200	1.12039300
C	-1.87142800	1.87957200	0.00081300
O	-1.38104900	1.54561200	-1.11839400
O	1.38872400	-1.54653000	-1.11900800
C	1.87585000	-1.88381400	0.00062300
O	1.53953100	-1.39761400	1.12068900
O	1.55016200	1.38345400	-1.11540700
C	1.88428800	1.87097700	0.00503600
O	1.39511700	1.53490200	1.12383400
O	-1.53999600	-1.38922700	-1.12277300
C	-1.87647700	-1.87892500	-0.00429200
O	-1.38957300	-1.54511100	1.11647100
Rh	0.00385700	-0.00099800	-1.18780400
C	2.96583200	-2.92094600	-0.00619400
C	-2.96659800	-2.91600000	-0.00096500
C	2.92151100	2.96092800	0.00114100
C	-2.96120200	2.91702500	-0.00627900
H	2.98675800	-3.45982600	0.94158800
H	3.92582000	-2.41083600	-0.13885600
H	2.82585900	-3.61175600	-0.83868300
H	-2.98914000	-3.44998300	-0.95148100
H	-2.82519300	-3.61111400	0.82769600
H	-3.92644200	-2.40678000	0.13607600
H	2.41160200	3.92116100	-0.13049600
H	3.45903100	2.98036500	0.94974800
H	3.61346300	2.82240300	-0.83063100
H	-3.92056500	2.40801800	-0.14748600

H	-2.98761900	3.45061300	0.94435300
H	-2.81640800	3.61237700	-0.83416600

PDA

0 1

C	-2.42615500	-1.62583600	-0.41282500
C	-1.11606600	-1.15932900	-0.46177800
C	-0.81659500	0.14315700	-0.04052000
C	-1.86034100	0.96447100	0.40990400
C	-3.16847000	0.49513100	0.44153400
C	-3.45847600	-0.80521000	0.03543400
H	-2.63971300	-2.63993000	-0.73779600
H	-0.32691700	-1.80442100	-0.82768400
H	-1.64953000	1.97675900	0.74595000
H	-3.96091900	1.14832300	0.79461400
H	-4.47869200	-1.17472200	0.06744900
C	1.85811200	-0.00773800	0.00536800
O	2.92474000	0.56898700	-0.05266100
O	1.72783600	-1.32749500	0.17926700
C	2.96130300	-2.04557800	0.29578300
H	2.68098400	-3.08882500	0.43542100
H	3.55719900	-1.92877000	-0.61204900
H	3.53121900	-1.68692500	1.15538500
C	0.56104500	0.67161900	-0.09262500
N	0.70999800	1.97682900	-0.18305300
N	0.84137600	3.09209400	-0.26205800

INT1

0 1

Rh	2.40480700	-0.47761500	0.23671300

O	2.01649000	-2.32226900	-0.70832900
C	0.85584400	-2.58542500	-1.10804200
O	-0.16363400	-1.82244800	-1.01875100
O	0.36083900	1.87456500	0.77256200
C	1.53190400	2.16699500	1.18534700
O	2.54117700	1.42185300	1.14236500
O	-0.35797200	-0.84384200	1.73451800
C	0.61040900	-1.32490800	2.41297200
O	1.82047700	-1.32338400	2.08079300
O	0.54046700	0.84169100	-1.93633000
C	1.76116300	0.85408200	-2.29692300
O	2.73838400	0.41716000	-1.63593800
Rh	0.01112300	0.03604500	-0.10997200
C	1.68945000	3.55566300	1.75380300
C	2.03577200	1.42050100	-3.66831500
C	0.23166400	-1.93957600	3.73778700
C	0.61620100	-3.91354100	-1.78297300
H	2.62599500	3.64044100	2.30525000
H	0.84257000	3.79339100	2.40140100
H	1.69557700	4.27330100	0.92653800
H	1.33772800	2.22775200	-3.89470800
H	3.06583500	1.77265000	-3.73416800
H	1.88969400	0.62456300	-4.40632900
H	-0.54733500	-2.69102800	3.58453000
H	1.10032400	-2.39366100	4.21420800
H	-0.18126100	-1.16281800	4.38812600
H	0.49493600	-3.74471700	-2.85772700
H	1.45729900	-4.58663200	-1.61694200
H	-0.30806000	-4.35983600	-1.40886100

C	-4.06929600	-2.44934400	0.20920000
C	-2.93800600	-1.66028100	0.10085500
C	-3.06035700	-0.28570900	-0.23791600
C	-4.35670000	0.25780200	-0.46738200
C	-5.47508400	-0.54489900	-0.37664500
C	-5.32827500	-1.89507300	-0.03474400
H	-3.98136500	-3.49580500	0.48049400
H	-1.95211700	-2.06657800	0.28816000
H	-4.46454700	1.30605800	-0.73147300
H	-6.46155900	-0.13368600	-0.56067400
H	-6.21204200	-2.52161200	0.04410500
C	-2.10332900	2.00311800	-0.47724600
O	-1.97716600	2.57128400	-1.53404700
O	-2.38914200	2.57600700	0.68779500
C	-2.30582700	4.00738300	0.69821300
H	-2.51613600	4.30659600	1.72367300
H	-1.30085000	4.31988300	0.40577000
H	-3.04185200	4.43862900	0.01650200
C	-1.91204100	0.53301800	-0.30604700

INT2

0 1

Rh	-0.78252900	0.04855600	0.27366700
O	-0.65106500	1.92545300	-0.61095800
C	-1.72166300	2.53914200	-0.90252300
O	-2.89124800	2.06732100	-0.80169400
O	-3.23776200	-1.82854300	0.59984600
C	-2.20688600	-2.33253400	1.13084500
O	-1.07167700	-1.77075200	1.21001100
O	-2.77607300	-0.61698300	-2.03269800

C	-1.60732400	-1.00463300	-2.31525300
O	-0.59790100	-0.90344400	-1.55315600
O	-3.36984100	0.84484100	1.81202000
C	-2.34133000	1.13656200	2.48622100
O	-1.14257600	0.98448000	2.10007600
Rh	-3.13797500	0.12601500	-0.12343000
C	-2.31987100	-3.72842100	1.68417300
C	-2.55305000	1.74863700	3.84516600
C	-1.36799500	-1.61682500	-3.66951900
C	-1.58436800	3.94205300	-1.43372900
H	-1.59307600	-3.88394000	2.48251800
H	-2.10556000	-4.43627600	0.87572800
H	-3.33274000	-3.91283100	2.04500100
H	-3.48695900	1.39055700	4.28038900
H	-1.71095100	1.52397700	4.50073300
H	-2.62129800	2.83521000	3.72526100
H	-0.90949300	-0.86135900	-4.31621500
H	-0.66973200	-2.45254400	-3.58173400
H	-2.30674000	-1.94653500	-4.11524000
H	-0.87731500	4.50394800	-0.81933900
H	-1.18888200	3.89445900	-2.45347500
H	-2.55273100	4.44236000	-1.44964900
C	6.38830100	0.86042200	0.03491700
C	5.03380000	0.94461200	-0.27490500
C	4.06471200	0.51767400	0.64034800
C	4.48322400	0.00083200	1.87053600
C	5.83776700	-0.08236100	2.18118700
C	6.79564800	0.34670000	1.26472000
H	7.12606400	1.19593800	-0.68800500

H	4.72970800	1.33559100	-1.24351200
H	3.74422500	-0.34655700	2.58826300
H	6.14579100	-0.48204900	3.14291100
H	7.85205600	0.28184200	1.50690100
C	2.25712300	1.54192300	-0.80577000
O	1.95135400	1.23956400	-1.93481600
O	2.37693000	2.80532300	-0.38321200
C	2.11718400	3.79689200	-1.37736500
H	2.12349600	4.75425800	-0.85676700
H	2.89945700	3.77970100	-2.14225500
H	1.14920100	3.61510500	-1.84610500
C	2.60683800	0.55138500	0.27945100
Si	2.08163100	-2.38614100	-0.65566100
C	0.72307200	-3.65408200	-0.40967700
H	-0.22022200	-3.32177400	-0.85088000
H	1.02858200	-4.58719200	-0.89989700
H	0.55740500	-3.86340400	0.65113500
C	2.34262000	-1.95102800	-2.45825800
H	2.34146800	-2.85900800	-3.07357900
H	1.55943300	-1.27519200	-2.80748600
H	3.30864300	-1.45253800	-2.59519800
C	3.69109900	-3.04564600	0.07096000
H	3.60488300	-3.24722300	1.14420500
H	3.96186700	-3.98509200	-0.42706600
H	4.51609700	-2.33994200	-0.07753000
C	1.58357000	0.09104000	1.24750600
C	1.83257200	-0.77306700	0.28686700
H	1.15575900	0.29702800	2.22074900

INT3

1 1

C	-4.96720000	0.34087000	0.08152400
C	-3.62608800	0.56012800	0.38107100
C	-2.65943900	0.53219700	-0.63129500
C	-3.06404500	0.26925400	-1.94167200
C	-4.40651800	0.05188700	-2.24281000
C	-5.36242100	0.08725700	-1.23134500
H	-5.70586000	0.36497800	0.87728200
H	-3.33043800	0.75362000	1.40816300
H	-2.32168800	0.22812400	-2.73516500
H	-4.70246100	-0.14767300	-3.26831400
H	-6.40905600	-0.08590000	-1.46208600
C	-0.73118000	1.69578300	0.66373600
O	0.41121300	1.72739800	1.11084900
O	-1.63465000	2.60255200	0.97296000
C	-1.21069400	3.64783400	1.86456400
H	-2.07997000	4.29059600	1.99055800
H	-0.90316600	3.22310700	2.82183400
H	-0.38224700	4.20323700	1.42121300
C	-1.19780000	0.65624200	-0.31109300
Si	-0.47493500	-2.33686700	0.50649600
C	-2.08232900	-3.09602700	-0.07751800
H	-2.09606400	-3.20498600	-1.16702200
H	-2.21682200	-4.09061700	0.36344800
H	-2.93930500	-2.47862300	0.21389800
C	1.02494300	-3.32543900	-0.03196500
H	1.96175300	-2.78954700	0.16006500
H	1.07101400	-4.28327600	0.49841500
H	0.98082400	-3.53883800	-1.10564200

C	-0.50862400	-1.97824300	2.34494800
H	-1.43038400	-1.44538900	2.60582100
H	-0.48680800	-2.90636800	2.92667400
H	0.32930600	-1.35307500	2.67403100
C	-0.16265800	0.21010800	-1.29751500
C	-0.37856600	-0.65450100	-0.33884600
H	0.25431600	0.45280600	-2.26709200
Cu	1.68494200	0.22522000	0.22632300
O	2.87117200	-0.76172100	1.66157800
O	3.24503900	1.07012900	-0.92201400
H	3.68605300	-1.02794200	1.20063600
C	4.36210900	0.61883100	-1.08608100
O	4.66667300	-0.55777200	-0.51658300
C	5.43565900	1.28623400	-1.88440600
H	5.76825700	0.62336300	-2.68968800
H	5.05511800	2.21544700	-2.30486800
H	6.29437800	1.49783200	-1.23861400
H	5.58481100	-0.82265800	-0.71667800
H	2.53773100	-1.55265000	2.11672100

TS1

1 1

C	-4.05613500	-0.55226700	-1.23075900
C	-2.74441000	-0.90657500	-0.92627700
C	-2.32668500	-1.06175100	0.40329500
C	-3.27570600	-0.86909500	1.41339600
C	-4.58946300	-0.51564400	1.11490800
C	-4.98593300	-0.35043000	-0.21040700
H	-4.35466200	-0.43658100	-2.26917400
H	-2.03664400	-1.07314000	-1.73332000

H	-2.97271900	-0.98603300	2.45135500
H	-5.30584000	-0.37321200	1.91902700
H	-6.00973600	-0.07681800	-0.44761600
C	-0.12850300	-2.30439100	-0.09107100
O	1.08820200	-2.30418200	-0.18221500
O	-0.89036600	-3.17713800	-0.76580100
C	-0.18746500	-4.09200900	-1.60726700
H	-0.95363900	-4.68757300	-2.10291400
H	0.41162800	-3.55554600	-2.34729000
H	0.46586300	-4.73577400	-1.01360700
C	-0.89843700	-1.34948200	0.75981600
Si	-0.72680300	2.46571800	-0.26307800
C	-2.29528900	2.29486400	0.69574500
H	-2.12316300	1.67436500	1.57911100
H	-2.63280700	3.28119200	1.03820300
H	-3.09010400	1.83522200	0.09943200
C	0.66650300	3.31604400	0.63342300
H	1.58982000	3.31539900	0.04325200
H	0.40769200	4.35731800	0.86215800
H	0.86709400	2.81260100	1.58380600
C	-0.28534400	1.33680600	-1.67470100
H	-0.13707000	0.31227000	-1.32466900
H	-1.08005300	1.32696000	-2.42950600
H	0.64541600	1.67322000	-2.14941200
C	-0.40166100	-1.13844200	2.14570900
C	0.02772100	-0.20528400	1.34541900
H	-0.40607800	-1.62837900	3.11012500
Cu	1.77018600	0.31347700	0.53541000
O	-1.31558600	3.88193600	-1.40118100

O	3.52471600	0.56992700	-0.37506600
H	-1.57440900	4.73178500	-0.98411100
C	4.52770400	-0.07353800	-0.09386000
O	4.49414200	-0.86970000	0.96389500
C	5.79184500	-0.01214600	-0.89111100
H	5.97813700	-0.99282900	-1.34211500
H	5.69910800	0.73759100	-1.67501000
H	6.63738000	0.23073500	-0.24035800
H	5.34086400	-1.33922700	1.08516000
H	-0.76756900	4.07887400	-2.19092100

INT4

1 2

C	4.78163400	1.66207900	-0.23875200
C	4.11052400	0.45320200	-0.07717900
C	2.90970900	0.20291100	-0.75519900
C	2.40372400	1.20177100	-1.59899300
C	3.07515900	2.41212400	-1.75887900
C	4.26735400	2.64875100	-1.07853500
H	5.71249100	1.83254800	0.29410500
H	4.52795500	-0.29966500	0.58649400
H	1.48291800	1.02256700	-2.14800400
H	2.66657200	3.16785100	-2.42355100
H	4.79300200	3.59012900	-1.20459600
C	2.43367700	-1.81940100	0.69379500
O	2.32679300	-1.29824200	1.80043500
O	2.73009100	-3.09735100	0.53857400
C	2.88482900	-3.86043900	1.74486600
H	3.12513900	-4.87263400	1.42437300
H	3.69458100	-3.44858600	2.35001500

H	1.95588000	-3.85174600	2.31889400
C	2.14672600	-1.07649200	-0.57310700
C	1.41936300	-1.75703300	-1.68042100
C	0.63993200	-1.06652600	-0.90122700
H	1.59636700	-2.34001400	-2.57301300
Cu	-0.70115900	0.10134800	-0.00684300
O	-2.01931500	1.32623600	0.93395800
C	-1.81315600	2.52763700	1.07598500
O	-0.77340200	3.05450500	0.44753900
C	-2.67491000	3.41885200	1.90455100
H	-3.11203800	4.19673000	1.26989600
H	-3.46477700	2.83708700	2.37612900
H	-2.06472100	3.90545100	2.67242700
H	-0.69291700	4.01313700	0.61913300
O	0.67573300	0.80571100	1.34348900
H	1.32558600	0.08948100	1.58914400
H	1.21162500	1.48762000	0.89645400
Si	-3.73684100	-0.99236000	-0.55251100
C	-4.92747600	0.43600100	-0.47684400
H	-5.06276600	0.88590800	-1.46622400
H	-5.90829400	0.09631000	-0.12504000
H	-4.57265100	1.21060000	0.21021700
C	-3.28969400	-1.68523800	1.12075900
H	-4.15226300	-2.18641600	1.57526300
H	-2.48923900	-2.43031000	1.03183400
H	-2.96406400	-0.90334800	1.81544300
C	-4.27251700	-2.31803400	-1.74846500
H	-4.42414600	-1.91433500	-2.75581700
H	-3.53924600	-3.12983600	-1.80945500

H	-5.22282600	-2.75625800	-1.42266800
O	-2.24376000	-0.37194200	-1.20367900
H	-1.91119700	-0.88067300	-1.96451700

INT5

1 1

C	4.76960200	0.62415200	-1.28685400
C	3.44161000	0.21930700	-1.19663300
C	2.76946600	0.26828400	0.02894300
C	3.44831200	0.73597000	1.15530800
C	4.77949000	1.13702300	1.06657600
C	5.44350100	1.08187100	-0.15562100
H	5.27957200	0.58197300	-2.24450200
H	2.92684000	-0.13953800	-2.08209600
H	2.93661700	0.79113800	2.11319300
H	5.29514200	1.49243400	1.95359300
H	6.48025100	1.39618700	-0.22767400
C	0.92979500	-1.38270500	-0.58468100
O	1.69496700	-2.15738600	-1.09648900
O	-0.40779700	-1.59578600	-0.57503200
C	-0.85313400	-2.81667300	-1.18947100
H	-1.93486000	-2.83039300	-1.06194400
H	-0.59052000	-2.81709200	-2.24830100
H	-0.39408500	-3.67272800	-0.69225400
C	1.31621900	-0.11197700	0.12865600
C	0.30935700	1.00482800	0.29143100
C	0.53846000	0.29130400	1.36392400
H	-0.01860700	1.92200700	-0.17941300
Cu	-1.58894900	-0.17114400	0.72478700
O	-3.36894700	0.20735800	-0.27760600

C	-3.65995100	1.31222700	-0.71029500
O	-2.78463000	2.29966000	-0.54818400
C	-4.94362400	1.62347300	-1.41110900
H	-4.73297500	2.00814700	-2.41442800
H	-5.55320500	0.72427000	-1.48121000
H	-5.48561200	2.39697000	-0.85745500
H	-3.11338800	3.13509400	-0.93099000
O	-2.58540300	-1.31068300	2.18594100
H	-2.18423100	-2.14103600	2.49215500
H	-3.48247500	-1.54153000	1.89058400
H	0.53090700	0.18944800	2.44186200

INT6

1 1

C	-1.07008700	-1.75112500	-0.73042200
C	0.28717600	-2.05920000	-0.57397700
C	-1.61055600	-0.61826800	-0.13969800
H	-1.73759400	-2.40078000	-1.29222600
H	0.50881500	-3.02058200	-1.05372800
C	-2.98335700	-0.17452400	-0.23326700
C	-3.42952800	0.90019400	0.56586000
C	-3.88841300	-0.77980600	-1.13181100
C	-4.73759900	1.34767200	0.47568200
H	-2.75853300	1.37449900	1.27607900
C	-5.19118700	-0.32068700	-1.22287100
H	-3.56642400	-1.59392800	-1.77110900
C	-5.61818100	0.73940300	-0.41887300
H	-5.07198100	2.16919800	1.09974700
H	-5.87926200	-0.78336700	-1.92219500
H	-6.64172700	1.09353700	-0.49293200

C	-0.70434400	0.21799800	0.73488000
O	-0.57488900	0.01061300	1.92138600
O	-0.14156700	1.19934500	0.05689400
C	0.69008900	2.09999300	0.81525400
H	0.94906400	2.90227900	0.12833100
H	0.13147400	2.48932000	1.66898500
H	1.58812100	1.58232000	1.16157700
Cu	1.84087100	-1.10572300	0.25227800
O	3.68841000	-0.23917200	-0.21550400
C	3.79245900	0.69801000	-0.98741000
O	2.68402300	1.19554900	-1.53519100
C	5.09633400	1.33055200	-1.36381300
H	5.07820500	2.39688700	-1.11710300
H	5.90985000	0.84238700	-0.82996000
H	5.25370600	1.23354800	-2.44297200
H	2.88878900	1.93466700	-2.13767800
O	2.13455400	-0.47570200	2.29007000
H	2.60434200	-1.09090200	2.87689600
H	1.23680900	-0.36944600	2.65379000

TS2

1 1

C	-0.82108300	-1.85238900	-0.56424500
C	0.49801500	-2.34703200	-0.39388500
C	-1.37440800	-0.79355100	0.09567500
H	-1.44043900	-2.40593000	-1.27545200
H	0.51399100	-3.44185600	-0.32383400
C	-2.71063800	-0.25221800	-0.18798100
C	-3.51170000	0.26702900	0.83940200
C	-3.21241500	-0.27521600	-1.49833300

C	-4.78641700	0.74657900	0.56102800
H	-3.14215200	0.28250200	1.85993800
C	-4.48348500	0.21115100	-1.77368200
H	-2.59364400	-0.65739500	-2.30583100
C	-5.27458300	0.72084200	-0.74394000
H	-5.40123900	1.13774100	1.36536100
H	-4.85660000	0.19682900	-2.79271100
H	-6.26942100	1.09832200	-0.95940300
C	-0.51984300	-0.24334000	1.18233800
O	0.33966100	-0.92200900	1.72864600
O	-0.72230400	1.03699200	1.44518100
C	0.12869900	1.60990300	2.45014300
H	-0.16530600	2.65592700	2.51794400
H	-0.02708900	1.10716100	3.40721600
H	1.17571600	1.52379900	2.15033900
Cu	2.12517300	-1.19290300	-0.35601600
O	3.23074200	0.31003300	-1.30762500
C	2.81603100	1.43241800	-1.07874700
O	1.66683200	1.55321900	-0.40466800
C	3.50274900	2.69079700	-1.50506700
H	3.75694000	3.28723100	-0.62289100
H	4.40872100	2.44541100	-2.05666600
H	2.83514500	3.28436700	-2.13748600
H	1.40881600	2.48782100	-0.29424800
O	3.05586100	-0.50784900	1.48334500
H	3.79245700	-1.02401600	1.84991900
H	2.30221300	-0.63665900	2.08700700

INT7a

1 1

C	0.88192800	-0.12801400	-1.90397200
C	2.22074400	-0.36128900	-1.73912000
C	0.18577400	-0.88493600	-0.88303400
H	0.44108000	0.44010300	-2.71329200
H	3.09736600	-0.11114800	-2.31754900
C	-1.26138700	-0.92526700	-0.63736600
C	-1.81328100	-1.67687400	0.41373100
C	-2.13233600	-0.19765400	-1.46251700
C	-3.19071700	-1.71019400	0.61366700
H	-1.16414100	-2.25347500	1.06398700
C	-3.50910000	-0.23959100	-1.26246400
H	-1.73498500	0.39759100	-2.27946600
C	-4.04750600	-0.99794800	-0.22522100
H	-3.59667300	-2.30809100	1.42456300
H	-4.16143400	0.32538300	-1.92169200
H	-5.12139500	-1.03378900	-0.07122500
C	1.19604100	-1.51482000	-0.19195400
O	2.40734400	-1.23874000	-0.70621100
O	1.13654900	-2.29465000	0.87444100
C	2.38226400	-2.78222400	1.39554000
H	2.11626800	-3.38245700	2.26367700
H	2.89113800	-3.40202900	0.65336100
H	3.01996300	-1.94624900	1.69120000
Cu	1.48576100	1.53152200	-0.44970800
O	-0.35466000	2.39295900	0.14012900
C	-1.01875700	1.79871600	0.97160400
O	-0.39657900	0.91731900	1.76872200
C	-2.47792400	2.01503300	1.20664700
H	-2.60709000	2.52786000	2.16637400

H	-2.89421900	2.62528800	0.40680500
H	-3.00115000	1.05436200	1.25640000
H	-1.03657900	0.42348900	2.31615600
O	2.38344400	0.91104900	1.36830100
H	1.65216800	0.92853400	2.00947300
H	3.10937800	1.42735100	1.75647900

INT7b

1 1

C	3.04794300	1.99468200	0.27297700
C	4.04384800	1.25804600	0.81925800
C	2.00233200	1.06844300	-0.08181500
H	3.04963000	3.06228200	0.10496200
H	5.02543800	1.49466100	1.19997500
C	0.67222100	1.30869700	-0.66083700
C	0.16152800	0.43527700	-1.64232300
C	-0.13221700	2.36019100	-0.20897100
C	-1.14929200	0.58114700	-2.10849800
H	0.81372900	-0.31721300	-2.08536400
C	-1.42629600	2.51802800	-0.69801300
H	0.25546000	3.04121900	0.54313700
C	-1.94665300	1.62035400	-1.63153500
H	-1.52885900	-0.10257900	-2.86214600
H	-2.03655500	3.34233900	-0.34040700
H	-2.95961800	1.74202400	-2.00311000
C	2.47181400	-0.14687600	0.31427600
O	3.69493400	-0.06160900	0.86141500
O	1.85942700	-1.35176600	0.34100700
C	2.51880100	-2.38254600	-0.43207200
H	1.89909600	-3.27410200	-0.33816000

H	2.58981900	-2.06991200	-1.47770200
H	3.51115700	-2.57193600	-0.01945400
Cu	-0.31264300	-1.14809000	0.02642200
O	-1.52052200	-0.08357800	1.41005100
C	-2.70236400	0.09501900	1.18867200
O	-3.32904800	-0.74437200	0.34775800
C	-3.53120500	1.17114400	1.81491200
H	-4.25662700	0.71489100	2.49769600
H	-2.88684000	1.85149800	2.36953200
H	-4.08382900	1.72039500	1.04641900
H	-4.26812200	-0.50335400	0.23701800
O	-1.51023300	-2.76931800	-0.57161000
H	-2.40250900	-2.44571500	-0.35288400
H	-1.39394100	-3.59436700	-0.07090200

TS3

3 1

O	1.20917200	2.35997700	-1.58959000
O	0.11293800	1.20399800	0.82281300
O	2.49535100	3.38191200	0.00091100
O	-0.58674600	-0.17346900	-1.43764500
N	-1.92518300	2.33058300	0.52915800
C	-2.59646800	1.32624300	-1.45583500
C	1.42184800	3.37477300	-0.73090500
C	-0.91918800	1.50364900	0.21072900
C	-0.05652000	2.55882300	-2.15387200
C	-3.46155600	1.06692400	-2.51431300
C	-0.51061800	3.79472300	-1.71605900
C	-2.94951400	2.25297300	-0.45179900
C	0.21969000	5.49772900	0.08078800

C	-1.26142100	0.85357000	-1.11560300
C	1.32475000	6.19097400	0.58649600
C	-1.25754700	7.08738400	1.15496600
C	-1.07250200	5.95308900	0.37500200
C	0.38066400	4.30812400	-0.76450300
C	-4.66865800	1.76442100	-2.56370500
C	-0.15243800	7.77401900	1.65604800
C	-4.98937600	2.69769400	-1.57666600
C	1.13433400	7.32370400	1.37072600
C	-4.13350100	2.95731600	-0.49546100
H	-1.91392000	2.97484700	1.31228200
H	-0.20063200	2.05495000	-3.09936400
H	-3.19870900	0.34688000	-3.28377000
H	2.33075800	5.86186700	0.34799100
H	-2.26051800	7.44149500	1.36914800
H	-1.93750100	5.42121600	-0.01905600
H	-5.36306400	1.58488700	-3.37721700
H	-0.29537400	8.66305900	2.26126200
H	-5.92842200	3.23899000	-1.64135800
H	1.99493600	7.86496100	1.74927300
H	-4.40126200	3.67691800	0.27242200
C	3.30993300	2.20271600	0.05377100
H	2.72416000	1.36717900	0.45165300
H	3.71244500	1.97204100	-0.93879600
H	4.12785400	2.44419700	0.72754100
O	0.01303000	-2.89164000	-0.16464300
O	2.10996500	-1.02833100	-0.19191300
N	4.12837400	-2.03604200	-0.27167400
H	4.63924400	-2.88827600	-0.48889900

C	1.53530700	-3.97893100	1.32840100
H	1.96451500	-3.02364400	1.63234800
H	2.32299900	-4.73849500	1.28248000
C	0.48924800	-4.40821400	2.35466300
H	1.04338700	-4.59573400	3.28015100
C	2.81865300	-2.05810200	-0.41156600
O	0.52068800	-1.45316400	2.37862700
O	-1.82923400	-0.91602900	0.94176400
N	-3.72356600	-1.48235400	2.00624200
H	-4.15916200	-1.71859900	2.89245300
C	-0.70544900	-3.49441600	2.61943600
H	-1.25528700	-3.33336300	1.69171100
H	-1.36383400	-3.97313000	3.35150100
H	0.06629800	-5.38283500	2.08963300
C	-2.41784200	-1.25401300	1.98661500
C	-4.58884900	-1.29679700	0.86491900
C	-5.49064400	-0.22483100	0.91073200
C	-4.52253700	-2.18864700	-0.21071600
C	-6.36467400	-0.07354300	-0.16233600
C	-5.40841600	-1.97690500	-1.26798000
C	-6.34094200	-0.93985700	-1.25735300
H	-7.08910100	0.73528600	-0.15246200
H	-5.38356800	-2.65882700	-2.11507000
C	4.91664500	-0.89395100	0.14448500
C	4.89209200	-0.50896700	1.49443500
C	5.76569400	-0.30444000	-0.80040800
C	5.75116200	0.52102500	1.87416200
C	6.59746400	0.72721100	-0.36497200
C	6.61413800	1.14589600	0.96656200

H	5.77498900	0.83760300	2.91246100
H	7.27045200	1.19475300	-1.07968500
C	2.20390400	-3.34297200	-0.95552800
C	0.51898200	-5.11282200	-0.64452800
C	1.73270800	-3.12909700	-2.39703700
H	2.94463000	-4.15021300	-0.91655600
C	0.04988900	-4.95615900	-2.08116900
H	1.32577300	-5.84773200	-0.54747500
H	-0.31072600	-5.39076000	0.00535900
C	1.16472400	-4.42396700	-2.97741400
H	2.58211800	-2.76836800	-2.98907800
H	0.96265100	-2.34989300	-2.38411500
H	-0.29003500	-5.93625800	-2.42743600
H	-0.81009800	-4.28061500	-2.09584600
H	0.78375600	-4.23692400	-3.98494600
H	1.96546200	-5.16807700	-3.07247800
N	1.03719900	-3.79789400	-0.09076600
C	-1.68468900	-1.34876400	3.31931100
C	0.25602000	-2.23764400	4.54789000
C	-1.41291800	0.05467000	3.86450900
H	-2.28442000	-1.92162400	4.03663200
C	0.53611300	-0.86390400	5.13585700
H	-0.42440400	-2.82542300	5.17377300
H	1.18219000	-2.79151700	4.39186700
C	-0.73631200	-0.02716300	5.23208900
H	-2.37429000	0.57667600	3.93063100
H	-0.78020200	0.58501200	3.14433800
H	0.98132500	-1.01123700	6.12425000
H	1.27680500	-0.36040600	4.50773200

H	-0.50238200	0.97776000	5.59191100
H	-1.42697400	-0.47247300	5.95925000
N	-0.37801400	-2.12380500	3.17394700
Mg	0.13588500	-1.01091800	0.48696600
Cu	1.78729900	0.23471900	-1.86564700
C	0.64466100	-0.62380100	-6.04695400
H	-0.17152300	-0.08321300	-6.53461900
H	0.52780600	-1.68004100	-6.30569400
H	1.60440900	-0.24954700	-6.39761600
C	0.53418600	-0.45969000	-4.56633700
O	-0.59450700	-0.91003900	-4.05515100
H	-0.63278500	-0.74131700	-3.07838200
O	1.41746000	0.05270500	-3.87473000
H	-1.43849100	4.25408800	-2.03546900
C	7.56652700	2.21618100	1.42778200
H	7.14479500	2.80082200	2.24914300
H	8.49484900	1.76259200	1.79144900
H	7.83019700	2.89564600	0.61422600
C	3.99190200	-1.21451100	2.48881200
H	2.94139900	-0.99273400	2.24993400
H	4.11568500	-2.29841400	2.35302300
C	4.24585900	-0.87381100	3.95517200
H	3.60281200	-1.48345300	4.59614300
H	5.28175000	-1.07462300	4.24166600
H	4.02699500	0.17598900	4.17087200
C	5.78728900	-0.74339400	-2.24662400
H	6.68566700	-0.34011000	-2.72123200
H	5.87427000	-1.83494500	-2.31977400
C	4.55339000	-0.27963700	-3.02599600

H	3.64309100	-0.73997400	-2.61367900
H	4.44541400	0.81036900	-2.98832300
H	4.60460900	-0.57019600	-4.07872600
C	-7.31684200	-0.77038600	-2.39127700
H	-7.66837100	0.26230400	-2.46525000
H	-6.87336100	-1.05922800	-3.34828500
H	-8.19757500	-1.40295800	-2.23671900
C	-5.48301700	0.72847700	2.08954400
H	-5.58284300	0.15577100	3.02213600
H	-4.49025200	1.20428600	2.14288600
C	-6.56578500	1.80248300	2.07008800
H	-6.48847900	2.43558000	2.95672700
H	-6.48106400	2.44462300	1.18770700
H	-7.56410100	1.35655500	2.06593200
C	-3.56754600	-3.36126800	-0.24445400
H	-2.53549400	-3.01259200	-0.10833700
H	-3.60675700	-3.79527100	-1.24921100
C	-3.90679200	-4.45952800	0.77135700
H	-4.91469600	-4.84730200	0.60391500
H	-3.20967900	-5.29938000	0.68084000
H	-3.86131700	-4.09401300	1.80265500

INT8

3 1

O	1.63017500	2.20176200	-1.08986900
O	0.27151600	1.16270400	1.23895200
O	3.04855800	3.19307400	0.32518800
O	-0.21947100	0.15295800	-1.26238800
N	-1.65661500	2.42585500	0.89599700
C	-2.07563600	1.87864700	-1.30607000

C	2.01076900	3.23993200	-0.40146000
C	-0.60777900	1.66763600	0.53842100
C	0.30526800	2.47681200	-1.63763100
C	-2.80130000	1.86646700	-2.48470400
C	0.09259400	3.90365700	-1.28314900
C	-2.57522700	2.54311200	-0.17859100
C	1.28902100	5.67909800	0.11487500
C	-0.66419600	1.42745800	-0.98512700
C	2.56164900	6.23060600	0.29997300
C	0.29153400	7.63858200	1.11481000
C	0.15398500	6.38693700	0.52721100
C	1.11438600	4.37216800	-0.53183100
C	-4.04294500	2.51198600	-2.50723100
C	1.56047100	8.18747400	1.29764000
C	-4.51981700	3.17581300	-1.37847100
C	2.69171800	7.48313600	0.89104500
C	-3.78670900	3.20749800	-0.18682700
H	-1.85341200	2.70111000	1.85407600
H	0.35901500	2.29234700	-2.71528900
H	-2.42288500	1.36008700	-3.36839100
H	3.44626100	5.69580600	-0.03158200
H	-0.59039500	8.18462800	1.43381600
H	-0.83193600	5.94705700	0.40072600
H	-4.63671300	2.50183800	-3.41514900
H	1.66756000	9.16436100	1.75838500
H	-5.47944900	3.68223500	-1.42053700
H	3.67892700	7.91234200	1.02757000
H	-4.15423000	3.72783100	0.69246800
C	3.74079500	1.91978400	0.52982600

H	3.00615400	1.15838500	0.80318100
H	4.28759600	1.66478700	-0.38058400
H	4.42335400	2.12404500	1.34939800
O	-0.48458000	-2.84631600	-0.28899300
O	1.91741100	-1.42195400	-0.06933600
N	3.74260700	-2.72491700	-0.29570000
H	4.11902700	-3.61248400	-0.62514300
C	0.78443800	-4.21612900	1.21662700
H	1.34166300	-3.34626600	1.56095100
H	1.45125700	-5.08167700	1.15679400
C	-0.34195800	-4.51407800	2.19740700
H	0.15097300	-4.78145100	3.13760900
C	2.44033500	-2.52974700	-0.39591300
O	0.10491100	-1.59529500	2.33618600
O	-2.06864400	-0.65914200	0.79270500
N	-4.06757200	-1.09012000	1.74441700
H	-4.54986000	-1.31367500	2.61099500
C	-1.40918400	-3.45321500	2.43799600
H	-1.88826300	-3.19045500	1.49500600
H	-2.15717800	-3.84758900	3.13159400
H	-0.88372000	-5.41670800	1.89874700
C	-2.74217800	-0.98268400	1.78797800
C	-4.87651100	-0.79346400	0.59300600
C	-5.70944800	0.33125000	0.65731300
C	-4.84045000	-1.63958400	-0.52326600
C	-6.52429000	0.60129400	-0.44256600
C	-5.66072100	-1.31394200	-1.60277400
C	-6.50671800	-0.20443500	-1.58115400
H	-7.18450300	1.46338700	-0.42434900

H	-5.64558500	-1.95493300	-2.48203300
C	4.67907700	-1.73053200	0.15578500
C	4.61881600	-1.27398400	1.48523500
C	5.66899500	-1.30622700	-0.74041100
C	5.57107300	-0.33539600	1.87893700
C	6.59299700	-0.35947500	-0.29173500
C	6.55943400	0.13721700	1.00886400
H	5.56103800	0.03313600	2.90080300
H	7.36595100	-0.01899600	-0.97700700
C	1.62647000	-3.65791500	-1.01373900
C	-0.34926000	-5.10640700	-0.80815400
C	1.25767400	-3.31779900	-2.46001300
H	2.21144300	-4.58277000	-0.98231600
C	-0.73036800	-4.83108700	-2.25214600
H	0.31818800	-5.96875100	-0.70863300
H	-1.23679800	-5.25298400	-0.19155900
C	0.49094400	-4.47777600	-3.09490300
H	2.18225600	-3.11312400	-3.01031700
H	0.64365100	-2.40961600	-2.45687600
H	-1.22254700	-5.72799800	-2.63959600
H	-1.45602000	-4.01324300	-2.27447700
H	0.18934800	-4.20419200	-4.10985800
H	1.15684000	-5.34658000	-3.17251200
N	0.36767400	-3.91759800	-0.20550400
C	-2.11267200	-1.21490100	3.15558900
C	-0.40561800	-2.40890400	4.45239100
C	-1.68410700	0.11613100	3.76810000
H	-2.83243300	-1.71825100	3.80934300
C	0.02714900	-1.11268600	5.11535800

H	-1.20807700	-2.91008000	5.00336400
H	0.43682100	-3.08989500	4.32973100
C	-1.11640600	-0.10273100	5.16938300
H	-2.56824400	0.76373400	3.79692900
H	-0.93556700	0.57250800	3.11324100
H	0.37897900	-1.35931100	6.12178400
H	0.87165600	-0.69754700	4.55727100
H	-0.76380400	0.84750100	5.57961300
H	-1.90872000	-0.47173500	5.83278300
N	-0.92323600	-2.15923600	3.05298000
Mg	-0.09782800	-1.03935900	0.43308100
Cu	2.00062800	-0.09851400	-1.72716200
C	0.62633300	-0.16676900	-5.84501300
H	-0.22369400	0.39531200	-6.23954500
H	0.47797600	-1.21809500	-6.11367700
H	1.55824200	0.19553400	-6.27649300
C	0.66253000	-0.05295700	-4.35441600
O	-0.46809500	-0.34251700	-3.76069500
H	-0.41009600	-0.20304400	-2.74164200
O	1.68511700	0.28264300	-3.74200200
H	-0.78777000	4.45791800	-1.59097300
C	7.57171300	1.14340600	1.48724300
H	8.26069300	0.68743100	2.20657500
H	8.16375100	1.53774600	0.65767000
H	7.08488900	1.98267100	1.99523300
C	3.58594200	-1.80506200	2.45730100
H	2.60630200	-1.36175700	2.23309800
H	3.47276800	-2.88257800	2.28107700
C	3.90609100	-1.57507000	3.93169100

H	3.16499800	-2.08626300	4.55361900
H	4.89341300	-1.96701100	4.19561600
H	3.87812300	-0.51350400	4.19580100
C	5.75276900	-1.81733100	-2.15956100
H	6.74002100	-1.56026200	-2.55357400
H	5.68753700	-2.91274000	-2.18015400
C	4.67497000	-1.22575700	-3.07264300
H	3.68386400	-1.60773900	-2.80503600
H	4.66147200	-0.13148400	-3.01294100
H	4.85141300	-1.50000500	-4.11738400
C	-7.36466500	0.12112900	-2.77512300
H	-8.02911600	0.96399900	-2.56805900
H	-6.74497000	0.38166800	-3.64050500
H	-7.98042300	-0.73637000	-3.06523600
C	-5.68823100	1.21584800	1.88670700
H	-5.91012500	0.60759300	2.77427200
H	-4.66010800	1.58146300	2.02710800
C	-6.64922900	2.39846800	1.85547900
H	-7.68806200	2.06339700	1.77547900
H	-6.55606000	2.97994700	2.77703500
H	-6.44345700	3.06507600	1.01153600
C	-3.99136600	-2.88826400	-0.56933200
H	-2.94481100	-2.64695900	-0.34913600
H	-4.00561800	-3.26626200	-1.59712900
C	-4.49604100	-3.99206000	0.36733800
H	-5.51948200	-4.28139200	0.10939800
H	-3.86553500	-4.88474900	0.28815800
H	-4.49471600	-3.66945100	1.41378800

11. *In silico* screening and molecular docking

***In silico* screening:** The high-throughput in virtual screening against PTP1B was performed with autodock vina software in ubuntu environment. Firstly, the PTP1B structure was downloaded from PDB library (PDB ID: 1T4J) and receptor. Pdbqt file was prepared. The ligands were generated through rdkit or chemdraw to give .sdf files. Then the ligands were converted to .pdbqt files and energy-minimization was performed on these ligands according to the following codes:

```
obabel -isdf ligand.sdf -osdf -O *.sdf -m
obminimize
obminimize -ff MMFF94 -n 1000 *.sdf
obabel -isdf *.sdf -opdbqt -O*.pdbqt --gen3D
ls >lig.txt
perl vina_linux.pl
lig.txt
tail -n11 *.log>results.txt
```

The *in virtual* hits were selected by the ΔG< -7.0 kcal/mol and reasonable binding pose. Four types of scaffolds including 2HF, carbolines, oxindole-branch, and CHBOs were screened out from the MREAL as shown in the scheme S6.

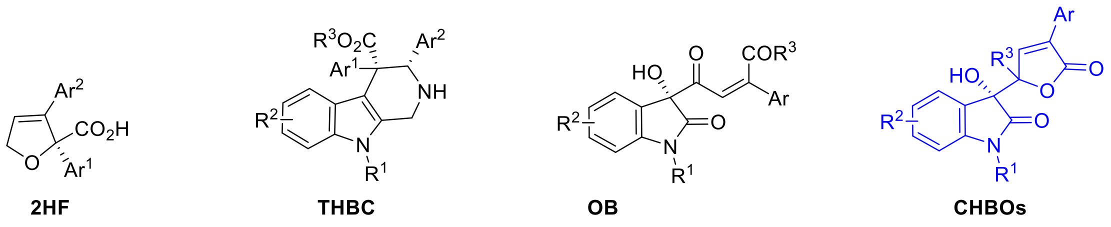

Scheme S6. Four types of scaffolds were identified as *in virtual* hits.

Molecular docking: The molecular docking of PTP1B and the enantiomers of **3ak** were conducted by AutoDock Vina software[10]. Ligands were created with ChemOffice 2014 Chem3D software. Energy-minimized conformations of **3ak** were output with .pdb format. Then the ligand torsion parameters were set in AutoDockTools to give.pdbqt format. The coordinates for PTP1B (PDB ID: 1T4J, https://www.rcsb.org/structure/1T4J) were downloaded from the Protein Data Bank. The original binding ligand was deleted from the .pdb file, and hydrogen atoms were

added. Then flexible and rigid PTP1B files were created in AutoDockTools. The docking results were analyzed and output by MacPyMOL 2.0.

12. Biological study

Protein expression and purification[7]: Human PTP1B construct was subcloned into pET28a(+) vector, which contains an N-terminal His6-tag. BL21 DE3 *E. coli* cells were transformed as follows: 2 µL of plasmid(100 ng/µL) and 200 µL of DE3 cells were incubated for 30 min on ice, a 45 second heat shock at 37 ℃, 2 min on ice, recovery with 800 µL SOC for 1 h at 37 ℃ followed by plating on a Kanamycin agar plate (50µg/mL).One colony was picked and used to inoculate 10 mL of LB media supplemented with kanamycin (50µg/mL).When the culture reached OD_{600} = 0.6, 10mL were transferred to one liter of LB media supplemented with Kanamycin (50 µg/mL) and grown at 37 ℃ to an OD_{600} = 0.6. Media is transferred to a 16 ℃ incubator. Then, the media was supplemented with 500 µM Isopropyl β-D-1-thiogalactopyranoside (IPTG) at OD_{600} = 0.8 and incubated for 16 h. Cells were centrifuged (4000 rpm, 20 min) and liquid was decanted. Cells were resuspended in lysis buffer (50 mM HEPES, pH 7.4, 150 mM KCl, 5 mM $MgCl_2$, 5% glycerol, 1 mM phenylmethane sulfonyl fluoride (PMSF), 2 mM 2-mercaptoethanol (*β*-met)). Cells were lysed in a cell crusher (pressure 1000 bar), the mixed products were collected, centrifuged (12000 rpm, 4°C, 20 min), and the supernatant was left to discard the precipitate. The nickel column was first equilibrated with equilibration solution, and then the protein supernatant was up-sampled twice to bind the protein. The proteins were then gradually eluted with imidazole (10, 20, 60, 200 mM imidazole). The target protein-containing fractions confirmed by SDS PAGE were placed in 30, 000 kDa MWCO protein ultrafiltration tubes and the imidazole were replaced with dialysis buffer (20 mM HEPES, 150 mM KCl, 1 mM $MgCl_2$). After the imidazole was removed cleanly, an equal volume of glycerol was added as a protective solution. When concentration was determined to be greater than or equal to 1 mg /mL, protein was frozen with liquid nitrogen. Protein was then stored in a -80 ℃ freezer.

***In Vitro* Phosphatase Activity Assays[8]**：Incubate 1 nM purified PTP1B protein with positive control or compound to be tested in reaction buffer (25 mM Bis-Tris propane pH 7.5,

50 mM NaCl, 2 mM EDTA, 2 mM DTT) for 10 min at 37°C in a total volume of 90 μL. Add 10 μL of substrate buffer containing DiFMUP (final concentration of 10 μM) to the reaction buffer and incubate for 10 min at 37°C. The fluorescence intensity at 455 nm was read using a multifunctional reader with 358 nm as the excitation light, and the measured fluorescence value was used to calculate the inhibition rate of the enzyme activity by the sample over the value of the blank wells. The IC_{50} values of the compounds were calculated by Graphpad Prism 7.0, using a non-linear fit of the inhibition rate to the concentration of the inhibitor. The IC_{50}s of ***Rac*-6ak (*S*, *S*)-3ak** and **(*R*, *R*)-3ak** are 1.46±0.28μM, 0.81±0.10μM and 3.31±0.20μM respectively.

Cellular anti-cancer immunity assay[9]: MB231 cells were seeded at a density of 5000 cells per well in a 96-well plate and incubated at 37 °C. For in vitro dose-escalation studies, the tumor cells were cultured in DMEM with 10% FBS and treated with either 0.5 ng/ml IFNγ or medium alone as a negative control. PTP1B inhibitors, including Osunprotafib as the positive control, were added, and cell confluence was monitored for 3 days. After the 3-day treatment period, viable cells were quantified using the CCK8 assay according to the manufacturer's protocol. The percent growth inhibition by the inhibitors at the indicated concentrations was calculated relative to the 'no compound/with IFNγ' control.

13. References

(1) Wei, C. M.; Li, C. J. Enantioselective direct-addition of terminal alkynes to imines catalyzed by copper(I)pybox complex in water and in toluene. *Journal of the American Chemical Society* **2002**, *124* (20), 5638-5639. DOI: 10.1021/ja026007t.

(2) Brunel, J. M. BINOL: A versatile chiral reagent. *Chemical Reviews* **2005**, *105* (3), 857-897, Review. DOI: 10.1021/cr040079g.

(3) Zhou, C.; Xu, J. Application of Chiral Bisamide Ligands in Asymmetric Catalytic Syntheses. *Current Organic Synthesis* **2013**, *10* (3), 394-410. DOI: 10.2174/1570179411310030005.

(4) Chen, D.-F.; Gong, L.-Z. Feng chiral <i>N</i>,<i>N</i>′-dioxide ligands: uniqueness and impacts. *Organic Chemistry Frontiers* **2023**, *10* (14), 3676-3683, Editorial Material. DOI: 10.1039/d3qo00566f.

(5) Gaussian 09, R. A., M. J. Frisch, G. W. Trucks, H. B. Schlegel, G. E. Scuseria, M. A. Robb, J. R. Cheeseman, G. Scalmani, V. Barone, G. A. Petersson, H. Nakatsuji, X. Li, M. Caricato, A. Marenich, J. Bloino, B. G. Janesko, R. Gomperts, B. Mennucci, H. P. Hratchian, J. V. Ortiz, A. F. Izmaylov, J. L. Sonnenberg, D. Williams-Young, F. Ding, F. Lipparini, F. Egidi, J. Goings, B. Peng, A. Petrone, T. Henderson, D. Ranasinghe, V. G. Zakrzewski, J. Gao, N. Rega, G. Zheng, W. Liang, M. Hada, M. Ehara, K. Toyota, R. Fukuda, J. Hasegawa, M. Ishida, T. Nakajima, Y. Honda, O. Kitao, H. Nakai, T. Vreven, K. Throssell, J. A. Montgomery, Jr., J. E. Peralta, F. Ogliaro, M. Bearpark, J. J. Heyd, E. Brothers, K. N. Kudin, V. N. Staroverov, T. Keith, R. Kobayashi, J. Normand, K. Raghavachari, A. Rendell, J. C. Burant, S. S. Iyengar, J. Tomasi, M. Cossi, J. M. Millam, M. Klene, C. Adamo, R. Cammi, J. W. Ochterski, R. L. Martin, K. Morokuma, O. Farkas, J. B. Foresman, and D. J. Fox, Gaussian, Inc., Wallingford CT, 2016.

(6) Walker, M.; Harvey, A. J. A.; Sen, A.; Dessent, C. E. H. Performance of M06, M06-2X, and M06-HF Density Functionals for Conformationally Flexible Anionic Clusters: M06 Functionals Perform Better than B3LYP for a Model System with Dispersion and Ionic Hydrogen-Bonding Interactions. *Journal of Physical Chemistry A* **2013**, *117* (47), 12590-12600. DOI: 10.1021/jp408166m.

(7) Gubiani, J. R.; Wijeratne, E. M. K.; Shi, T.; Araujo, A. R.; Arnold, A. E.; Chapman, E.; Gunatilaka, A. A. L. An epigenetic modifier induces production of (10′S)-verruculide B, an inhibitor of protein tyrosine phosphatases by <i>Phoma</i> sp nov LG0217, a fungal endophyte of <i>Parkinsonia microphylla</i>. *Bioorganic & Medicinal Chemistry* **2017**, *25* (6), 1860-1866. DOI: 10.1016/j.bmc.2017.01.048.

(8) Wiede, F.; Lu, K.-H.; Du, X.; Zeissig, M. N.; Xu, R.; Goh, P. K.; Xirouchaki, C. E.; Hogarth, S. J.; Greatorex, S.; Sek, K.; et al. PTP1B Is an Intracellular Checkpoint that Limits T- cell and CAR T- cell Antitumor Immunity. *Cancer Discovery* **2022**, *12* (3), 752-773, Article. DOI: 10.1158/2159-8290.cd-21-0694.

(9) Liang, S.; Tran, E.; Du, X.; Dong, J.; Sudholz, H.; Chen, H.; Qu, Z.; Huntington, N. D.; Babon, J. J.; Kershaw, N. J.; et al. A small molecule inhibitor of PTP1B and PTPN2 enhances T cell anti-tumor immunity. *Nature Communications* **2023**, *14* (1), Article. DOI: 10.1038/s41467-023-40170-8.

(10) Morris, G. M.; Huey, R.; Lindstrom, W.; Sanner, M. F.; Belew, R. K.; Goodsell, D. S.; Olson, A. J. AutoDock4 and AutoDockTools4: Automated Docking with Selective Receptor Flexibility. *Journal of Computational Chemistry* **2009**, *30* (16), 2785-2791. DOI: 10.1002/jcc.21256.
