# Supplementary material for: Multicatalysis-Enabled Multicomponent Reactions Generate a PTP1B Inhibitor
Source: ACS Cent Sci. 2025 May 19;11(6):938–49. doi: 10.1021/acscentsci.5c00041 (PMC12203432; doi:10.1021/acscentsci.5c00041)
Supplement: Supplementary file 2 [file oc5c00041_si_002.pdf]

oc-2025-000414.R1

Name: Peer Review Information for "Multicatalysis-Enabled Multicomponent Reactions Generate A PTP1B Inhibitor"

First Round of Reviewer Comments

Reviewer: 1

Comments to the Author

In this manuscript, the authors have developed a multicatalysis-promoted multicomponent cascade reaction (MMCR) for the synthesis of chiral hybrids of γ-butenolide and oxindole (CHBO). The authors have also shown the medicinal chemistry application of the final products. A lot of work related to methodology development and mechanistic investigations is described in this manuscript. This work therefore can be considered for publication in ACS Central Sci.

The following are some comments that might help authors to improve the readability of the manuscript.

Major comments

1) The introduction of the manuscript starts with VS followed by PTPB1 discussion. However, after reading the manuscript it seems like neither VS nor the target is the major focus of the work. The CHBOs are not synthesized keeping in mind the target or VS applications.

Therefore, it is better to provide more information about the MCRs and their advantages in medicinal chemistry. Why is it important to develop strategies like MMCRs and what gaps can it fill in the field of drug discovery. Some recent literature can be cited where MMCRs/MCRs have found applications in providing novel scaffolds or efficient synthesis drug or lead molecules or diverse libraries for VS.

2) Page 2, col 2, line 38*, " First, we intend to search for a catalyst system to control the chemo- and stereoselectivity in the zwitterion capture process"*

The context of this sentence is not clear. There should be some discussion/explanation about zwitterion capture before this together with citations.

3) Discussion of screening of condition screening in the form of Figure 3 is confusing

For e.g. In this sentence *"Feng-ligand L7 gave 18% ee, while the other ligands resulted in 0-6% ee. Interestingly, the rate and ee of the reaction was obviously improved if Mg(OTf)2 was used as a co-catalyst"*

It is not clear which result the authors are talking about in Figure 3. Similar confusion arises from other sentences in the absence of the Table. The authors should provide the Table with different entries for the conditions and the results and cite these in the text. Currently, this data is given in supplementary information but not cited in the manuscript at all. It would help if this Table can be provided in the main manuscript, at least with the most important entries.

4) Page 4, col 2, line 53, *"Overall, the recipe of the reaction was…. 4 hours"*

This final optimized condition should be somewhere in Figure 3, may be last row.

5) It is not clear why the authors selected specifically PTPB1 out of the thousands of available targets. In silico screening could have been done of any target. Do the structures of CHBOs bear any similarities with the known ligands? The rationale for selecting the target should be discussed briefly.

6) It is not clear how many molecules were used for the in-silico screening and what was the criteria to select the hits for experimental validation. Structures of other hits should also be provided with supplementary information. Was only one molecule **3ak** tested against the target?

7) Since the authors have already synthesized several CHBOs, the screening of closely related analogues of **3ak** can be performed to provide some SAR information.

8) The Autodock vina version and protein preparation method for docking studies should be provided.

Other comments: There are several typographical errors. A few are given below.

- Page 1, col 2, line 31 and 32, use only surname of authors before et al.
- Page 2, col 1 line 45 'substracte'
- Page 2, col 1, line 45, this sentence is not clear and needs to be rewritten "whereas the …………….library"

- Page 2, col 2, line 38, “ We started from the reaction of cyclopropene carboxylic acid (CCA) and
- istatins which gave good diastereoselectivity in our previous work”. Need citation at the end of this sentence.
- Page 3, col 1, line 52, ‘temperate reaction’, is it ‘template’??
- Page 3, col 1, line 57, correct “L4 is lack of reactivity......... initiation of the reaction”
    - Page 8, col2, line 30, correct typo “developed”

Reviewer: 2

Comments to the Author

The manuscript describes the development of a multicatalysis-enabled asymmetric multicomponent cascade reaction (MMCR) to synthesize chiral hybrids of gamma-butenolide and oxindole (CHBOs) with exceptional chemo-, diastereo-, and enantioselectivity. This methodology enabled the rapid generation of a diverse library of 50 CHBOs, facilitating the discovery of a potent protein tyrosine phosphatase 1B (PTP1B) inhibitor, (S,S)-3ak, with a sub-micromolar IC50 value. Experimental and computational studies elucidated a complex cascade mechanism involving cyclopropenation, desilylation, cycloisomerization, Aldol-type addition, and hydrolysis. The significance of chirality was highlighted through molecular docking, showing that (S,S)-3ak exhibits superior inhibitory activity compared to its enantiomer. This research bridges synthetic methodology and drug discovery, contributing to developing PTP1B-based cancer immunotherapeutics.

It is worth mentioning that the characterizations were well-conducted, with no issues observed in the spectra or other data. Considering the work's impact, significance, and broad appeal, I recommend publication after minor revisions.

1- Figure 4e (DFT calculations): It would be more effective to display the reaction pathway along with the calculated structures and their relative energies to provide a clearer visualization of the transformation.

2- Water played a role in the transformation, and other H-donors were subsequently tested. However, water itself was not tested in excess. If feasible, please include this experiment.

3- This MMCR methodology presents several advantages, as highlighted in the work. However, no limitations are discussed. Addressing this aspect would be important, even if only to outline potential research directions for future studies.

Reviewer: 3

Comments to the Author

On first impression, this manuscript looks very impressive – virtual screening to identify a hit as a potential inhibitor for PTP1B, the previous approach to the target was not sufficiently flexible, so a new multi component multi catalytic process was developed, and the resulting compounds have promising activity. The work has some positive components but the way it has been presented does not take into account what this group has done previously and ignores related work by others. This manuscript in its current form is not acceptable and needs to be totally rewritten with the work put in proper context. Below are some of the most serious problems.

Refs 9-11 describe the background literature on PTP1B inhibitors. However, they have failed to acknowledge that they have been involved in studies on PTP1B inhibitors since 2016 and many of the previous compound they have evaluated have are also isoindole derivatives. The relationship of this current work to their previous studies on PTP1B inhibitors should have been made clear

In the introduction there is an extensive discussion about virtual screening and so there is a suggestion that the new target was identified through virtual screening. However, there is little description about how the virtual screening was conducted and considering these compounds have features similar to the PTP1B inhibitors they had previously studied, one wonders whether the lead development required identification by an extensive virtual screening.

The synthetic sequence they have developed is impressive involving cyclopropane formation, followed by a ring expansion of the cyclopropane followed by a reaction with

isatin to form the desired product. There are issues of diastereoselectivity and enantioselectivity that needed to be overcome. In the introduction to their synthetic approach they state “As far as we know, however, only two examples of asymmetric synthesis of CHBOs have been reported via metal 14- or organocatalysis 15.” They do not indicate that ref 14 is their work and uses a related strategy to what they are doing here. They have done some very nice optimization studies in this manuscript but the relationship to the earlier work should have pointed out.

Afterwards they explore whether the cyclopropene formation could be generated and reacted further in a one pot procedure. As an introduction they state “In previous studies, alkynes are usually converted to cyclopropenes with 2-3 steps transformations, and then the isolated cyclopropenes are used as versatile substrates in many useful reactions,” They use the cyclopropanation of alkynes with aryldiazoacetates as a one-step procedure but this is a very well established procedure. The asymmetric entry into various cyclopropenes using aryldiazoacetates was first reported in 2004 (Org. Lett., 2004, 6, 1233-1236) and they have generated the same cyclopropane in their own recent publications (for example, Green Synthesis and Catalysis, Volume 5, Issue 3, August 2024, Pages 180-185). In Fig 6, they describe how the cyclopropenation can be extended to a range of aryldiazoacetates and alkynes leading to a variety of compounds after rearrangement. However, this is the cyclopropenation that was done asymmetrically in Org. Lett., 2004, 6, 1233-1236 and this work was not referenced.

In conclusion, this manuscript needs a severe rewrite and the background literature needs to be properly discussed. The enantioselective cascade sequence that was optimized is a nice chemical accomplishment and so it could be worth re-evaluating after the revision. If the paper is revised, it would be worth having some help from a native English speaker because the manuscript contains numerous idiomatic errors.

Reviewer: 4

Comments to the Author

In this manuscript, the authors developed a multicatalysis-promoted multicomponent cascade reaction (MMCR) of diazoesters, alkynes, isatins, and water to efficiently synthesize CHBOs with a broad substrate scope. The effect of substrate, (co)catalyst system, solvent on the cascade reaction, as well as the mechanism were fully investigated. Overall, this is a solid work with regards to synthesize enantiopure CHBOs. I recommend its publication after revisions. Below are some specific comments.

1. In page 3, line 52, “temperate” should be corrected as “template”.

2. Why did the solvent have a significant effect on the reaction of CCE 4a and isatin 2a? What about other solvents with H-bonding donors?

3. Is there any guiding principle for screening the catalyst system, such as the transition metal catalysts?

4. Please check the format of the references carefully.

5. The graphical abstract is of low resolution, it is recommended to change to a clearer version.

Author's Response to Peer Review Comments:

Response to editorial board:

1. ABSTRACT WORD COUNT: Please make sure the word count of your Abstract does not exceed 200 words.

Answer: Abstract has been revised to less than 200 words.

2. SI STATEMENT: If the manuscript is accompanied by any Supporting Information for Publication, a brief description of the supplementary material is required in the manuscript, before the reference list. The appropriate format is: Supporting Information.
   Brief statement in non-sentence format listing the contents of the material supplied as Supporting Information. Please list each supporting item individually.

*Examples of sufficient descriptions: “Supporting Information: 1H NMR spectra for all compounds” or “Additional experimental details, materials, and methods, including photographs of experimental setup.”

*Examples of insufficient descriptions: “Supporting Information: Figures S1-S3” or “Additional figures as mentioned in the text.”

SYNOPSIS LENGTH: The synopsis should be no more than 200 characters (including spaces).

SI GRAPHICS: Please label all graphics in the Supporting Information file in the following format: “Figure S1, S2…”, “Scheme S1, S2….” or “Table S1, S2…”, etc.

Answer: SI section has been revised according to the guidance.

Response to reviewers:

Reviewer 1

1) The introduction of the manuscript starts with VS followed by PTPB1 discussion. However, after reading the manuscript it seems like neither VS nor the target is the major focus of the work. The CHBOs are not synthesized keeping in mind the target or VS applications. Therefore, it is better to provide more information about the MCRs and their advantages in medicinal chemistry. Why is it important to develop strategies like MMCRs and what gaps can it fill in the field of drug discovery. Some recent literature can be cited where MMCRs/MCRs have found applications in providing novel scaffolds or efficient synthesis drug or lead molecules or diverse libraries for VS.

   Answer: Thank you for your helpful suggestion! We rewrote the induction by following your advice. We feel the logic flow gets much clearer than the previous version after taking in your wonderful thoughts on the section.

2) Page 2, col 2, line 38, “ First, we intend to search for a catalyst system to control the chemo- and stereoselectivity in the zwitterion capture process” The context of this sentence is not clear. There should be some discussion/explanation about zwitterion capture before this together with citations.

Answer: Thank you for your kind advice, we added the introduction of “highly reactive intermediate capture process” in the introduction (Fig. 1b) and cited the related references.

3) Discussion of screening of condition screening in the form of Figure 3 is confusing For e.g. In this sentence “Feng-ligand L7 gave 18% ee, while the other ligands resulted in 0-6% ee. Interestingly, the rate and ee of the reaction was obviously improved if Mg(OTf)2 was used as a co-catalyst” It is not clear which result the authors are talking about in Figure 3. Similar confusion arises from other sentences in the absence of the Table. The authors should provide the Table with different entries for the conditions and the results and cite these in the text. Currently, this data is given in supplementary information but not cited in the manuscript at all. It would help if this Table can be provided in the main manuscript, at least with the most important entries.

Answer: Thank you for your great advice, we redrew the table for the condition screening section. Please see table 1. We believe the revised description on the section gets clear by taking your advice.

4) Page 4, col 2, line 53, “Overall, the recipe of the reaction was…. 4 hours” This final optimized condition should be somewhere in Figure 3, may be last row.

Answer: Yes, dear reviewer, the final optimized condition was listed in the table 1, entry 25 and was cited in the text.

5) It is not clear why the authors selected specifically PTPB1 out of the thousands of available targets. In silico screening could have been done of any target. Do the structures of CHBOs bear any similarities with the known ligands? The rationale for selecting the target should be discussed briefly.

Answer: Upon your concern on the rationale for selecting PTP1B as the target, we added one paragraph to explain this point (Page 1, line 21-page 2, line 64).

6) It is not clear how many molecules were used for the in-silico screening and what was the criteria to select the hits for experimental validation. Structures of other hits should also be provided with supplementary information. Was only one molecule 3ak tested against the target?

Answer: Thank you for your concern on the in-silico screening! we screened the MREAL database with around 3000 scaffold-diversified molecles against PTP1B via molecular docking, prioritizing compounds with docking scores $\Delta G < -7.0$ kcal/mol and reasonable binding pose. This led to the identification of four promising scaffold classes: 2, 5-dihydrofurans (2HF), tetrahydrocarbolines (THCB), oxindole-branch (OB) and *rac*-CHBOs (Supporting information, scheme S6).

7) Since the authors have already synthesized several CHBOs, the screening of closely related analogues of 3ak can be performed to provide some SAR information.

Thank you for your kind suggestion on the SAR, we included the SAR information by adding table 2 and paragraph in Page 11, line373-397.

8) The Autodock vina version and protein preparation method for docking studies should be provided.

Thank you for your advice! And the detailed procedure of in-silico screening was provided in the supporting information, page S191.

Other comments: There are several typographical errors. A few are given below.

- Page 1, col 2, line 31 and 32, use only surname of authors before et al.
- Page 2, col 1 line 45 ‘substracte’

- ➢ Page 2, col 1, line 45, this sentence is not clear and needs to be rewritten “whereas the ……………..library”
- ➢ Page 2, col 2, line 38, “ We started from the reaction of cyclopropene carboxylic acid (CCA) and istatins which gave good diastereoselectivity in our previous work”. Need citation at the end of this sentence.
- ➢ Page 3, col 1, line 52, ‘temperate reaction’, is it ‘template’??
- ➢ Page 3, col 1, line 57, correct “L4 is lack of reactivity………. initiation of the reaction”
- ➢ Page 8, col2, line 30, correct typo “developed

Answer: Thank you so much for your patience to correct our typos and mistakes. We apologize for not doing it right. Now these mistakes have been corrected in the current manuscript.

Reviewer: 2

1- Figure 4e (DFT calculations): It would be more effective to display the reaction pathway along with the calculated structures and their relative energies to provide a clearer visualization of the transformation.

   Answer: Thank you for your kind suggestion! We have reorganized the figure to put the reaction pathway along with the calculated structures (Fig. 4). We highlighted the relative energies and structures of some important intermediates and transitional state.

2- Water played a role in the transformation, and other H-donors were subsequently tested. However, water itself was not tested in excess. If feasible, please include this experiment.

   Answer: Thank you for your insightful suggestion! The excess water in the reaction does not affect the yield or enantioselectivity until it reaches a level where it becomes the solvent. At that point, phase separation occurs, isolating the catalysts and substrates into different phases and resulting in no desired product.

3- This MMCR methodology presents several advantages, as highlighted in the work. However, no limitations are discussed. Addressing this aspect would be important, even if only to outline potential research directions for future studies.

Answer: Thank you for your considerate suggestion! The limitations have been discussed in Line 335-240. “However, achieving the reactivity of other cyclopropene derivatives, such as amides, thioesters, and acceptor-acceptor-type cyclopropenes, requires advanced catalytic strategies. Additionally, the reactivity of other carbonyl electrophiles, including aldehydes and

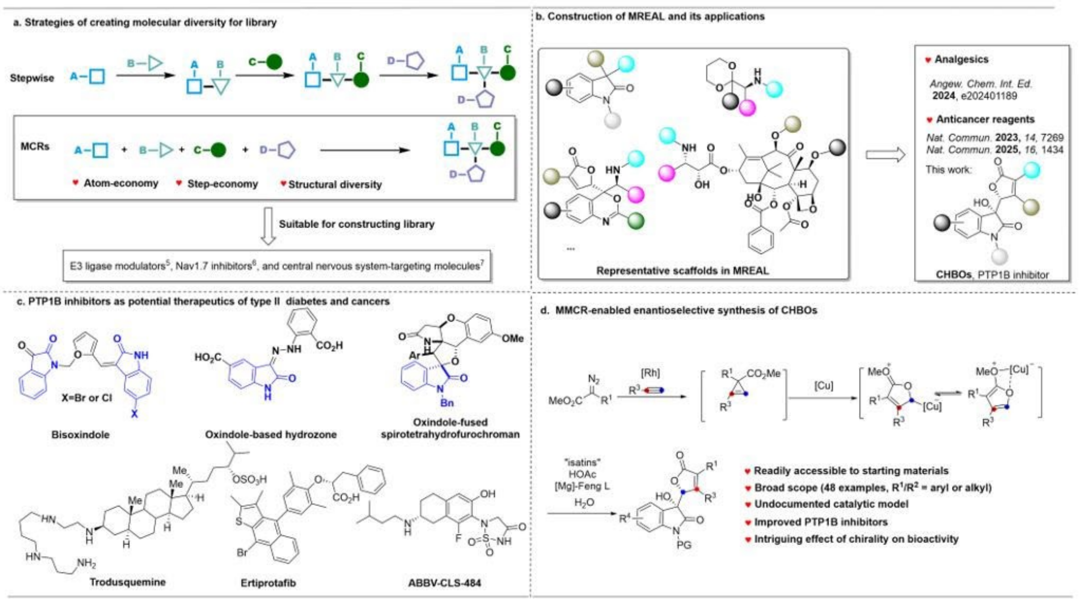

unactivated ketones, in the MMCR still requires in-depth investigation."

Reviewer: 3

1. Refs 9-11 describe the background literature on PTP1B inhibitors. However, they have failed to acknowledge that they have been involved in studies on PTP1B inhibitors since 2016 and many of the previous compound they have evaluated have are also isoindole derivatives. The relationship of this current work to their previous studies on PTP1B inhibitors should have been made clear

Answer: Thank you for your insightful suggestion! We have revised the section according to your comments. The Fig.1 is completely redrawn to demonstrate the background of PTP1B inhibitors and the relationship between the current CHBO PTP1B inhibitors and the previous ones.

2. In the introduction there is an extensive discussion about virtual screening and so there is a suggestion that the new target was identified through virtual screening. However, there is little description about how the virtual screening was conducted and considering these compounds have features similar to the PTP1B inhibitors they had previously studied, one wonders whether the lead development required identification by an extensive virtual screening.

Answer: Thank you for your critical suggestion! The introduction has been rewritten by focusing on the Multicomponent reactions and their applications in drug discovery. The detailed virtual screening procedures has been presented in the supporting information.

In addition, we did discover PTP1B inhibitors containing oxindole scaffold in the past. With this fact, it is reasonable to question the necessity of extensive virtual screening. But when we went to screen those oxindole-bearing compounds *in virtual* or *in vitro* against PTP1B and other targets, the bioactivities

surprisingly varied. We think the shape of the whole molecule may dictate the pocket-fit, and the oxindole scaffold alone may not be able to determine bioactivity.

3. The synthetic sequence they have developed is impressive involving cyclopropane formation, followed by a ring expansion of the cyclopropane followed by a reaction with isatin to form the desired product. There are issues of diastereoselectivity and enantioselectivity that needed to be overcome. In the introduction to their synthetic approach they state “As far as we know, however, only two examples of asymmetric synthesis of CHBOs have been reported via metal 14- or organocatalysis 15.” They do not indicate that ref 14 is their work and uses a related strategy to what they are doing here. They have done some very nice optimization studies in this manuscript but the relationship to the earlier work should have pointed out.

Answer: Thank you for pointing out this. The ref14 using chiral Sc-N,N’-dioxide complex to control the enantioselectivity does inspire us to use chiral Mg- N,N’-dioxide complex to achieve asymmetric catalytic synthesis of CHBOs via MMCR. And the catalysis relationship has been pointed out in Line115-119.

4. Afterwards they explore whether the cyclopropene formation could be generated and reacted further in a one pot procedure. As an introduction they state “In previous studies, alkynes are usually converted to cyclopropenes with 2-3 steps transformations, and then the isolated cyclopropenes are used as versatile substrates in many useful reactions,” They use the cyclopropanation of alkynes with aryldiazoacetates as a onestep procedure but this is a very well established procedure. The asymmetric entry into various cyclopropenes using aryldiazoacetates was first reported in 2004 (Org. Lett., 2004, 6, 1233-1236) and they have generated the same cyclopropane in their own recent publications (for example, Green Synthesis and Catalysis, Volume 5, Issue 3, August 2024, Pages 180-185). In Fig 6, they describe how the cyclopropenation can be extended to a range of aryldiazoacetates and alkynes leading to a variety of compounds after rearrangement. However, this is the cyclopropenation that was done asymmetrically in Org. Lett., 2004, 6, 1233-1236 and this work was not referenced.

Answer: Thank you for pointing these issues out for us! The references you mentioned and other cyclopropenation-related references have been added in line158-160: “cyclopropenes is well-established by the Davies group via the cyclopropenation between alkynes and carbenoids[35-39]”

Reviewer: 4

1. In page 3, line 52, “temperate” should be corrected as “template”.

Answer: Thank you for your careful checking on the spelling! The word “temperate” has been corrected “template”.

2. Why did the solvent have a significant effect on the reaction of CCE 4a and isatin 2a? What about other solvents with H-bonding donors?

Answer: Thank you for your concern on the solvent effect. We think the solubility of the substrates and the catalyst in solvent, and the weak coordination ability of the solvent are important. We went to test other H-bonding donors like MeOH according to your suggestion, and found the reactivity and selectivity dropped significantly (Table 1, entry 17). We propose that the MeOH can strongly coordinate with $Mg(ClO_4)_2$ and reduced the catalytic efficiency of the chiral Mg-N,N'-dioxide complex.

3. Is there any guiding principle for screening the catalyst system, such as the transition metal catalysts?

Answer: Thank you for your kind question on the guiding principle for screening the catalyst system. Transition metal catalyst like $CuBF_4(CH_3CN)_4$ is responsible for the rearrangement of cyclopropane, and the $Mg(ClO_4)_2$ is responsible for activation of carbonyl group of isatin. We think the hard-soft-acid-base (HSAB) theory could guide the selection of catalysts. The discussion is in the line117-120: "Mg-ligands complexes may outcompete Cu-ligands in interacting and activating carbonyl groups according to hard- soft- acid-base theory (HSAB)".

4. Please check the format of the references carefully.

Answer: Thank you for your advice on the references formatting. We have reformatted all the references according the requirements of the ACS Central Science.

5. The graphical abstract is of low resolution, it is recommended to change to a clearer version.

Answer: Thank you for pointing out the resolution issue of TOC. The resolution has been improved.

oc-2025-000414.R2

Name: Peer Review Information for "Multicatalysis-Enabled Multicomponent Reactions Generate A PTP1B Inhibitor"

Second Round of Reviewer Comments

Reviewer: 1

Comments to the Author

The authors have addressed my comments and improved the readability of the manuscript. The revised manuscript can be published.

Author's Response to Peer Review Comments:

Dear editors,

Thank you so much for your suggestions on revising the manuscript.

The changes have been made accordingly. There are 5 files will be uploaded, including

1. ACS-CentralScience-V3

2.ACS-CentralScience-V3-tracked

3.SI-ACS-CentralScience-V3

4. Cover Art Caption

5. Cover Art Pic

Best Reagards,

Taoda
